# Analysis of Expression Pattern of snoRNAs in Different Cancer Types with Machine Learning Algorithms

**DOI:** 10.3390/ijms20092185

**Published:** 2019-05-02

**Authors:** Xiaoyong Pan, Lei Chen, Kai-Yan Feng, Xiao-Hua Hu, Yu-Hang Zhang, Xiang-Yin Kong, Tao Huang, Yu-Dong Cai

**Affiliations:** 1 College of Life Science, Shanghai University, Shanghai 200444, China; xypan172436@gmail.com; 2 Department of Medical Informatics, Erasmus MC, 3015 CE Rotterdam, The Netherlands; 3 College of Information Engineering, Shanghai Maritime University, Shanghai 201306, China; chen_lei1@163.com; 4 Shanghai Key Laboratory of PMMP, East China Normal University, Shanghai 200241, China; 5 Department of Computer Science, Guangdong AIB Polytechnic, Guangzhou 510507, China; addland@126.com; 6 Department of Biostatistics and Computational Biology, School of Life Sciences, Fudan University, Shanghai 200438, China; xhhu@fudan.edu.cn; 7 Shanghai Institute of Nutrition and Health, Shanghai Institutes for Biological Sciences, Chinese Academy of Sciences, Shanghai 200031, China; zhangyh825@163.com

**Keywords:** snoRNA, cancer type, Monte Carlo feature selection, support vector machine, RIPPER algorithm

## Abstract

Small nucleolar RNAs (snoRNAs) are a new type of functional small RNAs involved in the chemical modifications of rRNAs, tRNAs, and small nuclear RNAs. It is reported that they play important roles in tumorigenesis via various regulatory modes. snoRNAs can both participate in the regulation of methylation and pseudouridylation and regulate the expression pattern of their host genes. This research investigated the expression pattern of snoRNAs in eight major cancer types in TCGA via several machine learning algorithms. The expression levels of snoRNAs were first analyzed by a powerful feature selection method, Monte Carlo feature selection (MCFS). A feature list and some informative features were accessed. Then, the incremental feature selection (IFS) was applied to the feature list to extract optimal features/snoRNAs, which can make the support vector machine (SVM) yield best performance. The discriminative snoRNAs included HBII-52-14, HBII-336, SNORD123, HBII-85-29, HBII-420, U3, HBI-43, SNORD116, SNORA73B, SCARNA4, HBII-85-20, etc., on which the SVM can provide a Matthew’s correlation coefficient (MCC) of 0.881 for predicting these eight cancer types. On the other hand, the informative features were fed into the Johnson reducer and repeated incremental pruning to produce error reduction (RIPPER) algorithms to generate classification rules, which can clearly show different snoRNAs expression patterns in different cancer types. The analysis results indicated that extracted discriminative snoRNAs can be important for identifying cancer samples in different types and the expression pattern of snoRNAs in different cancer types can be partly uncovered by quantitative recognition rules.

## 1. Introduction

Small nucleolar RNAs (snoRNAs) are a group of functional small RNAs that mainly participate in the chemical modifications of other functional RNAs, such as rRNAs, tRNAs, and small nuclear RNAs [1,2,3]. Generally, snoRNAs may participate in two major biological functions, namely, 2′-*O*-ribose methylation and pseudouridylation of pre-rRNAs [4,5]. snoRNAs interact with at least four protein molecules; thus, snoRNAs form a complicated RNA/protein complex for further modification processes [6,7]. For the detailed recognition on the functional RNA molecules, snoRNAs has a specific antisense nucleotide element containing 10–20 nucleotides that complementary pair with the targeted pre-RNA molecules [8]. After the precise identification and localization processes mediated by this antisense element, the four interacted protein molecules around snoRNAs are located at the correct physical position and contribute to the chemical modification of the target bases [7].

The basic biological processes of snoRNAs are relatively similar based on the objective modification pattern of pre-rRNAs; thus, snoRNAs can be classified into two major subtypes, namely, C/D and H/ACA boxes [9,10]. The C/D box snoRNAs have two major boxes, namely, C (RUGAUGA, R=purine) and D (CUGA), playing different biological roles during the modification targets of our snoRNPs [11]. They guide the position-specific 2′-*O*-methylation and are associated with the four evolutionarily conserved proteins, fibrillarin (methyltransferase), NOP56/NOL5A, NOP5/NOP58, and NHP2L1, which constitute the core of C/D snoRNPs. These motifs of snoRNAs and related RNPs are mainly involved in the methylation regulation of the targeted pre-rRNAs. According to recent publications [11,12], the direct methylation modification sites of C/D snoRNAs are located precisely 5 bp upstream of the D box of snoRNPs, reflecting the accurate positioning and effective regulatory contribution of snoRNAs.

Different from C/D snoRNAs, H/ACA box snoRNAs have two specific hairpins and two short single-stranded regions (H and ACA boxes), from which its cluster name was derived [13]. H/ACA snoRNAs direct RNA pseudouridylation of rRNA and are associated with dyskerin (pseudouridine synthase), GAR1, NHP2, and NOP10. Similar to D box in the C/D box snoRNP complexes, H and ACA boxes also do not only participate in the pseudouridylation modification of pre-rRNAs but more critically contribute to the precise recognition of the target uridylation sites of pre-rRNAs [14]. With the development of biological technologies and related studies, some novel snoRNA subgroups, such as composite H/ACA and C/D boxes and orphan snoRNAs, with relatively different biological functions have been constantly identified [15,16]. However, these groups of snoRNAs either have similar function pattern (e.g., composite H/ACA and C/D boxes), with general groups or have no validated experimental evidence on their specific biological functions (e.g., orphan snoRNAs). Therefore, on the basis of the existing literature, researchers conclude that snoRNAs with different functional subgroups mainly participate in the regulation of pre-rRNA methylation and pseudouridylation.

As a subgroup of noncoding RNAs, snoRNAs was also recently confirmed to involve in tumorigenesis in various regulatory modes as reported in recent publications [17,18,19,20]. First, snoRNAs participate in the regulation of methylation and pseudouridylation. Early studies confirmed that some biological components of snoRNPs, such as SNORA42 and U50, may have specific expression pattern or directly participate in tumorigenesis, indicating that snoRNPs may be functionally related to cancer [20,21]. Second, snoRNAs may also be functionally connected to cancer through its host genes. A recent study on Zfas1 on a tumor-associated nonprotein-coding snoRNA host gene confirmed that three C/D box snoRNAs might regulate the expression of their host gene Zfas1, and further indirectly mediate tumorigenesis [22]. Furthermore, a study on another gene named GAS5 confirmed that snoRNAs (C/D box) encoded by its own intron may contribute to the progression of tumorigenesis similar to its host gene, GAS5 [23]. Although confirmation is still needed on the topic of whether the snoRNA functions are independent of their respective host genes, both snoRNAs and the host genes may promote tumorigenesis in a cooperative and synergetic pattern, implying the complicated contribution of snoRNAs on tumorigenesis.

Thus, as we have analyzed above, snoRNAs have been confirmed to contribute to the tumorigenesis in their specific ways. On the other hand, several studies have been reported that miRNAs/lncRNAs are highly related to different diseases [24,25,26,27,28], including tumor, which gives a strong hint for the associations between snoRNAs and different tumors. The analysis of the expression pattern on them is an essential way. This study gave a computational investigation of the snoRNA expression pattern of different tumors. Recently, a systematic study [29] on the distribution of different snoRNAs in different tumor subtypes based on TCGA database has been presented, drawing a detailed blueprint of snoRNAs during tumorigenesis. According to the dataset provided by such study, we firstly screened out the specific expression pattern of snoRNAs in eight candidate tumor subtypes. Then, based on some powerful machine learning algorithms, we tried to screen out the core distinctive distributed snoRNAs among such eight candidate cancer subgroups and further established a qualitative snoRNA-based recognition standard for further tumor subtyping.

## 2. Results

### 2.1. Results of MCFS Method

In this study, we used the Monte Carlo feature selection (MCFS) to analysis expression levels of 1524 snoRNAs. Each feature was assigned a relative importance (RI) score and all features were ranked in descending order based on their RI scores. The obtained feature list is listed in Table S1.

**Figure 1:**
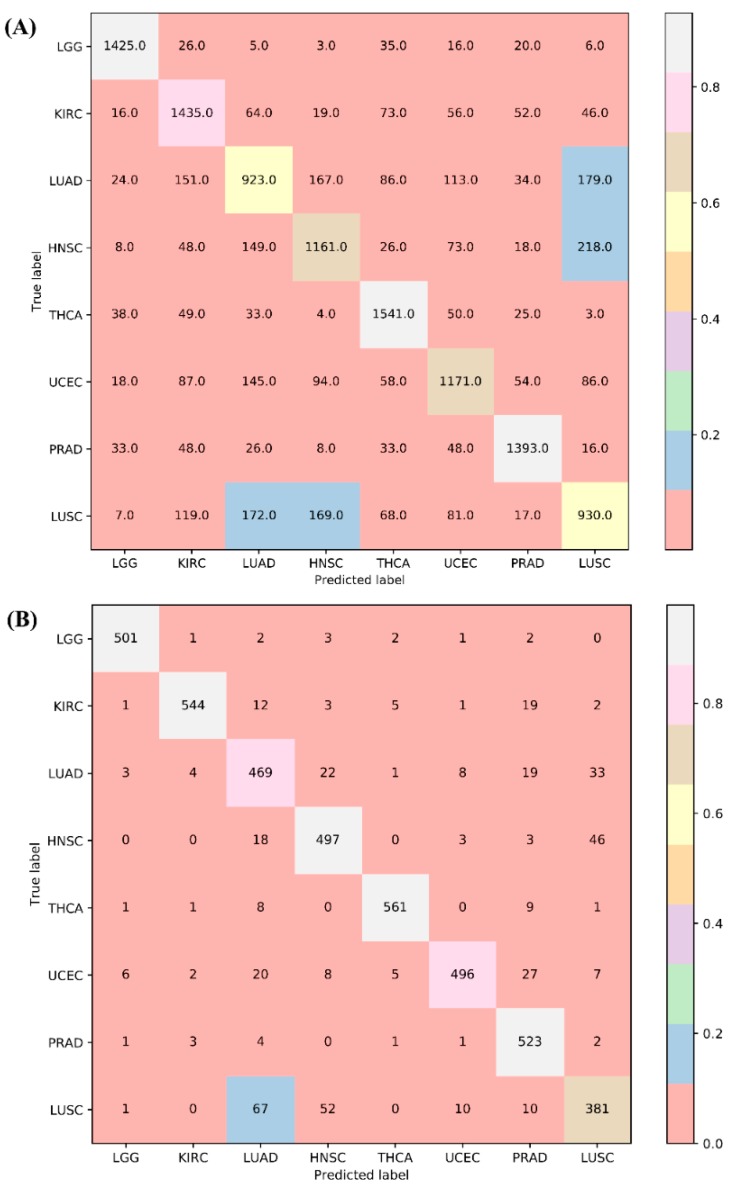
Confusion matrices for 10-fold cross-validation based on two classifiers. (**A**) The confusion matrix yielded by the 69 produced classification rules for classifying samples from 8 cancer types. The numbers were pooled by running 10-fold cross-validation on the training data thrice. (**B**) The confusion matrix yielded by the optimal support vector machine (SVM) classifier. The numbers were pooled by running 10-fold cross-validation on the training data once.

Furthermore, the MCFS method can generate some informative features. Here, 411 features were produced for the problem addressed in this study. Based on them, 69 classification rules were produced by the Johnson reducer algorithm and the repeated incremental pruning to produce error reduction (RIPPER) algorithm, which are listed in Table S2. To evaluate the performance of the rules yielded by the above two algorithms, 10-fold cross-validation was performed thrice, yielding the predicted accuracy of 0.750 and weighted accuracy of 0.751. The true positive rates (TPRs) and false positive rates (FPRs) of the individual classes are shown in Table 1. The confusion map is illustrated in Figure 1A.

### 2.2. Results of IFS with SVM

**Figure 2:**
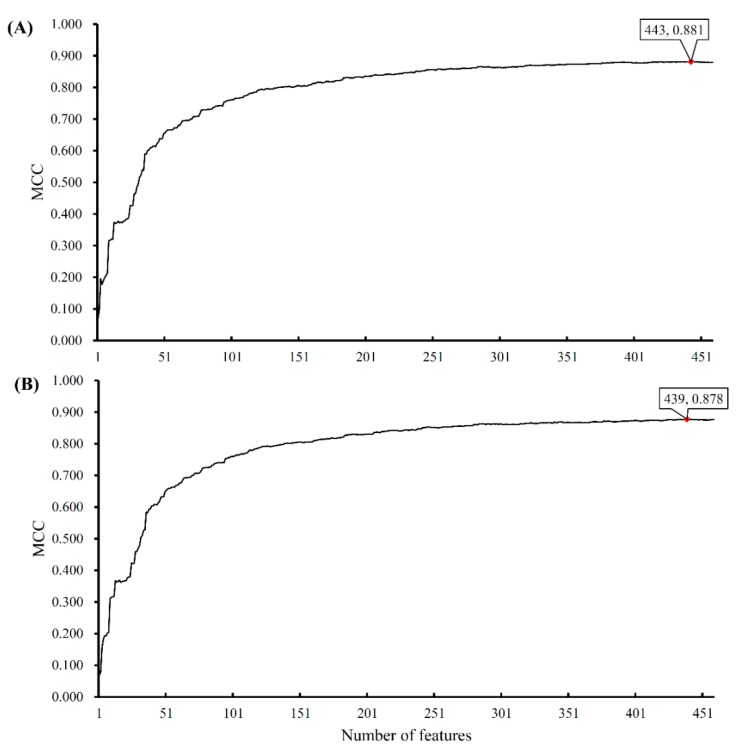
IFS curves derived from the IFS method and SVM classifiers. The x-axis is the number of features involved in building classifiers, while the y-axis is their corresponding Matthew’s correlation coefficient (MCC) values. (**A**) The IFS curve yielded by 10-fold cross-validation. (**B**) The IFS curve yielded by 5-fold cross-validation.

We also used support vector machines (SVMs) to classify samples consisting of important features that were selected from the incremental feature selection (IFS) method. First, a set of feature subsets with a step 1 were constructed. After testing the performance of SVMs on the samples consisting of features from individual feature subsets with 10-fold cross-validation once, we obtained the highest Matthew’s correlation coefficient (MCC) of 0.881 when the top 443 snoRNAs were used. In addition, we can yield an MCC value of 0.708 when using only the top 72 features. The predicted accuracies for individual cancers, overall accuracies, and MCCs by using a different number of features are listed in Table S3. Furthermore, we presented the MCC trends corresponding to the number of features involved in building the SVM classifiers, as shown in Figure 2A, in which the optimal MCC value of 0.881 is marked with a red rhombus. Accordingly, we termed the SVM classifier using top 443 features as the optimal SVM classifier. The confusion matrix generated by the 10-fold cross-validation on this classifier is illustrated in Figure 1B, from which we can see that the performance of the optimal SVM classifier was much better than that of 69 classification rules. The TPRs and FPRs of the individual classes yielded by such a classifier are listed in Table 1. Compared with those yielded by classification rules, the TPRs produced by the optimal SVM classifier were much higher and FPRs were lower (except one), suggesting the optimal SVM classifier gave a much better performance. In addition, we also counted the performance of the optimal SVM classifier on each of the ten folds. The highest and lowest accuracies for each cancer type were listed in Table 2.

Furthermore, we also used a 5-fold cross-validation to replace the 10-fold cross-validation in the above procedures. The MCC trends corresponding to the number of used features are shown in Figure 2B. The trends of MCC in the two curves were almost the same. The performance yielded by the 5-fold cross-validation was slightly lower than that obtained by the 10-fold cross-validation. It is reasonable because, in a 5-fold cross-validation, fewer samples were used to train the classifiers.

## 3. Discussion

On the basis of some machine learning methods, we identified not only a group of effective core regulatory snoRNAs that may participate in tumorigenesis and contribute to the distinction of the eight candidate tumor subtypes, but we also set up a series of qualitative rules for the precise recognition of different subtypes according to the expression pattern of the optimal parameters. According to recent publications, some obtained key distinctive snoRNAs and specific distinctive qualitative rules can be verified, validating the reliability of our results.

As we have mentioned above, snoRNAs are a group of small nucleolar RNAs that generally contribute to the regulation of RNA modifications. Ribosomal RNAs, transfer RNAs, and small nuclear RNAs are the three subgroups of snoRNAs. SnoRNAs generally act as a guide for specific modification on a target RNA in the form of RNA/protein complex together with multiple protein molecules. Such complexes (also called small nucleolar ribonuceloproteins) further contribute to the accurate chemical modification of a target RNA sequence. When analyzing the biological functions of snoRNAs, we just screened out the targets of snoRNAs and tried to explain the biological effects of these snoRNAs based regulatory roles on such targets, which may help explain the biological function of snoRNAs. Comparing to snoRNAs, miRNAs/lncRNAs do not directly affect the chemical structure and composition of mRNAs. Such RNAs regulate gene functions by affecting gene expression levels directly or indirectly. Thus, when analyzing the biological functions of such two kinds of RNAs, we analyzed the expression alteration effects of features in certain physical or pathological conditions. Therefore, the analysis of miRNAs/lncRNAs and snoRNAs are quite different. In this study, we applied the proper functional analysis on key distinctive snoRNAs and rules associated snoRNAs.

### 3.1. Analysis of Optimal Tumor-Associated snoRNAs

Based on the dataset provided by our reference and the newly applied computational approach, we identified a group of functional snoRNAs that may have distinctive expression pattern in eight tumor subtypes. Here, we presented a detailed analysis of a number of snoRNAs.

The first snoRNA is HBII-52-14, which is a C/D box snoRNA that was first cloned by Cavaill in 2000 [30]. As a transcript that is specifically expressed in the brain, HBII-52-14 may target the serotonin receptor (5HT-2C) in the brain, and regulate its functional methylation status [30,31]. Recent publications confirmed that the expression pattern of 5HT-2C, regulated by our identified snoRNA, is pathogenic and may be involved in the initiation and progression of low-grade glioma (LGG) [32]. This result implies that this parameter may be effective in differentiating glioma from other tumor subtypes. HBII-52-10, HBII-52-15, HBII-52-32, HBII-52-5, and HBII-52-4 have also been confirmed to target serotonin, connecting this regulatory function to the potential glioma tumorigenesis.

In addition to this series of snoRNAs, HBII-336 (with a rank of 3 in the feature list, yielded by the MCFS method) is also validated to contribute to the distinction of different tumor subtypes by recent publications. First described by Huttenhofer in 2001 [33], HBII-336 guides the 2′-*O*-ribose methylation of 18S rRNA A576 [33]. Recent publications also confirmed that the methylation of 18S rRNA may be functionally related to the initiation and progression of certain tumor subtypes, such as breast cancer, colorectal cancer, and renal carcinoma [34,35]. Comparing these findings with those results for the eight tumor subtypes investigated in this study, the expression of HBII-336 can be helpful for tumor subtyping.

HBI-43, as another identified snoRNA has also been confirmed to participate in the distinction of different cancer subtypes. According to recent publications, such snoRNA has been functionally related to a specific effective gene *TRIM25* [36]. Such a gene has been identified to have pathogenic expression pattern in breast cancer [37] and hepatocellular carcinoma [38]. Therefore, it is quite reasonable to speculate that as the regulator of *TRIM25*, the expression pattern of HBI-43 may also be sensitive to distinguish different cancer subtypes.

The next functional cluster of snoRNA is SNORD123 (rank 13), which was first reported by Yang et al. in 2006; moreover, this C/D box snoRNA has been initially predicted and further validated using Northern blot [39]. Although few studies have revealed in detail the potential biological function of SNORD123, a specific publication [40] in 2012 confirmed that this snoRNA may regulate the hypermethylation status of functional CpG islands in specific tumor subgroups, such as colorectal and lung cancer. Therefore, SNORD123 may be one of the significant biomarkers for the identification of LUAD and LUSC. Similar to SNORD123, SNORD19 has also been reported to have a different expression pattern in different tumor subtypes [41], implying its potential capacity for cancer subtyping.

The following snoRNA cluster is HBII-85-29, targeting the antisense sequence of SNURF-SNRNP-UBE3A (transcription unit) [30,42,43]. HBII-85-29 is specifically expressed in the brain and uterus. Thus, it may be functionally connected to LGG and uterine corpus endometrial carcinoma (UCEC). Recent publications confirmed that HBII-85-29 might be functionally connected to the pathogenesis of hypothalamus, further implying the potential relationship between snoRNA HBII-85-29 and LGG [44,45]. Therefore, HBII-85-29 may also be sufficiently specific for further subgrouping of the eight tumor subtypes.

The following identified cancer subtype-contributing snoRNA is also a C/D box snoRNA named HBII-420. HBII-420, which targets a hypothetical protein, was also first identified and validated by Huttenhofer in 2001 [33]. Although limited reports confirmed the contribution of HBII-420 in tumorigenesis, two studies on lung adenocarcinoma [46] and multiple myeloma [47] confirmed that with specific potential pathogenic expression pattern, HBII-420 might be functionally related to these two tumor subtypes. For the eight tumor subtypes in this study, differentiating lung adenocarcinoma from other tumor subtypes using HBII-420 is relatively effective.

Based on the expression pattern of optimal snoRNAs, we concluded that distinguishing eight tumor subtypes by using these snoRNAs is effective and accurate, validating that snoRNAs may also contribute to precise tumor subtyping.

### 3.2. Analysis of Optimal snoRNA-Based Tumor Subtyping Rules

In addition to the qualitative analysis mentioned above, based on the detailed expression level of snoRNA provided by our reference dataset in Section 4.1 [29], we set up a series of systematic quantitative distinctive rules for further detailed identification of each tumor subgroup among the eight cancer types. In total, we obtained 69 rules for all eight tumor subtypes (Table S2). However, due to the limitation of the manuscript, no space can be used to analyze each quantitative rule one by one. Therefore, to display the whole blueprint of these rules, we screened out one typical quantitative rule for each tumor subtype. The detailed analysis is listed below.

According to the quantitative rules listed in Table S2, the first identified tumor subtype is LGG. In the first rule (rule1), three effective parameters, namely, SNORD123, U49B, SNORA1, and U3 have been proposed. According to this rule, the high expression of U3 and low expression of SNORD123 and U94B may indicate that the potential tumor is LGG. According to recent publications, SNORD123 is downregulated in patients with glioma; this phenomenon is associated with personalized prognosis [48]. Moreover, a recent systematic analysis on LGG and high-grade glioma confirmed that U3 and U49B may have identical expression pattern compared with this rule in LGG [49,50].

The first ten rules contribute to LGG identification. However, the eleventh rule (rule11) contributes to LUSC identification, involving four optimal snoRNAs, including hTR, HBI-115, U83B, SNORA47, and ACA31. The high expression of hTR and U83B, together with the low expression of HBI-115, SNORA47, and ACA31, is unique in the snoRNA expression pattern of LUSC. According to a recent clinical study on LUSC progression, the expression of hTR snoRNA promotes tumorigenesis, corresponding with this rule [51]. Meanwhile, HBI-115, SNORA47 [52], and ACA31 all have unique expression patterns in lung cancer, especially in LUSC, according to a study on the early stage of lung cancer; thus, this result validates the unique indicating effects of these snoRNAs on cancer subtyping even at an early stage [52,53].

Removing the LGG and LUSC interference, the next subgroup of rules to be discussed contributes to PRAD identification. Rule22 involves four effective snoRNAs, namely, SNORA7, HBI-43, HBII-295, and HBII-52-32. The high expression of HBII-52-32, together with the low expression of SNORA7 and HBII-295, refers to PRAD recognition. Although no detailed reports have confirmed that these three snoRNAs may directly contribute to PRAD tumorigenesis, recent publications on C/D box snoRNAs confirmed the potential relationship between snoRNAs and prostate tumorigenesis [23]. As for HBI-43, such snoRNA has been identified to have a specific expression pattern in PRAD tumorigenesis and related cancer subtypes [54]. Therefore, SNORA7, HBI-43, HBII-295, and HBII-52-32 may be specific monitoring markers contributing to prostate cancer progression.

The next identified subgroup is LUAD. These rules involve multiple parameters including HBII-52-32, mgU6-47, SNORD115, U81, and SNORD7. Among these parameters, U81 has a relatively high expression level (≥ 451.69). According to recent publications, U81 is related to the invasion and metastatic progression pattern in multiple tumor subtypes [55,56]. The identification of this snoRNA as a potential parameter may reflect the high-grade malignancy of lung adenocarcinoma. In terms of the specificity of this snoRNA combination, all these snoRNAs and their respective patterns have been identified in LUAD; however, no previous reports are available on the distribution of snoRNA in LUAD [46].

In addition to the above-mentioned four tumor subgroups, the next identified tumor subtype is HNSC. We chose several rules involving SNORD116, SNORA73B, SCARNA4, and HBII-85-20, for further analysis. These four snoRNAs are all functional tumor-associated snoRNAs according to recent publications [47,57,58]. In terms of the tissue specificity of HNSC, a recent publication [59] confirmed that SNORD116 is one of the potential transcriptomic signatures for HNSC identification, corresponding to its specific expression pattern in these rules.

The next identified tumor subtype is UCEC. Tumor-associated snoRNAs, such as ACA56 [60], HBII-336 [61], SNORD19 [41], and U19 [56,62], have been screened out using four quantitative parameters for UCEC identification. Among these four parameters, U19 is a relatively unique parameter with expression level higher than 119, indicating its potential role in tumor subtyping. According to recent publications [56,62], snoRNA U19 (SNORA74), as an H/ACA box snoRNA, contributes to the regulation of the famous AKT/mTOR signaling pathway. The expression level of such gene has been screened out and functionally confirmed to participate in multiple tumor subtypes including UCEC and gallbladder cancer [62].

The next predicted tumor subgroup is THCA. We chose an optimal quantitative rule involving two optimal parameters, namely, SNORD123 and SNORD114, for the detailed analysis (see rule65). No direct reports confirmed the specific expression patterns of these two noncoding snoRNAs in THCA. However, a systematic study [63] on all noncoding RNAs in THCA reveals the potential expression tendency of these two parameters in tumor tissues compared with normal controls, corresponding to this rule and partially validating the efficacy and accuracy of our results.

The specific role of snoRNA in KIRC has been widely reported and confirmed [64,65]. According to our rules, the tumor samples of the eight tumor subtypes that do not have corresponding expression pattern with any of the rules we have extracted may be the kidney renal clear cell carcinoma samples.

According to the two discussion subsections presented above, some extracted optimal distinctive snoRNAs and quantitative rules can be validated by recent publications, validating the reliability of our results. Based on the whole blueprint of snoRNA distribution in multiple tumor subtypes as summarized by a systematic study [29], we further screened out the distinctive core snoRNAs and built up quantitative standards for the identification of different tumor subtypes based on the snoRNA expression. This study does not only provide a new tool for tumor subtyping but also deepens the understanding of the different distribution and contribution mechanisms of snoRNAs in various tumor subtypes.

## 4. Materials and Methods

### 4.1. Dataset

As shown in Gong et al. [29], the snoRNAs in cancers were investigated based on miRNA-sequencing data. We downloaded snoRNA expression levels from at http://bioinfo.life.hust.edu.cn/SNORic/download/. Originally, 31 cancer types were considered. However, many types of cancer have a much smaller number of samples than others. To train the cancer type classifier in a supervised way, we only kept the cancer types with sample sizes greater than 500 since trained models cannot generalize well to the classes with a small number of training samples. Given that breast invasive carcinoma had a much greater sample size than others, it was also not included in this study. Finally, we obtained eight major cancer types. The sample sizes of these eight cancer types are shown in Table 3. In total, the expression levels of 1524 snoRNAs were measured in these eight cancer types, and were used to classify different cancer samples. Since the expression levels of many snoRNAs were very low, we kept the 459 detectable snoRNAs with average RPKM (reads per kilobase per million mapped reads) greater than 1 in 8 cancer types to classify different cancer samples.

### 4.2. Feature Selection

snoRNAs may be functionally associated with different cancer types. To identify highly related snoRNAs for different cancer types, the MCFS [66] was first used to rank all available snoRNAs. Then, IFS [67] with a support vector machine (SVM) was further applied to identify important snoRNAs with the strong discriminative power for classifying different cancer types, those snoRNAs are further used to produce classification rules for classifying different cancer samples.

#### 4.2.1. MCFS

Monte Carlo feature selection (MCFS) [66] is used to rank input features based on multiple decision trees. It constructs multiple decision tree classifiers, which are grown from bootstrap samples that are randomly selected from the original training set and feature subsets. MCFS proceeds as follows: it generates *t* feature subset with *m* features that are randomly selected from original *M* features (*m* ≪ *M*, i.e., *m* is much smaller than *M*). For a given feature subset, *p* decision trees are grown on *p* bootstrap sample sets consisting of the features from this feature subset. The above process is repeated *t* times. In total, *t* feature subsets and p⋅t decision trees are obtained. The relative importance (RI) score of each feature is estimated based on the number of times a feature is involved in growing the p⋅t decision trees. In this study, we used the MCFS software package downloaded from http://www.ipipan.eu/staff/m.draminski/mcfs.html. According to the RI scores of features, a feature list was generated in a descending order of RI scores.

In addition, the MCFS method can produce some informative features, which were the features with the RI scores greater than a cutoff value. A permutation test and one-sided student’s *t*-test were performed to determine the cutoff value of RI scores. Features with RI scores higher than this cutoff value were picked up as informative features, which were deemed to be most important for classification.

For detailed description of MCFS, please refer to [66,68]. To date, MCFS is being applied to tackle different biological problems [69,70,71,72,73,74].

#### 4.2.2. IFS

Although the MCFS method can output informative features, a different classifier may need a different number of informative features to yield the best performance. Thus, we used IFS [67] to select important features according to their discriminate power for a given supervised classifier. Given a ranked feature list with *M* features by MCFS, denoted as $F=\left[f_{1},f_{2},\ldots ,f_{M}\right]$, in descending order, IFS first constructed a series of feature subsets, in which each feature subset had one more feature than the preceding one. $f_{1}$ has only the top 1 feature in the ranked feature list, $f_{2}$ has the top 1 and top 2 features, and so on. Then, a selected supervised classifier was used to test the classification performance on the samples consisting of features from each generated feature subset by using a 10-fold cross-validation [75,76,77,78,79,80,81,82]. Finally, we obtained a feature subset with the best performance and called features in such set as the optimal features. Furthermore, the corresponding classifier with features in such a set was called the optimal classifier.

#### 4.2.3. Rule Learning

As mentioned in Section 4.2.1, the MCFS method can extract informative features. Here, we adopted rule learning algorithms to build classification rules. Different from the supervised classifier mentioned in Section 4.2.2, which was always a black-box method, that is, its classification procedures were not clear and interpretable. The classification rules can provide a clear procedure of classification, and they can give more information for understanding the expression pattern differences of snoRNAs in different cancer types.

The procedures for generating rules proceed in two steps: (1) the Johnson Reducer algorithm [83] was applied on informative features to pick up a subset of them, which can have similar classification ability to using all informative features; (2) the rule learning algorithm, RIPPER algorithm [84], was applied on reduced features to extract classification rules. A rule set obtained by RIPPER algorithm describes an interaction between features (the left-hand side of the rule) and the target (the right-hand side of the rule). For example, a rule is denoted as an IF–THEN relationship according to the expression values, as follows: IF snoRNA1 ≥ 6.4 AND snoRNA2 ≥ 4.8, THEN cancer = HNSC. The above procedures for building classification rules were also included in the MCFS software package and we directly used them for further analysis. The RIPPER algorithm used to generate decision rules is described briefly in [85] (see Figure 1 in [85]).

### 4.3. Support Vector Machine (SVM)

SVM [86] is a popular supervised classifier for both linear and nonlinear data, and it is widely applied in many biological problems [76,87,88,89,90,91,92,93,94]. In addition, to be used for classification, SVM is adopted for regression tasks in the recent work [95]. The basic idea of SVM is to find a hyperplane with the maximum margin between two classes. For the nonlinear data, SVM first mapped these data into a linear high-dimensional space using kernel trick [96]. Then, a linear function is fitted in the high-dimensional space. SVM has a perfect solution for binary classification problems. However, many classification problems are multiclass classification. To solve the multiclass classification problem, a one-versus-the-rest strategy was adopted. Given a dataset with *M* classes, multiple binary SVM classifiers are trained for *M* classes, wherein each SVM classifier was trained to separate the sample of one class from the samples of the rest classes. Given a new sample, the one-versus-the-rest strategy assigns the label with the highest probability score among the scores estimated from the multiple binary SVMs.

### 4.4. Performance Measurement

In this study, we first calculated the prediction accuracy of individual classes by using a 10-fold cross-validation [75,97,98,99,100,101,102]. For each class, we calculated the individual TPR and FPR as follows:

(1) $$TPR=\frac{TP}{TP+FN}$$

(2) $$FPR=\frac{FP}{FP+TN}$$

where *TP*/*TN* is the number of correctly predicted positive/negative samples, and *FN*/*FP* is the number of wrongly predicted positive/negative samples.

Only TPR and FPR cannot objectively estimate the prediction performance of a classifier; Matthew’s correlation coefficient (MCC) [103,104,105] was also applied to evaluate the prediction performance. Given *N* samples and *C* classes, the matrix $X={\left(x_{ij}\right)}_{N\times C}$ represents the predicted classes of samples, and $x_{ij}\in \{0,1\}$ is a binary value; $x_{ij}$ is equals to 1 if the sample i is predicted to belong to class j; otherwise, it is 0. The matrix $Y={\left(y_{ij}\right)}_{N\times C}$ was defined as the true classes of *N* samples, where the binary variable $y_{ij}=1$ when the sample i belongs to class j; otherwise, it is 0.

The MCC is defined as follows:

(3) $$MCC=\frac{\mathrm{cov}(X,Y)}{\sqrt{\mathrm{cov}(X,X)\mathrm{cov}(Y,Y)}}=\frac{\sum_{i=1}^{n}\sum_{j=1}^{C}\left(x_{ij}-{\bar{x}}_{j})(y_{ij}-{\bar{y}}_{j}\right)}{\sqrt{\sum_{i=1}^{n}\sum_{j=1}^{C}{\left(x_{ij}-{\bar{x}}_{j}\right)}^{2}\sum_{i=1}^{n}\sum_{j=1}^{C}{\left(y_{ij}-{\bar{y}}_{j}\right)}^{2}}}$$

where ${\bar{x}}_{j}$ and ${\bar{y}}_{j}$ are the mean values of $x_{j}$ and $y_{j}$, respectively.

## Figures and Tables

**Table 1:** Results of 10-fold cross-validation by using 69 produced classification rules and the optimal support vector machine (SVM) classifier.

Cancer Type	Classification Rules		Optimal SVM Classifier	
	TPR ^†^	FPR ^‡^	TPR ^†^	FPR ^‡^
HNSC	0.683	0.040	0.877	0.023
KIRC	0.815	0.046	0.927	0.003
LGG	0.928	0.012	0.979	0.003
LUAD	0.550	0.051	0.839	0.034
LUSC	0.595	0.047	0.731	0.023
PRAD	0.868	0.019	0.978	0.023
THCA	0.884	0.033	0.966	0.004
UCEC	0.684	0.038	0.869	0.006

^†^ True positive rate, ^‡^ False positive rate.

**Table 2:** The lowest and highest accuracies for each cancer type yielded by the optimal SVM classifier on each of ten folds.

Cancer Type	Highest Accuracy	Lowest Accuracy
HNSC	0.930	0.807
KIRC	0.949	0.897
LGG	1.000	0.922
LUAD	0.875	0.786
LUSC	0.808	0.654
PRAD	1.000	0.925
THCA	1.000	0.931
UCEC	0.930	0.789

**Table 3:** Sample sizes of eight major cancer types.

Cancer Type	Name	Number of Samples
HNSC	Head and neck squamous cell	567
KIRC	Kidney renal clear cell carcinoma	587
LGG	Lower grade glioma	512
LUAD	Lung adenocarcinoma	559
LUSC	Lung squamous carcinoma	521
PRAD	Prostate adenocarcinoma	535
THCA	Thyroid carcinoma	581
UCEC	Uterine corpus endometrial carcinoma	571
Total	---	4433

## Supplementary Materials

Supplementary materials can be found at https://www.mdpi.com/1422-0067/20/9/2185/s1. Table S1. The features ranked by their RI values derived from the MCFS method, Table S2. Produced 69 classification rules for classifying samples from the 8 cancer types, Table S3. Corresponding accuracies of individual classes, overall accuracies, and MCCs by using different numbers of features selected by using the IFS method and SVM classifiers.
