# Supplementary material for: Analysis of Expression Pattern of snoRNAs in Different Cancer Types with Machine Learning Algorithms
Source: Int J Mol Sci. 2019 May 2;20(9):2185. doi: 10.3390/ijms20092185 (PMC6539089; doi:10.3390/ijms20092185)
Supplement: Supplementary file 1 [file ijms-20-02185-s001.zip › Table S1.docx]

**Table S1.** The features ranked by their RI values derived from the Monte Carlo feature selection (MCFS) method.

| **Rank** | **Attribute** | **RI** |
| --- | --- | --- |
| 1 | HBII-52-14;ENSG00000199960;SNORD115-14_chr15_25440067_25440148 | 0.3114 |
| 2 | HBII-52-10;ENSG00000201943;SNORD115-10_chr15_25432682_25432763 | 0.3110 |
| 3 | HBII-336;ENSG00000221740;SNORD93_chr7_22896231_22896305 | 0.2682 |
| 4 | HBII-52-15;ENSG00000201679;SNORD115-15_chr15_25442722_25442803 | 0.2518 |
| 5 | HBII-52-32;ENSG00000200949;SNORD115-32_chr15_25474113_25474195 | 0.2503 |
| 6 | HBII-52-42;ENSG00000201143;SNORD115-42_chr15_25492491_25492573 | 0.2353 |
| 7 | HBII-52-5;ENSG00000200503;SNORD115-5_chr15_25423884_25423966 | 0.2267 |
| 8 | HBII-52-6;ENSG00000200812;SNORD115-6_chr15_25425643_25425725 | 0.2258 |
| 9 | HBII-52-4;ENSG00000200680;SNORD115-4_chr15_25421978_25422060 | 0.2228 |
| 10 | HBI-43;ENSG00000212232;SNORD17_chr20_17943352_17943589 | 0.2201 |
| 11 | HBII-52-34;ENSG00000199311;SNORD115-34_chr15_25477533_25477615 | 0.2004 |
| 12 | HBII-52-12;ENSG00000199453;SNORD115-12_chr15_25436562_25436644 | 0.1952 |
| 13 | SNORD123;ENSG00000239112;SNORD123_chr5_9548947_9549017 | 0.1899 |
| 14 | HBII-52-17;ENSG00000201482;SNORD115-17_chr15_25446469_25446551 | 0.1858 |
| 15 | HBII-85-29;ENSG00000207245;SNORD116-29_chr15_25351666_25351751 | 0.1787 |
| 16 | HBII-52-33;ENSG00000200593;SNORD115-33_chr15_25475984_25476066 | 0.1714 |
| 17 | ENSG00000222345;SNORD19_chr3_52725394_52725469 | 0.1619 |
| 18 | HBII-52-38;ENSG00000201907;SNORD115-38_chr15_25484984_25485066 | 0.1617 |
| 19 | HBII-52-43;ENSG00000202373;SNORD115-43_chr15_25494344_25494426 | 0.1496 |
| 20 | HBII-52-9;ENSG00000199782;SNORD115-9_chr15_25430777_25430859 | 0.1488 |
| 21 | HBII-52-20;ENSG00000201969;SNORD115-20_chr15_25451408_25451490 | 0.1478 |
| 22 | HBII-52-18;ENSG00000200163;SNORD115-18_chr15_25448373_25448455 | 0.1469 |
| 23 | HBII-52-44;ENSG00000202261;SNORD115-44_chr15_25496005_25496087 | 0.1453 |
| 24 | HBII-85-27;ENSG00000251896;SNORD116-27_chr15_25346720_25346814 | 0.1443 |
| 25 | HBII-52-41;ENSG00000200478;SNORD115-41_chr15_25490624_25490706 | 0.1425 |
| 26 | HBII-420;ENSG00000221539;SNORD99_chr1_28905254_28905334 | 0.1399 |
| 27 | HBII-52-36;ENSG00000202499;SNORD115-36_chr15_25481231_25481313 | 0.1362 |
| 28 | HBII-52-21;ENSG00000199833;SNORD115-21_chr15_25453229_25453310 | 0.1336 |
| 29 | HBII-52-39;ENSG00000200564;SNORD115-39_chr15_25486892_25486974 | 0.1320 |
| 30 | HBII-52-8;ENSG00000200726;SNORD115-8_chr15_25429452_25429534 | 0.1303 |
| 31 | HBII-52-26_chr15_25463763_25463845 | 0.1288 |
| 32 | ENSG00000252529;RNU3P3_chr14_85738276_85738405 | 0.1285 |
| 33 | HBI-115;ENSG00000238961;SNORA47_chr5_76376258_76376396 | 0.1283 |
| 34 | U18C;ENSG00000199574;SNORD18C_chr15_66793588_66793656 | 0.1276 |
| 35 | ENSG00000252299;U3_chr9_90989184_90989274 | 0.1266 |
| 36 | HBII-52-11;ENSG00000200486;SNORD115-11_chr15_25434560_25434642 | 0.1262 |
| 37 | U79_chr1_173834485_173834570 | 0.1253 |
| 38 | ENSG00000199769;U3_chrX_70065931_70066145 | 0.1248 |
| 39 | mgU6-77;ENSG00000238917;SNORD10_chr17_7480128_7480276 | 0.1246 |
| 40 | HBI-100;ENSG00000252906;SCARNA3_chr1_175937532_175937676 | 0.1244 |
| 41 | ACA63;ENSG00000221643;SNORA77_chr1_203698708_203698833 | 0.1230 |
| 42 | U44_chr1_173835103_173835166 | 0.1227 |
| 43 | HBII-52-29;ENSG00000199704;SNORD115-29_chr15_25468392_25468474 | 0.1215 |
| 44 | U28_chr11_62622092_62622167 | 0.1215 |
| 45 | HBII-52-40;ENSG00000272460;SNORD115-40_chr15_25488760_25488842 | 0.1195 |
| 46 | ENSG00000200620;SNORA7_chrX_15734331_15734469 | 0.1187 |
| 47 | HBII-52-19;ENSG00000199968;SNORD115-19_chr15_25449503_25449585 | 0.1177 |
| 48 | ACA36B;ENSG00000222370;SNORA36B_chr1_220373887_220374018 | 0.1144 |
| 49 | U50_chr6_86387011_86387086 | 0.1129 |
| 50 | HBII-85-3;ENSG00000207014;SNORD116-3_chr15_25302005_25302102 | 0.1122 |
| 51 | HBII-52-13_chr15_25438467_25438549 | 0.1111 |
| 52 | HBII-85-9;ENSG00000206727;SNORD116-9_chr15_25318252_25318349 | 0.1093 |
| 53 | HBII-52-25;ENSG00000199489;SNORD115-25_chr15_25460687_25460769 | 0.1085 |
| 54 | HBII-85-20_chr15_25332807_25332901 | 0.1078 |
| 55 | HBII-85-21_chr15_25333949_25334043 | 0.1056 |
| 56 | HBII-85-22_chr15_25335068_25335162 | 0.1047 |
| 57 | ACA56;ENSG00000206693;SNORA56_chrX_154003272_154003401 | 0.1026 |
| 58 | ACA31_chr13_45911614_45911744 | 0.1021 |
| 59 | HBII-85-17;ENSG00000206656;SNORD116-17_chr15_25328733_25328827 | 0.1021 |
| 60 | U69;ENSG00000206622;SNORA69_chrX_118921315_118921447 | 0.1015 |
| 61 | U61;ENSG00000206979;SNORD61_chrX_135961357_135961430 | 0.1004 |
| 62 | HBII-52-22;ENSG00000201326;SNORD115-22_chr15_25455064_25455146 | 0.1003 |
| 63 | HBII-85-19;ENSG00000207460;SNORD116-19_chr15_25331672_25331766 | 0.0999 |
| 64 | HBII-85-8;ENSG00000207093;SNORD116-8_chr15_25315577_25315674 | 0.0998 |
| 65 | HBII-85-16;ENSG00000207263;SNORD116-16_chr15_25327913_25328007 | 0.0993 |
| 66 | U19;ENSG00000200959;SNORA74A_chr5_138614468_138614668 | 0.0983 |
| 67 | HBII-296A;ENSG00000212163;SNORD91A_chr17_2233474_2233664 | 0.0981 |
| 68 | HBII-52-3;ENSG00000199970;SNORD115-3_chr15_25420073_25420155 | 0.0967 |
| 69 | HBII-85-24;ENSG00000207279;SNORD116-24_chr15_25339182_25339276 | 0.0965 |
| 70 | ENSG00000212182;U3_chr2_114763018_114763232 | 0.0957 |
| 71 | U13;ENSG00000239039;SNORD13_chr8_33370991_33371096 | 0.0947 |
| 72 | U49A_chr17_16343349_16343420 | 0.0939 |
| 73 | HBII-180A;ENSG00000221241;SNORD88A_chr19_51302695_51302792 | 0.0912 |
| 74 | U60;ENSG00000206630;SNORD60_chr16_2205023_2205106 | 0.0911 |
| 75 | ENSG00000221461;U3_chr8_124192551_124192765 | 0.0911 |
| 76 | HBII-85-18;ENSG00000206688;SNORD116-18_chr15_25330530_25330624 | 0.0906 |
| 77 | U46;ENSG00000200913;SNORD46_chr1_45242162_45242265 | 0.0900 |
| 78 | snR38C_chr17_74554871_74554951 | 0.0898 |
| 79 | ACA26;ENSG00000252808;SCARNA4_chr1_155895748_155895877 | 0.0898 |
| 80 | HBII-85-6;ENSG00000207442;SNORD116-6_chr15_25310171_25310269 | 0.0885 |
| 81 | HBII-85-1;ENSG00000207063;SNORD116-1_chr15_25296622_25296719 | 0.0884 |
| 82 | U63;ENSG00000206989;SNORD63_chr5_137896731_137896799 | 0.0878 |
| 83 | HBII-85-14;ENSG00000206621;SNORD116-14_chr15_25325287_25325381 | 0.0878 |
| 84 | SNORA36C;ENSG00000207016;SNORA36C_chr2_69747174_69747306 | 0.0873 |
| 85 | U58B;ENSG00000271982;SNORD58B_chr18_47018033_47018099 | 0.0870 |
| 86 | HBII-85-25;ENSG00000252326;SNORD116-25_chr15_25342808_25342902 | 0.0862 |
| 87 | ACA3;ENSG00000200983;SNORA3_chr11_8705773_8705903 | 0.0858 |
| 88 | U105B;ENSG00000238531;SNORD105B_chr19_10220432_10220511 | 0.0851 |
| 89 | ENSG00000206903;SNORA24_chr15_65577799_65577929 | 0.0851 |
| 90 | U23;ENSG00000206885;SNORA75_chr2_232320510_232320647 | 0.0850 |
| 91 | U78;ENSG00000208317;SNORD78_chr1_173834759_173834824 | 0.0832 |
| 92 | U37;ENSG00000206775;SNORD37_chr19_3982504_3982570 | 0.0830 |
| 93 | HBII-85-26;ENSG00000251815;SNORD116-26_chr15_25344644_25344742 | 0.0829 |
| 94 | U29_chr11_62621375_62621440 | 0.0826 |
| 95 | ACA58_chr3_131197940_131198077 | 0.0825 |
| 96 | ENSG00000200206;SNORD74_chr15_86716429_86716501 | 0.0824 |
| 97 | HBII-85-2;ENSG00000207001;SNORD116-2_chr15_25299355_25299452 | 0.0820 |
| 98 | HBII-289;ENSG00000212283;SNORD89_chr2_101889397_101889511 | 0.0814 |
| 99 | HBII-85-23;ENSG00000207375;SNORD116-23_chr15_25336931_25337025 | 0.0810 |
| 100 | ENSG00000238503;SNORD18_chr2_12170429_12170498 | 0.0807 |
| 101 | U33;ENSG00000199631;SNORD33_chr19_49993872_49993956 | 0.0805 |
| 102 | U76_chr1_173835772_173835852 | 0.0797 |
| 103 | U21;ENSG00000206680;SNORD21_chr1_93302845_93302940 | 0.0784 |
| 104 | HBII-85-15;ENSG00000207174;SNORD116-15_chr15_25326432_25326526 | 0.0780 |
| 105 | U73a;ENSG00000208797;SNORD73A_chr4_152024978_152025043 | 0.0772 |
| 106 | HBII-52-1;ENSG00000201831;SNORD115-1_chr15_25415869_25415951 | 0.0770 |
| 107 | U17b;ENSG00000200087;SNORA73B_chr1_28835069_28835274 | 0.0770 |
| 108 | U95;ENSG00000264549;SNORD95_chr5_180670312_180670379 | 0.0769 |
| 109 | ENSG00000252787;SNORD19B_chr3_52722898_52722977 | 0.0742 |
| 110 | U49B_chr17_16342822_16342870 | 0.0739 |
| 111 | ACA35;ENSG00000252947;SCARNA1_chr1_28160911_28161077 | 0.0736 |
| 112 | ACA57;ENSG00000251898;SCARNA11_chr12_6690638_6690775 | 0.0734 |
| 113 | HBII-52-16;ENSG00000200757;SNORD115-16_chr15_25444594_25444676 | 0.0731 |
| 114 | HBII-180C;ENSG00000220988;SNORD88C_chr19_51305581_51305678 | 0.0721 |
| 115 | U77_chr1_173835438_173835508 | 0.0718 |
| 116 | HBII-52-30;ENSG00000200987;SNORD115-30_chr15_25470349_25470431 | 0.0700 |
| 117 | ACA36;ENSG00000206948;SNORA36A_chrX_153996802_153996934 | 0.0700 |
| 118 | ENSG00000221719;SNORA3_chr16_2846409_2846533 | 0.0692 |
| 119 | HBII-296B;ENSG00000275084;SNORD91B_chr17_2232310_2232531 | 0.0678 |
| 120 | U38A;ENSG00000202031;SNORD38A_chr1_45243513_45243584 | 0.0677 |
| 121 | U83;ENSG00000201785;SNORD117_chr6_31504150_31504226 | 0.0676 |
| 122 | 14q(II-3);ENSG00000201839;SNORD114-3_chr14_101419685_101419760 | 0.0655 |
| 123 | HBII-108B;ENSG00000238862;SNORD19B_chr3_52724759_52724843 | 0.0653 |
| 124 | HBII-82B;ENSG00000221514;SNORD111B_chr16_70563411_70563498 | 0.0647 |
| 125 | U82;ENSG00000202400;SNORD82_chr2_232325078_232325153 | 0.0645 |
| 126 | U24;ENSG00000206611;SNORD24_chr9_136216250_136216325 | 0.0642 |
| 127 | ACA43_chr9_139620555_139620691 | 0.0640 |
| 128 | U50B_chr6_86387306_86387377 | 0.0634 |
| 129 | U45A;ENSG00000207241;SNORD45A_chr1_76253573_76253657 | 0.0630 |
| 130 | U71b;ENSG00000235408;SNORA71B_chr20_37053731_37054002 | 0.0629 |
| 131 | 14q(II-26);ENSG00000200413;SNORD114-26_chr14_101453382_101453454 | 0.0629 |
| 132 | ENSG00000264346;SNORA77_chr22_20113925_20114049 | 0.0626 |
| 133 | ENSG00000212168;SNORD78_chr2_57771670_57771734 | 0.0625 |
| 134 | 14q(II-28);ENSG00000200480;SNORD114-28_chr14_101455466_101455538 | 0.0623 |
| 135 | 14q(I-8);ENSG00000200367;SNORD113-8_chr14_101409787_101409861 | 0.0614 |
| 136 | U45C;ENSG00000206620;SNORD45C_chr1_76252756_76252835 | 0.0612 |
| 137 | mgU6-47;ENSG00000207297;SNORD7_chr17_33900675_33900772 | 0.0610 |
| 138 | U83A;ENSG00000209482;SNORD83A_chr22_39711217_39711312 | 0.0610 |
| 139 | U83B;ENSG00000209480;SNORD83B_chr22_39709823_39709916 | 0.0610 |
| 140 | ENSG00000212309;SNORD70_chr2_203142831_203142915 | 0.0607 |
| 141 | 14q(II-12);ENSG00000202270;SNORD114-12_chr14_101435284_101435359 | 0.0606 |
| 142 | ENSG00000238707;SNORD2_chr10_58355723_58355791 | 0.0605 |
| 143 | ENSG00000265706;SNORD53_SNORD92_chr2_29150849_29150926 | 0.0605 |
| 144 | U18B;ENSG00000202529;SNORD18B_chr15_66794358_66794429 | 0.0599 |
| 145 | U3-3;ENSG00000264940;SNORD3C_chr17_19092978_19093558 | 0.0595 |
| 146 | 14q(II-23);ENSG00000200406;SNORD114-23_chr14_101450212_101450284 | 0.0590 |
| 147 | HBII-82;ENSG00000221066;SNORD111_chr16_70571907_70572001 | 0.0590 |
| 148 | 14q(II-25);ENSG00000200612;SNORD114-25_chr14_101452393_101452465 | 0.0585 |
| 149 | ACA60;ENSG00000199266;SNORA60_chr20_37078011_37078147 | 0.0583 |
| 150 | U3-2;ENSG00000265185;SNORD3B-1_chr17_18965224_18965982 | 0.0578 |
| 151 | HBII-52-2;ENSG00000199712;SNORD115-2_chr15_25417781_25417863 | 0.0575 |
| 152 | Z17B;ENSG00000238597;SNORD4B_chr17_27050698_27050772 | 0.0569 |
| 153 | U3-4_chr17_19015732_19015949 | 0.0567 |
| 154 | HBII-234;ENSG00000212534;SNORD70_chr2_203141153_203141241 | 0.0565 |
| 155 | 14q(II-17);ENSG00000201569;SNORD114-17_chr14_101441142_101441217 | 0.0564 |
| 156 | U32A;ENSG00000201675;SNORD32A_chr19_49993222_49993305 | 0.0563 |
| 157 | ENSG00000199363;SNORA63_chr3_183171602_183171732 | 0.0563 |
| 158 | U3-2B;ENSG00000262074;SNORD3B-2_chr17_18966659_18967449 | 0.0561 |
| 159 | 14q(II-21);ENSG00000272344;SNORD114-21_chr14_101448311_101448383 | 0.0561 |
| 160 | U3;ENSG00000263934;SNORD3A_chr17_19091328_19092027 | 0.0559 |
| 161 | U75_chr1_173836016_173836076 | 0.0554 |
| 162 | ACA7B;ENSG00000207088;SNORA7B_chr3_129116052_129116191 | 0.0554 |
| 163 | U59A;ENSG00000207031;SNORD59A_chr12_57038810_57038885 | 0.0551 |
| 164 | ACA62_chr17_62223698_62223831 | 0.0551 |
| 165 | SNORD126;ENSG00000238344;SNORD126_chr14_20794608_20794685 | 0.0550 |
| 166 | ENSG00000207215;U3_chr8_98370493_98370702 | 0.0547 |
| 167 | HBII-180B;ENSG00000221381;SNORD88B_chr19_51302285_51302382 | 0.0538 |
| 168 | HBII-295;ENSG00000212447;SNORD90_chr9_125642491_125642602 | 0.0529 |
| 169 | 14q(II-9);ENSG00000201240;SNORD114-9_chr14_101432365_101432437 | 0.0527 |
| 170 | HBII-52-23;ENSG00000201331;SNORD115-23_chr15_25456942_25457024 | 0.0526 |
| 171 | U103;ENSG00000200154;SNORD103A_chr1_31408532_31408623 | 0.0526 |
| 172 | HBII-13_chr15_25230246_25230313 | 0.0525 |
| 173 | 14q(I-5);ENSG00000272474;SNORD113-5_chr14_101404523_101404601 | 0.0525 |
| 174 | U34;ENSG00000202503;SNORD34_chr19_49994161_49994231 | 0.0520 |
| 175 | 14q(II-22);ENSG00000202293;SNORD114-22_chr14_101449262_101449334 | 0.0519 |
| 176 | U103B;ENSG00000202107;SNORD103B_chr1_31421961_31422052 | 0.0516 |
| 177 | ENSG00000200398;SNORD115-24_chr15_25458795_25458876 | 0.0507 |
| 178 | U17a_chr1_28833876_28834083 | 0.0503 |
| 179 | ENSG00000252792;U3_chr14_68212084_68212191 | 0.0502 |
| 180 | HBII-85-7;ENSG00000207133;SNORD116-7_chr15_25312933_25313030 | 0.0502 |
| 181 | mgh28S-2409;ENSG00000239195;SNORD5_chr11_93466393_93466466 | 0.0501 |
| 182 | HBII-436_chr15_25227140_25227215 | 0.0501 |
| 183 | ACA45_chr15_83424696_83424823 | 0.0499 |
| 184 | U74_chr1_173836811_173836883 | 0.0486 |
| 185 | U87;ENSG00000252010;SCARNA5_chr2_234184371_234184649 | 0.0486 |
| 186 | snR38A_chr17_74557714_74557786 | 0.0485 |
| 187 | ENSG00000221611;SNORD88_chr12_13124987_13125077 | 0.0484 |
| 188 | HBII-85-5;ENSG00000207191;SNORD116-5_chr15_25307478_25307575 | 0.0482 |
| 189 | 14q(II-14);ENSG00000199593;SNORD114-14_chr14_101438439_101438514 | 0.0481 |
| 190 | ENSG00000221750;SNORA11_chrX_54953738_54953866 | 0.0480 |
| 191 | ENSG00000212206;SNORA69_chr17_8232901_8233037 | 0.0478 |
| 192 | ENSG00000201009;SNORD46_chr7_132437783_132437886 | 0.0475 |
| 193 | 14q(II-5);ENSG00000199798;SNORD114-5_chr14_101421706_101421776 | 0.0474 |
| 194 | 14q(II-1);ENSG00000199575;SNORD114-1_chr14_101416169_101416241 | 0.0472 |
| 195 | 14q(I-7);ENSG00000200632;SNORD113-7_chr14_101407462_101407539 | 0.0471 |
| 196 | ENSG00000221376;SNORA77_chr15_69617632_69617759 | 0.0470 |
| 197 | ENSG00000201157;SNORA62_chr8_18837129_18837282 | 0.0468 |
| 198 | SNORD119;ENSG00000251806;SNORD119_chr20_2443604_2443686 | 0.0466 |
| 199 | ACA19;ENSG00000207468;SNORA19_chr10_120819522_120819650 | 0.0466 |
| 200 | ENSG00000207130;SNORA24_chr3_128433414_128433548 | 0.0460 |
| 201 | U80_chr1_173833966_173834044 | 0.0459 |
| 202 | ENSG00000238563;snoU13_chr6_114162001_114162089 | 0.0459 |
| 203 | U36C;ENSG00000252542;SNORD36C_chr9_136217700_136217768 | 0.0457 |
| 204 | mgU6-53B;ENSG00000199436;SNORD9_chr14_21860309_21860412 | 0.0452 |
| 205 | ENSG00000252193;SCARNA15_chr20_41933195_41933319 | 0.0450 |
| 206 | U42A;ENSG00000238649;SNORD42A_chr17_27050447_27050510 | 0.0449 |
| 207 | U104;ENSG00000199753;SNORD104_chr17_62223437_62223517 | 0.0446 |
| 208 | SNORA84;ENSG00000239183;SNORA84_chr9_95054742_95054875 | 0.0444 |
| 209 | ENSG00000252128;SNORD27_chr13_21714025_21714096 | 0.0443 |
| 210 | ACA53;ENSG00000212443;SNORA53_chr12_98993412_98993662 | 0.0443 |
| 211 | 14q(I-3);ENSG00000201700;SNORD113-3_chr14_101396255_101396328 | 0.0439 |
| 212 | ENSG00000207407;SNORA1_chr11_19612703_19612838 | 0.0438 |
| 213 | ACA1;ENSG00000206834;SNORA1_chr11_93465169_93465299 | 0.0434 |
| 214 | 14q(I-9);ENSG00000201950;SNORD113-9_chr14_101411985_101412057 | 0.0430 |
| 215 | U102;ENSG00000207500;SNORD102_chr13_27829200_27829272 | 0.0428 |
| 216 | HBII-239;ENSG00000223224;SNORD71_chr16_71792304_71792390 | 0.0427 |
| 217 | U81_chr1_173833283_173833360 | 0.0425 |
| 218 | 14q(I-6);ENSG00000200215;SNORD113-6_chr14_101405892_101405968 | 0.0424 |
| 219 | ENSG00000200652;SNORA25_chr16_28190419_28190544 | 0.0422 |
| 220 | U41;ENSG00000209702;SNORD41_chr19_12817262_12817332 | 0.0419 |
| 221 | HBII-52-31;ENSG00000202188;SNORD115-31_chr15_25472255_25472337 | 0.0418 |
| 222 | snR39B;ENSG00000238942;SNORD2_chr3_186502584_186502654 | 0.0415 |
| 223 | U97;ENSG00000238622;SNORD97_chr11_10823013_10823155 | 0.0415 |
| 224 | HBII-142;ENSG00000212158;SNORD66_chr3_184043483_184043559 | 0.0414 |
| 225 | U42B;ENSG00000238423;SNORD42B_chr17_27047567_27047634 | 0.0414 |
| 226 | HBII-52-48;ENSG00000201634;SNORD115-48_chr15_25514929_25515005 | 0.0414 |
| 227 | U58A;ENSG00000206602;SNORD58A_chr18_47017652_47017717 | 0.0412 |
| 228 | HBII-210;ENSG00000212452;SNORD69_chr3_52726751_52726828 | 0.0403 |
| 229 | U93;ENSG00000252481;SCARNA13_chr14_95999691_95999966 | 0.0401 |
| 230 | ENSG00000212532;SNORD66_chr6_51329488_51329563 | 0.0400 |
| 231 | mgU2-19/30_chr11_93454679_93455032 | 0.0399 |
| 232 | ENSG00000252213;SNORA74_chr5_138611869_138612009 | 0.0399 |
| 233 | ENSG00000199405;SNORA1_chr8_56815282_56815414 | 0.0397 |
| 234 | U71a;ENSG00000225091;SNORA71A_chr20_37055948_37056086 | 0.0396 |
| 235 | U62B_chr9_134365872_134365958 | 0.0392 |
| 236 | ACA3-2;ENSG00000212607;SNORA45_chr11_8706985_8707116 | 0.0392 |
| 237 | U18A;ENSG00000200623;SNORD18A_chr15_66795581_66795652 | 0.0392 |
| 238 | U94;ENSG00000208772;SNORD94_chr2_86362992_86363129 | 0.0390 |
| 239 | HBII-276_chr8_67834708_67834784 | 0.0390 |
| 240 | HBII-85-4_chr15_25304683_25304781 | 0.0390 |
| 241 | SNORD125;ENSG00000239127;SNORD125_chr22_29729151_29729247 | 0.0390 |
| 242 | U30_chr11_62621134_62621204 | 0.0386 |
| 243 | U59B_chr12_57037463_57037538 | 0.0384 |
| 244 | U57_chr20_2637584_2637656 | 0.0380 |
| 245 | U15A;ENSG00000206941;SNORD15A_chr11_75111434_75111582 | 0.0380 |
| 246 | ACA55;ENSG00000201457;SNORA55_chr1_40033045_40033182 | 0.0376 |
| 247 | 14q(II-16);ENSG00000199914;SNORD114-16_chr14_101439931_101440001 | 0.0375 |
| 248 | 14q(II-15);ENSG00000201557;SNORD114-15_chr14_101439006_101439078 | 0.0372 |
| 249 | U58C;ENSG00000202093;SNORD58C_chr18_47015613_47015678 | 0.0372 |
| 250 | U62A_chr9_134361051_134361137 | 0.0372 |
| 251 | ENSG00000212611;SNORD30_chrX_113260261_113260330 | 0.0372 |
| 252 | U105;ENSG00000209645;SNORD105_chr19_10218326_10218411 | 0.0368 |
| 253 | ACA24_chr4_119200344_119200475 | 0.0367 |
| 254 | ENSG00000238618;snoU13_chr1_51696925_51697028 | 0.0367 |
| 255 | U35A;ENSG00000200259;SNORD35A_chr19_49994431_49994517 | 0.0366 |
| 256 | hTR_chr3_169482397_169482945 | 0.0366 |
| 257 | HBII-429;ENSG00000221500;SNORD100_chr6_133137940_133138016 | 0.0365 |
| 258 | mgh18S-121;ENSG00000238578;SNORD4A_chr17_27049599_27049671 | 0.0364 |
| 259 | ENSG00000202252;SNORD14C_chr11_122930043_122930130 | 0.0363 |
| 260 | U86;ENSG00000212498;SNORD86_chr20_2636742_2636828 | 0.0362 |
| 261 | U43;ENSG00000263764;SNORD43_chr22_39715055_39715118 | 0.0362 |
| 262 | U101;ENSG00000206754;SNORD101_chr6_133136445_133136518 | 0.0357 |
| 263 | HBII-251;ENSG00000200181;SNORD85_chr1_31441009_31441084 | 0.0357 |
| 264 | U100;ENSG00000252712;SCARNA14_chr15_66639543_66639680 | 0.0355 |
| 265 | U71d;ENSG00000200354;SNORA71D_chr20_37062504_37062642 | 0.0353 |
| 266 | ACA2b;ENSG00000207313;SNORA2B_chr12_49061239_49061376 | 0.0351 |
| 267 | ENSG00000239027;snoU13_chr1_19858664_19858768 | 0.0351 |
| 268 | HBII-438B;ENSG00000239169;SNORD109B_chr15_25523489_25523556 | 0.0350 |
| 269 | E3;ENSG00000200320;SNORA63_chr3_186505087_186505222 | 0.0350 |
| 270 | ACA7;ENSG00000207496;SNORA7A_chr3_12881810_12881949 | 0.0350 |
| 271 | ACA11_chr4_1976362_1976487 | 0.0349 |
| 272 | ENSG00000271817;U3_chr4_159621843_159622060 | 0.0348 |
| 273 | ENSG00000252277;SNORD116-30_chr15_25353415_25353499 | 0.0348 |
| 274 | U22_chr11_62620381_62620507 | 0.0345 |
| 275 | U91;ENSG00000252139;SCARNA18_chr18_47340730_47340813 | 0.0345 |
| 276 | ENSG00000252050;SNORA31_chrX_38167365_38167494 | 0.0342 |
| 277 | HBII-52-35;ENSG00000201992;SNORD115-35_chr15_25479393_25479475 | 0.0342 |
| 278 | U65;ENSG00000201302;SNORA65_chr9_130210779_130210916 | 0.0339 |
| 279 | 14q(II-27);ENSG00000200636;SNORD114-27_chr14_101454497_101454567 | 0.0339 |
| 280 | U106;ENSG00000209042;SNORD12C_chr20_47895477_47895565 | 0.0338 |
| 281 | ENSG00000206976;SNORA7_chr11_3943797_3943933 | 0.0338 |
| 282 | ENSG00000202268;U3_chr3_45296902_45297112 | 0.0336 |
| 283 | ENSG00000212195;U3_chr17_56709003_56709197 | 0.0336 |
| 284 | SNORD124;ENSG00000238793;SNORD124_chr17_38183794_38183898 | 0.0335 |
| 285 | ACA32;ENSG00000206799;SNORA32_chr11_93464144_93464265 | 0.0334 |
| 286 | 14q(0)_chr14_101364256_101364333 | 0.0332 |
| 287 | U88;ENSG00000251791;SCARNA6_chr2_234197321_234197587 | 0.0332 |
| 288 | U67_chr17_7481272_7481409 | 0.0331 |
| 289 | ACA50;ENSG00000206952;SNORA50_chr16_58593699_58593835 | 0.0326 |
| 290 | U92;ENSG00000251733;SCARNA8_chr9_19063653_19063784 | 0.0326 |
| 291 | U15B;ENSG00000207445;SNORD15B_chr11_75115464_75115610 | 0.0325 |
| 292 | mgU12-22/U4-8;ENSG00000252139;SCARNA18_chr18_47340392_47340813 | 0.0325 |
| 293 | 14q(II-20);ENSG00000202048;SNORD114-20_chr14_101447340_101447412 | 0.0325 |
| 294 | ENSG00000206977;SNORA8_chr6_41800592_41800731 | 0.0323 |
| 295 | U71c;ENSG00000201512;SNORA71C_chr20_37058309_37058447 | 0.0323 |
| 296 | ENSG00000199787;SNORA42_chr16_30430946_30431080 | 0.0322 |
| 297 | HBII-438A_chr15_25287120_25287187 | 0.0322 |
| 298 | HBI-61;ENSG00000221420;SNORA81_chr3_186504463_186504641 | 0.0322 |
| 299 | ACA61_chr1_28906275_28906405 | 0.0321 |
| 300 | ACA4;ENSG00000263776;SNORA4_chr3_186505401_186505538 | 0.0320 |
| 301 | U20;ENSG00000207280;SNORD20_chr2_232321154_232321234 | 0.0319 |
| 302 | U31_chr11_62620796_62620867 | 0.0318 |
| 303 | U64;ENSG00000207405;SNORA64_chr16_2012973_2013107 | 0.0316 |
| 304 | ACA21;ENSG00000199293;SNORA21_chr17_37009115_37009248 | 0.0314 |
| 305 | U90;ENSG00000238741;SCARNA7_chr3_160232694_160233024 | 0.0311 |
| 306 | 14q(II-13);ENSG00000201247;SNORD114-13_chr14_101436215_101436289 | 0.0310 |
| 307 | U96a;ENSG00000272296;SNORD96A_chr5_180668814_180668892 | 0.0309 |
| 308 | mgU12-22/U4-8;ENSG00000251992;SCARNA17_chr18_47340392_47340813 | 0.0309 |
| 309 | ENSG00000252433;SNORA31_chr1_67568328_67568462 | 0.0308 |
| 310 | ACA20;ENSG00000207392;SNORA20_chr6_160201281_160201413 | 0.0308 |
| 311 | U53;ENSG00000265145;SNORD53_chr2_29149932_29150010 | 0.0308 |
| 312 | ENSG00000238327;snoU13_chrX_23525328_23525430 | 0.0306 |
| 313 | ENSG00000238325;snoU13_chr1_167010672_167010773 | 0.0305 |
| 314 | U54;ENSG00000238650;SNORD54_chr8_56986394_56986460 | 0.0302 |
| 315 | 14q(II-4);ENSG00000200832;SNORD114-4_chr14_101420710_101420785 | 0.0301 |
| 316 | ENSG00000202335;SNORD50_chr12_110934157_110934226 | 0.0301 |
| 317 | 14q(II-24);ENSG00000201899;SNORD114-24_chr14_101451113_101451185 | 0.0300 |
| 318 | U48;ENSG00000201823;SNORD48_chr6_31803039_31803103 | 0.0298 |
| 319 | U38B;ENSG00000207421;SNORD38B_chr1_45244061_45244130 | 0.0295 |
| 320 | ENSG00000253051;SNORA31_chr13_45910449_45910582 | 0.0295 |
| 321 | HBII-202;ENSG00000200084;SNORD68_chr16_89627837_89627925 | 0.0294 |
| 322 | 14q(I-4);ENSG00000201672;SNORD113-4_chr14_101402827_101402902 | 0.0293 |
| 323 | ACA33;ENSG00000200534;SNORA33_chr6_133138357_133138490 | 0.0291 |
| 324 | ENSG00000238790;snoU13_chr18_3811317_3811420 | 0.0289 |
| 325 | ENSG00000238484;snoU13_chr6_34660557_34660660 | 0.0288 |
| 326 | 14q(II-6);ENSG00000201263;SNORD114-6_chr14_101423502_101423574 | 0.0288 |
| 327 | 14q(II-10);ENSG00000200279;SNORD114-10_chr14_101433388_101433460 | 0.0284 |
| 328 | ENSG00000238410;snoU109_chr2_75716702_75716836 | 0.0283 |
| 329 | ACA16_chr1_28907431_28907565 | 0.0280 |
| 330 | U47_chr1_173833507_173833572 | 0.0280 |
| 331 | 14q(II-11);ENSG00000200608;SNORD114-11_chr14_101434447_101434522 | 0.0280 |
| 332 | ENSG00000200072;SNORD44_chr13_112706392_112706452 | 0.0280 |
| 333 | HBI-6;ENSG00000212588;SNORA26_chr4_53579415_53579537 | 0.0279 |
| 334 | HBII-316;ENSG00000264994;SNORD92_chr2_29136527_29136616 | 0.0279 |
| 335 | ENSG00000201882;snoU2-30_chrX_20154184_20154253 | 0.0279 |
| 336 | ENSG00000238754;snoU109_chr1_193026411_193026545 | 0.0278 |
| 337 | HBII-85-10;ENSG00000200661;SNORD116-10_chr15_25319259_25319363 | 0.0276 |
| 338 | E2;ENSG00000202363;SNORA62_chr3_39452544_39452698 | 0.0275 |
| 339 | U26_chr11_62622763_62622838 | 0.0273 |
| 340 | ACA41;ENSG00000207406;SNORA41_chr2_207026951_207027083 | 0.0272 |
| 341 | HBII-240;ENSG00000212296;SNORD72_chr5_40832757_40832837 | 0.0271 |
| 342 | mgh28S-2411;ENSG00000202314;SNORD6_chr11_93464668_93464740 | 0.0271 |
| 343 | ENSG00000252582;SNORA31_chr9_133896555_133896693 | 0.0271 |
| 344 | U25_chr11_62623036_62623103 | 0.0270 |
| 345 | ENSG00000238501;snoU13_chr14_45036116_45036209 | 0.0270 |
| 346 | ENSG00000201229;SNORA63_chr3_183169645_183169776 | 0.0267 |
| 347 | U56_chr20_2637269_2637340 | 0.0267 |
| 348 | ENSG00000252699;SNORA21_chr17_37007777_37007912 | 0.0265 |
| 349 | ENSG00000202269;U8_chr5_15110895_15111029 | 0.0265 |
| 350 | ACA44_chr1_28906892_28907024 | 0.0264 |
| 351 | ENSG00000201541;SNORA1_chr16_24344736_24344868 | 0.0263 |
| 352 | ACA9_chr7_45024976_45025109 | 0.0263 |
| 353 | ACA46;ENSG00000207493;SNORA46_chr16_58582402_58582537 | 0.0261 |
| 354 | ENSG00000212378;SNORD78_chr2_72987663_72987731 | 0.0261 |
| 355 | ENSG00000252829;SCARNA4_chr2_131687335_131687457 | 0.0258 |
| 356 | 14q(II-29);ENSG00000201689;SNORD114-29_chr14_101456427_101456497 | 0.0258 |
| 357 | HBII-85-11;ENSG00000206609;SNORD116-11_chr15_25321074_25321168 | 0.0257 |
| 358 | U51;ENSG00000207047;SNORD51_chr2_207026602_207026681 | 0.0257 |
| 359 | ENSG00000201448;SNORA63_chr1_36884051_36884179 | 0.0257 |
| 360 | HBII-95;ENSG00000238317;SNORD11_chr2_203157772_203157859 | 0.0257 |
| 361 | ENSG00000201619;SNORA67_chr1_179170622_179170762 | 0.0256 |
| 362 | U8;ENSG00000200463;SNORD118_chr17_8076770_8076906 | 0.0256 |
| 363 | 14q(II-30);ENSG00000201318;SNORD114-30_chr14_101458255_101458327 | 0.0256 |
| 364 | ACA59B;ENSG00000266079;SNORA59B_chr17_19460524_19461224 | 0.0255 |
| 365 | ENSG00000221148;SNORA3_chr12_84577103_84577227 | 0.0254 |
| 366 | U99;ENSG00000206597;SNORA57_chr11_62432893_62433042 | 0.0254 |
| 367 | U84;ENSG00000265236;SNORD84_chr6_31508877_31508955 | 0.0251 |
| 368 | U55;ENSG00000264294;SNORD55_chr1_45241536_45241615 | 0.0251 |
| 369 | ENSG00000212553;SNORD116_chr13_40431272_40431364 | 0.0251 |
| 370 | SNORA11E;ENSG00000221705;SNORA11E_chrX_51806442_51806570 | 0.0250 |
| 371 | 14q(II-19);ENSG00000199942;SNORD114-19_chr14_101442813_101442888 | 0.0248 |
| 372 | ENSG00000238772;snoU13_chr7_37209283_37209384 | 0.0244 |
| 373 | U70B;ENSG00000206937;SNORA70B_chr2_61644378_61644513 | 0.0242 |
| 374 | U85_chr12_6619387_6619717 | 0.0242 |
| 375 | ENSG00000223213;SNORD81_chr12_54185089_54185131 | 0.0240 |
| 376 | ENSG00000207094;SNORA67_chr7_88078407_88078546 | 0.0239 |
| 377 | ENSG00000207217;SNORA42_chr7_6056508_6056642 | 0.0238 |
| 378 | ENSG00000212598;U3_chr3_90079434_90079644 | 0.0238 |
| 379 | ENSG00000199321;SNORD60_chr10_128468271_128468343 | 0.0238 |
| 380 | ENSG00000252305;SNORA74_chr17_14080448_14080627 | 0.0238 |
| 381 | ENSG00000238536;snoU13_chr4_17530560_17530663 | 0.0237 |
| 382 | ENSG00000238326;snoU13_chr5_55296346_55296449 | 0.0236 |
| 383 | U16;ENSG00000199673;SNORD16_chr15_66795148_66795249 | 0.0236 |
| 384 | ENSG00000238860;snoU13_chr2_148081539_148081643 | 0.0235 |
| 385 | HBII-99B;ENSG00000222365;SNORD12B_chr20_47896855_47896946 | 0.0235 |
| 386 | ENSG00000222185;SNORD113_chr14_101464804_101464878 | 0.0235 |
| 387 | ENSG00000200237;SNORA70_chr19_9930630_9930770 | 0.0234 |
| 388 | ACA5c;ENSG00000201772;SNORA5C_chr7_45144504_45144641 | 0.0234 |
| 389 | ENSG00000207274;SNORA70_chr2_215711671_215711805 | 0.0234 |
| 390 | ENSG00000200418;SNORA63_chr3_186504112_186504234 | 0.0234 |
| 391 | U35B;ENSG00000200530;SNORD35B_chr19_50000975_50001063 | 0.0234 |
| 392 | U36A;ENSG00000199744;SNORD36A_chr9_136217310_136217383 | 0.0233 |
| 393 | ACA13;ENSG00000238363;SNORA13_chr5_111497181_111497314 | 0.0232 |
| 394 | U68;ENSG00000207166;SNORA68_chr19_17973396_17973529 | 0.0232 |
| 395 | ENSG00000202498;SNORD116_chr1_215803368_215803459 | 0.0231 |
| 396 | ENSG00000200969;SNORD95_chr9_84503833_84503900 | 0.0229 |
| 397 | ENSG00000199727;U3_chr2_105149231_105149442 | 0.0229 |
| 398 | ENSG00000221093;SNORA3_chr8_57033801_57033911 | 0.0229 |
| 399 | ACA6;ENSG00000206760;SNORA6_chr3_39449880_39450030 | 0.0229 |
| 400 | ENSG00000251930;U3_chr6_53012606_53012727 | 0.0229 |
| 401 | ACA18;ENSG00000207145;SNORA18_chr11_93466631_93466763 | 0.0228 |
| 402 | U52;ENSG00000201754;SNORD52_chr6_31804852_31804919 | 0.0228 |
| 403 | ENSG00000207098;SNORA70_chr21_34214171_34214305 | 0.0228 |
| 404 | ACA47_chr17_75085388_75085575 | 0.0227 |
| 405 | ACA54;ENSG00000207008;SNORA54_chr11_2985000_2985123 | 0.0227 |
| 406 | ENSG00000199474;SNORA27_chr16_12936344_12936467 | 0.0227 |
| 407 | ENSG00000238520;snoU13_chr2_115038661_115038752 | 0.0226 |
| 408 | ACA67;ENSG00000200792;SNORA80_chr21_33749495_33749631 | 0.0226 |
| 409 | ENSG00000199713;U8_chr18_56486115_56486248 | 0.0225 |
| 410 | ENSG00000200801;SNORD115-28_chr15_25467495_25467575 | 0.0225 |
| 411 | ENSG00000199977;SNORA73_chr18_19397585_19397789 | 0.0225 |
| 412 | ACA28;ENSG00000272533;SNORA28_chr14_103804185_103804311 | 0.0224 |
| 413 | SNORA11D;ENSG00000221475;SNORA11D_chrX_51933716_51933844 | 0.0222 |
| 414 | mgU2-25/61_chr1_109642814_109643234 | 0.0222 |
| 415 | ENSG00000202389;SNORA70_chr17_26349356_26349490 | 0.0221 |
| 416 | SNORD121B;ENSG00000238300;SNORD121B_chr9_33934294_33934374 | 0.0221 |
| 417 | SNORD127;ENSG00000239043;SNORD127_chr14_45580085_45580171 | 0.0221 |
| 418 | HBII-85-12;ENSG00000207197;SNORD116-12_chr15_25322196_25322290 | 0.0221 |
| 419 | HBII-108;ENSG00000212493;SNORD19_chr3_52723255_52723331 | 0.0220 |
| 420 | U27_chr11_62622483_62622555 | 0.0219 |
| 421 | HBII-55;ENSG00000221116;SNORD110_chr20_2634857_2634932 | 0.0219 |
| 422 | ENSG00000238906;snoU13_chr7_29952144_29952248 | 0.0218 |
| 423 | ENSG00000206909;SNORA70_chr5_170793122_170793256 | 0.0218 |
| 424 | ENSG00000238596;snoU13_chr4_184250451_184250554 | 0.0218 |
| 425 | ENSG00000238708;snoU13_chr2_69894491_69894593 | 0.0215 |
| 426 | ENSG00000206958;SNORA70_chr5_87678589_87678724 | 0.0214 |
| 427 | ENSG00000271907;SNORA35_chrX_114360879_114361007 | 0.0214 |
| 428 | ENSG00000206661;SNORA70_chr8_4985801_4985934 | 0.0213 |
| 429 | ENSG00000238533;snoU13_chr8_103400754_103400863 | 0.0213 |
| 430 | HBII-99;ENSG00000212304;SNORD12_chr20_47897219_47897309 | 0.0213 |
| 431 | HBII-85-28_chr15_25349787_25349880 | 0.0212 |
| 432 | ACA15;ENSG00000207168;SNORA15_chr7_56128162_56128295 | 0.0212 |
| 433 | ENSG00000201847;SNORD31_chr13_107973243_107973311 | 0.0211 |
| 434 | ACA65;ENSG00000221303;SNORA79_chr14_81669038_81669178 | 0.0211 |
| 435 | HBII-382_chr1_109643154_109643236 | 0.0211 |
| 436 | ENSG00000200130;SNORA63_chr7_64792309_64792386 | 0.0210 |
| 437 | ACA14b;ENSG00000207181;SNORA14B_chr1_235291117_235291252 | 0.0210 |
| 438 | ENSG00000222937;SNORD63_chr5_137894659_137894728 | 0.0210 |
| 439 | 14q(II-31);ENSG00000200089;SNORD114-31_chr14_101459572_101459647 | 0.0209 |
| 440 | ENSG00000238972;snoU13_chr14_73376145_73376231 | 0.0209 |
| 441 | ENSG00000200677;SNORD18_chr15_91298473_91298542 | 0.0209 |
| 442 | ENSG00000238934;ACA64_chr1_161110998_161111126 | 0.0208 |
| 443 | ENSG00000207502;SNORA42_chr1_116164493_116164626 | 0.0208 |
| 444 | HBII-419;ENSG00000221182;SNORD98_chr10_70514928_70514995 | 0.0208 |
| 445 | HBII-135_chr17_16344539_16344612 | 0.0208 |
| 446 | ENSG00000252969;SNORA70_chr1_12281205_12281328 | 0.0208 |
| 447 | ENSG00000199666;U3_chr1_91123307_91123520 | 0.0208 |
| 448 | ENSG00000200891;SNORD74_chr10_89754375_89754452 | 0.0207 |
| 449 | ENSG00000253094;SNORD36_chr13_23377284_23377363 | 0.0205 |
| 450 | ENSG00000206649;SNORA20_chr8_81229174_81229304 | 0.0205 |
| 451 | 14q(II-8)_chr14_101431117_101431188 | 0.0205 |
| 452 | ENSG00000207118;SNORD14D_chr11_122929617_122929703 | 0.0204 |
| 453 | ENSG00000202440;SNORD42_chr4_83323791_83323859 | 0.0203 |
| 454 | ENSG00000238902;snoU13_chr3_192251798_192251901 | 0.0203 |
| 455 | ENSG00000201129;SNORA58_chr1_154232203_154232338 | 0.0201 |
| 456 | U14B;ENSG00000272034;SNORD14A_chr11_17096199_17096291 | 0.0201 |
| 457 | ENSG00000252349;SNORA31_chr17_19565313_19565403 | 0.0201 |
| 458 | ENSG00000212295;SNORD28_chr6_156699884_156699958 | 0.0201 |
| 459 | snR38B;ENSG00000199961;SNORD1B_chr17_74557189_74557275 | 0.0200 |
| 460 | ACA59;ENSG00000239149;SNORA59A_chr1_12567299_12567451 | 0.0200 |
| 461 | ENSG00000201348;RNU105B_chr20_8811833_8812040 | 0.0200 |
| 462 | ENSG00000201791;SNORA63_chr1_178722789_178722907 | 0.0199 |
| 463 | ENSG00000251836;U3_chr10_120545264_120545475 | 0.0199 |
| 464 | ENSG00000238768;snoU13_chr11_71815120_71815216 | 0.0198 |
| 465 | ENSG00000222666;U3_chr10_28696969_28697180 | 0.0198 |
| 466 | ACA12;ENSG00000251869;SCARNA23_chrX_24762557_24762687 | 0.0198 |
| 467 | ENSG00000199934;SNORD81_chr1_86057963_86058039 | 0.0198 |
| 468 | ENSG00000251942;SCARNA16_chr2_53697585_53697767 | 0.0198 |
| 469 | ENSG00000238868;snoU13_chr7_140075363_140075466 | 0.0198 |
| 470 | ENSG00000212539;U3_chr18_54624501_54624699 | 0.0198 |
| 471 | ENSG00000201516;SNORA51_chr4_177019314_177019436 | 0.0197 |
| 472 | ACA23;ENSG00000201998;SNORA23_chr11_9450312_9450501 | 0.0197 |
| 473 | ENSG00000238311;snoU13_chr15_65989171_65989274 | 0.0197 |
| 474 | ENSG00000238854;SNORD5_chr8_142457575_142457649 | 0.0197 |
| 475 | ENSG00000212214;SNORA48_chrX_3450158_3450292 | 0.0197 |
| 476 | ENSG00000212144;U8_chr1_234729021_234729148 | 0.0197 |
| 477 | ENSG00000201398;U8_chr3_153725156_153725291 | 0.0197 |
| 478 | ENSG00000239142;U8_chr10_5135600_5135735 | 0.0196 |
| 479 | ENSG00000221496;U3_chr17_42381382_42381570 | 0.0196 |
| 480 | ENSG00000239140;snoU13_chr3_12674233_12674333 | 0.0196 |
| 481 | ENSG00000252258;SNORA70_chr18_3025432_3025564 | 0.0196 |
| 482 | ENSG00000221043;U3_chr5_149075312_149075522 | 0.0196 |
| 483 | ENSG00000251940;SNORA15_chr22_19237396_19237489 | 0.0195 |
| 484 | ACA14a;ENSG00000201643;SNORA14A_chr7_75573100_75573234 | 0.0195 |
| 485 | ENSG00000201502;SNORD74_chr12_93659793_93659872 | 0.0195 |
| 486 | ENSG00000252011;SNORA25_chr1_246996750_246996831 | 0.0194 |
| 487 | ENSG00000221044;U3_chr17_42102389_42102591 | 0.0194 |
| 488 | ACA10;ENSG00000206811;SNORA10_chr16_2012334_2012467 | 0.0194 |
| 489 | ENSG00000239080;snoU13_chrX_135216476_135216579 | 0.0193 |
| 490 | ENSG00000238306;snoU13_chr2_184814060_184814163 | 0.0192 |
| 491 | ENSG00000202374;SNORA62_chr4_68612954_68613104 | 0.0192 |
| 492 | ENSG00000212161;SNORD64_chr1_159821696_159821762 | 0.0192 |
| 493 | ENSG00000239067;snoU13_chr5_118945897_118946000 | 0.0191 |
| 494 | ACA27;ENSG00000207051;SNORA27_chr13_27829537_27829663 | 0.0190 |
| 495 | ENSG00000252129;SNORA74_chr17_15477333_15477512 | 0.0190 |
| 496 | ACA66;ENSG00000252577;SCARNA20_chr17_58308876_58309007 | 0.0190 |
| 497 | ENSG00000212302;SNORD41_chr14_23225970_23226038 | 0.0190 |
| 498 | U14A;ENSG00000201403;SNORD14B_chr11_17097324_17097415 | 0.0190 |
| 499 | ENSG00000238695;snoU13_chr4_142218928_142219032 | 0.0190 |
| 500 | ENSG00000221345;U3_chr6_71836597_71836808 | 0.0190 |
| 501 | ACA22;ENSG00000206634;SNORA22_chr7_65220512_65220646 | 0.0190 |
| 502 | HBII-85-13;ENSG00000207137;SNORD116-13_chr15_25324203_25324297 | 0.0188 |
| 503 | ACA17_chr9_139621198_139621331 | 0.0187 |
| 504 | ENSG00000200693;U3_chr14_64118015_64118217 | 0.0187 |
| 505 | ENSG00000202023;SNORD81_chr7_136972434_136972509 | 0.0187 |
| 506 | U109;ENSG00000238835;SCARNA18_chr5_82360022_82360156 | 0.0187 |
| 507 | ENSG00000202231;SNORA9_chrX_100077609_100077741 | 0.0187 |
| 508 | SNORA38B;ENSG00000200394;SNORA38B_chr17_65736784_65736915 | 0.0186 |
| 509 | ENSG00000212511;U3_chr15_37144841_37145059 | 0.0186 |
| 510 | ENSG00000238999;snoU13_chr6_101405036_101405139 | 0.0185 |
| 511 | ENSG00000252805;U3_chr2_219484524_219484660 | 0.0185 |
| 512 | ENSG00000202482;U3_chrX_71945976_71946190 | 0.0185 |
| 513 | ENSG00000200538;U3_chr17_46459734_46459948 | 0.0184 |
| 514 | ENSG00000239128;snoU13_chr3_47292013_47292116 | 0.0184 |
| 515 | ENSG00000207344;SNORA22_chr7_64526377_64526510 | 0.0184 |
| 516 | ACA37;ENSG00000207233;SNORA37_chr18_51748653_51748782 | 0.0183 |
| 517 | ENSG00000238552;snoU13_chr10_16440654_16440758 | 0.0183 |
| 518 | ENSG00000251944;U3_chr8_20472340_20472437 | 0.0183 |
| 519 | ENSG00000206780;SNORA75_chr4_17322369_17322505 | 0.0183 |
| 520 | ENSG00000206849;SNORA18_chr15_32220506_32220637 | 0.0182 |
| 521 | ENSG00000251778;SNORA3_chr21_43302314_43302409 | 0.0182 |
| 522 | ACA68;ENSG00000252835;SCARNA21_chr17_7809440_7809578 | 0.0181 |
| 523 | ENSG00000201592;snoU2_19_chrX_20154424_20154503 | 0.0181 |
| 524 | ACA25;ENSG00000207112;SNORA25_chr11_93463678_93463812 | 0.0181 |
| 525 | ENSG00000201674;U3_chrX_113187479_113187691 | 0.0180 |
| 526 | ENSG00000206731;SNORA36_chr2_27864910_27865036 | 0.0180 |
| 527 | ENSG00000207062;SNORA15_chr7_64530916_64531050 | 0.0179 |
| 528 | ENSG00000252765;SCARNA16_chr1_101598709_101598895 | 0.0179 |
| 529 | ENSG00000221040;U3_chr1_116821228_116821442 | 0.0178 |
| 530 | ENSG00000199212;RNU105C_chr8_55243247_55243455 | 0.0178 |
| 531 | U32B;ENSG00000201330;SNORD32B_chr6_29550026_29550109 | 0.0178 |
| 532 | ENSG00000206647;SNORA2_chr2_10295199_10295333 | 0.0178 |
| 533 | ENSG00000252543;U3_chr8_8916049_8916140 | 0.0177 |
| 534 | ENSG00000212445;SNORA48_chr16_68223190_68223324 | 0.0177 |
| 535 | ENSG00000212414;SNORD77_chr8_101016811_101016877 | 0.0177 |
| 536 | ENSG00000202479;SNORD14_chr2_25192257_25192346 | 0.0177 |
| 537 | ENSG00000252669;U3_chr1_153970517_153970627 | 0.0177 |
| 538 | ENSG00000200879;SNORD14E_chr11_122928785_122928869 | 0.0177 |
| 539 | ENSG00000252136;SNORA31_chr4_106027144_106027251 | 0.0176 |
| 540 | ENSG00000238498;snoU13_chr22_42472062_42472165 | 0.0176 |
| 541 | ENSG00000252798;SCARNA6_chr16_21598948_21599185 | 0.0175 |
| 542 | ENSG00000200496;U8_chr11_123172098_123172231 | 0.0175 |
| 543 | ENSG00000238295;snoU13_chr2_178210586_178210689 | 0.0175 |
| 544 | ENSG00000207187;SNORA64_chr2_30410300_30410432 | 0.0175 |
| 545 | ENSG00000199282;SNORA9_chr13_73160818_73160948 | 0.0175 |
| 546 | ACA38;ENSG00000200816;SNORA38_chr6_31590855_31590987 | 0.0174 |
| 547 | U89;ENSG00000238795;SCARNA12_chr12_7076499_7076769 | 0.0174 |
| 548 | ENSG00000238746;snoU13_chr9_97934777_97934880 | 0.0174 |
| 549 | ENSG00000253047;SNORA40_chr1_150573015_150573135 | 0.0173 |
| 550 | ENSG00000207119;U3_chr15_69750281_69750488 | 0.0173 |
| 551 | ENSG00000201003;SNORA58_chr1_54236691_54236826 | 0.0173 |
| 552 | ENSG00000212421;SNORA26_chr9_89875365_89875500 | 0.0172 |
| 553 | ENSG00000238852;snoU13_chr2_223562615_223562719 | 0.0171 |
| 554 | ACA67B;ENSG00000206633;SNORA80B_chr2_10586839_10586975 | 0.0171 |
| 555 | ENSG00000252840;SNORA44_chr1_151500307_151500414 | 0.0170 |
| 556 | ENSG00000238752;snoU13_chr11_65820615_65820699 | 0.0170 |
| 557 | ENSG00000238905;snoU13_chr3_57455872_57455977 | 0.0169 |
| 558 | ENSG00000206901;SNORA72_chr2_140268994_140269125 | 0.0169 |
| 559 | ENSG00000206897;SNORA9_chr12_124101255_124101387 | 0.0168 |
| 560 | ENSG00000201945;SNORA70_chr12_121544846_121544977 | 0.0168 |
| 561 | HBII-52-7_chr15_25427531_25427613 | 0.0168 |
| 562 | ACA30;ENSG00000206755;SNORA30_chr16_30721857_30721986 | 0.0167 |
| 563 | ENSG00000238936;SNORD65_chr8_41284174_41284246 | 0.0167 |
| 564 | ENSG00000200042;U3_chr14_92181260_92181474 | 0.0167 |
| 565 | ENSG00000212363;SNORA40_chr5_74178481_74178608 | 0.0167 |
| 566 | ENSG00000212165;U3_chr20_16963477_16963678 | 0.0167 |
| 567 | ENSG00000251793;U3_chr6_16148518_16148676 | 0.0167 |
| 568 | ENSG00000252175;U3_chrX_54091278_54091399 | 0.0166 |
| 569 | SNORA11C;ENSG00000221459;SNORA11C_chrX_47248047_47248176 | 0.0166 |
| 570 | ENSG00000212279;SNORD77_chr15_74783300_74783369 | 0.0166 |
| 571 | ENSG00000206947;SNORA20_chr7_39368602_39368733 | 0.0166 |
| 572 | ENSG00000238804;snoU13_chr17_47034012_47034115 | 0.0166 |
| 573 | HBII-95B;ENSG00000271852;SNORD11B_chr2_203156054_203156144 | 0.0165 |
| 574 | ENSG00000201898;SNORA72_chr1_224367343_224367469 | 0.0165 |
| 575 | ENSG00000212551;U3_chr11_32103310_32103518 | 0.0165 |
| 576 | ENSG00000252218;SCARNA15_chr6_43511881_43512007 | 0.0164 |
| 577 | U96b;ENSG00000208883;SNORD96B_chrX_109468213_109468291 | 0.0164 |
| 578 | ENSG00000202537;U8_chr2_86574185_86574318 | 0.0164 |
| 579 | ENSG00000238899;snoU13_chr5_57366015_57366118 | 0.0164 |
| 580 | ENSG00000206603;SNORA22_chr7_56123058_56123195 | 0.0163 |
| 581 | ENSG00000206637;SNORA70_chr1_201947589_201947723 | 0.0163 |
| 582 | U107;ENSG00000221716;SNORA11_chrX_54840802_54840933 | 0.0163 |
| 583 | ENSG00000207410;SNORA8_chr17_65267585_65267723 | 0.0163 |
| 584 | ENSG00000200026;U8_chr9_38147425_38147558 | 0.0163 |
| 585 | ENSG00000212273;SNORA40_chr8_134656841_134656968 | 0.0163 |
| 586 | ENSG00000207084;SNORA72_chr3_160414717_160414848 | 0.0162 |
| 587 | ENSG00000212458;SNORA48_chr4_1112672_1112801 | 0.0162 |
| 588 | ENSG00000252337;SNORA31_chr5_105882366_105882504 | 0.0162 |
| 589 | ENSG00000201809;U8_chr12_74158059_74158193 | 0.0162 |
| 590 | ENSG00000199552;SNORA63_chr5_40655065_40655196 | 0.0162 |
| 591 | ENSG00000238297;U3_chr7_107640237_107640427 | 0.0161 |
| 592 | ENSG00000221332;U3_chr6_76108577_76108783 | 0.0161 |
| 593 | ENSG00000201025;SNORD74_chr21_17657017_17657089 | 0.0161 |
| 594 | ENSG00000201816;SNORA73_chr18_53746625_53746825 | 0.0161 |
| 595 | ENSG00000221252;U3_chr6_42379938_42380134 | 0.0161 |
| 596 | ENSG00000200897;SNORD74_chr12_107768524_107768604 | 0.0161 |
| 597 | ENSG00000239035;snoU13_chr15_57570768_57570871 | 0.0160 |
| 598 | U66;ENSG00000207523;SNORA66_chr1_93306275_93306408 | 0.0160 |
| 599 | ENSG00000212149;SNORA40_chr17_41092592_41092719 | 0.0160 |
| 600 | ENSG00000207244;SNORA70_chr8_8819085_8819218 | 0.0160 |
| 601 | ENSG00000252709;U3_chr11_66762950_66763096 | 0.0160 |
| 602 | ENSG00000252868;snoZ278_chr15_29526072_29526183 | 0.0160 |
| 603 | ENSG00000206785;SNORA15_chr7_65225039_65225173 | 0.0160 |
| 604 | SNORD121A;ENSG00000238886;SNORD121A_chr9_33952762_33952852 | 0.0159 |
| 605 | ENSG00000238686;snoU13_chr11_3713273_3713375 | 0.0158 |
| 606 | ENSG00000201300;SNORD115-27_chr15_25465644_25465725 | 0.0158 |
| 607 | ENSG00000212558;SNORA26_chr2_10230332_10230446 | 0.0158 |
| 608 | ACA42;ENSG00000207475;SNORA42_chr1_155889699_155889836 | 0.0158 |
| 609 | HBII-52-37;ENSG00000200638;SNORD115-37_chr15_25483132_25483214 | 0.0157 |
| 610 | ACA64_chr16_2015184_2015311 | 0.0157 |
| 611 | ENSG00000212587;SNORA40_chr6_111169749_111169876 | 0.0157 |
| 612 | ENSG00000201980;SNORA62_chr11_4777875_4778027 | 0.0157 |
| 613 | ENSG00000199856;U3_chr18_7995568_7995782 | 0.0156 |
| 614 | ENSG00000212610;U3_chr1_218715033_218715241 | 0.0156 |
| 615 | ENSG00000238849;snoU13_chr7_23934647_23934753 | 0.0156 |
| 616 | ENSG00000238474;snoU13_chr6_109612458_109612561 | 0.0156 |
| 617 | ENSG00000239146;snoU13_chr3_185160007_185160107 | 0.0155 |
| 618 | ENSG00000221673;U3_chr1_220136028_220136206 | 0.0155 |
| 619 | U36B;ENSG00000200831;SNORD36B_chr9_136216948_136217023 | 0.0155 |
| 620 | ENSG00000200999;SNORD74_chr4_26703931_26704010 | 0.0155 |
| 621 | ENSG00000202189;SNORA30_chr9_20786926_20787054 | 0.0155 |
| 622 | ENSG00000200355;SNORA72_chr3_172489067_172489195 | 0.0155 |
| 623 | ENSG00000221400;U3_chr10_43912698_43912888 | 0.0154 |
| 624 | ENSG00000238694;snoU13_chr4_34967863_34967966 | 0.0154 |
| 625 | ENSG00000222145;SNORA73_chr6_89423961_89424149 | 0.0154 |
| 626 | ENSG00000212434;U3_chrX_68912800_68912998 | 0.0154 |
| 627 | ENSG00000239145;snoU13_chr7_137805834_137805937 | 0.0154 |
| 628 | U19-2;ENSG00000212402;SNORA74B_chr5_172447728_172447932 | 0.0154 |
| 629 | ENSG00000238657;snoU13_chr9_135968439_135968538 | 0.0154 |
| 630 | ENSG00000200051;SNORD45_chr5_139906937_139907013 | 0.0153 |
| 631 | ACA39_chr20_37076725_37076861 | 0.0152 |
| 632 | ENSG00000201376;SNORA70_chr14_51710808_51710942 | 0.0152 |
| 633 | ENSG00000238888;snoU13_chr2_9119024_9119128 | 0.0152 |
| 634 | ENSG00000238674;snoU13_chr5_18844006_18844109 | 0.0151 |
| 635 | ENSG00000201368;U3_chr5_35429166_35429378 | 0.0151 |
| 636 | ENSG00000200222;U3_chr3_64057484_64057695 | 0.0150 |
| 637 | ENSG00000252440;U3_chr9_139496967_139497062 | 0.0150 |
| 638 | ENSG00000253007;SNORA76_chr22_34100772_34100906 | 0.0150 |
| 639 | ENSG00000212187;SNORA26_chr1_212198903_212199024 | 0.0150 |
| 640 | ENSG00000238390;SNORA81_chr21_33036619_33036795 | 0.0150 |
| 641 | SNORA11B;ENSG00000221102;SNORA11B_chr14_91592768_91592897 | 0.0149 |
| 642 | HBII-166;ENSG00000212135;SNORD67_chr11_46783938_46784049 | 0.0149 |
| 643 | ENSG00000252824;SNORA48_chr7_101837356_101837444 | 0.0149 |
| 644 | ENSG00000252404;SCARNA16_chr1_9142755_9142938 | 0.0149 |
| 645 | ENSG00000200288;SNORA18_chr3_177342070_177342197 | 0.0148 |
| 646 | ENSG00000212626;SNORA48_chr18_7301018_7301151 | 0.0148 |
| 647 | ENSG00000238983;snoU13_chr10_75061539_75061642 | 0.0148 |
| 648 | ENSG00000208308;SNORA40_chr2_135894198_135894325 | 0.0147 |
| 649 | ENSG00000201863;SNORA51_chr4_40084603_40084726 | 0.0147 |
| 650 | ENSG00000212624;SNORA26_chr1_52190447_52190568 | 0.0147 |
| 651 | ENSG00000199851;U3_chr6_27983346_27983553 | 0.0146 |
| 652 | ACA49;ENSG00000208892;SNORA49_chr12_132515768_132515905 | 0.0146 |
| 653 | mgU6-53;ENSG00000200785;SNORD8_chr14_21865451_21865560 | 0.0145 |
| 654 | ENSG00000252981;U3_chr2_170672789_170672888 | 0.0145 |
| 655 | ENSG00000221164;SNORA11_chr10_74885838_74885965 | 0.0145 |
| 656 | U45B;ENSG00000201487;SNORD45B_chr1_76255161_76255233 | 0.0144 |
| 657 | ENSG00000253014;U3_chr14_61073785_61073883 | 0.0144 |
| 658 | ENSG00000223111;SNORA74_chr10_51611024_51611224 | 0.0144 |
| 659 | ENSG00000238581;snoU13_chr21_39559551_39559656 | 0.0143 |
| 660 | ACA40;ENSG00000210825;SNORA40_chr11_93468275_93468402 | 0.0143 |
| 661 | ENSG00000252689;SNORA20_chrY_18250128_18250259 | 0.0142 |
| 662 | ENSG00000212277;SNORD43_chr11_74427732_74427794 | 0.0142 |
| 663 | ENSG00000252329;SCARNA16_chr11_12926364_12926535 | 0.0142 |
| 664 | ENSG00000199411;SNORD62_chr9_139988797_139988882 | 0.0142 |
| 665 | ENSG00000238645;snoU13_chr16_53368474_53368576 | 0.0142 |
| 666 | ENSG00000206913;SNORA7_chr11_73963459_73963597 | 0.0142 |
| 667 | ENSG00000253068;SNORD112_chr10_103862019_103862089 | 0.0142 |
| 668 | ENSG00000207199;SNORD38_chr8_38876134_38876202 | 0.0141 |
| 669 | ACA48;ENSG00000209582;SNORA48_chr17_7478030_7478165 | 0.0141 |
| 670 | ENSG00000206987;U8_chr15_32718489_32718621 | 0.0140 |
| 671 | U70;ENSG00000207165;SNORA70_chrX_153628621_153628756 | 0.0140 |
| 672 | ENSG00000200318;U3_chr15_59052666_59052880 | 0.0139 |
| 673 | ENSG00000251979;U3_chr4_160428047_160428180 | 0.0138 |
| 674 | ENSG00000272166;SNORD5_chr3_25597034_25597097 | 0.0138 |
| 675 | ENSG00000212342;SNORA12_chr8_131175218_131175373 | 0.0137 |
| 676 | ACA8;ENSG00000207304;SNORA8_chr11_93465526_93465665 | 0.0137 |
| 677 | ENSG00000201346;U3_chr20_3364876_3365086 | 0.0137 |
| 678 | ENSG00000206898;SNORA51_chr2_10436173_10436306 | 0.0137 |
| 679 | ENSG00000199815;SNORA43_chr3_8973179_8973316 | 0.0136 |
| 680 | U108;ENSG00000212464;SNORA12_chr10_101996912_101997059 | 0.0136 |
| 681 | ENSG00000222489;SNORA79_chr14_20791338_20791485 | 0.0136 |
| 682 | HBII-115;ENSG00000221803;SNORD23_chr19_48259109_48259219 | 0.0136 |
| 683 | ENSG00000252580;SNORA31_chr9_26796171_26796302 | 0.0134 |
| 684 | ENSG00000212590;SNORA26_chr7_152306398_152306519 | 0.0134 |
| 685 | ENSG00000238345;snoU13_chr3_10182834_10182931 | 0.0133 |
| 686 | ENSG00000238781;snoU13_chr7_4729111_4729214 | 0.0132 |
| 687 | ENSG00000238402;snoU13_chr9_75142152_75142255 | 0.0132 |
| 688 | ENSG00000206761;U3_chr14_101611818_101612032 | 0.0132 |
| 689 | ENSG00000252921;U3_chr18_23879079_23879219 | 0.0132 |
| 690 | ACA2a;ENSG00000206612;SNORA2A_chr12_49050430_49050565 | 0.0132 |
| 691 | ENSG00000238425;snoU13_chr18_2774872_2774961 | 0.0131 |
| 692 | U73b;ENSG00000201264;SNORD73_chr4_152023208_152023283 | 0.0129 |
| 693 | ENSG00000212455;SNORA40_chr2_18221872_18221999 | 0.0129 |
| 694 | ENSG00000200545;U3_chr10_19887176_19887391 | 0.0127 |
| 695 | ENSG00000201042;SNORA38_chr12_119326239_119326369 | 0.0127 |
| 696 | ENSG00000201407;SNORA68_chrX_24151303_24151434 | 0.0126 |
| 697 | ENSG00000239114;snoU13_chr16_30290569_30290672 | 0.0125 |
| 698 | ACA51;ENSG00000271798;SNORA51_chr20_2635712_2635844 | 0.0125 |
| 699 | ENSG00000212422;U3_chr7_10262424_10262637 | 0.0125 |
| 700 | ENSG00000207516;SNORA41_chr15_45829448_45829575 | 0.0124 |
| 701 | ENSG00000212266;SNORA40_chr1_118231243_118231364 | 0.0124 |
| 702 | ACA52;ENSG00000199785;SNORA52_chr11_811680_811814 | 0.0124 |
| 703 | ENSG00000199927;U3_chr3_30345813_30346027 | 0.0123 |
| 704 | ENSG00000239153;snoU13_chr11_115497800_115497903 | 0.0123 |
| 705 | ENSG00000200706;SNORD45_chr6_38175050_38175121 | 0.0123 |
| 706 | ENSG00000220986;SNORA50_chr5_68456633_68456766 | 0.0122 |
| 707 | ENSG00000252517;SNORD59_chr2_195528831_195528898 | 0.0122 |
| 708 | ENSG00000212249;U8_chr5_68169781_68169914 | 0.0122 |
| 709 | ENSG00000238770;snoU13_chr2_202946598_202946701 | 0.0121 |
| 710 | ENSG00000238932;snoU13_chr13_45202705_45202809 | 0.0121 |
| 711 | ENSG00000201388;SNORA68_chr19_33099243_33099375 | 0.0121 |
| 712 | ENSG00000252284;SNORD28_chr18_56267863_56267930 | 0.0121 |
| 713 | ENSG00000223182;SNORA74_chr10_51383526_51383726 | 0.0120 |
| 714 | ENSG00000238572;snoU13_chr2_173020757_173020848 | 0.0118 |
| 715 | ENSG00000212321;U3_chrX_121974279_121974497 | 0.0118 |
| 716 | ENSG00000212615;SNORD58_chr14_45557446_45557511 | 0.0118 |
| 717 | ENSG00000251866;SCARNA21_chr1_15868660_15868799 | 0.0116 |
| 718 | ENSG00000201660;SNORA20_chrX_5328163_5328292 | 0.0116 |
| 719 | ENSG00000238771;snoU13_chr1_12799693_12799796 | 0.0115 |
| 720 | ENSG00000200422;SNORD45_chrX_86401736_86401807 | 0.0115 |
| 721 | ACA5;ENSG00000206838;SNORA5A_chr7_45143947_45144081 | 0.0114 |
| 722 | ENSG00000252408;SNORD38_chr19_3521246_3521318 | 0.0114 |
| 723 | ENSG00000199370;U3_chr7_148086741_148086950 | 0.0113 |
| 724 | ENSG00000239055;snoU13_chr9_131511637_131511740 | 0.0112 |
| 725 | ACA34;ENSG00000221491;SNORA34_chr12_49048164_49048301 | 0.0112 |
| 726 | ENSG00000212579;SNORA40_chr6_35619595_35619722 | 0.0111 |
| 727 | U70D_chr16_71732469_71732604 | 0.0111 |
| 728 | ENSG00000251878;SNORD79_chr4_185353120_185353203 | 0.0111 |
| 729 | ENSG00000253049;SNORA43_chr3_3144597_3144699 | 0.0110 |
| 730 | U72;ENSG00000207067;SNORA72_chr8_99054313_99054445 | 0.0110 |
| 731 | ENSG00000252719;SNORA18_chrX_138992063_138992193 | 0.0110 |
| 732 | ENSG00000239065;snoU13_chr8_9724417_9724520 | 0.0110 |
| 733 | ENSG00000252425;SNORA18_chr15_34889659_34889759 | 0.0109 |
| 734 | ENSG00000201329;U3_chr8_43233441_43233652 | 0.0108 |
| 735 | ENSG00000264452;snoZ6_chr21_45858918_45858993 | 0.0106 |
| 736 | ENSG00000238624;snoU13_chr8_27612549_27612639 | 0.0105 |
| 737 | ENSG00000238767;snoU13_chr15_59682407_59682516 | 0.0104 |
| 738 | ENSG00000239193;snoU13_chr16_29550583_29550686 | 0.0104 |
| 739 | ENSG00000253090;SNORA73_chr6_108306863_108306983 | 0.0103 |
| 740 | ENSG00000201944;SNORA72_chr1_205700349_205700480 | 0.0102 |
| 741 | ENSG00000252657;SNORA70_chr17_28104637_28104762 | 0.0102 |
| 742 | ENSG00000238954;snoU13_chr16_21468647_21468750 | 0.0101 |
| 743 | ENSG00000238339;snoU13_chr2_179990263_179990364 | 0.0100 |
| 744 | ENSG00000239005;ACA64_chr4_140357522_140357648 | 0.0099 |
| 745 | ENSG00000238670;snoU13_chr3_121310141_121310241 | 0.0099 |
| 746 | ENSG00000238519;U8_chr15_30677162_30677294 | 0.0098 |
| 747 | U70G;ENSG00000206650;SNORA70G_chr12_69021013_69021155 | 0.0097 |
| 748 | ENSG00000212490;SNORA26_chr4_53614304_53614426 | 0.0097 |
| 749 | ENSG00000252438;SNORD45_chr10_12457927_12457999 | 0.0096 |
| 750 | ENSG00000221060;SNORA11_chr14_70270920_70271048 | 0.0096 |
| 751 | ENSG00000199857;SNORD50_chr4_77323229_77323299 | 0.0096 |
| 752 | ENSG00000201542;SNORA62_chr1_35775875_35776026 | 0.0095 |
| 753 | ENSG00000239037;snoU13_chrX_152616931_152617034 | 0.0095 |
| 754 | ENSG00000252985;SNORD116_chr9_129189172_129189249 | 0.0095 |
| 755 | ENSG00000212175;SNORA12_chr2_55792839_55792986 | 0.0095 |
| 756 | ENSG00000212479;U3_chr21_30552747_30552960 | 0.0092 |
| 757 | ENSG00000239017;snoU13_chrX_48420090_48420189 | 0.0092 |
| 758 | ENSG00000238639;snoU13_chr16_29448818_29448921 | 0.0092 |
| 759 | ENSG00000238851;snoU13_chr21_37630724_37630829 | 0.0090 |
| 760 | ENSG00000238541;snoU13_chr4_57972409_57972511 | 0.0089 |
| 761 | ENSG00000238693;snoU13_chr11_133306833_133306934 | 0.0089 |
| 762 | ENSG00000252923;SCARNA16_chr2_197550872_197551054 | 0.0089 |
| 763 | ENSG00000238463;snoU13_chr13_95862598_95862702 | 0.0088 |
| 764 | ENSG00000221300;SNORD75_chr2_42667517_42667576 | 0.0088 |
| 765 | ENSG00000207022;SNORA51_chr1_93311616_93311739 | 0.0087 |
| 766 | ENSG00000238433;snoU13_chr8_59574800_59574903 | 0.0087 |
| 767 | ENSG00000238712;snoU13_chr16_21900888_21900991 | 0.0087 |
| 768 | ENSG00000238628;snoU13_chr6_88430633_88430736 | 0.0087 |
| 769 | ENSG00000212134;SNORA40_chr20_29964122_29964249 | 0.0085 |
| 770 | ENSG00000252777;SCARNA24_chr1_29016177_29016306 | 0.0085 |
| 771 | ENSG00000201810;U8_chr3_180260489_180260624 | 0.0085 |
| 772 | ENSG00000238372;snoU13_chr8_102859484_102859588 | 0.0084 |
| 773 | ENSG00000207027;SNORA67_chr8_23663742_23663890 | 0.0084 |
| 774 | ENSG00000201701;SNORA25_chr3_32079018_32079145 | 0.0084 |
| 775 | ENSG00000212593;SNORA75_chr16_23453162_23453242 | 0.0084 |
| 776 | ENSG00000238594;snoU13_chr6_150485720_150485822 | 0.0084 |
| 777 | ENSG00000212383;SNORA48_chr12_57255159_57255292 | 0.0083 |
| 778 | ENSG00000207100;SNORA8_chrX_132286782_132286920 | 0.0082 |
| 779 | ACA5b;ENSG00000200656;SNORA5B_chr7_45145566_45145698 | 0.0082 |
| 780 | ENSG00000238383;snoU13_chr4_22351073_22351177 | 0.0081 |
| 781 | ENSG00000238838;snoU13_chr19_38839649_38839750 | 0.0081 |
| 782 | ENSG00000239041;snoU13_chr2_173556027_173556130 | 0.0080 |
| 783 | ENSG00000238462;snoU13_chr2_9672371_9672474 | 0.0080 |
| 784 | ENSG00000239137;snoU13_chr19_55678539_55678642 | 0.0079 |
| 785 | ENSG00000221639;SNORA3_chr4_74129677_74129801 | 0.0079 |
| 786 | ENSG00000252601;SNORA18_chr5_29070603_29070717 | 0.0079 |
| 787 | ENSG00000252241;U3_chr1_193700988_193701074 | 0.0079 |
| 788 | ENSG00000238359;snoU13_chr3_171411001_171411104 | 0.0079 |
| 789 | ENSG00000207299;SNORD56_chr11_89851559_89851629 | 0.0076 |
| 790 | ENSG00000202233;U3_chr7_25304275_25304377 | 0.0075 |
| 791 | ENSG00000238900;snoU13_chr10_12187662_12187765 | 0.0074 |
| 792 | ENSG00000238776;snoU13_chr14_96790045_96790124 | 0.0074 |
| 793 | ENSG00000238430;snoU13_chr1_173629236_173629339 | 0.0073 |
| 794 | ENSG00000251737;snoU13_chr22_18124172_18124285 | 0.0073 |
| 795 | ENSG00000238857;snoU13_chr7_2590504_2590599 | 0.0073 |
| 796 | ENSG00000222588;SNORA19_chr10_120820495_120820623 | 0.0073 |
| 797 | U70C;ENSG00000207268;SNORA70C_chr9_119943344_119943480 | 0.0072 |
| 798 | ENSG00000212567;SNORA57_chr5_40790179_40790306 | 0.0070 |
| 799 | ENSG00000238815;snoU13_chr17_49175535_49175639 | 0.0070 |
| 800 | ENSG00000200113;SNORA51_chr7_37459231_37459370 | 0.0070 |
| 801 | ENSG00000238929;snoU13_chr3_37238232_37238335 | 0.0070 |
| 802 | ENSG00000199783;SNORD56_chr22_25297114_25297181 | 0.0069 |
| 803 | ENSG00000212440;SNORA75_chr12_9439269_9439418 | 0.0069 |
| 804 | ENSG00000238892;snoU13_chr11_66988379_66988487 | 0.0068 |
| 805 | ENSG00000238666;snoU13_chr11_85805234_85805337 | 0.0067 |
| 806 | ENSG00000263723;SNORD39_chr2_238778547_238778625 | 0.0066 |
| 807 | ENSG00000221633;U3_chr3_108737757_108737963 | 0.0066 |
| 808 | ENSG00000239087;snoU13_chr18_9277977_9278077 | 0.0065 |
| 809 | ENSG00000238388;snoU13_chr11_100805005_100805109 | 0.0065 |
| 810 | ENSG00000239068;snoU13_chr4_53376179_53376282 | 0.0064 |
| 811 | ENSG00000252054;SNORA73_chr7_54933511_54933717 | 0.0064 |
| 812 | ENSG00000212181;SNORA48_chr2_149018143_149018277 | 0.0063 |
| 813 | ENSG00000207109;SNORD38_chr3_53367087_53367155 | 0.0062 |
| 814 | ENSG00000252441;SNORA64_chrX_114779969_114780049 | 0.0062 |
| 815 | ENSG00000212415;SNORD77_chr15_33262928_33262992 | 0.0061 |
| 816 | ENSG00000238387;snoU13_chr11_9599575_9599678 | 0.0060 |
| 817 | ENSG00000238683;snoU13_chr16_69742475_69742578 | 0.0060 |
| 818 | ENSG00000239157;snoU13_chr20_48556833_48556937 | 0.0059 |
| 819 | ENSG00000252228;SNORA48_chr6_30100582_30100743 | 0.0058 |
| 820 | ENSG00000207147;SNORA51_chr21_41885071_41885206 | 0.0057 |
| 821 | ENSG00000201710;SNORD113_chr14_101446329_101446403 | 0.0055 |
| 822 | ENSG00000238507;snoU13_chr16_62438155_62438258 | 0.0055 |
| 823 | ENSG00000251735;SNORA25_chr14_47429626_47429749 | 0.0055 |
| 824 | ENSG00000238369;snoU13_chr5_149408912_149409015 | 0.0055 |
| 825 | ENSG00000201733;SNORA43_chr11_66200234_66200373 | 0.0055 |
| 826 | ENSG00000202379;SNORA70_chr3_108293412_108293545 | 0.0053 |
| 827 | ENSG00000202283;SNORA33_chr6_104031010_104031143 | 0.0052 |
| 828 | ENSG00000252682;SNORD59_chr1_154260936_154261003 | 0.0052 |
| 829 | ENSG00000221638;U3_chr2_75855079_75855293 | 0.0052 |
| 830 | ENSG00000212229;SNORD65_chr6_67920301_67920373 | 0.0052 |
| 831 | ENSG00000239093;snoU13_chr3_187141103_187141207 | 0.0051 |
| 832 | ENSG00000200063;SNORA30_chr17_76396016_76396144 | 0.0051 |
| 833 | ENSG00000202377;SNORA25_chr7_115221369_115221496 | 0.0051 |
| 834 | ENSG00000252691;SCARNA18_chr1_26332707_26332790 | 0.0050 |
| 835 | ENSG00000238863;snoU13_chr18_3424257_3424347 | 0.0050 |
| 836 | ENSG00000238570;snoU13_chr2_162415054_162415156 | 0.0050 |
| 837 | ENSG00000252505;SNORA70_chr8_33397558_33397655 | 0.0049 |
| 838 | ENSG00000202343;SNORA2_chr6_149915761_149915895 | 0.0049 |
| 839 | ENSG00000221245;SNORA11_chr4_120490854_120490980 | 0.0049 |
| 840 | ENSG00000238577;snoU13_chr10_116416269_116416353 | 0.0049 |
| 841 | ENSG00000252016;ACA64_chrX_87403793_87403919 | 0.0048 |
| 842 | ENSG00000252722;SCARNA20_chr15_75413877_75414007 | 0.0048 |
| 843 | ENSG00000252531;SNORA73_chr2_3628160_3628322 | 0.0048 |
| 844 | ENSG00000200385;SNORA42_chr14_38189630_38189766 | 0.0047 |
| 845 | ENSG00000212264;SNORD65_chr7_23436065_23436135 | 0.0047 |
| 846 | ENSG00000202059;SNORA1_chr2_203917008_203917141 | 0.0047 |
| 847 | ENSG00000252904;SNORA76_chr5_65257011_65257147 | 0.0047 |
| 848 | ENSG00000238819;SNORD11_chr15_46643860_46643944 | 0.0046 |
| 849 | ENSG00000238688;snoU13_chr5_76079921_76080024 | 0.0045 |
| 850 | ENSG00000238322;snoU13_chr6_25435508_25435590 | 0.0045 |
| 851 | ENSG00000239123;snoU13_chr7_138167225_138167328 | 0.0045 |
| 852 | ENSG00000252143;SCARNA17_chr22_21899186_21899328 | 0.0043 |
| 853 | ENSG00000200075;SNORA25_chr8_130880836_130880962 | 0.0043 |
| 854 | ENSG00000252314;SCARNA18_chr22_21743917_21743999 | 0.0043 |
| 855 | ENSG00000239159;snoU13_chr5_79535306_79535408 | 0.0043 |
| 856 | ENSG00000238821;snoU13_chr1_28843988_28844087 | 0.0042 |
| 857 | ENSG00000238918;snoU13_chr10_73085085_73085188 | 0.0042 |
| 858 | ENSG00000238947;snoU13_chr17_80099539_80099649 | 0.0041 |
| 859 | ENSG00000239182;SNORA35_chrX_117872018_117872145 | 0.0041 |
| 860 | ENSG00000212371;SNORA46_chr14_77932627_77932779 | 0.0040 |
| 861 | ENSG00000221498;SNORA77_chr2_183928963_183929074 | 0.0040 |
| 862 | ENSG00000251818;SCARNA20_chr17_26345796_26345933 | 0.0040 |
| 863 | ENSG00000212533;SNORA75_chr12_31229801_31229950 | 0.0039 |
| 864 | U70F;ENSG00000206869;SNORA70F_chr2_165544152_165544287 | 0.0038 |
| 865 | ENSG00000238436;snoU13_chr12_58062727_58062822 | 0.0038 |
| 866 | ENSG00000201467;SNORA16_chrX_17062503_17062634 | 0.0038 |
| 867 | ENSG00000212228;SNORA48_chr16_11367970_11368093 | 0.0038 |
| 868 | ENSG00000202275;SNORD51_chr14_104263609_104263685 | 0.0038 |
| 869 | ENSG00000238764;snoU13_chrX_17376053_17376156 | 0.0037 |
| 870 | ENSG00000239126;snoU13_chr3_4920283_4920386 | 0.0037 |
| 871 | ENSG00000238975;snoU13_chr1_113195210_113195310 | 0.0037 |
| 872 | ENSG00000264379;SNORD39_chr9_21524306_21524381 | 0.0037 |
| 873 | ENSG00000239008;ACA64_chrX_80112575_80112701 | 0.0037 |
| 874 | ENSG00000238375;snoU13_chr6_37218980_37219082 | 0.0036 |
| 875 | ENSG00000212586;SNORA8_chr6_38790083_38790180 | 0.0036 |
| 876 | ENSG00000251730;SNORA4_chr3_183171916_183172050 | 0.0036 |
| 877 | ENSG00000238901;snoU13_chr8_123683530_123683633 | 0.0036 |
| 878 | ENSG00000251805;SCARNA21_chr2_24496483_24496610 | 0.0035 |
| 879 | ENSG00000252048;U3_chr4_184097914_184098084 | 0.0035 |
| 880 | ENSG00000238824;snoU13_chr9_140017961_140018060 | 0.0035 |
| 881 | ENSG00000207171;SNORA51_chr4_169392560_169392690 | 0.0035 |
| 882 | ENSG00000221125;U3_chr19_50452574_50452784 | 0.0035 |
| 883 | ENSG00000238798;snoU13_chr1_220160853_220160956 | 0.0034 |
| 884 | ENSG00000212347;SNORD77_chrX_45752347_45752414 | 0.0033 |
| 885 | ENSG00000239072;snoU13_chr2_70182777_70182879 | 0.0033 |
| 886 | ENSG00000238853;snoU13_chr14_103255828_103255928 | 0.0033 |
| 887 | ENSG00000266692;snoZ6_chr21_45857004_45857058 | 0.0033 |
| 888 | ENSG00000239191;snoU13_chr5_149923152_149923251 | 0.0032 |
| 889 | ENSG00000239013;snoU13_chr16_48526309_48526412 | 0.0032 |
| 890 | ENSG00000212581;U8_chr2_186526501_186526633 | 0.0032 |
| 891 | ENSG00000212594;U8_chr12_103985890_103986021 | 0.0031 |
| 892 | ENSG00000238437;snoU13_chr11_93530379_93530479 | 0.0031 |
| 893 | 14q(II-2);ENSG00000200823;SNORD114-2_chr14_101418192_101418270 | 0.0031 |
| 894 | ENSG00000252071;snoU13_chr19_47451546_47451631 | 0.0031 |
| 895 | ENSG00000199959;SNORA2_chr1_84743004_84743140 | 0.0031 |
| 896 | ENSG00000252992;SCARNA11_chr11_117134515_117134668 | 0.0030 |
| 897 | ENSG00000212604;SNORA48_chr15_26254087_26254221 | 0.0030 |
| 898 | ENSG00000200191;U8_chr8_72727729_72727860 | 0.0030 |
| 899 | ENSG00000252571;SCARNA17_chr22_20455222_20455364 | 0.0030 |
| 900 | ENSG00000212620;SNORA75_chr4_80764256_80764382 | 0.0030 |
| 901 | ENSG00000238528;snoU13_chr12_66739499_66739602 | 0.0029 |
| 902 | ENSG00000252557;SNORD112_chr7_148983533_148983604 | 0.0029 |
| 903 | ENSG00000252762;SNORA31_chr4_82849466_82849592 | 0.0029 |
| 904 | ENSG00000252473;SNORA67_chr2_39510798_39510932 | 0.0029 |
| 905 | ENSG00000207249;SNORA72_chr2_105349692_105349820 | 0.0029 |
| 906 | ENSG00000252024;SCARNA18_chr22_20455450_20455532 | 0.0029 |
| 907 | ENSG00000251987;SNORD63_chr3_53564349_53564428 | 0.0029 |
| 908 | ENSG00000200235;SNORA27_chr5_139959252_139959377 | 0.0029 |
| 909 | ENSG00000251922;SNORA14_chr10_6059017_6059157 | 0.0029 |
| 910 | U98b;ENSG00000201544;SNORA16B_chr1_212526158_212526292 | 0.0028 |
| 911 | ENSG00000238626;snoU13_chrX_124062368_124062466 | 0.0028 |
| 912 | ENSG00000200377;SNORD56_chr2_154303327_154303397 | 0.0028 |
| 913 | ENSG00000221398;SNORA11_chr21_35828408_35828535 | 0.0028 |
| 914 | ENSG00000238889;snoU13_chr1_25672765_25672868 | 0.0028 |
| 915 | ENSG00000202216;SNORA43_chr2_180799128_180799265 | 0.0028 |
| 916 | ENSG00000201151;SNORD56_chr20_33704939_33705010 | 0.0027 |
| 917 | ENSG00000212580;SNORA75_chr2_50116006_50116124 | 0.0027 |
| 918 | ENSG00000238342;snoU13_chr15_33187401_33187502 | 0.0027 |
| 919 | ENSG00000199633;SNORA63_chr15_78383514_78383639 | 0.0027 |
| 920 | ENSG00000212461;SNORA17_chr12_72033135_72033263 | 0.0026 |
| 921 | ENSG00000252204;SNORA25_chr12_31964022_31964124 | 0.0026 |
| 922 | ENSG00000238679;snoU13_chr1_119801664_119801764 | 0.0026 |
| 923 | ENSG00000206592;SNORA18_chr5_78516765_78516896 | 0.0026 |
| 924 | ENSG00000239044;snoU13_chr7_129984255_129984358 | 0.0026 |
| 925 | ENSG00000200991;SNORA25_chr15_85395729_85395846 | 0.0026 |
| 926 | ENSG00000238832;snoU109_chr7_107243808_107243952 | 0.0026 |
| 927 | ENSG00000201036;SNORD113_chr14_101445339_101445427 | 0.0025 |
| 928 | ENSG00000239089;snoU13_chr20_34708884_34708994 | 0.0025 |
| 929 | ENSG00000238526;snoU13_chr1_150234093_150234196 | 0.0025 |
| 930 | ENSG00000238753;snoU13_chr4_54989267_54989371 | 0.0025 |
| 931 | 14q(II-18);ENSG00000202142;SNORD114-18_chr14_101442161_101442233 | 0.0025 |
| 932 | ENSG00000238661;snoU13_chr12_31456651_31456750 | 0.0025 |
| 933 | ENSG00000252605;SCARNA18_chr22_21899414_21899496 | 0.0024 |
| 934 | ENSG00000252677;SNORA81_chr18_19291911_19292080 | 0.0024 |
| 935 | ENSG00000239052;snoU13_chr2_44466707_44466800 | 0.0024 |
| 936 | ENSG00000212338;SNORA67_chr1_179165608_179165714 | 0.0024 |
| 937 | ENSG00000238458;snoU13_chr6_18402182_18402283 | 0.0024 |
| 938 | ENSG00000238887;snoU13_chr22_41310883_41310986 | 0.0023 |
| 939 | ENSG00000238318;snoU13_chr4_71677963_71678063 | 0.0023 |
| 940 | ENSG00000238592;snoU13_chr12_65154648_65154753 | 0.0023 |
| 941 | ENSG00000238940;snoU13_chr12_102183593_102183693 | 0.0023 |
| 942 | ENSG00000251795;SNORA66_chr1_93303575_93303627 | 0.0023 |
| 943 | ENSG00000252435;snoU13_chr3_128792139_128792241 | 0.0023 |
| 944 | ENSG00000252849;snosnR60_Z15_chr7_131600994_131601081 | 0.0022 |
| 945 | ENSG00000223027;SNORA57_chr10_27077946_27078086 | 0.0022 |
| 946 | ENSG00000238496;snoU13_chr8_9789230_9789333 | 0.0022 |
| 947 | U70E;ENSG00000207221;SNORA70E_chr11_82752505_82752640 | 0.0022 |
| 948 | ENSG00000207002;SNORA5_chr3_123532924_123533054 | 0.0022 |
| 949 | ENSG00000252296;SNORA25_chrX_99355082_99355219 | 0.0021 |
| 950 | ENSG00000239064;snoU13_chr2_71157470_71157545 | 0.0021 |
| 951 | ENSG00000238734;snoU13_chr16_70266882_70266985 | 0.0021 |
| 952 | ENSG00000238583;snoU13_chr15_45783782_45783885 | 0.0020 |
| 953 | ENSG00000253065;SNORA40_chr5_167460096_167460210 | 0.0020 |
| 954 | ENSG00000206878;SNORA51_chr1_228788183_228788307 | 0.0020 |
| 955 | ENSG00000238685;ACA64_chr16_12391054_12391180 | 0.0020 |
| 956 | ENSG00000251830;SNORD46_chr6_24166501_24166602 | 0.0020 |
| 957 | ENSG00000252609;snoU13_chr6_157712343_157712431 | 0.0020 |
| 958 | ENSG00000238673;snoU13_chr7_56168352_56168445 | 0.0020 |
| 959 | ENSG00000238316;snoU13_chr1_26968777_26968880 | 0.0020 |
| 960 | ENSG00000251893;SNORA70_chr12_76082932_76083084 | 0.0019 |
| 961 | HBI-36;ENSG00000208839;SNORA35_chrX_113865258_113865386 | 0.0019 |
| 962 | ENSG00000252993;SNORA25_chr10_93835999_93836122 | 0.0019 |
| 963 | ENSG00000239091;snoU13_chr10_103131609_103131709 | 0.0019 |
| 964 | ENSG00000271922;snoU13_chr3_154047505_154047608 | 0.0019 |
| 965 | ENSG00000238328;snoU13_chr2_101193931_101194019 | 0.0019 |
| 966 | ENSG00000272464;snoU13_chr12_120486234_120486332 | 0.0019 |
| 967 | ENSG00000239025;snoU13_chr15_43000890_43000990 | 0.0019 |
| 968 | ENSG00000252249;SNORA18_chr6_94588824_94588947 | 0.0018 |
| 969 | ENSG00000238334;snoU13_chr5_68471651_68471754 | 0.0018 |
| 970 | ENSG00000238800;snoU13_chr12_101752526_101752627 | 0.0018 |
| 971 | ENSG00000239172;snoU13_chr16_22492795_22492898 | 0.0017 |
| 972 | ENSG00000200294;SNORA36_chr10_73940616_73940747 | 0.0017 |
| 973 | ENSG00000201245;SNORA25_chr13_100436111_100436237 | 0.0017 |
| 974 | ENSG00000238453;snoU13_chr7_77053000_77053105 | 0.0017 |
| 975 | ENSG00000238692;snoU13_chr11_57096333_57096435 | 0.0017 |
| 976 | ENSG00000212565;SNORA68_chr17_46958202_46958329 | 0.0016 |
| 977 | ENSG00000221455;U3_chr6_93500171_93500382 | 0.0016 |
| 978 | 14q(I-2);ENSG00000212384;SNORD113-2_chr14_101393678_101393750 | 0.0016 |
| 979 | ACA29;ENSG00000206910;SNORA29_chr6_160206625_160206765 | 0.0016 |
| 980 | ENSG00000252387;snoU13_chr5_170710663_170710717 | 0.0016 |
| 981 | ENSG00000238568;snoU13_chr3_72622116_72622219 | 0.0015 |
| 982 | ENSG00000238569;snoU13_chr22_38181533_38181614 | 0.0015 |
| 983 | ENSG00000252020;SCARNA17_chr22_21744085_21744227 | 0.0015 |
| 984 | ENSG00000238565;snoU13_chr3_53317216_53317319 | 0.0015 |
| 985 | ENSG00000252679;snoU13_chr11_101927694_101927748 | 0.0015 |
| 986 | ENSG00000252022;SNORD33_chr16_76503405_76503492 | 0.0015 |
| 987 | ENSG00000238736;snoU13_chr2_219193303_219193403 | 0.0014 |
| 988 | ENSG00000252920;SNORA31_chr1_152984958_152985090 | 0.0014 |
| 989 | ENSG00000238986;snoU13_chr1_24993474_24993578 | 0.0014 |
| 990 | ENSG00000239094;snoU13_chr1_226503762_226503865 | 0.0014 |
| 991 | ENSG00000251802;SNORA31_chr7_34932029_34932161 | 0.0014 |
| 992 | ENSG00000253085;SCARNA11_chr1_14022565_14022694 | 0.0014 |
| 993 | ENSG00000201807;SNORA27_chr6_137176836_137176969 | 0.0014 |
| 994 | ENSG00000238910;snoU13_chr22_32349391_32349493 | 0.0013 |
| 995 | ENSG00000238740;snoU13_chr5_68834988_68835091 | 0.0013 |
| 996 | ENSG00000212608;SNORA26_chr3_53421740_53421863 | 0.0013 |
| 997 | ENSG00000238409;snoU13_chr16_11387067_11387170 | 0.0013 |
| 998 | ENSG00000252049;SNORA40_chr10_23455914_23456030 | 0.0013 |
| 999 | HBII-437;ENSG00000239014;SNORD108_chr15_25232071_25232142 | 0.0013 |
| 1000 | ENSG00000239096;snoU13_chr3_179332563_179332666 | 0.0013 |
| 1001 | ENSG00000238546;snoU13_chr2_130184995_130185098 | 0.0013 |
| 1002 | ENSG00000238349;snoU13_chr19_11549997_11550096 | 0.0012 |
| 1003 | ENSG00000238395;snoU13_chr12_49354521_49354619 | 0.0012 |
| 1004 | ENSG00000212391;SNORA48_chr2_227833705_227833838 | 0.0012 |
| 1005 | ENSG00000239161;snoU13_chr2_197079943_197080046 | 0.0012 |
| 1006 | ENSG00000207419;SNORA67_chr6_11710052_11710193 | 0.0012 |
| 1007 | ENSG00000272025;SNORA74_chr2_65385796_65385994 | 0.0012 |
| 1008 | ENSG00000251881;SNORD112_chr15_80434400_80434469 | 0.0012 |
| 1009 | ENSG00000238885;snoU13_chr18_49072717_49072818 | 0.0012 |
| 1010 | ENSG00000251858;SNORA31_chr14_42063666_42063794 | 0.0012 |
| 1011 | ENSG00000252291;SNORA31_chrX_5979685_5979790 | 0.0012 |
| 1012 | ENSG00000212432;SNORA75_chr12_9597654_9597801 | 0.0011 |
| 1013 | ENSG00000253059;SNORA31_chr14_36225533_36225669 | 0.0011 |
| 1014 | ENSG00000238739;snoU13_chr7_90236318_90236422 | 0.0011 |
| 1015 | ENSG00000239188;snoU13_chrX_141961746_141961849 | 0.0011 |
| 1016 | ENSG00000252230;SNORD111_chr19_35128601_35128686 | 0.0011 |
| 1017 | ENSG00000239121;snoU13_chr16_58652579_58652668 | 0.0011 |
| 1018 | ENSG00000207177;SNORA51_chr5_110020495_110020628 | 0.0011 |
| 1019 | ENSG00000199571;SNORA22_chr12_40213552_40213685 | 0.0011 |
| 1020 | ENSG00000252989;SNORA31_chr3_100364965_100365041 | 0.0011 |
| 1021 | ENSG00000252409;SCARNA21_chr3_41729360_41729525 | 0.0011 |
| 1022 | ENSG00000212538;U3_chr1_163892907_163893109 | 0.0010 |
| 1023 | ENSG00000252000;ACA59_chr2_179887984_179888105 | 0.0010 |
| 1024 | ENSG00000201500;SNORD113_chr14_101443726_101443803 | 0.0010 |
| 1025 | ENSG00000238407;snoU13_chr13_98538063_98538166 | 0.0010 |
| 1026 | ENSG00000239133;snoU13_chr7_99011601_99011701 | 0.0010 |
| 1027 | ENSG00000238591;snoU13_chr21_17080978_17081077 | 0.0010 |
| 1028 | ENSG00000253027;SNORA70_chr19_45268207_45268357 | 0.0010 |
| 1029 | ENSG00000238408;snoU13_chr13_40021252_40021353 | 0.0009 |
| 1030 | ENSG00000238576;snoU13_chr1_220310506_220310608 | 0.0009 |
| 1031 | ENSG00000253009;U3_chrX_150070594_150070709 | 0.0009 |
| 1032 | ENSG00000252045;SNORA33_chr21_33910609_33910744 | 0.0009 |
| 1033 | ENSG00000239049;snoU13_chr2_88606879_88606982 | 0.0009 |
| 1034 | ENSG00000238361;snoU13_chr12_93659405_93659508 | 0.0009 |
| 1035 | ENSG00000221139;SNORD23_chr18_19030614_19030717 | 0.0009 |
| 1036 | ENSG00000252380;SNORD112_chr14_61729679_61729750 | 0.0009 |
| 1037 | ENSG00000252526;SNORA70_chr16_50185553_50185642 | 0.0009 |
| 1038 | ENSG00000239079;snoU13_chr11_122596916_122597019 | 0.0009 |
| 1039 | ENSG00000207503;SNORA32_chr21_42911133_42911253 | 0.0009 |
| 1040 | ENSG00000252727;SCARNA11_chr12_8750105_8750236 | 0.0009 |
| 1041 | ENSG00000238791;snoU13_chr8_95918245_95918347 | 0.0009 |
| 1042 | ENSG00000238350;snoU13_chr3_47507839_47507938 | 0.0009 |
| 1043 | ENSG00000207430;U8_chr15_30873387_30873519 | 0.0008 |
| 1044 | ENSG00000264997;SNORD39_chr11_109134221_109134291 | 0.0008 |
| 1045 | ENSG00000238676;snoU13_chr17_7937805_7937902 | 0.0008 |
| 1046 | ENSG00000251967;SNORD77_chr3_46692000_46692080 | 0.0008 |
| 1047 | ENSG00000238440;snoU13_chr12_64749542_64749645 | 0.0008 |
| 1048 | ENSG00000238982;snoU13_chr18_29674467_29674569 | 0.0008 |
| 1049 | ENSG00000238995;snoU13_chr11_82554927_82555027 | 0.0008 |
| 1050 | ENSG00000253060;SCARNA20_chr1_171737210_171737315 | 0.0008 |
| 1051 | ENSG00000252800;SCARNA20_chr14_63945990_63946131 | 0.0008 |
| 1052 | ENSG00000238538;snoU13_chr1_24208745_24208842 | 0.0008 |
| 1053 | ENSG00000251901;snR65_chr13_96366931_96367032 | 0.0008 |
| 1054 | ENSG00000252888;SNORA31_chr10_78595135_78595260 | 0.0007 |
| 1055 | ENSG00000252265;snoU13_chr7_65899494_65899605 | 0.0007 |
| 1056 | ENSG00000252189;U3_chr10_88505597_88505706 | 0.0007 |
| 1057 | ENSG00000238984;snoU13_chr7_137860931_137861028 | 0.0007 |
| 1058 | ENSG00000252238;SNORA31_chr2_4874591_4874674 | 0.0007 |
| 1059 | ENSG00000251828;SNORA31_chr5_79602054_79602187 | 0.0007 |
| 1060 | ENSG00000201465;SNORA51_chr7_137872744_137872875 | 0.0007 |
| 1061 | ENSG00000238611;snoU13_chr6_42473238_42473344 | 0.0007 |
| 1062 | ENSG00000238816;snoU13_chr4_79561254_79561356 | 0.0007 |
| 1063 | ENSG00000252274;SCARNA24_chr17_70814975_70815111 | 0.0007 |
| 1064 | ENSG00000252572;SNORD112_chr18_5579413_5579484 | 0.0007 |
| 1065 | ENSG00000238336;snoU13_chr7_129959119_129959222 | 0.0007 |
| 1066 | ENSG00000238319;snoU13_chr4_184195614_184195717 | 0.0007 |
| 1067 | ENSG00000252227;SNORD36_chr20_46764686_46764766 | 0.0007 |
| 1068 | ENSG00000238466;snoU13_chr8_21889861_21889963 | 0.0007 |
| 1069 | ENSG00000252290;SNORA25_chr1_237718340_237718471 | 0.0007 |
| 1070 | ENSG00000238963;U8_chr6_155226215_155226291 | 0.0007 |
| 1071 | ENSG00000238557;snoU13_chr7_152293008_152293115 | 0.0007 |
| 1072 | ENSG00000252774;SNORA48_chr15_64634738_64634897 | 0.0007 |
| 1073 | ENSG00000252672;snoZ185_chr7_116073268_116073354 | 0.0006 |
| 1074 | ENSG00000212517;SNORA26_chr20_5102063_5102185 | 0.0006 |
| 1075 | ENSG00000212589;SNORA17_chr10_115580218_115580349 | 0.0006 |
| 1076 | ENSG00000239024;snoU13_chr17_2558973_2559076 | 0.0006 |
| 1077 | 14q(II-7);ENSG00000199390;SNORD114-7_chr14_101429390_101429467 | 0.0006 |
| 1078 | ENSG00000200150;SNORD113_chr14_101422577_101422649 | 0.0006 |
| 1079 | ENSG00000252461;SNORA43_chr16_28892801_28892928 | 0.0006 |
| 1080 | ENSG00000212411;SNORD115_chr10_29864235_29864310 | 0.0006 |
| 1081 | ENSG00000239136;snoU13_chr6_163647942_163648037 | 0.0006 |
| 1082 | ENSG00000251926;snoU13_chr10_70472715_70472835 | 0.0006 |
| 1083 | ENSG00000238834;snoU13_chr16_47154830_47154932 | 0.0006 |
| 1084 | ENSG00000201316;SNORA7_chr8_51927581_51927725 | 0.0006 |
| 1085 | ENSG00000221083;SNORA77_chr1_8571854_8571978 | 0.0006 |
| 1086 | ENSG00000238799;snoU13_chr17_59039151_59039254 | 0.0006 |
| 1087 | ENSG00000238822;snoU13_chr12_131299898_131299995 | 0.0006 |
| 1088 | ENSG00000252083;SNORA40_chr5_23972297_23972403 | 0.0006 |
| 1089 | ENSG00000212618;snoMBII-202_chr17_18847312_18847386 | 0.0006 |
| 1090 | ENSG00000252119;ACA64_chr11_119194003_119194119 | 0.0006 |
| 1091 | ENSG00000252870;ACA59_chr11_114998938_114999093 | 0.0006 |
| 1092 | ENSG00000239038;snoU13_chr16_48692292_48692396 | 0.0006 |
| 1093 | ENSG00000238896;snoU13_chr6_11079248_11079346 | 0.0006 |
| 1094 | ENSG00000238403;snoU13_chr17_80540381_80540476 | 0.0006 |
| 1095 | ENSG00000238960;snoU13_chr15_22348678_22348778 | 0.0006 |
| 1096 | ENSG00000252459;SNORA27_chr12_115175509_115175603 | 0.0005 |
| 1097 | ENSG00000238991;snoU13_chr10_90820788_90820888 | 0.0005 |
| 1098 | ENSG00000238914;snoU13_chr12_104114720_104114822 | 0.0005 |
| 1099 | ENSG00000201957;SNORA25_chr3_178886871_178886998 | 0.0005 |
| 1100 | ENSG00000238544;snoU13_chr16_50386100_50386203 | 0.0005 |
| 1101 | ENSG00000212224;SNORA26_chr20_40341118_40341239 | 0.0005 |
| 1102 | ENSG00000238588;snoU13_chr10_101361537_101361637 | 0.0005 |
| 1103 | ENSG00000252109;snoZ178_chr17_50303618_50303707 | 0.0005 |
| 1104 | ENSG00000239130;snoU13_chr10_15928357_15928464 | 0.0005 |
| 1105 | ENSG00000238656;snoU13_chr8_113576104_113576205 | 0.0005 |
| 1106 | ENSG00000253092;SNORA81_chr3_183170355_183170552 | 0.0005 |
| 1107 | ENSG00000239103;snoU13_chr5_122345237_122345335 | 0.0005 |
| 1108 | ENSG00000200536;SNORA25_chr1_110815106_110815229 | 0.0005 |
| 1109 | ENSG00000238630;snoU13_chr19_52637506_52637593 | 0.0005 |
| 1110 | ENSG00000207444;SNORD56B_chr14_71865054_71865124 | 0.0005 |
| 1111 | ENSG00000252192;SNORA9_chr12_122976660_122976788 | 0.0005 |
| 1112 | ENSG00000251740;U8_chr10_5090055_5090173 | 0.0005 |
| 1113 | ENSG00000238718;snoU13_chr14_36256065_36256168 | 0.0005 |
| 1114 | ENSG00000252687;SNORD112_chr6_37151373_37151444 | 0.0005 |
| 1115 | ENSG00000239186;snoU13_chr16_87348326_87348429 | 0.0005 |
| 1116 | ENSG00000238744;snoU13_chr4_169231843_169231946 | 0.0005 |
| 1117 | ENSG00000238343;snoU13_chr16_68347142_68347243 | 0.0005 |
| 1118 | ENSG00000238856;snoU13_chr1_224524493_224524591 | 0.0004 |
| 1119 | ENSG00000239155;snoU13_chr9_30178473_30178575 | 0.0004 |
| 1120 | ENSG00000238895;snoU13_chr12_128882977_128883067 | 0.0004 |
| 1121 | ENSG00000238748;snoU13_chr12_100795923_100796028 | 0.0004 |
| 1122 | ENSG00000252640;snoU109_chr16_68664754_68664871 | 0.0004 |
| 1123 | ENSG00000238702;snoU13_chr5_50617817_50617919 | 0.0004 |
| 1124 | ENSG00000239056;snoU13_chr22_37344522_37344599 | 0.0004 |
| 1125 | ENSG00000199566;SNORA64_chr12_47739949_47740080 | 0.0004 |
| 1126 | ENSG00000239111;snoU13_chr1_112738124_112738228 | 0.0004 |
| 1127 | ENSG00000238840;U8_chr10_5004628_5004760 | 0.0004 |
| 1128 | ENSG00000252844;SNORD112_chr10_103027694_103027765 | 0.0004 |
| 1129 | ENSG00000238575;snoU109_chr18_2555357_2555491 | 0.0004 |
| 1130 | ENSG00000212278;SNORD81_chr5_18236232_18236305 | 0.0004 |
| 1131 | ENSG00000238729;snoU13_chrX_47138526_47138629 | 0.0004 |
| 1132 | ENSG00000252458;SNORA68_chr5_158657202_158657330 | 0.0004 |
| 1133 | ENSG00000238970;snoU13_chr10_59998418_59998521 | 0.0004 |
| 1134 | ENSG00000202434;SNORA4_chr2_198269442_198269578 | 0.0004 |
| 1135 | ENSG00000201393;SNORA71_chr10_80127264_80127380 | 0.0004 |
| 1136 | ENSG00000252114;SNORA73_chr14_23486367_23486590 | 0.0004 |
| 1137 | ENSG00000238530;snoU13_chr9_117327530_117327632 | 0.0004 |
| 1138 | ENSG00000252778;SCARNA20_chr11_8576563_8576693 | 0.0004 |
| 1139 | ENSG00000251938;snoU13_chr3_47130098_47130169 | 0.0004 |
| 1140 | ENSG00000206961;SNORA51_chr2_170584382_170584513 | 0.0004 |
| 1141 | ENSG00000238341;snoU13_chr2_122463543_122463633 | 0.0004 |
| 1142 | ENSG00000238313;snoU13_chr9_38190404_38190504 | 0.0004 |
| 1143 | ENSG00000252706;snoU13_chr7_33155178_33155242 | 0.0004 |
| 1144 | ENSG00000251838;SNORA31_chr11_37723675_37723770 | 0.0004 |
| 1145 | ENSG00000238549;snoU13_chr20_34304393_34304492 | 0.0004 |
| 1146 | ENSG00000238483;snoU13_chr13_46809805_46809908 | 0.0004 |
| 1147 | ENSG00000238612;snoU13_chr17_65403959_65404062 | 0.0004 |
| 1148 | ENSG00000239166;snoU13_chr1_6812779_6812881 | 0.0004 |
| 1149 | ENSG00000238946;snoU13_chr17_1519486_1519589 | 0.0004 |
| 1150 | ENSG00000238665;snoU13_chr13_20449327_20449425 | 0.0004 |
| 1151 | ENSG00000238485;snoU13_chrX_138784446_138784549 | 0.0004 |
| 1152 | ENSG00000238864;snoU13_chr5_31824686_31824789 | 0.0004 |
| 1153 | ENSG00000238428;snoU13_chr2_219049702_219049805 | 0.0004 |
| 1154 | ENSG00000238841;snoU13_chr7_66255341_66255451 | 0.0004 |
| 1155 | ENSG00000200753;SNORD56_chr7_20416954_20417024 | 0.0003 |
| 1156 | ENSG00000212211;U3_chr3_60842062_60842277 | 0.0003 |
| 1157 | ENSG00000212145;U8_chr3_41940603_41940745 | 0.0003 |
| 1158 | ENSG00000199231;snoU2_19_chr7_62636318_62636397 | 0.0003 |
| 1159 | ENSG00000199470;SNORA64_chr7_12740383_12740514 | 0.0003 |
| 1160 | ENSG00000238818;snoU13_chr1_16237392_16237492 | 0.0003 |
| 1161 | ENSG00000251974;SNORA19_chr2_86591259_86591388 | 0.0003 |
| 1162 | ENSG00000252692;SNORD60_chr1_206261008_206261093 | 0.0003 |
| 1163 | ENSG00000238564;snoU13_chr15_40242907_40243009 | 0.0003 |
| 1164 | ENSG00000239154;snoU13_chr11_101929036_101929139 | 0.0003 |
| 1165 | ENSG00000238715;snoU13_chr15_65863311_65863411 | 0.0003 |
| 1166 | ENSG00000238878;snoU13_chr13_22101939_22102039 | 0.0003 |
| 1167 | ENSG00000251744;SNORA25_chr8_140120673_140120777 | 0.0003 |
| 1168 | ENSG00000252693;SNORA40_chrX_123331593_123331699 | 0.0003 |
| 1169 | ENSG00000201209;SNORD42_chr22_28628744_28628811 | 0.0003 |
| 1170 | ENSG00000253067;snoZ6_chr5_131415759_131415826 | 0.0003 |
| 1171 | ENSG00000252170;SNORD112_chr3_121964613_121964684 | 0.0003 |
| 1172 | ENSG00000238642;snoU13_chr3_10175069_10175172 | 0.0003 |
| 1173 | ENSG00000212529;SNORA57_chr5_171679338_171679482 | 0.0003 |
| 1174 | ENSG00000238451;snoU13_chr5_70384459_70384562 | 0.0003 |
| 1175 | ENSG00000252443;SNORA62_chr16_69819897_69820016 | 0.0003 |
| 1176 | ENSG00000238763;snoU13_chr11_65920092_65920196 | 0.0003 |
| 1177 | ENSG00000239098;snoU13_chr7_24301228_24301333 | 0.0003 |
| 1178 | ENSG00000238337;snoU13_chr2_135823202_135823304 | 0.0003 |
| 1179 | ENSG00000238717;snoU13_chr5_56152975_56153078 | 0.0003 |
| 1180 | ENSG00000252683;U3_chr19_57645418_57645635 | 0.0003 |
| 1181 | ENSG00000238525;snoU13_chr3_101220999_101221101 | 0.0003 |
| 1182 | ENSG00000252112;SNORD63_chr17_28573775_28573838 | 0.0003 |
| 1183 | ENSG00000252096;SNORA31_chr20_4849630_4849764 | 0.0003 |
| 1184 | ENSG00000238939;snoU13_chr6_151668162_151668247 | 0.0003 |
| 1185 | ENSG00000238579;snoU13_chr4_57860241_57860336 | 0.0003 |
| 1186 | ENSG00000238992;snoU13_chr3_125164905_125165007 | 0.0003 |
| 1187 | ENSG00000252009;SNORD112_chr14_101463990_101464061 | 0.0003 |
| 1188 | ENSG00000252110;snoU13_chr9_6566332_6566425 | 0.0003 |
| 1189 | ENSG00000252434;SNORD112_chr20_39045515_39045586 | 0.0003 |
| 1190 | ENSG00000253091;U3_chr6_114102518_114102667 | 0.0003 |
| 1191 | ENSG00000238515;snoU13_chr10_23688460_23688528 | 0.0003 |
| 1192 | ENSG00000238309;snoU13_chr18_12533423_12533524 | 0.0003 |
| 1193 | ENSG00000239073;snoU13_chr12_93744392_93744492 | 0.0003 |
| 1194 | ENSG00000252799;snoU13_chr22_21878047_21878123 | 0.0003 |
| 1195 | ENSG00000222604;SNORA7_chr14_75178654_75178748 | 0.0003 |
| 1196 | ENSG00000238298;snoU13_chr9_133325241_133325342 | 0.0003 |
| 1197 | ENSG00000238545;snoU13_chr1_226491963_226492064 | 0.0003 |
| 1198 | ENSG00000252088;snoU13_chr17_45298008_45298091 | 0.0002 |
| 1199 | ENSG00000238663;snoU13_chr2_216167489_216167587 | 0.0002 |
| 1200 | ENSG00000239077;snoU13_chr2_114605710_114605813 | 0.0002 |
| 1201 | ENSG00000212293;SNORA16_chr13_32994527_32994653 | 0.0002 |
| 1202 | ENSG00000239045;snoU13_chr7_151765985_151766090 | 0.0002 |
| 1203 | ENSG00000239141;snoU13_chr3_172692932_172693032 | 0.0002 |
| 1204 | ENSG00000238935;snoU13_chr7_75742116_75742214 | 0.0002 |
| 1205 | ENSG00000238745;snoU13_chr5_137959168_137959271 | 0.0002 |
| 1206 | ENSG00000251817;snoU13_chr1_173250216_173250309 | 0.0002 |
| 1207 | ENSG00000252852;SNORA31_chr8_118325767_118325900 | 0.0002 |
| 1208 | ENSG00000251709;snoU13_chr19_39540545_39540607 | 0.0002 |
| 1209 | ENSG00000238732;snoU13_chr10_43305675_43305778 | 0.0002 |
| 1210 | ENSG00000238582;snoU13_chr2_208938785_208938886 | 0.0002 |
| 1211 | ENSG00000238843;snoU13_chr1_156499122_156499221 | 0.0002 |
| 1212 | ENSG00000238598;snoU13_chr9_78291823_78291928 | 0.0002 |
| 1213 | ENSG00000238511;snoU13_chr1_153726600_153726703 | 0.0002 |
| 1214 | ENSG00000199894;snoU2_19_chr4_111354265_111354344 | 0.0002 |
| 1215 | ENSG00000238966;SNORD112_chr8_41901022_41901093 | 0.0002 |
| 1216 | ENSG00000252853;SNORD112_chr1_206855308_206855380 | 0.0002 |
| 1217 | ENSG00000238522;snoU13_chr13_97709633_97709734 | 0.0002 |
| 1218 | ENSG00000238807;snoU13_chr17_4017902_4018004 | 0.0002 |
| 1219 | ENSG00000222095;SNORD113_chr14_101460594_101460667 | 0.0002 |
| 1220 | ENSG00000238475;snoU13_chr12_63044955_63045058 | 0.0002 |
| 1221 | ENSG00000238595;snoU13_chr8_76805035_76805139 | 0.0002 |
| 1222 | ENSG00000238376;snoU13_chr18_28652470_28652573 | 0.0002 |
| 1223 | ENSG00000239132;snoU13_chr6_76575681_76575783 | 0.0002 |
| 1224 | ENSG00000238394;snoU13_chr7_5525026_5525132 | 0.0002 |
| 1225 | ENSG00000253036;snR65_chr2_146092638_146092740 | 0.0002 |
| 1226 | ENSG00000239066;snoU13_chr22_22872116_22872211 | 0.0002 |
| 1227 | ENSG00000238608;snoU13_chr9_86067188_86067290 | 0.0002 |
| 1228 | ENSG00000239011;snoU13_chr5_118404992_118405095 | 0.0002 |
| 1229 | ENSG00000252122;SNORA76_chr16_75443230_75443364 | 0.0002 |
| 1230 | ENSG00000239100;snoU13_chr15_58892331_58892434 | 0.0002 |
| 1231 | ENSG00000238652;snoU13_chr4_88689637_88689733 | 0.0002 |
| 1232 | ENSG00000251861;SCARNA20_chr1_204697119_204697234 | 0.0002 |
| 1233 | ENSG00000238869;snoU13_chr13_103374152_103374255 | 0.0002 |
| 1234 | ENSG00000251848;snoU13_chrX_2729941_2730021 | 0.0002 |
| 1235 | ENSG00000238865;snoU13_chr12_92938668_92938763 | 0.0002 |
| 1236 | ENSG00000238465;snoU13_chr2_70139827_70139931 | 0.0002 |
| 1237 | ENSG00000251949;SNORD112_chr14_101392317_101392388 | 0.0002 |
| 1238 | ENSG00000239015;snoU13_chr6_111967530_111967630 | 0.0002 |
| 1239 | ENSG00000238802;snoU13_chr4_129677181_129677284 | 0.0002 |
| 1240 | ENSG00000238651;snoU13_chr13_41754117_41754223 | 0.0002 |
| 1241 | ENSG00000239059;snoU13_chr6_34650575_34650679 | 0.0002 |
| 1242 | ENSG00000253028;SNORA31_chr7_52337133_52337265 | 0.0002 |
| 1243 | ENSG00000238859;snoU13_chr1_171451046_171451148 | 0.0002 |
| 1244 | ENSG00000239148;U8_chr10_5046902_5047034 | 0.0002 |
| 1245 | ENSG00000239034;snoU13_chr4_186867237_186867340 | 0.0002 |
| 1246 | ENSG00000238494;snoU13_chr15_44005342_44005445 | 0.0002 |
| 1247 | ENSG00000212395;SNORA67_chr9_134658247_134658345 | 0.0002 |
| 1248 | ENSG00000252638;snoU13_chr1_236464280_236464351 | 0.0002 |
| 1249 | ENSG00000252834;snoR442_chr4_83870321_83870397 | 0.0002 |
| 1250 | ENSG00000251999;SNORA31_chr7_32831426_32831558 | 0.0002 |
| 1251 | ENSG00000252945;snoU83B_chr14_51311343_51311430 | 0.0001 |
| 1252 | ENSG00000238809;snoU13_chr2_62492130_62492233 | 0.0001 |
| 1253 | ENSG00000271794;snoU13_chr1_11229564_11229670 | 0.0001 |
| 1254 | ENSG00000238605;snoU13_chr5_137450891_137450993 | 0.0001 |
| 1255 | ENSG00000238450;snoU13_chr8_71516767_71516872 | 0.0001 |
| 1256 | ENSG00000252525;snoU13_chrX_70660367_70660477 | 0.0001 |
| 1257 | ENSG00000251699;SNORD27_chr9_8888136_8888207 | 0.0001 |
| 1258 | ENSG00000238473;snoU13_chrX_47941136_47941239 | 0.0001 |
| 1259 | ENSG00000222966;SNORA40_chr7_99549656_99549781 | 0.0001 |
| 1260 | ENSG00000238806;snoU13_chr17_15141102_15141200 | 0.0001 |
| 1261 | ENSG00000252602;U8_chr15_30935128_30935255 | 0.0001 |
| 1262 | ENSG00000238377;SNORD61_chr3_99940365_99940436 | 0.0001 |
| 1263 | ENSG00000239135;snoU13_chr17_48322257_48322365 | 0.0001 |
| 1264 | ENSG00000238684;snoU13_chr16_28683554_28683657 | 0.0001 |
| 1265 | ENSG00000238893;snoU13_chr13_21543457_21543555 | 0.0001 |
| 1266 | ENSG00000238422;snoU13_chr8_124278882_124278985 | 0.0001 |
| 1267 | ENSG00000238556;snoU13_chr21_40717300_40717383 | 0.0001 |
| 1268 | ENSG00000239083;snoU13_chr15_20557339_20557434 | 0.0001 |
| 1269 | ENSG00000238654;snoU13_chr9_5890150_5890256 | 0.0001 |
| 1270 | ENSG00000239129;snoU13_chr17_28210024_28210127 | 0.0001 |
| 1271 | ENSG00000202183;SNORA4_chrX_81816650_81816796 | 0.0001 |
| 1272 | ENSG00000251704;SNORA40_chr2_16380471_16380563 | 0.0001 |
| 1273 | ENSG00000212428;SNORD115_chr15_25404368_25404426 | 0.0001 |
| 1274 | ENSG00000238974;snoU13_chr6_108564659_108564762 | 0.0001 |
| 1275 | ENSG00000238931;snoU13_chr1_66037232_66037335 | 0.0001 |
| 1276 | ENSG00000252236;SNORA26_chr1_156161854_156161994 | 0.0001 |
| 1277 | ENSG00000253013;SNORA31_chr4_187051175_187051309 | 0.0001 |
| 1278 | ENSG00000252365;SNORD22_chr13_94021100_94021213 | 0.0001 |
| 1279 | ENSG00000253052;U3_chr12_30052836_30052940 | 0.0001 |
| 1280 | ENSG00000252405;snoMe28S-Am2634_chr16_7481218_7481297 | 0.0001 |
| 1281 | ENSG00000202517;SNORA64_chr3_40280099_40280232 | 0.0001 |
| 1282 | ENSG00000252203;snoR442_chr10_68254457_68254557 | 0.0001 |
| 1283 | ENSG00000238352;snoU13_chr16_28797731_28797834 | 0.0001 |
| 1284 | ENSG00000238354;snoU13_chr7_54427585_54427688 | 0.0001 |
| 1285 | ENSG00000238571;snoU13_chr1_202166756_202166859 | 0.0001 |
| 1286 | ENSG00000199196;SNORA25_chr13_34674848_34674975 | 0.0001 |
| 1287 | ENSG00000201853;snosnR60_Z15_chr2_125886409_125886490 | 0.0001 |
| 1288 | ENSG00000238701;snoU13_chr3_131999462_131999522 | 0.0001 |
| 1289 | ENSG00000253042;SNORA70_chr1_202496438_202496555 | 0.0001 |
| 1290 | 14q(I-1);ENSG00000202191;SNORD113-1_chr14_101391157_101391229 | 0.0001 |
| 1291 | ENSG00000238625;snoU13_chr11_116191061_116191162 | 0.0001 |
| 1292 | ENSG00000238438;snoU13_chr6_1186753_1186855 | 0.0001 |
| 1293 | ENSG00000238472;snoU13_chr10_101779351_101779456 | 0.0001 |
| 1294 | ENSG00000238805;snoU13_chr1_155385349_155385453 | 0.0001 |
| 1295 | ENSG00000252078;SNORD112_chr18_34222576_34222647 | 0.0001 |
| 1296 | ENSG00000238329;snoU13_chr16_18881488_18881591 | 0.0001 |
| 1297 | ENSG00000252883;SNORD112_chr12_62511668_62511739 | 0.0001 |
| 1298 | ENSG00000252946;SNORD112_chr1_204873875_204873946 | 0.0001 |
| 1299 | ENSG00000201384;SNORA32_chr14_77667369_77667483 | 0.0001 |
| 1300 | ENSG00000238459;snoU13_chr7_98482613_98482713 | 0.0001 |
| 1301 | ENSG00000251911;SNORD112_chr7_104611734_104611805 | 0.0001 |
| 1302 | ENSG00000238351;snoU13_chr4_40870886_40870987 | 0.0001 |
| 1303 | ENSG00000252102;SNORA63_chr7_64791632_64791729 | 0.0001 |
| 1304 | ENSG00000239170;snoU13_chr2_233543673_233543775 | 0.0001 |
| 1305 | ENSG00000238486;snoU13_chr19_52172658_52172761 | 0.0001 |
| 1306 | ENSG00000239173;snoU13_chr17_77687493_77687588 | 0.0001 |
| 1307 | ENSG00000238314;snoU13_chr21_26842421_26842513 | 0.0001 |
| 1308 | ENSG00000238491;snoU13_chr3_197066328_197066422 | 0.0001 |
| 1309 | ENSG00000239134;snoU13_chr8_92476263_92476367 | 0.0001 |
| 1310 | ENSG00000238687;snoU13_chr8_104677523_104677622 | 0.0001 |
| 1311 | ENSG00000251860;snoU13_chr17_7514499_7514591 | 0.0001 |
| 1312 | ENSG00000252873;SNORD112_chr14_101383915_101383982 | 0.0001 |
| 1313 | ENSG00000252917;SNORA74_chr12_42740863_42741016 | 0.0001 |
| 1314 | ENSG00000239144;snoU13_chr1_49617064_49617175 | 0.0001 |
| 1315 | ENSG00000207432;U8_chr15_30404401_30404533 | 0.0001 |
| 1316 | ENSG00000252295;snoU13_chr5_122131820_122131922 | 0.0001 |
| 1317 | ENSG00000239116;snoU13_chr4_184503348_184503454 | 0.0001 |
| 1318 | ENSG00000238371;snoU13_chr2_16043792_16043895 | 0.0001 |
| 1319 | ENSG00000238481;snoU13_chr2_157278707_157278811 | 0.0001 |
| 1320 | ENSG00000238418;snoU13_chr17_74320277_74320380 | 0.0001 |
| 1321 | ENSG00000252448;SNORA63_chr1_38349909_38349989 | 0.0001 |
| 1322 | ENSG00000201827;SNORA33_chr3_133270030_133270159 | 0.0001 |
| 1323 | ENSG00000238979;snoU13_chr2_88310621_88310721 | 0.0001 |
| 1324 | ENSG00000251846;SNORD36_chrX_153121158_153121222 | 0.0001 |
| 1325 | ENSG00000238355;snoU13_chr10_57327499_57327602 | 0.0001 |
| 1326 | ENSG00000238296;snoU13_chr1_101694220_101694321 | 0.0000 |
| 1327 | ENSG00000212528;SNORD115-47_chr15_25513613_25513698 | 0.0000 |
| 1328 | ENSG00000238492;snoU13_chr14_20232066_20232166 | 0.0000 |
| 1329 | ENSG00000239063;snoU13_chr1_52877114_52877194 | 0.0000 |
| 1330 | ENSG00000265335;snoMe28S-Am2634_chr17_19323592_19323655 | 0.0000 |
| 1331 | ENSG00000238615;snoU13_chr15_25138894_25138995 | 0.0000 |
| 1332 | ENSG00000238566;snoU13_chr8_85832053_85832148 | 0.0000 |
| 1333 | ENSG00000238858;snoU13_chr17_33381407_33381513 | 0.0000 |
| 1334 | ENSG00000238464;snoU13_chr6_74103636_74103736 | 0.0000 |
| 1335 | ENSG00000252014;SNORA70_chr4_132689295_132689404 | 0.0000 |
| 1336 | ENSG00000238756;snoU13_chr2_54066862_54066966 | 0.0000 |
| 1337 | ENSG00000238988;snoU13_chr18_60861822_60861898 | 0.0000 |
| 1338 | ENSG00000239125;snoU13_chr10_112336118_112336219 | 0.0000 |
| 1339 | ENSG00000252354;SNORD112_chr1_172317283_172317357 | 0.0000 |
| 1340 | ENSG00000272272;snoU13_chr9_140315596_140315690 | 0.0000 |
| 1341 | ENSG00000239058;snoU13_chrX_123267134_123267227 | 0.0000 |
| 1342 | ENSG00000206886;SNORA70_chr6_82473741_82473873 | 0.0000 |
| 1343 | ENSG00000238567;snoU13_chr2_172002840_172002937 | 0.0000 |
| 1344 | ENSG00000238714;snoU13_chr8_42083151_42083252 | 0.0000 |
| 1345 | ENSG00000253072;snoMBII-202_chr11_15503354_15503439 | 0.0000 |
| 1346 | ENSG00000251749;U3_chr10_21774567_21774666 | 0.0000 |
| 1347 | ENSG00000238760;snoU13_chr2_97647019_97647121 | 0.0000 |
| 1348 | ENSG00000199473;SNORA63_chr7_17412825_17412950 | 0.0000 |
| 1349 | ENSG00000252771;snoU13_chr21_43024746_43024843 | 0.0000 |
| 1350 | ENSG00000238907;snoU13_chr18_19840539_19840640 | 0.0000 |
| 1351 | ENSG00000252559;SNORD112_chr8_110608472_110608543 | 0.0000 |
| 1352 | ENSG00000238761;snoU13_chr1_112913626_112913729 | 0.0000 |
| 1353 | ENSG00000252565;SNORD112_chr8_10641565_10641629 | 0.0000 |
| 1354 | ENSG00000238978;snoU13_chr14_82928432_82928532 | 0.0000 |
| 1355 | ENSG00000238722;snoU13_chr2_3079444_3079544 | 0.0000 |
| 1356 | ENSG00000201811;SNORA71_chr20_37070679_37070812 | 0.0000 |
| 1357 | ENSG00000239061;snoU13_chr14_102825136_102825239 | 0.0000 |
| 1358 | ENSG00000238401;snoU13_chr1_207212039_207212145 | 0.0000 |
| 1359 | ENSG00000238330;snoU13_chr14_74265718_74265821 | 0.0000 |
| 1360 | ENSG00000206776;SNORA32_chr8_120400588_120400709 | 0.0000 |
| 1361 | ENSG00000238922;snoU13_chr7_110749324_110749413 | 0.0000 |
| 1362 | ENSG00000238620;snoU13_chr10_106159563_106159661 | 0.0000 |
| 1363 | ENSG00000252447;snoU13_chr11_47749098_47749189 | 0.0000 |
| 1364 | ENSG00000238301;snoU13_chr4_47307951_47308054 | 0.0000 |
| 1365 | ENSG00000238690;snoU13_chr2_57243196_57243299 | 0.0000 |
| 1366 | ENSG00000252495;SNORD112_chr1_246970417_246970487 | 0.0000 |
| 1367 | ENSG00000238629;snoU13_chr13_111066552_111066653 | 0.0000 |
| 1368 | ENSG00000238384;snoU13_chr7_94905898_94905996 | 0.0000 |
| 1369 | ENSG00000238416;snoU13_chr3_73131138_73131241 | 0.0000 |
| 1370 | ENSG00000271796;snoU13_chr22_20434077_20434153 | 0.0000 |
| 1371 | ENSG00000238691;snoU13_chr17_18049866_18049969 | 0.0000 |
| 1372 | ENSG00000239026;snoU13_chr5_174877819_174877921 | 0.0000 |
| 1373 | ENSG00000272310;snoU13_chr16_11561497_11561585 | 0.0000 |
| 1374 | ENSG00000238305;snoU13_chr13_100436308_100436411 | 0.0000 |
| 1375 | ENSG00000252878;SNORA70_chr9_67864533_67864627 | 0.0000 |
| 1376 | ENSG00000206853;SNORA51_chr8_60049931_60050061 | 0.0000 |
| 1377 | ENSG00000251844;snoMe28S-Am2634_chr12_97945915_97946006 | 0.0000 |
| 1378 | ENSG00000238891;snoU13_chr3_15358127_15358229 | 0.0000 |
| 1379 | ENSG00000252427;SNORD67_chr11_46780316_46780423 | 0.0000 |
| 1380 | ENSG00000212377;SNORD37_chr13_73028040_73028105 | 0.0000 |
| 1381 | ENSG00000201666;SNORD74_chrX_28942186_28942255 | 0.0000 |
| 1382 | ENSG00000199392;SNORA25_chr18_75143519_75143645 | 0.0000 |
| 1383 | ENSG00000252724;SNORA70_chr9_42037464_42037558 | 0.0000 |
| 1384 | ENSG00000239046;snoU13_chr1_202200792_202200892 | 0.0000 |
| 1385 | ENSG00000238811;snoU13_chrX_112244247_112244356 | 0.0000 |
| 1386 | ENSG00000238400;snoU13_chr5_65526957_65527060 | 0.0000 |
| 1387 | ENSG00000252536;U3_chr20_55182733_55182849 | 0.0000 |
| 1388 | ENSG00000238535;snoU13_chr15_43905880_43905983 | 0.0000 |
| 1389 | ENSG00000252668;snoU13_chr6_4076623_4076707 | 0.0000 |
| 1390 | ENSG00000238938;snoU13_chr6_128934346_128934452 | 0.0000 |
| 1391 | ENSG00000200112;SNORD56_chr12_116290453_116290523 | 0.0000 |
| 1392 | ENSG00000200492;U3_chr6_92829240_92829349 | 0.0000 |
| 1393 | ENSG00000200733;SNORD38_chr13_90489424_90489491 | 0.0000 |
| 1394 | ENSG00000201133;SNORA4_chr7_51003047_51003190 | 0.0000 |
| 1395 | ENSG00000201410;snoZ40_chr3_86174366_86174437 | 0.0000 |
| 1396 | ENSG00000202449;SNORA63_chr4_14692334_14692456 | 0.0000 |
| 1397 | ENSG00000212191;SNORD65_chr4_179607040_179607110 | 0.0000 |
| 1398 | ENSG00000212270;SNORD37_chr14_26275854_26275918 | 0.0000 |
| 1399 | ENSG00000212380;SNORD115-45_chr15_25509657_25509737 | 0.0000 |
| 1400 | ENSG00000212397;snosnR66_chr11_112473077_112473175 | 0.0000 |
| 1401 | ENSG00000221711;SNORD75_chr4_78623900_78623957 | 0.0000 |
| 1402 | ENSG00000223004;SNORD29_chr5_24811590_24811657 | 0.0000 |
| 1403 | ENSG00000223294;SNORD83_chr12_64969445_64969521 | 0.0000 |
| 1404 | ENSG00000238294;snoU13_chr20_55050699_55050801 | 0.0000 |
| 1405 | ENSG00000238312;snoU13_chr3_100920676_100920779 | 0.0000 |
| 1406 | ENSG00000238321;snoU13_chr16_82487925_82488023 | 0.0000 |
| 1407 | ENSG00000238348;snoU13_chr9_19310689_19310786 | 0.0000 |
| 1408 | ENSG00000238368;snoU13_chr2_120674894_120675000 | 0.0000 |
| 1409 | ENSG00000238389;snoU13_chr1_94616974_94617077 | 0.0000 |
| 1410 | ENSG00000238398;snoU13_chr3_162896662_162896763 | 0.0000 |
| 1411 | ENSG00000238414;snoU13_chr10_27157094_27157193 | 0.0000 |
| 1412 | ENSG00000238445;snoU13_chr18_3572938_3573041 | 0.0000 |
| 1413 | ENSG00000238455;snoU13_chr13_57133758_57133859 | 0.0000 |
| 1414 | ENSG00000238480;snoU13_chr3_122469219_122469322 | 0.0000 |
| 1415 | ENSG00000238488;snoU13_chr7_136525840_136525943 | 0.0000 |
| 1416 | ENSG00000238502;snoU13_chr15_102223718_102223819 | 0.0000 |
| 1417 | ENSG00000238506;snoU13_chr7_52391494_52391585 | 0.0000 |
| 1418 | ENSG00000238513;snoU13_chr15_56247899_56248002 | 0.0000 |
| 1419 | ENSG00000238514;snoU13_chr19_29421910_29422012 | 0.0000 |
| 1420 | ENSG00000238521;snoU13_chr2_75645972_75646075 | 0.0000 |
| 1421 | ENSG00000238537;snoU13_chr18_20481487_20481589 | 0.0000 |
| 1422 | ENSG00000238543;snoU13_chr2_157266656_157266758 | 0.0000 |
| 1423 | ENSG00000238559;snoU13_chr15_40899410_40899510 | 0.0000 |
| 1424 | ENSG00000238587;snoU13_chr7_87253473_87253574 | 0.0000 |
| 1425 | ENSG00000238631;snoU13_chr6_134527549_134527652 | 0.0000 |
| 1426 | ENSG00000238646;snoU13_chr3_32214148_32214252 | 0.0000 |
| 1427 | ENSG00000238662;snoU13_chr2_167215240_167215343 | 0.0000 |
| 1428 | ENSG00000238696;snoU13_chr2_65135721_65135820 | 0.0000 |
| 1429 | ENSG00000238699;snoU13_chr16_29077576_29077679 | 0.0000 |
| 1430 | ENSG00000238703;snoU13_chr16_28453987_28454090 | 0.0000 |
| 1431 | ENSG00000238724;snoU13_chr11_114397851_114397954 | 0.0000 |
| 1432 | ENSG00000238733;snoU13_chr7_128701585_128701688 | 0.0000 |
| 1433 | ENSG00000238747;snoU13_chr6_90533014_90533115 | 0.0000 |
| 1434 | ENSG00000238769;snoU13_chr12_77556706_77556805 | 0.0000 |
| 1435 | ENSG00000238775;snoU13_chr6_111284669_111284772 | 0.0000 |
| 1436 | ENSG00000238792;snoU13_chr9_96995760_96995863 | 0.0000 |
| 1437 | ENSG00000238796;snoU13_chr5_133186548_133186648 | 0.0000 |
| 1438 | ENSG00000238801;snoU13_chr6_4643996_4644088 | 0.0000 |
| 1439 | ENSG00000238855;snoU13_chr11_126151552_126151649 | 0.0000 |
| 1440 | ENSG00000238871;snoU13_chr1_38215874_38215974 | 0.0000 |
| 1441 | ENSG00000238872;snoU13_chr1_174169267_174169368 | 0.0000 |
| 1442 | ENSG00000238874;snoU13_chr3_128792938_128793045 | 0.0000 |
| 1443 | ENSG00000238920;snoU13_chrX_40144349_40144452 | 0.0000 |
| 1444 | ENSG00000238925;snoU13_chr4_58358435_58358539 | 0.0000 |
| 1445 | ENSG00000238945;snoU13_chr1_45824324_45824420 | 0.0000 |
| 1446 | ENSG00000238948;snoU13_chr4_103780550_103780653 | 0.0000 |
| 1447 | ENSG00000238951;snoU13_chr2_113028848_113028944 | 0.0000 |
| 1448 | ENSG00000238969;snoU13_chrX_32866024_32866122 | 0.0000 |
| 1449 | ENSG00000238981;snoU13_chr15_92378609_92378706 | 0.0000 |
| 1450 | ENSG00000238985;snoU13_chr1_231031178_231031250 | 0.0000 |
| 1451 | ENSG00000238996;snoU13_chr9_94907365_94907468 | 0.0000 |
| 1452 | ENSG00000239000;snoU13_chr10_68374995_68375096 | 0.0000 |
| 1453 | ENSG00000239018;snoU13_chr2_75862267_75862370 | 0.0000 |
| 1454 | ENSG00000239020;snoU13_chr1_17776259_17776359 | 0.0000 |
| 1455 | ENSG00000239031;snoU13_chr18_10389895_10389997 | 0.0000 |
| 1456 | ENSG00000239033;snoU13_chr12_31603104_31603207 | 0.0000 |
| 1457 | ENSG00000239054;snoU13_chr1_223085186_223085289 | 0.0000 |
| 1458 | ENSG00000239084;snoU13_chr5_118804654_118804750 | 0.0000 |
| 1459 | ENSG00000239086;snoU13_chr11_92462496_92462581 | 0.0000 |
| 1460 | ENSG00000239095;snoU13_chr6_112797928_112798029 | 0.0000 |
| 1461 | ENSG00000239171;snoU13_chr21_30761707_30761810 | 0.0000 |
| 1462 | ENSG00000239176;snoU13_chr1_90233771_90233877 | 0.0000 |
| 1463 | ENSG00000239197;snoU109_chr15_92960777_92960919 | 0.0000 |
| 1464 | ENSG00000251700;SNORD112_chr16_70902110_70902171 | 0.0000 |
| 1465 | ENSG00000251715;SNORA68_chr13_73034657_73034739 | 0.0000 |
| 1466 | ENSG00000251721;snoZ5_chr2_159390712_159390796 | 0.0000 |
| 1467 | ENSG00000251762;snoU13_chr6_7040693_7040779 | 0.0000 |
| 1468 | ENSG00000251769;SNORD112_chr14_101382173_101382230 | 0.0000 |
| 1469 | ENSG00000251775;ACA59_chr2_64110383_64110525 | 0.0000 |
| 1470 | ENSG00000251796;SNORA70_chrY_28393531_28393668 | 0.0000 |
| 1471 | ENSG00000251800;U3_chr3_146671746_146671865 | 0.0000 |
| 1472 | ENSG00000251801;SNORD112_chr2_229452519_229452589 | 0.0000 |
| 1473 | ENSG00000251822;SNORA19_chr12_60757603_60757716 | 0.0000 |
| 1474 | ENSG00000251824;SNORD112_chr14_49410650_49410717 | 0.0000 |
| 1475 | ENSG00000251833;snoR26_chr20_6841661_6841755 | 0.0000 |
| 1476 | ENSG00000251847;snoZ13_snr52_chr9_120493653_120493749 | 0.0000 |
| 1477 | ENSG00000251863;SNORD112_chr12_33515518_33515590 | 0.0000 |
| 1478 | ENSG00000251909;U8_chr10_5237847_5237936 | 0.0000 |
| 1479 | ENSG00000251918;SNORD112_chr14_101406904_101406974 | 0.0000 |
| 1480 | ENSG00000251925;SNORA70_chrY_25569246_25569383 | 0.0000 |
| 1481 | ENSG00000251959;snoR442_chr10_17721187_17721296 | 0.0000 |
| 1482 | ENSG00000252040;snoU109_chr16_68664126_68664257 | 0.0000 |
| 1483 | ENSG00000252058;snoU13_chr20_4863030_4863122 | 0.0000 |
| 1484 | ENSG00000252077;SNORD112_chr16_50532072_50532141 | 0.0000 |
| 1485 | ENSG00000252133;SNORA70_chr9_46669492_46669586 | 0.0000 |
| 1486 | ENSG00000252138;SNORA40_chr16_7776843_7776953 | 0.0000 |
| 1487 | ENSG00000252144;SNORD112_chr14_101405405_101405475 | 0.0000 |
| 1488 | ENSG00000252154;SNORD112_chr13_97618195_97618268 | 0.0000 |
| 1489 | ENSG00000252158;SNORA72_chr8_132445023_132445116 | 0.0000 |
| 1490 | ENSG00000252188;SNORA40_chr7_138309805_138309924 | 0.0000 |
| 1491 | ENSG00000252190;SCARNA17_chr1_26332497_26332621 | 0.0000 |
| 1492 | ENSG00000252199;SNORA70_chr21_10385953_10386047 | 0.0000 |
| 1493 | ENSG00000252200;snoZ6_chr19_45229248_45229322 | 0.0000 |
| 1494 | ENSG00000252256;SNORD112_chr9_86096182_86096252 | 0.0000 |
| 1495 | ENSG00000252281;snoZ247_chr2_41961517_41961596 | 0.0000 |
| 1496 | ENSG00000252298;SNORD112_chr2_84085947_84086023 | 0.0000 |
| 1497 | ENSG00000252300;SNORD112_chr4_141196195_141196266 | 0.0000 |
| 1498 | ENSG00000252352;SNORA40_chr17_67351274_67351359 | 0.0000 |
| 1499 | ENSG00000252356;SNORD112_chr19_42648206_42648277 | 0.0000 |
| 1500 | ENSG00000252359;SNORD112_chr1_165041710_165041781 | 0.0000 |
| 1501 | ENSG00000252372;SNORD112_chr15_76109433_76109504 | 0.0000 |
| 1502 | ENSG00000252388;snoU13_chr4_178752503_178752589 | 0.0000 |
| 1503 | ENSG00000252402;snoU13_chr22_21765339_21765415 | 0.0000 |
| 1504 | ENSG00000252476;SNORD112_chr6_137861537_137861608 | 0.0000 |
| 1505 | ENSG00000252502;SNORD112_chr2_33216326_33216391 | 0.0000 |
| 1506 | ENSG00000252537;SNORA31_chr10_16047807_16047922 | 0.0000 |
| 1507 | ENSG00000252550;SNORA25_chr13_106549872_106549992 | 0.0000 |
| 1508 | ENSG00000252566;SNORD112_chr4_108038489_108038561 | 0.0000 |
| 1509 | ENSG00000252576;snR65_chr18_5558188_5558272 | 0.0000 |
| 1510 | ENSG00000252592;SNORD112_chr11_82283051_82283122 | 0.0000 |
| 1511 | ENSG00000252617;SNORA70_chr9_46042781_46042875 | 0.0000 |
| 1512 | ENSG00000252646;SNORD112_chr1_54991059_54991126 | 0.0000 |
| 1513 | ENSG00000252728;SNORD112_chr1_35409357_35409425 | 0.0000 |
| 1514 | ENSG00000252740;SNORD112_chr1_163355655_163355726 | 0.0000 |
| 1515 | ENSG00000252790;SNORD112_chr1_184647068_184647141 | 0.0000 |
| 1516 | ENSG00000252932;SNORD112_chr6_78646918_78646988 | 0.0000 |
| 1517 | ENSG00000252961;SNORD112_chrX_55930020_55930089 | 0.0000 |
| 1518 | ENSG00000253004;U8_chr3_135518536_135518622 | 0.0000 |
| 1519 | ENSG00000253041;SNORA31_chr9_104084759_104084815 | 0.0000 |
| 1520 | ENSG00000253076;SNORD112_chr3_112856813_112856888 | 0.0000 |
| 1521 | ENSG00000271842;snoU13_chr3_175457059_175457164 | 0.0000 |
| 1522 | ENSG00000272015;SNORA62_chr21_40266709_40266791 | 0.0000 |
| 1523 | ENSG00000272179;snoU13_chrX_119678170_119678273 | 0.0000 |
| 1524 | ENSG00000272237;SNORA25_chr2_228752347_228752460 | 0.0000 |
