# Supplementary material for: Analysis of Expression Pattern of snoRNAs in Different Cancer Types with Machine Learning Algorithms
Source: Int J Mol Sci. 2019 May 2;20(9):2185. doi: 10.3390/ijms20092185 (PMC6539089; doi:10.3390/ijms20092185)
Supplement: Supplementary file 1 [file ijms-20-02185-s001.zip › Table S2.docx]

**Table S2.** The produced 62 classification rules for classifying samples from 8 cancers.

| rule1 | (SNORD123;ENSG00000239112;SNORD123_chr5_9548947_9549017 <= 6.24) and (ENSG00000199769;U3_chrX_70065931_70066145 >= 29.809999) and (U18C;ENSG00000199574;SNORD18C_chr15_66793588_66793656 <= 56.610001) => cancer=LGG (319.0/3.0) |
| --- | --- |
| rule2 | (ENSG00000206903;SNORA24_chr15_65577799_65577929 <= 17.02) and (ENSG00000252299;U3_chr9_90989184_90989274 >= 337.230011) and (U49B_chr17_16342822_16342870 <= 32.610001) and (HBII-85-17;ENSG00000206656;SNORD116-17_chr15_25328733_25328827 >= 25.299999) => cancer=LGG (105.0/3.0) |
| rule3 | (SNORD123;ENSG00000239112;SNORD123_chr5_9548947_9549017 <= 6.54) and (14q(I-8);ENSG00000200367;SNORD113-8_chr14_101409787_101409861 >= 37.75) and (ENSG00000199363;SNORA63_chr3_183171602_183171732 >= 0.57) => cancer=LGG (30.0/0.0) |
| rule4 | (HBII-289;ENSG00000212283;SNORD89_chr2_101889397_101889511 <= 41.279999) and (HBII-438B;ENSG00000239169;SNORD109B_chr15_25523489_25523556 >= 10.53) and (14q(II-20);ENSG00000202048;SNORD114-20_chr14_101447340_101447412 >= 5.29) and (ACA13;ENSG00000238363;SNORA13_chr5_111497181_111497314 <= 4.44) => cancer=LGG (33.0/3.0) |
| rule5 | (ENSG00000222345;SNORD19_chr3_52725394_52725469 <= 539.640015) and (ENSG00000252529;RNU3LGG_chr14_85738276_85738405 >= 166.619995) and (SNORD123;ENSG00000239112;SNORD123_chr5_9548947_9549017 <= 14.3) and (14q(II-28);ENSG00000200480;SNORD114-28_chr14_101455466_101455538 >= 4.44) => cancer=LGG (19.0/4.0) |
| rule6 | (SNORD123;ENSG00000239112;SNORD123_chr5_9548947_9549017 <= 0) and (ACA63;ENSG00000221643;SNORA77_chr1_203698708_203698833 >= 21.299999) and (ACA43_chr9_139620555_139620691 <= 3.21) => cancer=LGG (8.0/1.0) |
| rule7 | (14q(II-1);ENSG00000199575;SNORD114-1_chr14_101416169_101416241 >= 4607.350098) and (ACA45_chr15_83424696_83424823 >= 662.539978) => cancer=LGG (5.0/1.0) |
| rule8 | (hTR_chr3_169482397_169482945 >= 2.37) and (HBI-115;ENSG00000238961;SNORA47_chr5_76376258_76376396 <= 23.530001) and (U83B;ENSG00000209480;SNORD83B_chr22_39709823_39709916 >= 410.540009) and (U17b;ENSG00000200087;SNORA73B_chr1_28835069_28835274 <= 68.099998) => cancer=LUSC (105.0/10.0) |
| rule9 | (U83B;ENSG00000209480;SNORD83B_chr22_39709823_39709916 >= 282.850006) and (HBII-85-19;ENSG00000207460;SNORD116-19_chr15_25331672_25331766 <= 116.150002) and (ENSG00000201882;snoU2-30_chrX_20154184_20154253 >= 57.5) and (U3-4_chr17_19015732_19015949 <= 810.01001) and (ENSG00000200072;SNORD44_chr13_112706392_112706452 >= 4.72) => cancer=LUSC (83.0/12.0) |
| rule10 | (HBII-52-6;ENSG00000200812;SNORD115-6_chr15_25425643_25425725 <= 1.41) and (HBII-316;ENSG00000264994;SNORD92_chr2_29136527_29136616 >= 176.369995) and (HBI-115;ENSG00000238961;SNORA47_chr5_76376258_76376396 <= 23.59) and (U83A;ENSG00000209482;SNORD83A_chr22_39711217_39711312 >= 361.119995) and (SNORD127;ENSG00000239043;SNORD127_chr14_45580085_45580171 <= 368.320007) => cancer=LUSC (64.0/14.0) |
| rule11 | (HBII-52-32;ENSG00000200949;SNORD115-32_chr15_25474113_25474195 <= 3.89) and (U30_chr11_62621134_62621204 <= 37981.761719) and (U87;ENSG00000252010;SCARNA5_chr2_234184371_234184649 >= 44.040001) and (SNORD119;ENSG00000251806;SNORD119_chr20_2443604_2443686 >= 592.030029) and (HBII-251;ENSG00000200181;SNORD85_chr1_31441009_31441084 <= 1837.540039) and (HBII-13_chr15_25230246_25230313 >= 97.43) => cancer=LUSC (37.0/7.0) |
| rule12 | (HBII-52-5;ENSG00000200503;SNORD115-5_chr15_25423884_25423966 <= 2.52) and (ACA54;ENSG00000207008;SNORA54_chr11_2985000_2985123 >= 13.46) and (U30_chr11_62621134_62621204 <= 34159.75) and (HBII-85-16;ENSG00000207263;SNORD116-16_chr15_25327913_25328007 <= 55.529999) and (ENSG00000252792;U3_chr14_68212084_68212191 <= 1.41) and (14q(II-6);ENSG00000201263;SNORD114-6_chr14_101423502_101423574 >= 2.45) and (HBII-85-2;ENSG00000207001;SNORD116-2_chr15_25299355_25299452 >= 10.98) and (U84;ENSG00000265236;SNORD84_chr6_31508877_31508955 >= 155.330002) => cancer=LUSC (33.0/1.0) |
| rule13 | (HBII-52-5;ENSG00000200503;SNORD115-5_chr15_25423884_25423966 <= 2.52) and (HBII-316;ENSG00000264994;SNORD92_chr2_29136527_29136616 >= 100.449997) and (snR39B;ENSG00000238942;SNORD2_chr3_186502584_186502654 >= 2234.040039) and (ACA47_chr17_75085388_75085575 >= 4.98) and (ACA31_chr13_45911614_45911744 <= 678.380005) => cancer=LUSC (54.0/12.0) |
| rule14 | (HBII-52-14;ENSG00000199960;SNORD115-14_chr15_25440067_25440148 <= 5.63) and (hTR_chr3_169482397_169482945 >= 1.33) and (U37;ENSG00000206775;SNORD37_chr19_3982504_3982570 >= 441.429993) and (14q(II-27);ENSG00000200636;SNORD114-27_chr14_101454497_101454567 >= 5.04) and (ENSG00000252433;SNORA31_chr1_67568328_67568462 <= 9.61) => cancer=LUSC (23.0/2.0) |
| rule15 | (HBII-52-15;ENSG00000201679;SNORD115-15_chr15_25442722_25442803 <= 2.73) and (U79_chr1_173834485_173834570 <= 88.239998) and (HBI-100;ENSG00000252906;SCARNA3_chr1_175937532_175937676 >= 157.270004) and (U3;ENSG00000263934;SNORD3A_chr17_19091328_19092027 <= 49.27) and (U70B;ENSG00000206937;SNORA70B_chr2_61644378_61644513 <= 1.31) and (ACA63;ENSG00000221643;SNORA77_chr1_203698708_203698833 >= 10.59) => cancer=LUSC (36.0/4.0) |
| rule16 | (HBII-52-15;ENSG00000201679;SNORD115-15_chr15_25442722_25442803 <= 0) and (mgU6-53B;ENSG00000199436;SNORD9_chr14_21860309_21860412 >= 19.389999) and (ACA35;ENSG00000252947;SCARNA1_chr1_28160911_28161077 <= 1.4) and (HBII-295;ENSG00000212447;SNORD90_chr9_125642491_125642602 <= 1562.25) and (ACA31_chr13_45911614_45911744 <= 98.400002) and (14q(II-17);ENSG00000201569;SNORD114-17_chr14_101441142_101441217 >= 16.59) and (ENSG00000238707;SNORD2_chr10_58355723_58355791 >= 12.78) => cancer=LUSC (23.0/2.0) |
| rule17 | (ENSG00000200620;SNORA7_chrX_15734331_15734469 <= 9.42) and (HBI-43;ENSG00000212232;SNORD17_chr20_17943352_17943589 <= 8.36) and (HBII-52-32;ENSG00000200949;SNORD115-32_chr15_25474113_25474195 >= 1.65) and (ENSG00000252787;SNORD19B_chr3_52722898_52722977 >= 7.97) and (ENSG00000212195;U3_chr17_56709003_56709197 <= 14.97) => cancer=PRAD (252.0/4.0) |
| rule18 | (ENSG00000200620;SNORA7_chrX_15734331_15734469 <= 10.5) and (ENSG00000200206;SNORD74_chr15_86716429_86716501 >= 1.31) and (U69;ENSG00000206622;SNORA69_chrX_118921315_118921447 >= 72.940002) and (U32A;ENSG00000201675;SNORD32A_chr19_49993222_49993305 <= 628.23999) => cancer=PRAD (101.0/2.0) |
| rule19 | (U13;ENSG00000239039;SNORD13_chr8_33370991_33371096 <= 297.149994) and (HBII-180C;ENSG00000220988;SNORD88C_chr19_51305581_51305678 >= 111.809998) and (U58B;ENSG00000271982;SNORD58B_chr18_47018033_47018099 <= 306.910004) and (ACA57;ENSG00000251898;SCARNA11_chr12_6690638_6690775 <= 33.610001) and (mgU6-47;ENSG00000207297;SNORD7_chr17_33900675_33900772 <= 64.080002) => cancer=PRAD (72.0/0.0) |
| rule20 | (ENSG00000200620;SNORA7_chrX_15734331_15734469 <= 5.06) and (HBII-336;ENSG00000221740;SNORD93_chr7_22896231_22896305 <= 123.639999) and (HBII-52-15;ENSG00000201679;SNORD115-15_chr15_25442722_25442803 >= 1.01) => cancer=PRAD (40.0/4.0) |
| rule21 | (HBII-180B;ENSG00000221381;SNORD88B_chr19_51302285_51302382 >= 6.91) and (mgU6-47;ENSG00000207297;SNORD7_chr17_33900675_33900772 <= 45.099998) and (U19;ENSG00000200959;SNORA74A_chr5_138614468_138614668 >= 8.24) and (ACA36B;ENSG00000222370;SNORA36B_chr1_220373887_220374018 >= 124.290001) => cancer=PRAD (27.0/1.0) |
| rule22 | (ENSG00000200620;SNORA7_chrX_15734331_15734469 <= 15.15) and (ACA24_chr4_119200344_119200475 >= 671.679993) and (ACA63;ENSG00000221643;SNORA77_chr1_203698708_203698833 <= 33.779999) => cancer=PRAD (18.0/4.0) |
| rule23 | (U58B;ENSG00000271982;SNORD58B_chr18_47018033_47018099 <= 92.089996) and (HBII-296B;ENSG00000275084;SNORD91B_chr17_2232310_2232531 <= 4.27) and (HBII-85-27;ENSG00000251896;SNORD116-27_chr15_25346720_25346814 >= 2.76) and (14q(II-1);ENSG00000199575;SNORD114-1_chr14_101416169_101416241 >= 28.15) and (14q(I-6);ENSG00000200215;SNORD113-6_chr14_101405892_101405968 >= 1.87) => cancer=PRAD (15.0/1.0) |
| rule24 | (HBII-420;ENSG00000221539;SNORD99_chr1_28905254_28905334 <= 435.119995) and (ENSG00000212598;U3_chr3_90079434_90079644 >= 0.39) and (U13;ENSG00000239039;SNORD13_chr8_33370991_33371096 <= 136.429993) => cancer=PRAD (9.0/0.0) |
| rule25 | (HBII-180A;ENSG00000221241;SNORD88A_chr19_51302695_51302792 >= 148.149994) and (14q(II-29);ENSG00000201689;SNORD114-29_chr14_101456427_101456497 <= 0) => cancer=PRAD (4.0/1.0) |
| rule26 | (HBII-85-29;ENSG00000207245;SNORD116-29_chr15_25351666_25351751 <= 36.669998) and (U50_chr6_86387011_86387086 <= 538.840027) and (ACA7;ENSG00000207496;SNORA7A_chr3_12881810_12881949 >= 40.799999) and (ACA36;ENSG00000206948;SNORA36A_chrX_153996802_153996934 >= 6.28) and (ENSG00000265706;SNORD53_SNORD92_chr2_29150849_29150926 <= 27.219999) => cancer=LUAD (107.0/8.0) |
| rule27 | (HBII-52-17;ENSG00000201482;SNORD115-17_chr15_25446469_25446551 <= 3.38) and (mgU6-47;ENSG00000207297;SNORD7_chr17_33900675_33900772 <= 37.040001) and (ACA32;ENSG00000206799;SNORA32_chr11_93464144_93464265 >= 20.290001) and (U81_chr1_173833283_173833360 >= 453.929993) and (HBII-180A;ENSG00000221241;SNORD88A_chr19_51302695_51302792 <= 21.879999) => cancer=LUAD (62.0/3.0) |
| rule28 | (HBII-85-29;ENSG00000207245;SNORD116-29_chr15_25351666_25351751 <= 35.02) and (ACA31_chr13_45911614_45911744 <= 59.080002) and (ENSG00000207215;U3_chr8_98370493_98370702 >= 17.18) and (SNORD127;ENSG00000239043;SNORD127_chr14_45580085_45580171 <= 56.400002) and (ENSG00000212532;SNORD66_chr6_51329488_51329563 <= 2.2) => cancer=LUAD (68.0/14.0) |
| rule29 | (HBII-52-6;ENSG00000200812;SNORD115-6_chr15_25425643_25425725 <= 3.59) and (ACA31_chr13_45911614_45911744 <= 125.900002) and (ACA3;ENSG00000200983;SNORA3_chr11_8705773_8705903 >= 64.470001) and (HBII-210;ENSG00000212452;SNORD69_chr3_52726751_52726828 >= 422.940002) and (mgU6-47;ENSG00000207297;SNORD7_chr17_33900675_33900772 <= 55.389999) and (ACA3-2;ENSG00000212607;SNORA45_chr11_8706985_8707116 >= 47.009998) => cancer=LUAD (70.0/10.0) |
| rule30 | (HBII-85-29;ENSG00000207245;SNORD116-29_chr15_25351666_25351751 <= 28.73) and (U86;ENSG00000212498;SNORD86_chr20_2636742_2636828 <= 1.72) and (U33;ENSG00000199631;SNORD33_chr19_49993872_49993956 >= 119.690002) and (ENSG00000202269;U8_chr5_15110895_15111029 <= 4.66) and (ACA62_chr17_62223698_62223831 >= 6.99) => cancer=LUAD (50.0/11.0) |
| rule31 | (HBII-52-8;ENSG00000200726;SNORD115-8_chr15_25429452_25429534 <= 1.35) and (ENSG00000206976;SNORA7_chr11_3943797_3943933 >= 0.52) and (ACA31_chr13_45911614_45911744 <= 66.139999) and (HBII-296A;ENSG00000212163;SNORD91A_chr17_2233474_2233664 <= 27.01) and (14q(II-27);ENSG00000200636;SNORD114-27_chr14_101454497_101454567 <= 6.79) => cancer=LUAD (35.0/7.0) |
| rule32 | (HBII-52-6;ENSG00000200812;SNORD115-6_chr15_25425643_25425725 <= 3.7) and (ACA32;ENSG00000206799;SNORA32_chr11_93464144_93464265 >= 19.809999) and (U3-4_chr17_19015732_19015949 <= 742.150024) and (snR38A_chr17_74557714_74557786 <= 58.689999) and (ENSG00000200620;SNORA7_chrX_15734331_15734469 >= 39.5) => cancer=LUAD (38.0/9.0) |
| rule33 | (HBII-52-8;ENSG00000200726;SNORD115-8_chr15_25429452_25429534 <= 1.73) and (ACA3;ENSG00000200983;SNORA3_chr11_8705773_8705903 >= 135.149994) and (HBII-234;ENSG00000212534;SNORD70_chr2_203141153_203141241 >= 57.330002) and (snR38A_chr17_74557714_74557786 <= 117.720001) and (HBII-251;ENSG00000200181;SNORD85_chr1_31441009_31441084 <= 3658.649902) => cancer=LUAD (23.0/1.0) |
| rule34 | (HBII-52-17;ENSG00000201482;SNORD115-17_chr15_25446469_25446551 <= 1.63) and (U60;ENSG00000206630;SNORD60_chr16_2205023_2205106 <= 3205.929932) and (mgU2-19/30_chr11_93454679_93455032 >= 10.88) and (ENSG00000252213;SNORA74_chr5_138611869_138612009 <= 1.32) and (ACA61_chr1_28906275_28906405 >= 19.26) => cancer=LUAD (28.0/7.0) |
| rule35 | (ACA60;ENSG00000199266;SNORA60_chr20_37078011_37078147 <= 14.72) and (ENSG00000223213;SNORD81_chr12_54185089_54185131 >= 1.6) and (ENSG00000212532;SNORD66_chr6_51329488_51329563 >= 5.53) and (U83;ENSG00000201785;SNORD117_chr6_31504150_31504226 >= 60.029999) and (14q(II-10);ENSG00000200279;SNORD114-10_chr14_101433388_101433460 >= 1.35) => cancer=LUAD (15.0/1.0) |
| rule36 | (HBI-115;ENSG00000238961;SNORA47_chr5_76376258_76376396 <= 12.05) and (ACA19;ENSG00000207468;SNORA19_chr10_120819522_120819650 >= 23.4) and (mgh18S-121;ENSG00000238578;SNORD4A_chr17_27049599_27049671 >= 330.769989) and (HBII-289;ENSG00000212283;SNORD89_chr2_101889397_101889511 <= 159.070007) => cancer=LUAD (35.0/11.0) |
| rule37 | (HBII-85-27;ENSG00000251896;SNORD116-27_chr15_25346720_25346814 <= 26.65) and (U17b;ENSG00000200087;SNORA73B_chr1_28835069_28835274 >= 28.18) and (ACA26;ENSG00000252808;SCARNA4_chr1_155895748_155895877 <= 99.300003) and (HBII-85-20_chr15_25332807_25332901 <= 26.74) => cancer=HNSC (242.0/4.0) |
| rule38 | (HBII-85-27;ENSG00000251896;SNORD116-27_chr15_25346720_25346814 <= 27.76) and (U97;ENSG00000238622;SNORD97_chr11_10823013_10823155 >= 15.8) and (ACA26;ENSG00000252808;SCARNA4_chr1_155895748_155895877 <= 105.800003) and (U34;ENSG00000202503;SNORD34_chr19_49994161_49994231 >= 174.940002) => cancer=HNSC (70.0/3.0) |
| rule39 | (HBII-85-27;ENSG00000251896;SNORD116-27_chr15_25346720_25346814 <= 26.129999) and (14q(II-12);ENSG00000202270;SNORD114-12_chr14_101435284_101435359 >= 42.34) and (U3-4_chr17_19015732_19015949 >= 556.309998) and (U53;ENSG00000265145;SNORD53_chr2_29149932_29150010 <= 17.719999) => cancer=HNSC (68.0/9.0) |
| rule40 | (HBII-85-29;ENSG00000207245;SNORD116-29_chr15_25351666_25351751 <= 27.85) and (U37;ENSG00000206775;SNORD37_chr19_3982504_3982570 >= 143.720001) and (HBI-115;ENSG00000238961;SNORA47_chr5_76376258_76376396 <= 6.62) and (U17b;ENSG00000200087;SNORA73B_chr1_28835069_28835274 >= 40.41) => cancer=HNSC (45.0/10.0) |
| rule41 | (HBII-85-29;ENSG00000207245;SNORD116-29_chr15_25351666_25351751 <= 11.97) and (14q(II-13);ENSG00000201247;SNORD114-13_chr14_101436215_101436289 >= 6.75) and (HBII-85-27;ENSG00000251896;SNORD116-27_chr15_25346720_25346814 <= 8.08) => cancer=HNSC (31.0/5.0) |
| rule42 | (HBII-52-32;ENSG00000200949;SNORD115-32_chr15_25474113_25474195 <= 2.23) and (HBII-336;ENSG00000221740;SNORD93_chr7_22896231_22896305 >= 354.890015) and (HBII-85-27;ENSG00000251896;SNORD116-27_chr15_25346720_25346814 <= 47.349998) and (ENSG00000238503;SNORD18_chr2_12170429_12170498 >= 13.87) and (ENSG00000200620;SNORA7_chrX_15734331_15734469 <= 32.560001) and (U86;ENSG00000212498;SNORD86_chr20_2636742_2636828 >= 1.69) => cancer=HNSC (30.0/5.0) |
| rule43 | (HBII-85-29;ENSG00000207245;SNORD116-29_chr15_25351666_25351751 <= 10.84) and (HBII-336;ENSG00000221740;SNORD93_chr7_22896231_22896305 >= 377.529999) and (HBII-420;ENSG00000221539;SNORD99_chr1_28905254_28905334 >= 2233.189941) and (14q(I-3);ENSG00000201700;SNORD113-3_chr14_101396255_101396328 >= 1.38) => cancer=HNSC (17.0/4.0) |
| rule44 | (HBII-85-23;ENSG00000207375;SNORD116-23_chr15_25336931_25337025 <= 18.92) and (ENSG00000202252;SNORD14C_chr11_122930043_122930130 >= 354.429993) and (ACA26;ENSG00000252808;SCARNA4_chr1_155895748_155895877 <= 116.839996) and (U90;ENSG00000238741;SCARNA7_chr3_160232694_160233024 >= 3.86) => cancer=HNSC (10.0/0.0) |
| rule45 | (ACA56;ENSG00000206693;SNORA56_chrX_154003272_154003401 >= 17.940001) and (HBII-336;ENSG00000221740;SNORD93_chr7_22896231_22896305 <= 343.98999) and (ENSG00000222345;SNORD19_chr3_52725394_52725469 >= 2101.379883) and (U79_chr1_173834485_173834570 >= 119.029999) => cancer=UCEC (216.0/7.0) |
| rule46 | (U19;ENSG00000200959;SNORA74A_chr5_138614468_138614668 >= 8.54) and (U104;ENSG00000199753;SNORD104_chr17_62223437_62223517 <= 10527.219727) and (ACA35;ENSG00000252947;SCARNA1_chr1_28160911_28161077 >= 1.43) and (ACA47_chr17_75085388_75085575 <= 7.77) => cancer=UCEC (101.0/5.0) |
| rule47 | (HBII-52-4;ENSG00000200680;SNORD115-4_chr15_25421978_25422060 <= 3.02) and (ACA31_chr13_45911614_45911744 >= 241.610001) and (HBII-85-26;ENSG00000251815;SNORD116-26_chr15_25344644_25344742 >= 64.019997) and (U58B;ENSG00000271982;SNORD58B_chr18_47018033_47018099 <= 709.719971) => cancer=UCEC (51.0/1.0) |
| rule48 | (HBII-52-10;ENSG00000201943;SNORD115-10_chr15_25432682_25432763 <= 6.84) and (HBI-115;ENSG00000238961;SNORA47_chr5_76376258_76376396 >= 30.52) and (U53;ENSG00000265145;SNORD53_chr2_29149932_29150010 <= 36.73) and (HBII-108B;ENSG00000238862;SNORD19B_chr3_52724759_52724843 >= 44.209999) => cancer=UCEC (40.0/5.0) |
| rule49 | (HBII-52-14;ENSG00000199960;SNORD115-14_chr15_25440067_25440148 <= 10.24) and (U21;ENSG00000206680;SNORD21_chr1_93302845_93302940 <= 378.980011) and (HBII-251;ENSG00000200181;SNORD85_chr1_31441009_31441084 >= 453.5) => cancer=UCEC (62.0/10.0) |
| rule50 | (U19;ENSG00000200959;SNORA74A_chr5_138614468_138614668 >= 10.49) and (ACA35;ENSG00000252947;SCARNA1_chr1_28160911_28161077 >= 13.82) => cancer=UCEC (28.0/4.0) |
| rule51 | (U104;ENSG00000199753;SNORD104_chr17_62223437_62223517 <= 6110.52002) and (U44_chr1_173835103_173835166 >= 3994.75) => cancer=UCEC (19.0/7.0) |
| rule52 | (U19;ENSG00000200959;SNORA74A_chr5_138614468_138614668 >= 13.94) and (U19;ENSG00000200959;SNORA74A_chr5_138614468_138614668 >= 27.92) => cancer=UCEC (17.0/5.0) |
| rule53 | (U95;ENSG00000264549;SNORD95_chr5_180670312_180670379 <= 1085.359985) and (ENSG00000212182;U3_chr2_114763018_114763232 >= 816.590027) => cancer=UCEC (6.0/2.0) |
| rule54 | (14q(I-3);ENSG00000201700;SNORD113-3_chr14_101396255_101396328 >= 46.150002) and (ENSG00000264346;SNORA77_chr22_20113925_20114049 >= 14.66) => cancer=UCEC (6.0/0.0) |
| rule55 | (HBI-100;ENSG00000252906;SCARNA3_chr1_175937532_175937676 >= 174.110001) and (HBII-52-40;ENSG00000272460;SNORD115-40_chr15_25488760_25488842 >= 6.16) and (snR38A_chr17_74557714_74557786 <= 69.690002) => cancer=THCA (410.0/5.0) |
| rule56 | (HBII-336;ENSG00000221740;SNORD93_chr7_22896231_22896305 <= 218.110001) and (ACA57;ENSG00000251898;SCARNA11_chr12_6690638_6690775 >= 23.809999) and (U50B_chr6_86387306_86387377 >= 32.68) and (14q(I-3);ENSG00000201700;SNORD113-3_chr14_101396255_101396328 <= 18.690001) => cancer=THCA (96.0/2.0) |
| rule57 | (SNORD123;ENSG00000239112;SNORD123_chr5_9548947_9549017 <= 19.299999) and (SNORA36C;ENSG00000207016;SNORA36C_chr2_69747174_69747306 >= 34.560001) => cancer=THCA (32.0/7.0) |
| rule58 | (U60;ENSG00000206630;SNORD60_chr16_2205023_2205106 <= 735.619995) and (ENSG00000207502;SNORA42_chr1_116164493_116164626 >= 0.64) => cancer=THCA (11.0/0.0) |
| rule59 | (ACA36B;ENSG00000222370;SNORA36B_chr1_220373887_220374018 >= 313.950012) and (ACA3;ENSG00000200983;SNORA3_chr11_8705773_8705903 >= 126.199997) => cancer=THCA (18.0/5.0) |
| rule60 | (U45C;ENSG00000206620;SNORD45C_chr1_76252756_76252835 <= 153.220001) and (HBII-85-26;ENSG00000251815;SNORD116-26_chr15_25344644_25344742 >= 82.68) and (14q(II-5);ENSG00000199798;SNORD114-5_chr14_101421706_101421776 <= 2.65) => cancer=THCA (9.0/0.0) |
| rule61 | (HBII-95;ENSG00000238317;SNORD11_chr2_203157772_203157859 <= 0) and (ENSG00000212195;U3_chr17_56709003_56709197 <= 1.51) => cancer=THCA (3.0/0.0) |
| rule62 | Others => cancer=KIRC (749.0/206.0) |
