# Supplementary material for: Analysis of Expression Pattern of snoRNAs in Different Cancer Types with Machine Learning Algorithms
Source: Int J Mol Sci. 2019 May 2;20(9):2185. doi: 10.3390/ijms20092185 (PMC6539089; doi:10.3390/ijms20092185)
Supplement: Supplementary file 1 [file ijms-20-02185-s001.zip › Table S3.docx]

**Table S3.** Corresponding accuracies of individual classes, overall accuracy, and Matthew’s correlation coefficients (MCCs) using a different number of features selected by the incremental feature selection (IFS) method and support vector machine (SVM) classifiers.

| **Feature number** | **HNSC** | **KIRC** | **LGG** | **LUAD** | **LUSC** | **PRAD** | **THCA** | **UCEC** | **Overall accuracy** | **MCC** |
| --- | --- | --- | --- | --- | --- | --- | --- | --- | --- | --- |
| 1 | 0.0000 | 0.1687 | 0.0078 | 0.0000 | 0.0000 | 0.0000 | 0.0895 | 0.9825 | 0.1615 | 0.0696 |
| 2 | 0.3933 | 0.2300 | 0.0039 | 0.0000 | 0.0000 | 0.0000 | 0.1876 | 0.7180 | 0.1983 | 0.0955 |
| 3 | 0.6402 | 0.1772 | 0.0039 | 0.0000 | 0.0000 | 0.0430 | 0.5215 | 0.7513 | 0.2761 | 0.1951 |
| 4 | 1.0000 | 0.2402 | 0.0391 | 0.0000 | 0.0000 | 0.0822 | 0.5559 | 0.0630 | 0.2551 | 0.1818 |
| 5 | 1.0000 | 0.2743 | 0.0371 | 0.0000 | 0.0000 | 0.0729 | 0.5611 | 0.0525 | 0.2576 | 0.1897 |
| 6 | 1.0000 | 0.2862 | 0.0352 | 0.0000 | 0.0000 | 0.0897 | 0.5680 | 0.0473 | 0.2612 | 0.1938 |
| 7 | 1.0000 | 0.3254 | 0.0469 | 0.0036 | 0.0000 | 0.0841 | 0.5611 | 0.0560 | 0.2678 | 0.2007 |
| 8 | 1.0000 | 0.3118 | 0.0547 | 0.0000 | 0.0000 | 0.1813 | 0.5645 | 0.0595 | 0.2790 | 0.2142 |
| 9 | 1.0000 | 0.3186 | 0.0547 | 0.0036 | 0.0000 | 0.2206 | 0.5577 | 0.0420 | 0.2820 | 0.2173 |
| 10 | 0.9947 | 0.3441 | 0.5410 | 0.0054 | 0.0441 | 0.3514 | 0.5250 | 0.0280 | 0.3560 | 0.3167 |
| 11 | 0.9965 | 0.3509 | 0.5332 | 0.0072 | 0.0461 | 0.3626 | 0.5353 | 0.0385 | 0.3607 | 0.3211 |
| 12 | 0.9947 | 0.3475 | 0.5449 | 0.0107 | 0.0461 | 0.3701 | 0.5301 | 0.0228 | 0.3600 | 0.3195 |
| 13 | 0.9859 | 0.4327 | 0.5508 | 0.0394 | 0.1132 | 0.4131 | 0.6988 | 0.0280 | 0.4103 | 0.3740 |
| 14 | 0.9859 | 0.4344 | 0.5469 | 0.0411 | 0.1132 | 0.4037 | 0.6971 | 0.0158 | 0.4074 | 0.3703 |
| 15 | 0.9859 | 0.4412 | 0.5469 | 0.0483 | 0.1113 | 0.4037 | 0.6971 | 0.0175 | 0.4092 | 0.3721 |
| 16 | 0.9859 | 0.4412 | 0.5645 | 0.0537 | 0.1113 | 0.3981 | 0.7022 | 0.0140 | 0.4115 | 0.3740 |
| 17 | 0.8871 | 0.5094 | 0.5625 | 0.1556 | 0.1344 | 0.4037 | 0.6781 | 0.0315 | 0.4230 | 0.3739 |
| 18 | 0.8836 | 0.4974 | 0.5664 | 0.1610 | 0.1344 | 0.4037 | 0.6781 | 0.0333 | 0.4223 | 0.3726 |
| 19 | 0.8801 | 0.5128 | 0.5684 | 0.1717 | 0.1344 | 0.4056 | 0.6850 | 0.0228 | 0.4252 | 0.3753 |
| 20 | 0.8871 | 0.5179 | 0.5605 | 0.1717 | 0.1344 | 0.4093 | 0.6867 | 0.0263 | 0.4270 | 0.3774 |
| 21 | 0.8801 | 0.5162 | 0.5645 | 0.1753 | 0.1344 | 0.4056 | 0.6747 | 0.0280 | 0.4250 | 0.3740 |
| 22 | 0.8836 | 0.5179 | 0.5645 | 0.1771 | 0.1344 | 0.4075 | 0.6781 | 0.0245 | 0.4261 | 0.3753 |
| 23 | 0.8854 | 0.5315 | 0.5645 | 0.1771 | 0.1324 | 0.4075 | 0.6885 | 0.0193 | 0.4286 | 0.3783 |
| 24 | 0.8889 | 0.5349 | 0.5645 | 0.1682 | 0.1324 | 0.4262 | 0.6936 | 0.0420 | 0.4342 | 0.3844 |
| 25 | 0.8889 | 0.5434 | 0.5684 | 0.1610 | 0.1344 | 0.4430 | 0.7022 | 0.0438 | 0.4385 | 0.3894 |
| 26 | 0.8801 | 0.5400 | 0.5684 | 0.1467 | 0.1382 | 0.5439 | 0.6850 | 0.2557 | 0.4728 | 0.4248 |
| 27 | 0.8765 | 0.5417 | 0.5703 | 0.1449 | 0.1363 | 0.5402 | 0.6850 | 0.2680 | 0.4735 | 0.4251 |
| 28 | 0.8783 | 0.5503 | 0.5664 | 0.1431 | 0.1401 | 0.5364 | 0.6919 | 0.2609 | 0.4742 | 0.4260 |
| 29 | 0.8854 | 0.5486 | 0.5664 | 0.1449 | 0.1401 | 0.5402 | 0.6988 | 0.2504 | 0.4751 | 0.4273 |
| 30 | 0.8836 | 0.5417 | 0.5645 | 0.1467 | 0.1401 | 0.5364 | 0.7091 | 0.2522 | 0.4751 | 0.4274 |
| 31 | 0.8854 | 0.5451 | 0.5703 | 0.1324 | 0.1401 | 0.5364 | 0.7108 | 0.2539 | 0.4751 | 0.4276 |
| 32 | 0.8924 | 0.5417 | 0.6230 | 0.1413 | 0.1382 | 0.5776 | 0.7384 | 0.2417 | 0.4895 | 0.4453 |
| 33 | 0.8854 | 0.5809 | 0.6367 | 0.1646 | 0.1440 | 0.6187 | 0.7573 | 0.2977 | 0.5136 | 0.4711 |
| 34 | 0.4780 | 0.5860 | 0.6621 | 0.7496 | 0.1843 | 0.6430 | 0.8072 | 0.3363 | 0.5581 | 0.5080 |
| 35 | 0.4832 | 0.5860 | 0.7090 | 0.7513 | 0.1804 | 0.6467 | 0.8141 | 0.3398 | 0.5658 | 0.5166 |
| 36 | 0.4868 | 0.5911 | 0.7070 | 0.7460 | 0.1823 | 0.6467 | 0.8055 | 0.3398 | 0.5651 | 0.5156 |
| 37 | 0.6420 | 0.5911 | 0.7148 | 0.6404 | 0.1766 | 0.7009 | 0.8072 | 0.3538 | 0.5804 | 0.5306 |
| 38 | 0.6455 | 0.5928 | 0.7520 | 0.6297 | 0.1804 | 0.7103 | 0.8141 | 0.3713 | 0.5888 | 0.5399 |
| 39 | 0.6473 | 0.6184 | 0.7598 | 0.5850 | 0.2284 | 0.7196 | 0.8193 | 0.3888 | 0.5973 | 0.5481 |
| 40 | 0.7160 | 0.6610 | 0.7734 | 0.5134 | 0.2572 | 0.8617 | 0.8847 | 0.4448 | 0.6407 | 0.5968 |
| 41 | 0.6984 | 0.6610 | 0.8105 | 0.5259 | 0.2610 | 0.8804 | 0.8898 | 0.4799 | 0.6522 | 0.6092 |
| 42 | 0.6931 | 0.6610 | 0.8125 | 0.5277 | 0.2802 | 0.8860 | 0.8916 | 0.4834 | 0.6555 | 0.6130 |
| 43 | 0.6914 | 0.6661 | 0.7988 | 0.5313 | 0.2822 | 0.8897 | 0.8933 | 0.4851 | 0.6560 | 0.6135 |
| 44 | 0.6949 | 0.6797 | 0.8164 | 0.5581 | 0.2841 | 0.8953 | 0.9019 | 0.4991 | 0.6675 | 0.6263 |
| 45 | 0.6949 | 0.6831 | 0.8203 | 0.5599 | 0.2860 | 0.8953 | 0.8985 | 0.4921 | 0.6675 | 0.6262 |
| 46 | 0.7037 | 0.6814 | 0.8281 | 0.5277 | 0.2917 | 0.9178 | 0.9019 | 0.4939 | 0.6693 | 0.6287 |
| 47 | 0.7037 | 0.6865 | 0.8301 | 0.5367 | 0.2956 | 0.9178 | 0.9053 | 0.4974 | 0.6727 | 0.6325 |
| 48 | 0.7478 | 0.7104 | 0.8320 | 0.5206 | 0.2975 | 0.9364 | 0.9260 | 0.5131 | 0.6869 | 0.6493 |
| 49 | 0.7496 | 0.7206 | 0.8320 | 0.5224 | 0.2994 | 0.9346 | 0.9346 | 0.5324 | 0.6923 | 0.6553 |
| 50 | 0.7513 | 0.7223 | 0.8340 | 0.5259 | 0.3033 | 0.9364 | 0.9415 | 0.5342 | 0.6952 | 0.6584 |
| 51 | 0.7513 | 0.7189 | 0.8359 | 0.5277 | 0.3033 | 0.9421 | 0.9398 | 0.5359 | 0.6959 | 0.6592 |
| 52 | 0.7531 | 0.7206 | 0.8398 | 0.5295 | 0.3052 | 0.9439 | 0.9415 | 0.5447 | 0.6989 | 0.6624 |
| 53 | 0.7531 | 0.7223 | 0.8340 | 0.5295 | 0.3071 | 0.9439 | 0.9415 | 0.5394 | 0.6979 | 0.6614 |
| 54 | 0.7619 | 0.7223 | 0.8359 | 0.5331 | 0.3052 | 0.9458 | 0.9415 | 0.5569 | 0.7020 | 0.6661 |
| 55 | 0.7637 | 0.7189 | 0.8359 | 0.5367 | 0.3071 | 0.9458 | 0.9398 | 0.5534 | 0.7018 | 0.6659 |
| 56 | 0.7672 | 0.7138 | 0.8379 | 0.5331 | 0.3090 | 0.9458 | 0.9398 | 0.5482 | 0.7009 | 0.6649 |
| 57 | 0.7637 | 0.7189 | 0.8457 | 0.5385 | 0.3282 | 0.9458 | 0.9415 | 0.5587 | 0.7065 | 0.6706 |
| 58 | 0.7743 | 0.7240 | 0.8438 | 0.5438 | 0.3282 | 0.9458 | 0.9398 | 0.5674 | 0.7099 | 0.6746 |
| 59 | 0.7725 | 0.7206 | 0.8398 | 0.5403 | 0.3359 | 0.9439 | 0.9415 | 0.5639 | 0.7088 | 0.6732 |
| 60 | 0.7778 | 0.7411 | 0.8496 | 0.5581 | 0.3225 | 0.9458 | 0.9432 | 0.5674 | 0.7149 | 0.6801 |
| 61 | 0.7778 | 0.7411 | 0.8496 | 0.5617 | 0.3205 | 0.9458 | 0.9415 | 0.5674 | 0.7149 | 0.6802 |
| 62 | 0.7778 | 0.7376 | 0.8516 | 0.5581 | 0.3167 | 0.9458 | 0.9398 | 0.5674 | 0.7135 | 0.6787 |
| 63 | 0.7778 | 0.7376 | 0.8555 | 0.5546 | 0.3205 | 0.9458 | 0.9398 | 0.5709 | 0.7144 | 0.6797 |
| 64 | 0.7795 | 0.7376 | 0.8555 | 0.5510 | 0.3244 | 0.9402 | 0.9415 | 0.5709 | 0.7142 | 0.6793 |
| 65 | 0.7831 | 0.7342 | 0.8555 | 0.5492 | 0.3282 | 0.9402 | 0.9415 | 0.5639 | 0.7135 | 0.6786 |
| 66 | 0.7778 | 0.7530 | 0.8672 | 0.5581 | 0.3359 | 0.9495 | 0.9415 | 0.5762 | 0.7214 | 0.6873 |
| 67 | 0.7795 | 0.7819 | 0.8672 | 0.5510 | 0.3589 | 0.9495 | 0.9449 | 0.5744 | 0.7275 | 0.6936 |
| 68 | 0.7795 | 0.7785 | 0.8711 | 0.5492 | 0.3608 | 0.9533 | 0.9449 | 0.5762 | 0.7282 | 0.6943 |
| 69 | 0.7778 | 0.7802 | 0.8691 | 0.5546 | 0.3608 | 0.9533 | 0.9449 | 0.5762 | 0.7286 | 0.6948 |
| 70 | 0.7743 | 0.7768 | 0.8809 | 0.5581 | 0.3628 | 0.9551 | 0.9449 | 0.5884 | 0.7316 | 0.6979 |
| 71 | 0.7619 | 0.7836 | 0.8887 | 0.5528 | 0.3762 | 0.9570 | 0.9449 | 0.5972 | 0.7340 | 0.7004 |
| 72 | 0.7637 | 0.7836 | 0.8867 | 0.5546 | 0.3781 | 0.9570 | 0.9432 | 0.5989 | 0.7345 | 0.7009 |
| 73 | 0.7707 | 0.8143 | 0.9004 | 0.5492 | 0.4088 | 0.9626 | 0.9466 | 0.6025 | 0.7455 | 0.7134 |
| 74 | 0.7160 | 0.8143 | 0.9004 | 0.6369 | 0.4165 | 0.9607 | 0.9518 | 0.6042 | 0.7512 | 0.7185 |
| 75 | 0.7160 | 0.8143 | 0.9043 | 0.6351 | 0.4165 | 0.9607 | 0.9518 | 0.6095 | 0.7521 | 0.7195 |
| 76 | 0.7160 | 0.8126 | 0.9043 | 0.6351 | 0.4165 | 0.9607 | 0.9518 | 0.6077 | 0.7516 | 0.7190 |
| 77 | 0.7178 | 0.8126 | 0.9043 | 0.6351 | 0.4184 | 0.9607 | 0.9518 | 0.6077 | 0.7521 | 0.7195 |
| 78 | 0.7196 | 0.8194 | 0.9043 | 0.6333 | 0.4184 | 0.9607 | 0.9518 | 0.6060 | 0.7528 | 0.7202 |
| 79 | 0.7213 | 0.8228 | 0.9043 | 0.6351 | 0.4242 | 0.9607 | 0.9518 | 0.6165 | 0.7557 | 0.7235 |
| 80 | 0.7249 | 0.8194 | 0.9063 | 0.6369 | 0.4299 | 0.9589 | 0.9518 | 0.6112 | 0.7559 | 0.7238 |
| 81 | 0.7302 | 0.8177 | 0.9063 | 0.6422 | 0.4280 | 0.9589 | 0.9518 | 0.6095 | 0.7566 | 0.7246 |
| 82 | 0.7266 | 0.8348 | 0.9063 | 0.6440 | 0.4299 | 0.9589 | 0.9501 | 0.6165 | 0.7595 | 0.7279 |
| 83 | 0.7302 | 0.8330 | 0.9063 | 0.6404 | 0.4299 | 0.9589 | 0.9501 | 0.6147 | 0.7591 | 0.7273 |
| 84 | 0.7584 | 0.8330 | 0.9063 | 0.6243 | 0.4280 | 0.9589 | 0.9484 | 0.6165 | 0.7604 | 0.7291 |
| 85 | 0.7584 | 0.8313 | 0.9063 | 0.6225 | 0.4299 | 0.9589 | 0.9501 | 0.6200 | 0.7609 | 0.7296 |
| 86 | 0.7584 | 0.8416 | 0.9063 | 0.6190 | 0.4319 | 0.9589 | 0.9484 | 0.6375 | 0.7640 | 0.7330 |
| 87 | 0.7637 | 0.8484 | 0.9102 | 0.6225 | 0.4395 | 0.9589 | 0.9484 | 0.6375 | 0.7674 | 0.7369 |
| 88 | 0.7637 | 0.8484 | 0.9102 | 0.6279 | 0.4376 | 0.9589 | 0.9484 | 0.6375 | 0.7679 | 0.7374 |
| 89 | 0.7637 | 0.8467 | 0.9082 | 0.6297 | 0.4395 | 0.9589 | 0.9484 | 0.6375 | 0.7679 | 0.7374 |
| 90 | 0.7690 | 0.8467 | 0.9082 | 0.6297 | 0.4472 | 0.9589 | 0.9501 | 0.6462 | 0.7708 | 0.7408 |
| 91 | 0.7690 | 0.8467 | 0.9082 | 0.6333 | 0.4453 | 0.9589 | 0.9501 | 0.6462 | 0.7710 | 0.7411 |
| 92 | 0.7690 | 0.8467 | 0.9082 | 0.6333 | 0.4472 | 0.9589 | 0.9501 | 0.6497 | 0.7717 | 0.7418 |
| 93 | 0.7690 | 0.8467 | 0.9082 | 0.6333 | 0.4472 | 0.9589 | 0.9501 | 0.6532 | 0.7722 | 0.7423 |
| 94 | 0.7743 | 0.8467 | 0.9082 | 0.6333 | 0.4491 | 0.9589 | 0.9501 | 0.6515 | 0.7728 | 0.7431 |
| 95 | 0.7690 | 0.8467 | 0.9082 | 0.6369 | 0.4530 | 0.9570 | 0.9501 | 0.6532 | 0.7731 | 0.7432 |
| 96 | 0.7795 | 0.8501 | 0.9082 | 0.6297 | 0.4568 | 0.9589 | 0.9535 | 0.6497 | 0.7746 | 0.7451 |
| 97 | 0.7795 | 0.8501 | 0.9082 | 0.6315 | 0.4549 | 0.9589 | 0.9535 | 0.6480 | 0.7744 | 0.7449 |
| 98 | 0.7707 | 0.8518 | 0.9102 | 0.6476 | 0.4472 | 0.9589 | 0.9518 | 0.6585 | 0.7760 | 0.7464 |
| 99 | 0.7725 | 0.8518 | 0.9102 | 0.6458 | 0.4511 | 0.9589 | 0.9535 | 0.6585 | 0.7767 | 0.7472 |
| 100 | 0.7725 | 0.8518 | 0.9121 | 0.6476 | 0.4472 | 0.9589 | 0.9518 | 0.6567 | 0.7762 | 0.7467 |
| 101 | 0.7760 | 0.8484 | 0.9160 | 0.6440 | 0.4587 | 0.9607 | 0.9535 | 0.6567 | 0.7780 | 0.7488 |
| 102 | 0.7743 | 0.8484 | 0.9160 | 0.6512 | 0.4549 | 0.9607 | 0.9535 | 0.6585 | 0.7785 | 0.7493 |
| 103 | 0.7848 | 0.8501 | 0.9199 | 0.6565 | 0.4549 | 0.9607 | 0.9553 | 0.6620 | 0.7819 | 0.7533 |
| 104 | 0.7848 | 0.8501 | 0.9180 | 0.6565 | 0.4549 | 0.9607 | 0.9553 | 0.6620 | 0.7816 | 0.7530 |
| 105 | 0.7813 | 0.8586 | 0.9219 | 0.6565 | 0.4549 | 0.9607 | 0.9570 | 0.6637 | 0.7832 | 0.7547 |
| 106 | 0.7813 | 0.8569 | 0.9258 | 0.6565 | 0.4530 | 0.9607 | 0.9570 | 0.6637 | 0.7832 | 0.7548 |
| 107 | 0.7778 | 0.8586 | 0.9258 | 0.6959 | 0.5067 | 0.9607 | 0.9570 | 0.6760 | 0.7958 | 0.7684 |
| 108 | 0.7919 | 0.8637 | 0.9258 | 0.6852 | 0.5086 | 0.9607 | 0.9570 | 0.6760 | 0.7972 | 0.7700 |
| 109 | 0.7989 | 0.8637 | 0.9258 | 0.6995 | 0.5086 | 0.9607 | 0.9587 | 0.6760 | 0.8001 | 0.7733 |
| 110 | 0.8025 | 0.8637 | 0.9238 | 0.6959 | 0.5125 | 0.9607 | 0.9587 | 0.6778 | 0.8006 | 0.7738 |
| 111 | 0.8007 | 0.8654 | 0.9258 | 0.6995 | 0.5144 | 0.9607 | 0.9587 | 0.6708 | 0.8006 | 0.7740 |
| 112 | 0.8007 | 0.8654 | 0.9199 | 0.7013 | 0.5144 | 0.9607 | 0.9570 | 0.6725 | 0.8001 | 0.7735 |
| 113 | 0.8007 | 0.8654 | 0.9199 | 0.7030 | 0.5182 | 0.9607 | 0.9570 | 0.6743 | 0.8010 | 0.7745 |
| 114 | 0.7989 | 0.8654 | 0.9199 | 0.7030 | 0.5182 | 0.9607 | 0.9570 | 0.6760 | 0.8010 | 0.7745 |
| 115 | 0.8007 | 0.8637 | 0.9199 | 0.7013 | 0.5278 | 0.9607 | 0.9570 | 0.6760 | 0.8019 | 0.7755 |
| 116 | 0.8007 | 0.8637 | 0.9238 | 0.6977 | 0.5278 | 0.9607 | 0.9587 | 0.6760 | 0.8022 | 0.7757 |
| 117 | 0.8148 | 0.8637 | 0.9180 | 0.6708 | 0.5566 | 0.9664 | 0.9604 | 0.6935 | 0.8065 | 0.7806 |
| 118 | 0.8183 | 0.8637 | 0.9180 | 0.6708 | 0.5547 | 0.9664 | 0.9604 | 0.6953 | 0.8069 | 0.7812 |
| 119 | 0.8236 | 0.8722 | 0.9160 | 0.6655 | 0.5451 | 0.9645 | 0.9587 | 0.6935 | 0.8060 | 0.7802 |
| 120 | 0.8219 | 0.8722 | 0.9180 | 0.6601 | 0.5451 | 0.9645 | 0.9604 | 0.7005 | 0.8065 | 0.7807 |
| 121 | 0.8166 | 0.8705 | 0.9160 | 0.6583 | 0.5509 | 0.9645 | 0.9604 | 0.7040 | 0.8062 | 0.7803 |
| 122 | 0.8307 | 0.8688 | 0.9219 | 0.6655 | 0.5605 | 0.9645 | 0.9604 | 0.7023 | 0.8103 | 0.7849 |
| 123 | 0.8272 | 0.8808 | 0.9297 | 0.6905 | 0.5547 | 0.9701 | 0.9621 | 0.7005 | 0.8155 | 0.7909 |
| 124 | 0.8307 | 0.8808 | 0.9336 | 0.6869 | 0.5547 | 0.9664 | 0.9621 | 0.7023 | 0.8157 | 0.7911 |
| 125 | 0.8448 | 0.8808 | 0.9355 | 0.6834 | 0.5451 | 0.9682 | 0.9621 | 0.7023 | 0.8164 | 0.7920 |
| 126 | 0.8377 | 0.8842 | 0.9395 | 0.6691 | 0.5643 | 0.9682 | 0.9604 | 0.7110 | 0.8177 | 0.7934 |
| 127 | 0.8377 | 0.8842 | 0.9395 | 0.6691 | 0.5681 | 0.9682 | 0.9604 | 0.7093 | 0.8180 | 0.7936 |
| 128 | 0.8360 | 0.8842 | 0.9395 | 0.6708 | 0.5643 | 0.9664 | 0.9604 | 0.7145 | 0.8180 | 0.7936 |
| 129 | 0.8377 | 0.8842 | 0.9395 | 0.6780 | 0.5720 | 0.9664 | 0.9604 | 0.7180 | 0.8204 | 0.7964 |
| 130 | 0.8377 | 0.8842 | 0.9395 | 0.6798 | 0.5681 | 0.9664 | 0.9604 | 0.7198 | 0.8204 | 0.7964 |
| 131 | 0.8413 | 0.8842 | 0.9414 | 0.6798 | 0.5701 | 0.9664 | 0.9621 | 0.7145 | 0.8209 | 0.7969 |
| 132 | 0.8360 | 0.8859 | 0.9375 | 0.6744 | 0.5547 | 0.9682 | 0.9621 | 0.7180 | 0.8182 | 0.7939 |
| 133 | 0.8377 | 0.8859 | 0.9395 | 0.6816 | 0.5566 | 0.9682 | 0.9621 | 0.7145 | 0.8193 | 0.7952 |
| 134 | 0.8413 | 0.8859 | 0.9395 | 0.6852 | 0.5585 | 0.9682 | 0.9621 | 0.7163 | 0.8207 | 0.7968 |
| 135 | 0.8377 | 0.8876 | 0.9414 | 0.6869 | 0.5585 | 0.9701 | 0.9621 | 0.7198 | 0.8216 | 0.7978 |
| 136 | 0.8448 | 0.8859 | 0.9414 | 0.6852 | 0.5547 | 0.9682 | 0.9621 | 0.7198 | 0.8213 | 0.7976 |
| 137 | 0.8448 | 0.8859 | 0.9414 | 0.6852 | 0.5547 | 0.9682 | 0.9621 | 0.7233 | 0.8218 | 0.7981 |
| 138 | 0.8430 | 0.8859 | 0.9414 | 0.6852 | 0.5547 | 0.9682 | 0.9621 | 0.7233 | 0.8216 | 0.7978 |
| 139 | 0.8395 | 0.8876 | 0.9434 | 0.7102 | 0.5873 | 0.9682 | 0.9621 | 0.7128 | 0.8272 | 0.8040 |
| 140 | 0.8395 | 0.8876 | 0.9434 | 0.7120 | 0.5873 | 0.9682 | 0.9621 | 0.7110 | 0.8272 | 0.8040 |
| 141 | 0.8395 | 0.8859 | 0.9434 | 0.7120 | 0.5873 | 0.9682 | 0.9621 | 0.7128 | 0.8272 | 0.8040 |
| 142 | 0.8430 | 0.8859 | 0.9414 | 0.7084 | 0.5854 | 0.9682 | 0.9621 | 0.7128 | 0.8268 | 0.8035 |
| 143 | 0.8448 | 0.8842 | 0.9434 | 0.7102 | 0.5816 | 0.9682 | 0.9621 | 0.7128 | 0.8268 | 0.8036 |
| 144 | 0.8466 | 0.8893 | 0.9453 | 0.7156 | 0.5873 | 0.9682 | 0.9621 | 0.7198 | 0.8301 | 0.8074 |
| 145 | 0.8466 | 0.8893 | 0.9492 | 0.7120 | 0.5893 | 0.9682 | 0.9621 | 0.7145 | 0.8297 | 0.8068 |
| 146 | 0.8466 | 0.8893 | 0.9492 | 0.7120 | 0.5893 | 0.9682 | 0.9621 | 0.7145 | 0.8297 | 0.8068 |
| 147 | 0.8430 | 0.8893 | 0.9492 | 0.7156 | 0.5893 | 0.9682 | 0.9621 | 0.7233 | 0.8308 | 0.8081 |
| 148 | 0.8430 | 0.8893 | 0.9492 | 0.7174 | 0.5873 | 0.9682 | 0.9621 | 0.7233 | 0.8308 | 0.8081 |
| 149 | 0.8430 | 0.8859 | 0.9512 | 0.7191 | 0.5912 | 0.9682 | 0.9621 | 0.7250 | 0.8315 | 0.8088 |
| 150 | 0.8430 | 0.8876 | 0.9512 | 0.7174 | 0.5873 | 0.9682 | 0.9621 | 0.7268 | 0.8313 | 0.8086 |
| 151 | 0.8430 | 0.8876 | 0.9531 | 0.7174 | 0.5873 | 0.9682 | 0.9621 | 0.7268 | 0.8315 | 0.8088 |
| 152 | 0.8430 | 0.8876 | 0.9531 | 0.7174 | 0.5873 | 0.9682 | 0.9621 | 0.7268 | 0.8315 | 0.8088 |
| 153 | 0.8307 | 0.8876 | 0.9512 | 0.7299 | 0.5969 | 0.9682 | 0.9604 | 0.7285 | 0.8324 | 0.8096 |
| 154 | 0.8272 | 0.8893 | 0.9512 | 0.7263 | 0.5950 | 0.9682 | 0.9604 | 0.7285 | 0.8315 | 0.8086 |
| 155 | 0.8325 | 0.8893 | 0.9551 | 0.7245 | 0.6008 | 0.9682 | 0.9604 | 0.7285 | 0.8331 | 0.8103 |
| 156 | 0.8307 | 0.8876 | 0.9531 | 0.7335 | 0.6104 | 0.9682 | 0.9604 | 0.7356 | 0.8356 | 0.8130 |
| 157 | 0.8289 | 0.8876 | 0.9551 | 0.7317 | 0.6084 | 0.9682 | 0.9604 | 0.7356 | 0.8351 | 0.8125 |
| 158 | 0.8325 | 0.8876 | 0.9531 | 0.7317 | 0.6104 | 0.9682 | 0.9604 | 0.7356 | 0.8356 | 0.8130 |
| 159 | 0.8289 | 0.8876 | 0.9512 | 0.7335 | 0.6104 | 0.9682 | 0.9604 | 0.7373 | 0.8353 | 0.8128 |
| 160 | 0.8289 | 0.8876 | 0.9512 | 0.7335 | 0.6046 | 0.9682 | 0.9604 | 0.7373 | 0.8346 | 0.8120 |
| 161 | 0.8307 | 0.8876 | 0.9512 | 0.7335 | 0.6046 | 0.9682 | 0.9604 | 0.7356 | 0.8346 | 0.8121 |
| 162 | 0.8307 | 0.8876 | 0.9531 | 0.7281 | 0.6200 | 0.9682 | 0.9604 | 0.7356 | 0.8360 | 0.8136 |
| 163 | 0.8307 | 0.8910 | 0.9531 | 0.7299 | 0.6219 | 0.9701 | 0.9604 | 0.7320 | 0.8367 | 0.8143 |
| 164 | 0.8307 | 0.8893 | 0.9551 | 0.7317 | 0.6219 | 0.9701 | 0.9604 | 0.7303 | 0.8367 | 0.8144 |
| 165 | 0.8289 | 0.8944 | 0.9570 | 0.7352 | 0.6084 | 0.9682 | 0.9621 | 0.7373 | 0.8371 | 0.8149 |
| 166 | 0.8289 | 0.8927 | 0.9570 | 0.7442 | 0.6084 | 0.9682 | 0.9621 | 0.7408 | 0.8385 | 0.8164 |
| 167 | 0.8289 | 0.8944 | 0.9570 | 0.7478 | 0.6084 | 0.9682 | 0.9621 | 0.7408 | 0.8392 | 0.8171 |
| 168 | 0.8272 | 0.8978 | 0.9570 | 0.7478 | 0.6065 | 0.9720 | 0.9604 | 0.7391 | 0.8392 | 0.8171 |
| 169 | 0.8254 | 0.8995 | 0.9570 | 0.7478 | 0.6142 | 0.9720 | 0.9621 | 0.7426 | 0.8407 | 0.8189 |
| 170 | 0.8236 | 0.8978 | 0.9551 | 0.7460 | 0.6161 | 0.9720 | 0.9604 | 0.7443 | 0.8401 | 0.8181 |
| 171 | 0.8342 | 0.8978 | 0.9570 | 0.7585 | 0.6180 | 0.9720 | 0.9621 | 0.7373 | 0.8428 | 0.8213 |
| 172 | 0.8377 | 0.8961 | 0.9570 | 0.7567 | 0.6180 | 0.9720 | 0.9621 | 0.7373 | 0.8428 | 0.8213 |
| 173 | 0.8377 | 0.8961 | 0.9570 | 0.7567 | 0.6200 | 0.9720 | 0.9621 | 0.7391 | 0.8432 | 0.8218 |
| 174 | 0.8307 | 0.8961 | 0.9570 | 0.7478 | 0.6161 | 0.9720 | 0.9621 | 0.7426 | 0.8412 | 0.8194 |
| 175 | 0.8307 | 0.8944 | 0.9551 | 0.7496 | 0.6180 | 0.9720 | 0.9621 | 0.7391 | 0.8407 | 0.8189 |
| 176 | 0.8289 | 0.8944 | 0.9570 | 0.7496 | 0.6180 | 0.9720 | 0.9621 | 0.7426 | 0.8412 | 0.8194 |
| 177 | 0.8289 | 0.8961 | 0.9551 | 0.7496 | 0.6180 | 0.9720 | 0.9621 | 0.7426 | 0.8412 | 0.8194 |
| 178 | 0.8289 | 0.8961 | 0.9551 | 0.7460 | 0.6219 | 0.9720 | 0.9621 | 0.7443 | 0.8414 | 0.8196 |
| 179 | 0.8289 | 0.8961 | 0.9551 | 0.7496 | 0.6219 | 0.9720 | 0.9621 | 0.7443 | 0.8419 | 0.8202 |
| 180 | 0.8289 | 0.8961 | 0.9551 | 0.7478 | 0.6219 | 0.9720 | 0.9621 | 0.7443 | 0.8416 | 0.8199 |
| 181 | 0.8289 | 0.8961 | 0.9551 | 0.7496 | 0.6219 | 0.9720 | 0.9621 | 0.7443 | 0.8419 | 0.8202 |
| 182 | 0.8307 | 0.8944 | 0.9551 | 0.7478 | 0.6219 | 0.9720 | 0.9621 | 0.7443 | 0.8416 | 0.8199 |
| 183 | 0.8325 | 0.8910 | 0.9590 | 0.7746 | 0.6334 | 0.9738 | 0.9587 | 0.7601 | 0.8484 | 0.8275 |
| 184 | 0.8325 | 0.8927 | 0.9590 | 0.7800 | 0.6430 | 0.9720 | 0.9604 | 0.7583 | 0.8502 | 0.8296 |
| 185 | 0.8342 | 0.8961 | 0.9590 | 0.7782 | 0.6430 | 0.9720 | 0.9604 | 0.7583 | 0.8507 | 0.8301 |
| 186 | 0.8325 | 0.8944 | 0.9590 | 0.7764 | 0.6372 | 0.9720 | 0.9604 | 0.7601 | 0.8495 | 0.8288 |
| 187 | 0.8325 | 0.8944 | 0.9590 | 0.7710 | 0.6430 | 0.9701 | 0.9604 | 0.7583 | 0.8491 | 0.8283 |
| 188 | 0.8307 | 0.8944 | 0.9590 | 0.7728 | 0.6430 | 0.9701 | 0.9604 | 0.7583 | 0.8491 | 0.8283 |
| 189 | 0.8342 | 0.8944 | 0.9590 | 0.7728 | 0.6468 | 0.9701 | 0.9604 | 0.7566 | 0.8498 | 0.8290 |
| 190 | 0.8325 | 0.8944 | 0.9629 | 0.7818 | 0.6468 | 0.9701 | 0.9587 | 0.7566 | 0.8509 | 0.8303 |
| 191 | 0.8289 | 0.8995 | 0.9648 | 0.7764 | 0.6430 | 0.9682 | 0.9587 | 0.7461 | 0.8486 | 0.8278 |
| 192 | 0.8307 | 0.8995 | 0.9648 | 0.7764 | 0.6449 | 0.9682 | 0.9587 | 0.7478 | 0.8493 | 0.8285 |
| 193 | 0.8307 | 0.8995 | 0.9648 | 0.7800 | 0.6449 | 0.9682 | 0.9587 | 0.7496 | 0.8500 | 0.8293 |
| 194 | 0.8307 | 0.9012 | 0.9648 | 0.7800 | 0.6449 | 0.9682 | 0.9587 | 0.7513 | 0.8504 | 0.8298 |
| 195 | 0.8307 | 0.8978 | 0.9648 | 0.7800 | 0.6449 | 0.9682 | 0.9587 | 0.7496 | 0.8498 | 0.8290 |
| 196 | 0.8413 | 0.9029 | 0.9629 | 0.7674 | 0.6583 | 0.9682 | 0.9587 | 0.7601 | 0.8529 | 0.8326 |
| 197 | 0.8395 | 0.9029 | 0.9648 | 0.7674 | 0.6564 | 0.9682 | 0.9604 | 0.7583 | 0.8527 | 0.8324 |
| 198 | 0.8395 | 0.9029 | 0.9648 | 0.7710 | 0.6583 | 0.9682 | 0.9604 | 0.7601 | 0.8536 | 0.8334 |
| 199 | 0.8519 | 0.9029 | 0.9648 | 0.7728 | 0.6564 | 0.9664 | 0.9604 | 0.7566 | 0.8545 | 0.8345 |
| 200 | 0.8536 | 0.9012 | 0.9668 | 0.7728 | 0.6564 | 0.9682 | 0.9604 | 0.7583 | 0.8552 | 0.8353 |
| 201 | 0.8501 | 0.8995 | 0.9648 | 0.7818 | 0.6545 | 0.9682 | 0.9604 | 0.7583 | 0.8552 | 0.8353 |
| 202 | 0.8448 | 0.9012 | 0.9668 | 0.7835 | 0.6545 | 0.9682 | 0.9604 | 0.7601 | 0.8554 | 0.8355 |
| 203 | 0.8466 | 0.8995 | 0.9668 | 0.7853 | 0.6564 | 0.9664 | 0.9604 | 0.7583 | 0.8554 | 0.8355 |
| 204 | 0.8483 | 0.8995 | 0.9668 | 0.7835 | 0.6583 | 0.9664 | 0.9604 | 0.7583 | 0.8556 | 0.8358 |
| 205 | 0.8554 | 0.9012 | 0.9648 | 0.7800 | 0.6583 | 0.9664 | 0.9656 | 0.7863 | 0.8604 | 0.8411 |
| 206 | 0.8554 | 0.9012 | 0.9648 | 0.7818 | 0.6583 | 0.9664 | 0.9656 | 0.7863 | 0.8606 | 0.8414 |
| 207 | 0.8554 | 0.9029 | 0.9629 | 0.7818 | 0.6583 | 0.9664 | 0.9656 | 0.7846 | 0.8604 | 0.8411 |
| 208 | 0.8501 | 0.9029 | 0.9648 | 0.7818 | 0.6449 | 0.9682 | 0.9656 | 0.7846 | 0.8586 | 0.8391 |
| 209 | 0.8519 | 0.8995 | 0.9648 | 0.7853 | 0.6468 | 0.9664 | 0.9656 | 0.7846 | 0.8588 | 0.8393 |
| 210 | 0.8554 | 0.9012 | 0.9648 | 0.7853 | 0.6488 | 0.9664 | 0.9656 | 0.7828 | 0.8595 | 0.8401 |
| 211 | 0.8501 | 0.9012 | 0.9668 | 0.7835 | 0.6507 | 0.9664 | 0.9639 | 0.7793 | 0.8583 | 0.8388 |
| 212 | 0.8519 | 0.9029 | 0.9688 | 0.7835 | 0.6468 | 0.9664 | 0.9639 | 0.7793 | 0.8586 | 0.8391 |
| 213 | 0.8501 | 0.9012 | 0.9688 | 0.7835 | 0.6449 | 0.9664 | 0.9639 | 0.7863 | 0.8588 | 0.8393 |
| 214 | 0.8536 | 0.9012 | 0.9688 | 0.7835 | 0.6468 | 0.9664 | 0.9639 | 0.7863 | 0.8595 | 0.8401 |
| 215 | 0.8536 | 0.9012 | 0.9688 | 0.7853 | 0.6449 | 0.9664 | 0.9639 | 0.7846 | 0.8592 | 0.8398 |
| 216 | 0.8554 | 0.9029 | 0.9688 | 0.7835 | 0.6488 | 0.9664 | 0.9656 | 0.7916 | 0.8610 | 0.8419 |
| 217 | 0.8536 | 0.9029 | 0.9688 | 0.7818 | 0.6507 | 0.9664 | 0.9656 | 0.7898 | 0.8606 | 0.8413 |
| 218 | 0.8571 | 0.9029 | 0.9688 | 0.7835 | 0.6488 | 0.9664 | 0.9656 | 0.7898 | 0.8610 | 0.8419 |
| 219 | 0.8607 | 0.9046 | 0.9668 | 0.7871 | 0.6526 | 0.9720 | 0.9656 | 0.7863 | 0.8626 | 0.8437 |
| 220 | 0.8607 | 0.9029 | 0.9668 | 0.7889 | 0.6507 | 0.9720 | 0.9656 | 0.7863 | 0.8624 | 0.8435 |
| 221 | 0.8607 | 0.9029 | 0.9668 | 0.7889 | 0.6507 | 0.9720 | 0.9639 | 0.7863 | 0.8622 | 0.8432 |
| 222 | 0.8589 | 0.9046 | 0.9668 | 0.7907 | 0.6507 | 0.9720 | 0.9639 | 0.7863 | 0.8624 | 0.8434 |
| 223 | 0.8571 | 0.9046 | 0.9668 | 0.7943 | 0.6526 | 0.9720 | 0.9639 | 0.7881 | 0.8631 | 0.8442 |
| 224 | 0.8571 | 0.9046 | 0.9668 | 0.8014 | 0.6526 | 0.9720 | 0.9639 | 0.7846 | 0.8635 | 0.8447 |
| 225 | 0.8571 | 0.9046 | 0.9668 | 0.7996 | 0.6545 | 0.9720 | 0.9639 | 0.7828 | 0.8633 | 0.8445 |
| 226 | 0.8571 | 0.9046 | 0.9668 | 0.7996 | 0.6545 | 0.9720 | 0.9639 | 0.7846 | 0.8635 | 0.8447 |
| 227 | 0.8571 | 0.9029 | 0.9668 | 0.7996 | 0.6545 | 0.9720 | 0.9639 | 0.7846 | 0.8633 | 0.8445 |
| 228 | 0.8554 | 0.9063 | 0.9688 | 0.8032 | 0.6526 | 0.9720 | 0.9639 | 0.7916 | 0.8649 | 0.8462 |
| 229 | 0.8536 | 0.9063 | 0.9688 | 0.8014 | 0.6526 | 0.9720 | 0.9639 | 0.7916 | 0.8644 | 0.8457 |
| 230 | 0.8536 | 0.9063 | 0.9688 | 0.8050 | 0.6488 | 0.9720 | 0.9639 | 0.7968 | 0.8651 | 0.8465 |
| 231 | 0.8519 | 0.9063 | 0.9688 | 0.8086 | 0.6526 | 0.9720 | 0.9639 | 0.7951 | 0.8656 | 0.8470 |
| 232 | 0.8519 | 0.9063 | 0.9688 | 0.8068 | 0.6545 | 0.9720 | 0.9639 | 0.8004 | 0.8662 | 0.8477 |
| 233 | 0.8519 | 0.9063 | 0.9688 | 0.8068 | 0.6526 | 0.9720 | 0.9639 | 0.7916 | 0.8649 | 0.8462 |
| 234 | 0.8554 | 0.9063 | 0.9688 | 0.7996 | 0.6430 | 0.9720 | 0.9639 | 0.7881 | 0.8628 | 0.8440 |
| 235 | 0.8607 | 0.9080 | 0.9688 | 0.8014 | 0.6411 | 0.9720 | 0.9639 | 0.7951 | 0.8647 | 0.8460 |
| 236 | 0.8607 | 0.9080 | 0.9688 | 0.8050 | 0.6430 | 0.9720 | 0.9639 | 0.7968 | 0.8656 | 0.8470 |
| 237 | 0.8642 | 0.9097 | 0.9688 | 0.8140 | 0.6488 | 0.9720 | 0.9639 | 0.8021 | 0.8687 | 0.8506 |
| 238 | 0.8677 | 0.9080 | 0.9648 | 0.8157 | 0.6507 | 0.9720 | 0.9656 | 0.8179 | 0.8712 | 0.8534 |
| 239 | 0.8695 | 0.9080 | 0.9648 | 0.8122 | 0.6526 | 0.9720 | 0.9656 | 0.8179 | 0.8712 | 0.8534 |
| 240 | 0.8748 | 0.9097 | 0.9648 | 0.8140 | 0.6603 | 0.9720 | 0.9656 | 0.8249 | 0.8741 | 0.8567 |
| 241 | 0.8730 | 0.9097 | 0.9648 | 0.8157 | 0.6603 | 0.9720 | 0.9656 | 0.8231 | 0.8739 | 0.8565 |
| 242 | 0.8607 | 0.9097 | 0.9668 | 0.8211 | 0.6660 | 0.9720 | 0.9673 | 0.8214 | 0.8739 | 0.8564 |
| 243 | 0.8624 | 0.9097 | 0.9668 | 0.8211 | 0.6660 | 0.9720 | 0.9673 | 0.8231 | 0.8744 | 0.8569 |
| 244 | 0.8571 | 0.9097 | 0.9688 | 0.8175 | 0.6660 | 0.9720 | 0.9673 | 0.8249 | 0.8737 | 0.8562 |
| 245 | 0.8607 | 0.9097 | 0.9688 | 0.8175 | 0.6660 | 0.9720 | 0.9673 | 0.8249 | 0.8741 | 0.8567 |
| 246 | 0.8554 | 0.9097 | 0.9707 | 0.8175 | 0.6679 | 0.9720 | 0.9673 | 0.8249 | 0.8739 | 0.8564 |
| 247 | 0.8554 | 0.9097 | 0.9707 | 0.8175 | 0.6660 | 0.9720 | 0.9656 | 0.8249 | 0.8734 | 0.8559 |
| 248 | 0.8554 | 0.9097 | 0.9707 | 0.8193 | 0.6679 | 0.9720 | 0.9656 | 0.8266 | 0.8741 | 0.8567 |
| 249 | 0.8554 | 0.9097 | 0.9707 | 0.8211 | 0.6679 | 0.9720 | 0.9656 | 0.8266 | 0.8744 | 0.8569 |
| 250 | 0.8589 | 0.9097 | 0.9707 | 0.8193 | 0.6660 | 0.9720 | 0.9656 | 0.8249 | 0.8741 | 0.8567 |
| 251 | 0.8695 | 0.9097 | 0.9707 | 0.8193 | 0.6718 | 0.9720 | 0.9656 | 0.8214 | 0.8757 | 0.8585 |
| 252 | 0.8695 | 0.9114 | 0.9707 | 0.8211 | 0.6737 | 0.9720 | 0.9656 | 0.8231 | 0.8766 | 0.8595 |
| 253 | 0.8660 | 0.9131 | 0.9707 | 0.8175 | 0.6814 | 0.9720 | 0.9656 | 0.8161 | 0.8759 | 0.8587 |
| 254 | 0.8695 | 0.9182 | 0.9707 | 0.8193 | 0.6852 | 0.9720 | 0.9656 | 0.8161 | 0.8777 | 0.8607 |
| 255 | 0.8695 | 0.9148 | 0.9707 | 0.8175 | 0.6891 | 0.9720 | 0.9656 | 0.8179 | 0.8777 | 0.8607 |
| 256 | 0.8713 | 0.9182 | 0.9707 | 0.8193 | 0.6891 | 0.9720 | 0.9656 | 0.8179 | 0.8786 | 0.8618 |
| 257 | 0.8677 | 0.9182 | 0.9707 | 0.8193 | 0.6891 | 0.9720 | 0.9656 | 0.8179 | 0.8782 | 0.8612 |
| 258 | 0.8677 | 0.9182 | 0.9707 | 0.8175 | 0.6871 | 0.9720 | 0.9656 | 0.8126 | 0.8771 | 0.8600 |
| 259 | 0.8624 | 0.9182 | 0.9688 | 0.8229 | 0.6871 | 0.9720 | 0.9656 | 0.8126 | 0.8768 | 0.8597 |
| 260 | 0.8607 | 0.9182 | 0.9688 | 0.8247 | 0.6891 | 0.9720 | 0.9656 | 0.8144 | 0.8773 | 0.8602 |
| 261 | 0.8642 | 0.9182 | 0.9707 | 0.8211 | 0.6891 | 0.9720 | 0.9656 | 0.8196 | 0.8782 | 0.8612 |
| 262 | 0.8660 | 0.9199 | 0.9707 | 0.8229 | 0.6929 | 0.9720 | 0.9656 | 0.8196 | 0.8793 | 0.8625 |
| 263 | 0.8677 | 0.9199 | 0.9707 | 0.8211 | 0.6929 | 0.9720 | 0.9656 | 0.8161 | 0.8789 | 0.8620 |
| 264 | 0.8695 | 0.9148 | 0.9746 | 0.8229 | 0.6871 | 0.9720 | 0.9673 | 0.8161 | 0.8786 | 0.8618 |
| 265 | 0.8677 | 0.9182 | 0.9746 | 0.8265 | 0.6852 | 0.9720 | 0.9673 | 0.8196 | 0.8795 | 0.8628 |
| 266 | 0.8695 | 0.9114 | 0.9746 | 0.8301 | 0.6852 | 0.9738 | 0.9673 | 0.8179 | 0.8793 | 0.8626 |
| 267 | 0.8677 | 0.9097 | 0.9746 | 0.8247 | 0.6871 | 0.9738 | 0.9673 | 0.8179 | 0.8784 | 0.8615 |
| 268 | 0.8677 | 0.9080 | 0.9746 | 0.8247 | 0.6871 | 0.9738 | 0.9673 | 0.8179 | 0.8782 | 0.8613 |
| 269 | 0.8713 | 0.9080 | 0.9746 | 0.8265 | 0.6891 | 0.9738 | 0.9673 | 0.8196 | 0.8793 | 0.8626 |
| 270 | 0.8748 | 0.9097 | 0.9746 | 0.8318 | 0.6871 | 0.9738 | 0.9690 | 0.8196 | 0.8807 | 0.8641 |
| 271 | 0.8748 | 0.9097 | 0.9746 | 0.8336 | 0.6852 | 0.9738 | 0.9690 | 0.8214 | 0.8809 | 0.8644 |
| 272 | 0.8748 | 0.9097 | 0.9746 | 0.8318 | 0.6852 | 0.9738 | 0.9690 | 0.8196 | 0.8804 | 0.8639 |
| 273 | 0.8748 | 0.9114 | 0.9746 | 0.8318 | 0.6833 | 0.9738 | 0.9690 | 0.8214 | 0.8807 | 0.8641 |
| 274 | 0.8783 | 0.9080 | 0.9746 | 0.8318 | 0.6814 | 0.9738 | 0.9690 | 0.8196 | 0.8802 | 0.8636 |
| 275 | 0.8783 | 0.9080 | 0.9746 | 0.8318 | 0.6814 | 0.9738 | 0.9690 | 0.8214 | 0.8804 | 0.8639 |
| 276 | 0.8801 | 0.9080 | 0.9746 | 0.8336 | 0.6814 | 0.9738 | 0.9690 | 0.8196 | 0.8807 | 0.8641 |
| 277 | 0.8801 | 0.9080 | 0.9746 | 0.8336 | 0.6814 | 0.9738 | 0.9690 | 0.8231 | 0.8811 | 0.8647 |
| 278 | 0.8801 | 0.9080 | 0.9746 | 0.8372 | 0.6795 | 0.9738 | 0.9690 | 0.8231 | 0.8813 | 0.8649 |
| 279 | 0.8783 | 0.9080 | 0.9746 | 0.8372 | 0.6795 | 0.9738 | 0.9673 | 0.8214 | 0.8807 | 0.8641 |
| 280 | 0.8765 | 0.9080 | 0.9746 | 0.8390 | 0.6814 | 0.9738 | 0.9673 | 0.8266 | 0.8816 | 0.8652 |
| 281 | 0.8730 | 0.9063 | 0.9746 | 0.8479 | 0.6910 | 0.9738 | 0.9673 | 0.8266 | 0.8831 | 0.8670 |
| 282 | 0.8748 | 0.9063 | 0.9746 | 0.8444 | 0.6871 | 0.9738 | 0.9673 | 0.8266 | 0.8825 | 0.8662 |
| 283 | 0.8660 | 0.9063 | 0.9746 | 0.8408 | 0.6891 | 0.9757 | 0.9673 | 0.8266 | 0.8813 | 0.8649 |
| 284 | 0.8677 | 0.9063 | 0.9746 | 0.8408 | 0.6891 | 0.9757 | 0.9673 | 0.8266 | 0.8816 | 0.8651 |
| 285 | 0.8642 | 0.9097 | 0.9746 | 0.8229 | 0.7006 | 0.9757 | 0.9673 | 0.8284 | 0.8809 | 0.8643 |
| 286 | 0.8642 | 0.9097 | 0.9746 | 0.8211 | 0.7006 | 0.9757 | 0.9673 | 0.8284 | 0.8807 | 0.8640 |
| 287 | 0.8642 | 0.9097 | 0.9746 | 0.8211 | 0.7006 | 0.9757 | 0.9673 | 0.8284 | 0.8807 | 0.8640 |
| 288 | 0.8642 | 0.9114 | 0.9746 | 0.8301 | 0.6967 | 0.9757 | 0.9673 | 0.8319 | 0.8820 | 0.8656 |
| 289 | 0.8660 | 0.9131 | 0.9746 | 0.8301 | 0.6967 | 0.9757 | 0.9673 | 0.8284 | 0.8820 | 0.8656 |
| 290 | 0.8695 | 0.9131 | 0.9746 | 0.8336 | 0.7006 | 0.9757 | 0.9673 | 0.8301 | 0.8836 | 0.8674 |
| 291 | 0.8695 | 0.9131 | 0.9746 | 0.8336 | 0.7006 | 0.9757 | 0.9673 | 0.8301 | 0.8836 | 0.8674 |
| 292 | 0.8695 | 0.9165 | 0.9746 | 0.8336 | 0.7006 | 0.9757 | 0.9673 | 0.8319 | 0.8843 | 0.8682 |
| 293 | 0.8695 | 0.9148 | 0.9746 | 0.8336 | 0.7006 | 0.9757 | 0.9673 | 0.8319 | 0.8841 | 0.8679 |
| 294 | 0.8677 | 0.9148 | 0.9746 | 0.8336 | 0.7025 | 0.9757 | 0.9673 | 0.8319 | 0.8841 | 0.8679 |
| 295 | 0.8677 | 0.9148 | 0.9746 | 0.8390 | 0.6967 | 0.9757 | 0.9673 | 0.8301 | 0.8838 | 0.8676 |
| 296 | 0.8642 | 0.9131 | 0.9746 | 0.8354 | 0.6967 | 0.9757 | 0.9673 | 0.8301 | 0.8827 | 0.8663 |
| 297 | 0.8642 | 0.9131 | 0.9746 | 0.8336 | 0.6967 | 0.9757 | 0.9673 | 0.8284 | 0.8822 | 0.8658 |
| 298 | 0.8660 | 0.9131 | 0.9746 | 0.8336 | 0.6967 | 0.9757 | 0.9673 | 0.8301 | 0.8827 | 0.8664 |
| 299 | 0.8642 | 0.9131 | 0.9746 | 0.8336 | 0.6948 | 0.9757 | 0.9690 | 0.8319 | 0.8827 | 0.8663 |
| 300 | 0.8624 | 0.9114 | 0.9766 | 0.8515 | 0.6929 | 0.9757 | 0.9690 | 0.8336 | 0.8847 | 0.8687 |
| 301 | 0.8660 | 0.9114 | 0.9766 | 0.8533 | 0.7006 | 0.9738 | 0.9690 | 0.8371 | 0.8865 | 0.8708 |
| 302 | 0.8642 | 0.9131 | 0.9746 | 0.8569 | 0.6948 | 0.9757 | 0.9690 | 0.8371 | 0.8863 | 0.8705 |
| 303 | 0.8642 | 0.9131 | 0.9746 | 0.8569 | 0.6948 | 0.9757 | 0.9690 | 0.8371 | 0.8863 | 0.8705 |
| 304 | 0.8660 | 0.9131 | 0.9746 | 0.8640 | 0.7006 | 0.9776 | 0.9690 | 0.8354 | 0.8881 | 0.8726 |
| 305 | 0.8660 | 0.9131 | 0.9746 | 0.8640 | 0.7006 | 0.9776 | 0.9690 | 0.8389 | 0.8886 | 0.8731 |
| 306 | 0.8660 | 0.9131 | 0.9746 | 0.8640 | 0.6987 | 0.9776 | 0.9690 | 0.8389 | 0.8883 | 0.8728 |
| 307 | 0.8660 | 0.9131 | 0.9746 | 0.8640 | 0.6987 | 0.9776 | 0.9690 | 0.8389 | 0.8883 | 0.8728 |
| 308 | 0.8660 | 0.9131 | 0.9746 | 0.8640 | 0.6987 | 0.9776 | 0.9690 | 0.8389 | 0.8883 | 0.8728 |
| 309 | 0.8660 | 0.9131 | 0.9746 | 0.8623 | 0.6929 | 0.9776 | 0.9690 | 0.8389 | 0.8874 | 0.8718 |
| 310 | 0.8660 | 0.9131 | 0.9746 | 0.8623 | 0.6967 | 0.9776 | 0.9690 | 0.8371 | 0.8877 | 0.8721 |
| 311 | 0.8642 | 0.9131 | 0.9746 | 0.8623 | 0.6948 | 0.9776 | 0.9690 | 0.8371 | 0.8872 | 0.8716 |
| 312 | 0.8642 | 0.9131 | 0.9746 | 0.8605 | 0.6987 | 0.9776 | 0.9690 | 0.8371 | 0.8874 | 0.8718 |
| 313 | 0.8642 | 0.9131 | 0.9746 | 0.8623 | 0.6987 | 0.9776 | 0.9690 | 0.8389 | 0.8879 | 0.8723 |
| 314 | 0.8642 | 0.9131 | 0.9746 | 0.8623 | 0.7006 | 0.9776 | 0.9690 | 0.8371 | 0.8879 | 0.8723 |
| 315 | 0.8642 | 0.9131 | 0.9746 | 0.8623 | 0.6987 | 0.9776 | 0.9690 | 0.8389 | 0.8879 | 0.8723 |
| 316 | 0.8677 | 0.9148 | 0.9746 | 0.8605 | 0.6987 | 0.9776 | 0.9690 | 0.8389 | 0.8883 | 0.8728 |
| 317 | 0.8677 | 0.9165 | 0.9746 | 0.8605 | 0.6987 | 0.9776 | 0.9690 | 0.8389 | 0.8886 | 0.8731 |
| 318 | 0.8642 | 0.9182 | 0.9766 | 0.8569 | 0.7025 | 0.9757 | 0.9690 | 0.8441 | 0.8890 | 0.8735 |
| 319 | 0.8660 | 0.9199 | 0.9766 | 0.8533 | 0.7025 | 0.9757 | 0.9690 | 0.8459 | 0.8892 | 0.8738 |
| 320 | 0.8624 | 0.9199 | 0.9766 | 0.8533 | 0.6987 | 0.9776 | 0.9690 | 0.8459 | 0.8886 | 0.8730 |
| 321 | 0.8624 | 0.9199 | 0.9766 | 0.8551 | 0.6987 | 0.9776 | 0.9690 | 0.8459 | 0.8888 | 0.8733 |
| 322 | 0.8624 | 0.9199 | 0.9766 | 0.8551 | 0.6967 | 0.9776 | 0.9690 | 0.8441 | 0.8883 | 0.8728 |
| 323 | 0.8660 | 0.9199 | 0.9766 | 0.8551 | 0.7006 | 0.9757 | 0.9690 | 0.8459 | 0.8892 | 0.8738 |
| 324 | 0.8660 | 0.9199 | 0.9766 | 0.8533 | 0.7025 | 0.9757 | 0.9690 | 0.8476 | 0.8895 | 0.8740 |
| 325 | 0.8660 | 0.9199 | 0.9785 | 0.8551 | 0.7025 | 0.9757 | 0.9690 | 0.8476 | 0.8899 | 0.8746 |
| 326 | 0.8642 | 0.9199 | 0.9785 | 0.8551 | 0.7025 | 0.9757 | 0.9690 | 0.8476 | 0.8897 | 0.8743 |
| 327 | 0.8677 | 0.9199 | 0.9785 | 0.8551 | 0.7025 | 0.9776 | 0.9690 | 0.8476 | 0.8904 | 0.8751 |
| 328 | 0.8642 | 0.9182 | 0.9766 | 0.8551 | 0.7083 | 0.9794 | 0.9690 | 0.8476 | 0.8904 | 0.8751 |
| 329 | 0.8660 | 0.9165 | 0.9766 | 0.8533 | 0.7063 | 0.9794 | 0.9690 | 0.8476 | 0.8899 | 0.8746 |
| 330 | 0.8642 | 0.9165 | 0.9766 | 0.8533 | 0.7063 | 0.9794 | 0.9690 | 0.8476 | 0.8897 | 0.8743 |
| 331 | 0.8660 | 0.9165 | 0.9766 | 0.8533 | 0.7063 | 0.9794 | 0.9690 | 0.8476 | 0.8899 | 0.8746 |
| 332 | 0.8607 | 0.9182 | 0.9766 | 0.8533 | 0.7102 | 0.9794 | 0.9690 | 0.8476 | 0.8899 | 0.8745 |
| 333 | 0.8607 | 0.9131 | 0.9766 | 0.8533 | 0.7063 | 0.9794 | 0.9690 | 0.8494 | 0.8890 | 0.8735 |
| 334 | 0.8624 | 0.9165 | 0.9766 | 0.8497 | 0.7102 | 0.9776 | 0.9690 | 0.8511 | 0.8897 | 0.8743 |
| 335 | 0.8624 | 0.9182 | 0.9766 | 0.8551 | 0.7102 | 0.9776 | 0.9690 | 0.8529 | 0.8908 | 0.8756 |
| 336 | 0.8607 | 0.9182 | 0.9785 | 0.8426 | 0.7140 | 0.9794 | 0.9673 | 0.8511 | 0.8895 | 0.8740 |
| 337 | 0.8624 | 0.9199 | 0.9785 | 0.8426 | 0.7121 | 0.9794 | 0.9673 | 0.8546 | 0.8901 | 0.8747 |
| 338 | 0.8624 | 0.9199 | 0.9785 | 0.8426 | 0.7121 | 0.9794 | 0.9673 | 0.8564 | 0.8904 | 0.8750 |
| 339 | 0.8624 | 0.9199 | 0.9785 | 0.8444 | 0.7121 | 0.9794 | 0.9673 | 0.8564 | 0.8906 | 0.8753 |
| 340 | 0.8607 | 0.9250 | 0.9785 | 0.8426 | 0.7140 | 0.9794 | 0.9656 | 0.8546 | 0.8906 | 0.8753 |
| 341 | 0.8589 | 0.9250 | 0.9785 | 0.8408 | 0.7140 | 0.9794 | 0.9656 | 0.8564 | 0.8904 | 0.8750 |
| 342 | 0.8589 | 0.9250 | 0.9785 | 0.8426 | 0.7121 | 0.9794 | 0.9673 | 0.8546 | 0.8904 | 0.8750 |
| 343 | 0.8607 | 0.9233 | 0.9785 | 0.8444 | 0.7121 | 0.9794 | 0.9673 | 0.8546 | 0.8906 | 0.8753 |
| 344 | 0.8571 | 0.9233 | 0.9766 | 0.8408 | 0.7102 | 0.9794 | 0.9656 | 0.8564 | 0.8892 | 0.8737 |
| 345 | 0.8624 | 0.9233 | 0.9766 | 0.8426 | 0.7121 | 0.9794 | 0.9656 | 0.8564 | 0.8904 | 0.8750 |
| 346 | 0.8642 | 0.9233 | 0.9766 | 0.8426 | 0.7102 | 0.9794 | 0.9656 | 0.8564 | 0.8904 | 0.8750 |
| 347 | 0.8607 | 0.9216 | 0.9766 | 0.8390 | 0.7121 | 0.9794 | 0.9656 | 0.8564 | 0.8895 | 0.8740 |
| 348 | 0.8660 | 0.9216 | 0.9766 | 0.8408 | 0.7140 | 0.9794 | 0.9673 | 0.8546 | 0.8906 | 0.8753 |
| 349 | 0.8571 | 0.9216 | 0.9766 | 0.8426 | 0.7140 | 0.9794 | 0.9673 | 0.8564 | 0.8899 | 0.8745 |
| 350 | 0.8607 | 0.9216 | 0.9766 | 0.8444 | 0.7140 | 0.9794 | 0.9690 | 0.8564 | 0.8908 | 0.8755 |
| 351 | 0.8624 | 0.9216 | 0.9766 | 0.8390 | 0.7140 | 0.9794 | 0.9656 | 0.8564 | 0.8899 | 0.8745 |
| 352 | 0.8660 | 0.9216 | 0.9746 | 0.8444 | 0.7121 | 0.9794 | 0.9673 | 0.8511 | 0.8901 | 0.8748 |
| 353 | 0.8642 | 0.9216 | 0.9746 | 0.8462 | 0.7159 | 0.9794 | 0.9673 | 0.8529 | 0.8908 | 0.8755 |
| 354 | 0.8624 | 0.9233 | 0.9766 | 0.8426 | 0.7140 | 0.9794 | 0.9673 | 0.8546 | 0.8906 | 0.8753 |
| 355 | 0.8607 | 0.9233 | 0.9766 | 0.8408 | 0.7121 | 0.9794 | 0.9673 | 0.8511 | 0.8895 | 0.8740 |
| 356 | 0.8607 | 0.9233 | 0.9766 | 0.8408 | 0.7063 | 0.9794 | 0.9673 | 0.8511 | 0.8888 | 0.8732 |
| 357 | 0.8607 | 0.9216 | 0.9766 | 0.8426 | 0.7083 | 0.9794 | 0.9673 | 0.8511 | 0.8890 | 0.8735 |
| 358 | 0.8607 | 0.9199 | 0.9766 | 0.8390 | 0.7102 | 0.9794 | 0.9673 | 0.8511 | 0.8886 | 0.8729 |
| 359 | 0.8554 | 0.9216 | 0.9785 | 0.8444 | 0.7140 | 0.9794 | 0.9673 | 0.8529 | 0.8897 | 0.8742 |
| 360 | 0.8554 | 0.9216 | 0.9785 | 0.8462 | 0.7159 | 0.9794 | 0.9673 | 0.8529 | 0.8901 | 0.8747 |
| 361 | 0.8448 | 0.9216 | 0.9785 | 0.8479 | 0.7198 | 0.9776 | 0.9673 | 0.8511 | 0.8890 | 0.8734 |
| 362 | 0.8519 | 0.9216 | 0.9785 | 0.8497 | 0.7159 | 0.9794 | 0.9690 | 0.8511 | 0.8901 | 0.8748 |
| 363 | 0.8501 | 0.9216 | 0.9785 | 0.8533 | 0.7159 | 0.9794 | 0.9690 | 0.8546 | 0.8908 | 0.8755 |
| 364 | 0.8466 | 0.9216 | 0.9785 | 0.8533 | 0.7217 | 0.9794 | 0.9690 | 0.8494 | 0.8904 | 0.8750 |
| 365 | 0.8571 | 0.9216 | 0.9785 | 0.8426 | 0.7198 | 0.9776 | 0.9690 | 0.8511 | 0.8901 | 0.8748 |
| 366 | 0.8554 | 0.9233 | 0.9785 | 0.8426 | 0.7198 | 0.9776 | 0.9690 | 0.8494 | 0.8899 | 0.8745 |
| 367 | 0.8554 | 0.9233 | 0.9785 | 0.8426 | 0.7179 | 0.9776 | 0.9690 | 0.8494 | 0.8897 | 0.8742 |
| 368 | 0.8554 | 0.9233 | 0.9785 | 0.8426 | 0.7198 | 0.9776 | 0.9707 | 0.8511 | 0.8904 | 0.8750 |
| 369 | 0.8554 | 0.9233 | 0.9785 | 0.8408 | 0.7179 | 0.9794 | 0.9690 | 0.8476 | 0.8895 | 0.8740 |
| 370 | 0.8571 | 0.9233 | 0.9785 | 0.8444 | 0.7179 | 0.9776 | 0.9690 | 0.8459 | 0.8897 | 0.8742 |
| 371 | 0.8536 | 0.9233 | 0.9785 | 0.8479 | 0.7083 | 0.9776 | 0.9673 | 0.8529 | 0.8892 | 0.8737 |
| 372 | 0.8501 | 0.9216 | 0.9785 | 0.8479 | 0.7217 | 0.9776 | 0.9673 | 0.8494 | 0.8897 | 0.8742 |
| 373 | 0.8501 | 0.9216 | 0.9785 | 0.8479 | 0.7217 | 0.9776 | 0.9673 | 0.8494 | 0.8897 | 0.8742 |
| 374 | 0.8554 | 0.9216 | 0.9785 | 0.8479 | 0.7198 | 0.9776 | 0.9673 | 0.8476 | 0.8899 | 0.8745 |
| 375 | 0.8536 | 0.9216 | 0.9766 | 0.8479 | 0.7198 | 0.9776 | 0.9673 | 0.8494 | 0.8897 | 0.8742 |
| 376 | 0.8519 | 0.9216 | 0.9785 | 0.8462 | 0.7179 | 0.9776 | 0.9673 | 0.8511 | 0.8895 | 0.8740 |
| 377 | 0.8519 | 0.9216 | 0.9785 | 0.8497 | 0.7236 | 0.9776 | 0.9690 | 0.8546 | 0.8913 | 0.8760 |
| 378 | 0.8519 | 0.9216 | 0.9785 | 0.8497 | 0.7217 | 0.9776 | 0.9690 | 0.8546 | 0.8910 | 0.8758 |
| 379 | 0.8536 | 0.9199 | 0.9785 | 0.8479 | 0.7198 | 0.9776 | 0.9707 | 0.8564 | 0.8910 | 0.8758 |
| 380 | 0.8554 | 0.9182 | 0.9785 | 0.8479 | 0.7198 | 0.9776 | 0.9707 | 0.8546 | 0.8908 | 0.8755 |
| 381 | 0.8536 | 0.9182 | 0.9785 | 0.8462 | 0.7198 | 0.9776 | 0.9707 | 0.8564 | 0.8906 | 0.8753 |
| 382 | 0.8624 | 0.9182 | 0.9785 | 0.8444 | 0.7255 | 0.9776 | 0.9690 | 0.8581 | 0.8922 | 0.8770 |
| 383 | 0.8624 | 0.9182 | 0.9785 | 0.8444 | 0.7255 | 0.9776 | 0.9690 | 0.8599 | 0.8924 | 0.8773 |
| 384 | 0.8607 | 0.9182 | 0.9785 | 0.8462 | 0.7255 | 0.9776 | 0.9690 | 0.8564 | 0.8919 | 0.8768 |
| 385 | 0.8642 | 0.9165 | 0.9785 | 0.8497 | 0.7294 | 0.9794 | 0.9690 | 0.8546 | 0.8931 | 0.8781 |
| 386 | 0.8642 | 0.9182 | 0.9785 | 0.8479 | 0.7332 | 0.9794 | 0.9690 | 0.8546 | 0.8935 | 0.8786 |
| 387 | 0.8624 | 0.9182 | 0.9785 | 0.8479 | 0.7351 | 0.9794 | 0.9690 | 0.8546 | 0.8935 | 0.8786 |
| 388 | 0.8660 | 0.9199 | 0.9785 | 0.8479 | 0.7332 | 0.9794 | 0.9707 | 0.8564 | 0.8944 | 0.8796 |
| 389 | 0.8642 | 0.9199 | 0.9766 | 0.8444 | 0.7332 | 0.9794 | 0.9690 | 0.8564 | 0.8933 | 0.8783 |
| 390 | 0.8607 | 0.9182 | 0.9785 | 0.8426 | 0.7255 | 0.9813 | 0.9690 | 0.8564 | 0.8919 | 0.8768 |
| 391 | 0.8607 | 0.9182 | 0.9785 | 0.8444 | 0.7255 | 0.9813 | 0.9690 | 0.8599 | 0.8926 | 0.8776 |
| 392 | 0.8607 | 0.9182 | 0.9785 | 0.8444 | 0.7274 | 0.9813 | 0.9690 | 0.8581 | 0.8926 | 0.8776 |
| 393 | 0.8607 | 0.9182 | 0.9785 | 0.8462 | 0.7255 | 0.9813 | 0.9690 | 0.8564 | 0.8924 | 0.8773 |
| 394 | 0.8607 | 0.9182 | 0.9785 | 0.8462 | 0.7236 | 0.9813 | 0.9690 | 0.8564 | 0.8922 | 0.8771 |
| 395 | 0.8607 | 0.9182 | 0.9785 | 0.8462 | 0.7236 | 0.9813 | 0.9690 | 0.8564 | 0.8922 | 0.8770 |
| 396 | 0.8713 | 0.9199 | 0.9766 | 0.8354 | 0.7198 | 0.9794 | 0.9690 | 0.8564 | 0.8915 | 0.8763 |
| 397 | 0.8695 | 0.9199 | 0.9766 | 0.8354 | 0.7198 | 0.9794 | 0.9690 | 0.8564 | 0.8913 | 0.8760 |
| 398 | 0.8748 | 0.9199 | 0.9766 | 0.8390 | 0.7217 | 0.9794 | 0.9690 | 0.8546 | 0.8924 | 0.8773 |
| 399 | 0.8748 | 0.9199 | 0.9766 | 0.8408 | 0.7236 | 0.9794 | 0.9690 | 0.8581 | 0.8933 | 0.8784 |
| 400 | 0.8730 | 0.9199 | 0.9766 | 0.8408 | 0.7236 | 0.9794 | 0.9690 | 0.8564 | 0.8928 | 0.8778 |
| 401 | 0.8730 | 0.9199 | 0.9766 | 0.8426 | 0.7236 | 0.9794 | 0.9690 | 0.8564 | 0.8931 | 0.8781 |
| 402 | 0.8713 | 0.9199 | 0.9766 | 0.8390 | 0.7179 | 0.9813 | 0.9690 | 0.8546 | 0.8917 | 0.8765 |
| 403 | 0.8713 | 0.9199 | 0.9766 | 0.8390 | 0.7179 | 0.9813 | 0.9690 | 0.8546 | 0.8917 | 0.8766 |
| 404 | 0.8713 | 0.9199 | 0.9766 | 0.8372 | 0.7294 | 0.9776 | 0.9690 | 0.8599 | 0.8931 | 0.8781 |
| 405 | 0.8713 | 0.9216 | 0.9766 | 0.8390 | 0.7294 | 0.9794 | 0.9673 | 0.8581 | 0.8933 | 0.8783 |
| 406 | 0.8695 | 0.9199 | 0.9766 | 0.8390 | 0.7274 | 0.9794 | 0.9673 | 0.8599 | 0.8928 | 0.8778 |
| 407 | 0.8695 | 0.9199 | 0.9766 | 0.8408 | 0.7313 | 0.9794 | 0.9673 | 0.8616 | 0.8938 | 0.8788 |
| 408 | 0.8677 | 0.9216 | 0.9766 | 0.8462 | 0.7313 | 0.9794 | 0.9673 | 0.8599 | 0.8942 | 0.8794 |
| 409 | 0.8695 | 0.9199 | 0.9766 | 0.8462 | 0.7294 | 0.9794 | 0.9673 | 0.8581 | 0.8938 | 0.8789 |
| 410 | 0.8695 | 0.9182 | 0.9766 | 0.8462 | 0.7274 | 0.9776 | 0.9673 | 0.8581 | 0.8931 | 0.8781 |
| 411 | 0.8677 | 0.9182 | 0.9766 | 0.8462 | 0.7274 | 0.9776 | 0.9673 | 0.8581 | 0.8928 | 0.8778 |
| 412 | 0.8677 | 0.9199 | 0.9766 | 0.8444 | 0.7294 | 0.9776 | 0.9690 | 0.8581 | 0.8933 | 0.8783 |
| 413 | 0.8677 | 0.9233 | 0.9805 | 0.8497 | 0.7294 | 0.9794 | 0.9690 | 0.8616 | 0.8956 | 0.8809 |
| 414 | 0.8660 | 0.9233 | 0.9805 | 0.8515 | 0.7332 | 0.9794 | 0.9673 | 0.8651 | 0.8962 | 0.8817 |
| 415 | 0.8677 | 0.9250 | 0.9785 | 0.8497 | 0.7313 | 0.9794 | 0.9673 | 0.8651 | 0.8960 | 0.8814 |
| 416 | 0.8695 | 0.9250 | 0.9785 | 0.8479 | 0.7332 | 0.9794 | 0.9673 | 0.8599 | 0.8956 | 0.8809 |
| 417 | 0.8695 | 0.9250 | 0.9785 | 0.8497 | 0.7313 | 0.9794 | 0.9673 | 0.8616 | 0.8958 | 0.8812 |
| 418 | 0.8695 | 0.9233 | 0.9785 | 0.8497 | 0.7313 | 0.9794 | 0.9690 | 0.8616 | 0.8958 | 0.8812 |
| 419 | 0.8642 | 0.9250 | 0.9766 | 0.8462 | 0.7332 | 0.9813 | 0.9673 | 0.8651 | 0.8953 | 0.8806 |
| 420 | 0.8642 | 0.9250 | 0.9766 | 0.8462 | 0.7332 | 0.9813 | 0.9673 | 0.8651 | 0.8953 | 0.8806 |
| 421 | 0.8642 | 0.9250 | 0.9766 | 0.8462 | 0.7351 | 0.9794 | 0.9673 | 0.8669 | 0.8956 | 0.8809 |
| 422 | 0.8642 | 0.9250 | 0.9766 | 0.8462 | 0.7332 | 0.9794 | 0.9673 | 0.8669 | 0.8953 | 0.8806 |
| 423 | 0.8642 | 0.9250 | 0.9766 | 0.8462 | 0.7351 | 0.9813 | 0.9690 | 0.8651 | 0.8958 | 0.8811 |
| 424 | 0.8642 | 0.9250 | 0.9766 | 0.8426 | 0.7294 | 0.9813 | 0.9707 | 0.8651 | 0.8949 | 0.8801 |
| 425 | 0.8677 | 0.9199 | 0.9766 | 0.8408 | 0.7274 | 0.9813 | 0.9707 | 0.8616 | 0.8938 | 0.8788 |
| 426 | 0.8677 | 0.9216 | 0.9785 | 0.8408 | 0.7274 | 0.9813 | 0.9707 | 0.8616 | 0.8942 | 0.8794 |
| 427 | 0.8677 | 0.9216 | 0.9785 | 0.8408 | 0.7274 | 0.9813 | 0.9707 | 0.8616 | 0.8942 | 0.8794 |
| 428 | 0.8677 | 0.9216 | 0.9785 | 0.8408 | 0.7274 | 0.9813 | 0.9707 | 0.8616 | 0.8942 | 0.8794 |
| 429 | 0.8677 | 0.9250 | 0.9805 | 0.8336 | 0.7255 | 0.9813 | 0.9707 | 0.8599 | 0.8935 | 0.8786 |
| 430 | 0.8695 | 0.9233 | 0.9805 | 0.8354 | 0.7255 | 0.9813 | 0.9707 | 0.8581 | 0.8935 | 0.8786 |
| 431 | 0.8713 | 0.9233 | 0.9805 | 0.8390 | 0.7255 | 0.9813 | 0.9707 | 0.8599 | 0.8944 | 0.8796 |
| 432 | 0.8677 | 0.9250 | 0.9805 | 0.8372 | 0.7274 | 0.9813 | 0.9707 | 0.8581 | 0.8940 | 0.8791 |
| 433 | 0.8660 | 0.9233 | 0.9805 | 0.8390 | 0.7255 | 0.9813 | 0.9707 | 0.8581 | 0.8935 | 0.8786 |
| 434 | 0.8642 | 0.9233 | 0.9785 | 0.8390 | 0.7274 | 0.9813 | 0.9707 | 0.8564 | 0.8931 | 0.8780 |
| 435 | 0.8660 | 0.9233 | 0.9785 | 0.8390 | 0.7255 | 0.9813 | 0.9707 | 0.8599 | 0.8935 | 0.8786 |
| 436 | 0.8677 | 0.9233 | 0.9785 | 0.8426 | 0.7294 | 0.9813 | 0.9707 | 0.8581 | 0.8944 | 0.8796 |
| 437 | 0.8695 | 0.9233 | 0.9785 | 0.8408 | 0.7294 | 0.9813 | 0.9707 | 0.8581 | 0.8944 | 0.8796 |
| 438 | 0.8677 | 0.9233 | 0.9785 | 0.8390 | 0.7313 | 0.9813 | 0.9707 | 0.8581 | 0.8942 | 0.8793 |
| 439 | 0.8642 | 0.9233 | 0.9805 | 0.8390 | 0.7313 | 0.9794 | 0.9707 | 0.8564 | 0.8935 | 0.8785 |
| 440 | 0.8642 | 0.9233 | 0.9805 | 0.8390 | 0.7274 | 0.9794 | 0.9707 | 0.8564 | 0.8931 | 0.8780 |
| 441 | 0.8642 | 0.9233 | 0.9805 | 0.8390 | 0.7255 | 0.9794 | 0.9707 | 0.8546 | 0.8926 | 0.8775 |
| 442 | 0.8642 | 0.9233 | 0.9805 | 0.8408 | 0.7217 | 0.9794 | 0.9707 | 0.8546 | 0.8924 | 0.8773 |
| 443 | 0.8748 | 0.9233 | 0.9805 | 0.8408 | 0.7236 | 0.9794 | 0.9690 | 0.8546 | 0.8938 | 0.8789 |
| 444 | 0.8748 | 0.9216 | 0.9785 | 0.8408 | 0.7255 | 0.9776 | 0.9690 | 0.8616 | 0.8942 | 0.8794 |
| 445 | 0.8748 | 0.9216 | 0.9805 | 0.8408 | 0.7255 | 0.9776 | 0.9690 | 0.8616 | 0.8944 | 0.8796 |
| 446 | 0.8765 | 0.9216 | 0.9805 | 0.8408 | 0.7236 | 0.9776 | 0.9690 | 0.8616 | 0.8944 | 0.8796 |
| 447 | 0.8765 | 0.9233 | 0.9805 | 0.8390 | 0.7236 | 0.9776 | 0.9690 | 0.8616 | 0.8944 | 0.8796 |
| 448 | 0.8765 | 0.9233 | 0.9805 | 0.8408 | 0.7236 | 0.9776 | 0.9690 | 0.8616 | 0.8947 | 0.8799 |
| 449 | 0.8765 | 0.9233 | 0.9785 | 0.8372 | 0.7255 | 0.9776 | 0.9690 | 0.8634 | 0.8944 | 0.8796 |
| 450 | 0.8730 | 0.9199 | 0.9785 | 0.8372 | 0.7274 | 0.9794 | 0.9690 | 0.8616 | 0.8938 | 0.8788 |
| 451 | 0.8660 | 0.9199 | 0.9805 | 0.8390 | 0.7313 | 0.9794 | 0.9690 | 0.8616 | 0.8938 | 0.8788 |
| 452 | 0.8642 | 0.9216 | 0.9805 | 0.8408 | 0.7313 | 0.9794 | 0.9690 | 0.8599 | 0.8938 | 0.8788 |
| 453 | 0.8642 | 0.9216 | 0.9805 | 0.8426 | 0.7313 | 0.9794 | 0.9690 | 0.8634 | 0.8944 | 0.8796 |
| 454 | 0.8677 | 0.9216 | 0.9805 | 0.8426 | 0.7313 | 0.9794 | 0.9690 | 0.8564 | 0.8940 | 0.8791 |
| 455 | 0.8660 | 0.9216 | 0.9785 | 0.8444 | 0.7313 | 0.9794 | 0.9690 | 0.8581 | 0.8940 | 0.8791 |
| 456 | 0.8677 | 0.9216 | 0.9785 | 0.8426 | 0.7313 | 0.9794 | 0.9690 | 0.8581 | 0.8940 | 0.8791 |
| 457 | 0.8642 | 0.9199 | 0.9805 | 0.8426 | 0.7274 | 0.9794 | 0.9690 | 0.8564 | 0.8928 | 0.8778 |
| 458 | 0.8642 | 0.9199 | 0.9785 | 0.8426 | 0.7274 | 0.9794 | 0.9690 | 0.8581 | 0.8928 | 0.8778 |
| 459 | 0.8660 | 0.9199 | 0.9805 | 0.8444 | 0.7294 | 0.9794 | 0.9690 | 0.8599 | 0.8940 | 0.8791 |
| 460 | 0.8660 | 0.9216 | 0.9785 | 0.8408 | 0.7274 | 0.9813 | 0.9690 | 0.8634 | 0.8940 | 0.8791 |
| 461 | 0.8660 | 0.9199 | 0.9785 | 0.8426 | 0.7294 | 0.9813 | 0.9690 | 0.8634 | 0.8942 | 0.8793 |
| 462 | 0.8730 | 0.9199 | 0.9805 | 0.8479 | 0.7274 | 0.9813 | 0.9673 | 0.8634 | 0.8956 | 0.8809 |
| 463 | 0.8713 | 0.9216 | 0.9805 | 0.8462 | 0.7313 | 0.9813 | 0.9673 | 0.8634 | 0.8958 | 0.8812 |
| 464 | 0.8713 | 0.9199 | 0.9805 | 0.8462 | 0.7294 | 0.9813 | 0.9673 | 0.8634 | 0.8953 | 0.8807 |
| 465 | 0.8713 | 0.9216 | 0.9805 | 0.8462 | 0.7294 | 0.9813 | 0.9673 | 0.8651 | 0.8958 | 0.8812 |
| 466 | 0.8713 | 0.9216 | 0.9805 | 0.8444 | 0.7274 | 0.9813 | 0.9673 | 0.8651 | 0.8953 | 0.8807 |
| 467 | 0.8695 | 0.9216 | 0.9785 | 0.8444 | 0.7236 | 0.9813 | 0.9673 | 0.8669 | 0.8947 | 0.8799 |
| 468 | 0.8730 | 0.9216 | 0.9805 | 0.8497 | 0.7217 | 0.9813 | 0.9673 | 0.8651 | 0.8956 | 0.8809 |
| 469 | 0.8730 | 0.9216 | 0.9805 | 0.8515 | 0.7236 | 0.9813 | 0.9673 | 0.8651 | 0.8960 | 0.8814 |
| 470 | 0.8730 | 0.9216 | 0.9805 | 0.8462 | 0.7236 | 0.9813 | 0.9673 | 0.8651 | 0.8953 | 0.8807 |
| 471 | 0.8748 | 0.9216 | 0.9805 | 0.8497 | 0.7294 | 0.9813 | 0.9673 | 0.8669 | 0.8969 | 0.8825 |
| 472 | 0.8748 | 0.9216 | 0.9805 | 0.8497 | 0.7294 | 0.9813 | 0.9673 | 0.8669 | 0.8969 | 0.8825 |
| 473 | 0.8783 | 0.9182 | 0.9805 | 0.8479 | 0.7332 | 0.9813 | 0.9673 | 0.8687 | 0.8974 | 0.8830 |
| 474 | 0.8765 | 0.9182 | 0.9805 | 0.8462 | 0.7332 | 0.9813 | 0.9690 | 0.8687 | 0.8971 | 0.8827 |
| 475 | 0.8730 | 0.9148 | 0.9805 | 0.8462 | 0.7274 | 0.9813 | 0.9690 | 0.8687 | 0.8956 | 0.8809 |
| 476 | 0.8730 | 0.9148 | 0.9805 | 0.8479 | 0.7274 | 0.9813 | 0.9690 | 0.8669 | 0.8956 | 0.8809 |
| 477 | 0.8748 | 0.9165 | 0.9805 | 0.8408 | 0.7294 | 0.9813 | 0.9690 | 0.8704 | 0.8958 | 0.8812 |
| 478 | 0.8748 | 0.9165 | 0.9805 | 0.8408 | 0.7294 | 0.9813 | 0.9690 | 0.8722 | 0.8960 | 0.8814 |
| 479 | 0.8730 | 0.9165 | 0.9805 | 0.8426 | 0.7255 | 0.9813 | 0.9690 | 0.8722 | 0.8956 | 0.8809 |
| 480 | 0.8748 | 0.9165 | 0.9805 | 0.8444 | 0.7313 | 0.9813 | 0.9673 | 0.8722 | 0.8965 | 0.8820 |
| 481 | 0.8748 | 0.9165 | 0.9805 | 0.8444 | 0.7332 | 0.9813 | 0.9673 | 0.8722 | 0.8967 | 0.8822 |
| 482 | 0.8765 | 0.9165 | 0.9805 | 0.8462 | 0.7313 | 0.9813 | 0.9673 | 0.8687 | 0.8965 | 0.8820 |
| 483 | 0.8765 | 0.9182 | 0.9805 | 0.8479 | 0.7313 | 0.9813 | 0.9673 | 0.8687 | 0.8969 | 0.8825 |
| 484 | 0.8765 | 0.9182 | 0.9805 | 0.8479 | 0.7332 | 0.9813 | 0.9673 | 0.8687 | 0.8971 | 0.8827 |
| 485 | 0.8695 | 0.9165 | 0.9805 | 0.8444 | 0.7332 | 0.9813 | 0.9690 | 0.8704 | 0.8960 | 0.8814 |
| 486 | 0.8713 | 0.9165 | 0.9805 | 0.8408 | 0.7294 | 0.9813 | 0.9673 | 0.8669 | 0.8947 | 0.8799 |
| 487 | 0.8695 | 0.9148 | 0.9805 | 0.8390 | 0.7294 | 0.9813 | 0.9673 | 0.8669 | 0.8940 | 0.8791 |
| 488 | 0.8713 | 0.9148 | 0.9805 | 0.8408 | 0.7313 | 0.9813 | 0.9673 | 0.8687 | 0.8949 | 0.8802 |
| 489 | 0.8713 | 0.9148 | 0.9805 | 0.8390 | 0.7294 | 0.9813 | 0.9673 | 0.8704 | 0.8947 | 0.8799 |
| 490 | 0.8730 | 0.9148 | 0.9805 | 0.8390 | 0.7313 | 0.9813 | 0.9673 | 0.8722 | 0.8953 | 0.8807 |
| 491 | 0.8748 | 0.9148 | 0.9805 | 0.8408 | 0.7351 | 0.9813 | 0.9673 | 0.8687 | 0.8958 | 0.8812 |
| 492 | 0.8730 | 0.9182 | 0.9805 | 0.8354 | 0.7351 | 0.9813 | 0.9673 | 0.8651 | 0.8949 | 0.8802 |
| 493 | 0.8730 | 0.9165 | 0.9824 | 0.8390 | 0.7351 | 0.9813 | 0.9673 | 0.8669 | 0.8956 | 0.8809 |
| 494 | 0.8765 | 0.9165 | 0.9824 | 0.8372 | 0.7313 | 0.9813 | 0.9673 | 0.8687 | 0.8956 | 0.8809 |
| 495 | 0.8765 | 0.9199 | 0.9824 | 0.8354 | 0.7313 | 0.9813 | 0.9673 | 0.8687 | 0.8958 | 0.8812 |
| 496 | 0.8748 | 0.9216 | 0.9824 | 0.8336 | 0.7428 | 0.9813 | 0.9673 | 0.8687 | 0.8969 | 0.8824 |
| 497 | 0.8765 | 0.9216 | 0.9824 | 0.8336 | 0.7370 | 0.9813 | 0.9673 | 0.8687 | 0.8965 | 0.8819 |
| 498 | 0.8765 | 0.9216 | 0.9824 | 0.8372 | 0.7351 | 0.9813 | 0.9673 | 0.8704 | 0.8969 | 0.8825 |
| 499 | 0.8730 | 0.9199 | 0.9824 | 0.8336 | 0.7351 | 0.9794 | 0.9673 | 0.8722 | 0.8958 | 0.8811 |
| 500 | 0.8783 | 0.9199 | 0.9824 | 0.8336 | 0.7370 | 0.9794 | 0.9673 | 0.8687 | 0.8962 | 0.8817 |
| 501 | 0.8695 | 0.9216 | 0.9805 | 0.8336 | 0.7332 | 0.9776 | 0.9673 | 0.8669 | 0.8942 | 0.8793 |
| 502 | 0.8695 | 0.9216 | 0.9805 | 0.8372 | 0.7332 | 0.9776 | 0.9673 | 0.8669 | 0.8947 | 0.8799 |
| 503 | 0.8677 | 0.9199 | 0.9805 | 0.8283 | 0.7294 | 0.9794 | 0.9673 | 0.8704 | 0.8933 | 0.8783 |
| 504 | 0.8730 | 0.9199 | 0.9805 | 0.8283 | 0.7294 | 0.9794 | 0.9673 | 0.8687 | 0.8938 | 0.8788 |
| 505 | 0.8713 | 0.9165 | 0.9785 | 0.8301 | 0.7274 | 0.9776 | 0.9673 | 0.8704 | 0.8928 | 0.8778 |
| 506 | 0.8713 | 0.9165 | 0.9785 | 0.8318 | 0.7294 | 0.9794 | 0.9673 | 0.8704 | 0.8935 | 0.8786 |
| 507 | 0.8713 | 0.9165 | 0.9785 | 0.8318 | 0.7294 | 0.9794 | 0.9673 | 0.8704 | 0.8935 | 0.8786 |
| 508 | 0.8713 | 0.9165 | 0.9805 | 0.8301 | 0.7294 | 0.9794 | 0.9673 | 0.8704 | 0.8935 | 0.8786 |
| 509 | 0.8730 | 0.9199 | 0.9805 | 0.8354 | 0.7313 | 0.9794 | 0.9673 | 0.8687 | 0.8949 | 0.8801 |
| 510 | 0.8713 | 0.9182 | 0.9805 | 0.8354 | 0.7313 | 0.9776 | 0.9673 | 0.8722 | 0.8947 | 0.8799 |
| 511 | 0.8713 | 0.9182 | 0.9805 | 0.8354 | 0.7313 | 0.9776 | 0.9673 | 0.8722 | 0.8947 | 0.8799 |
| 512 | 0.8695 | 0.9182 | 0.9805 | 0.8354 | 0.7313 | 0.9776 | 0.9673 | 0.8722 | 0.8944 | 0.8796 |
| 513 | 0.8730 | 0.9182 | 0.9805 | 0.8354 | 0.7313 | 0.9776 | 0.9673 | 0.8722 | 0.8949 | 0.8801 |
| 514 | 0.8730 | 0.9148 | 0.9805 | 0.8354 | 0.7274 | 0.9776 | 0.9673 | 0.8669 | 0.8933 | 0.8783 |
| 515 | 0.8748 | 0.9165 | 0.9805 | 0.8408 | 0.7332 | 0.9776 | 0.9690 | 0.8687 | 0.8956 | 0.8809 |
| 516 | 0.8748 | 0.9165 | 0.9805 | 0.8390 | 0.7332 | 0.9776 | 0.9707 | 0.8687 | 0.8956 | 0.8809 |
| 517 | 0.8730 | 0.9165 | 0.9805 | 0.8408 | 0.7332 | 0.9776 | 0.9707 | 0.8651 | 0.8951 | 0.8804 |
| 518 | 0.8730 | 0.9216 | 0.9785 | 0.8354 | 0.7274 | 0.9776 | 0.9707 | 0.8651 | 0.8942 | 0.8794 |
| 519 | 0.8695 | 0.9216 | 0.9785 | 0.8301 | 0.7332 | 0.9776 | 0.9707 | 0.8634 | 0.8935 | 0.8786 |
| 520 | 0.8713 | 0.9216 | 0.9785 | 0.8301 | 0.7332 | 0.9776 | 0.9707 | 0.8669 | 0.8942 | 0.8793 |
| 521 | 0.8695 | 0.9199 | 0.9785 | 0.8301 | 0.7332 | 0.9776 | 0.9707 | 0.8651 | 0.8935 | 0.8786 |
| 522 | 0.8713 | 0.9233 | 0.9785 | 0.8318 | 0.7370 | 0.9776 | 0.9707 | 0.8634 | 0.8947 | 0.8799 |
| 523 | 0.8713 | 0.9250 | 0.9785 | 0.8336 | 0.7351 | 0.9757 | 0.9707 | 0.8634 | 0.8947 | 0.8799 |
| 524 | 0.8677 | 0.9250 | 0.9785 | 0.8265 | 0.7351 | 0.9757 | 0.9707 | 0.8651 | 0.8935 | 0.8786 |
| 525 | 0.8677 | 0.9250 | 0.9785 | 0.8229 | 0.7313 | 0.9757 | 0.9707 | 0.8634 | 0.8924 | 0.8773 |
| 526 | 0.8695 | 0.9250 | 0.9785 | 0.8283 | 0.7332 | 0.9757 | 0.9707 | 0.8669 | 0.8940 | 0.8791 |
| 527 | 0.8695 | 0.9250 | 0.9785 | 0.8318 | 0.7294 | 0.9757 | 0.9707 | 0.8704 | 0.8944 | 0.8796 |
| 528 | 0.8730 | 0.9250 | 0.9785 | 0.8283 | 0.7313 | 0.9757 | 0.9707 | 0.8704 | 0.8947 | 0.8799 |
| 529 | 0.8730 | 0.9250 | 0.9785 | 0.8301 | 0.7313 | 0.9757 | 0.9690 | 0.8704 | 0.8947 | 0.8799 |
| 530 | 0.8730 | 0.9250 | 0.9785 | 0.8301 | 0.7332 | 0.9757 | 0.9690 | 0.8704 | 0.8949 | 0.8801 |
| 531 | 0.8730 | 0.9250 | 0.9785 | 0.8301 | 0.7274 | 0.9757 | 0.9690 | 0.8669 | 0.8938 | 0.8788 |
| 532 | 0.8713 | 0.9250 | 0.9785 | 0.8283 | 0.7294 | 0.9757 | 0.9690 | 0.8687 | 0.8938 | 0.8788 |
| 533 | 0.8713 | 0.9250 | 0.9766 | 0.8283 | 0.7294 | 0.9757 | 0.9690 | 0.8687 | 0.8935 | 0.8786 |
| 534 | 0.8713 | 0.9267 | 0.9766 | 0.8283 | 0.7313 | 0.9757 | 0.9690 | 0.8651 | 0.8935 | 0.8786 |
| 535 | 0.8730 | 0.9267 | 0.9766 | 0.8318 | 0.7274 | 0.9757 | 0.9690 | 0.8651 | 0.8938 | 0.8788 |
| 536 | 0.8730 | 0.9267 | 0.9766 | 0.8283 | 0.7274 | 0.9757 | 0.9690 | 0.8669 | 0.8935 | 0.8786 |
| 537 | 0.8713 | 0.9267 | 0.9766 | 0.8265 | 0.7274 | 0.9757 | 0.9690 | 0.8669 | 0.8931 | 0.8780 |
| 538 | 0.8713 | 0.9267 | 0.9766 | 0.8247 | 0.7274 | 0.9757 | 0.9690 | 0.8669 | 0.8928 | 0.8778 |
| 539 | 0.8660 | 0.9250 | 0.9766 | 0.8265 | 0.7255 | 0.9757 | 0.9690 | 0.8599 | 0.8910 | 0.8757 |
| 540 | 0.8677 | 0.9250 | 0.9766 | 0.8265 | 0.7236 | 0.9738 | 0.9690 | 0.8599 | 0.8908 | 0.8755 |
| 541 | 0.8713 | 0.9250 | 0.9766 | 0.8265 | 0.7255 | 0.9738 | 0.9690 | 0.8599 | 0.8915 | 0.8763 |
| 542 | 0.8695 | 0.9250 | 0.9766 | 0.8265 | 0.7313 | 0.9738 | 0.9690 | 0.8599 | 0.8919 | 0.8768 |
| 543 | 0.8695 | 0.9267 | 0.9766 | 0.8283 | 0.7255 | 0.9738 | 0.9690 | 0.8599 | 0.8917 | 0.8765 |
| 544 | 0.8695 | 0.9267 | 0.9766 | 0.8283 | 0.7236 | 0.9738 | 0.9690 | 0.8616 | 0.8917 | 0.8765 |
| 545 | 0.8730 | 0.9250 | 0.9766 | 0.8283 | 0.7255 | 0.9738 | 0.9690 | 0.8616 | 0.8922 | 0.8770 |
| 546 | 0.8730 | 0.9250 | 0.9766 | 0.8283 | 0.7236 | 0.9738 | 0.9690 | 0.8616 | 0.8919 | 0.8768 |
| 547 | 0.8730 | 0.9250 | 0.9766 | 0.8318 | 0.7294 | 0.9738 | 0.9690 | 0.8599 | 0.8928 | 0.8778 |
| 548 | 0.8730 | 0.9233 | 0.9766 | 0.8336 | 0.7294 | 0.9738 | 0.9690 | 0.8634 | 0.8933 | 0.8783 |
| 549 | 0.8748 | 0.9233 | 0.9766 | 0.8318 | 0.7294 | 0.9738 | 0.9690 | 0.8634 | 0.8933 | 0.8783 |
| 550 | 0.8748 | 0.9233 | 0.9766 | 0.8336 | 0.7274 | 0.9738 | 0.9690 | 0.8634 | 0.8933 | 0.8783 |
| 551 | 0.8730 | 0.9233 | 0.9766 | 0.8301 | 0.7274 | 0.9738 | 0.9690 | 0.8634 | 0.8926 | 0.8776 |
| 552 | 0.8748 | 0.9233 | 0.9766 | 0.8336 | 0.7140 | 0.9757 | 0.9690 | 0.8651 | 0.8922 | 0.8771 |
| 553 | 0.8713 | 0.9233 | 0.9766 | 0.8318 | 0.7159 | 0.9757 | 0.9690 | 0.8634 | 0.8915 | 0.8763 |
| 554 | 0.8713 | 0.9233 | 0.9766 | 0.8318 | 0.7159 | 0.9757 | 0.9690 | 0.8634 | 0.8915 | 0.8763 |
| 555 | 0.8713 | 0.9233 | 0.9766 | 0.8283 | 0.7179 | 0.9757 | 0.9690 | 0.8634 | 0.8913 | 0.8760 |
| 556 | 0.8713 | 0.9233 | 0.9766 | 0.8318 | 0.7236 | 0.9757 | 0.9690 | 0.8634 | 0.8924 | 0.8773 |
| 557 | 0.8713 | 0.9233 | 0.9766 | 0.8301 | 0.7255 | 0.9757 | 0.9690 | 0.8634 | 0.8924 | 0.8773 |
| 558 | 0.8730 | 0.9233 | 0.9766 | 0.8265 | 0.7274 | 0.9757 | 0.9690 | 0.8616 | 0.8922 | 0.8770 |
| 559 | 0.8713 | 0.9233 | 0.9766 | 0.8247 | 0.7313 | 0.9757 | 0.9690 | 0.8616 | 0.8922 | 0.8770 |
| 560 | 0.8713 | 0.9216 | 0.9766 | 0.8283 | 0.7313 | 0.9757 | 0.9690 | 0.8616 | 0.8924 | 0.8773 |
| 561 | 0.8713 | 0.9216 | 0.9766 | 0.8301 | 0.7332 | 0.9757 | 0.9690 | 0.8616 | 0.8928 | 0.8778 |
| 562 | 0.8730 | 0.9216 | 0.9766 | 0.8283 | 0.7313 | 0.9757 | 0.9690 | 0.8616 | 0.8926 | 0.8775 |
| 563 | 0.8713 | 0.9216 | 0.9766 | 0.8283 | 0.7313 | 0.9757 | 0.9690 | 0.8616 | 0.8924 | 0.8773 |
| 564 | 0.8695 | 0.9216 | 0.9785 | 0.8318 | 0.7332 | 0.9757 | 0.9673 | 0.8616 | 0.8928 | 0.8778 |
| 565 | 0.8713 | 0.9216 | 0.9785 | 0.8301 | 0.7332 | 0.9757 | 0.9673 | 0.8616 | 0.8928 | 0.8778 |
| 566 | 0.8730 | 0.9216 | 0.9805 | 0.8336 | 0.7274 | 0.9757 | 0.9673 | 0.8616 | 0.8931 | 0.8781 |
| 567 | 0.8730 | 0.9216 | 0.9785 | 0.8336 | 0.7255 | 0.9757 | 0.9673 | 0.8634 | 0.8928 | 0.8778 |
| 568 | 0.8730 | 0.9216 | 0.9805 | 0.8336 | 0.7255 | 0.9757 | 0.9673 | 0.8634 | 0.8931 | 0.8781 |
| 569 | 0.8713 | 0.9216 | 0.9805 | 0.8318 | 0.7255 | 0.9757 | 0.9673 | 0.8634 | 0.8926 | 0.8775 |
| 570 | 0.8713 | 0.9233 | 0.9824 | 0.8318 | 0.7255 | 0.9757 | 0.9673 | 0.8634 | 0.8931 | 0.8780 |
| 571 | 0.8713 | 0.9233 | 0.9824 | 0.8318 | 0.7255 | 0.9757 | 0.9673 | 0.8634 | 0.8931 | 0.8780 |
| 572 | 0.8713 | 0.9233 | 0.9824 | 0.8318 | 0.7255 | 0.9757 | 0.9673 | 0.8599 | 0.8926 | 0.8775 |
| 573 | 0.8713 | 0.9216 | 0.9805 | 0.8301 | 0.7274 | 0.9757 | 0.9673 | 0.8599 | 0.8922 | 0.8770 |
| 574 | 0.8713 | 0.9285 | 0.9824 | 0.8265 | 0.7236 | 0.9757 | 0.9673 | 0.8599 | 0.8924 | 0.8773 |
| 575 | 0.8713 | 0.9285 | 0.9824 | 0.8301 | 0.7217 | 0.9757 | 0.9673 | 0.8564 | 0.8922 | 0.8770 |
| 576 | 0.8677 | 0.9250 | 0.9805 | 0.8318 | 0.7255 | 0.9757 | 0.9673 | 0.8599 | 0.8922 | 0.8770 |
| 577 | 0.8730 | 0.9250 | 0.9824 | 0.8354 | 0.7198 | 0.9720 | 0.9690 | 0.8581 | 0.8924 | 0.8773 |
| 578 | 0.8748 | 0.9267 | 0.9824 | 0.8354 | 0.7179 | 0.9720 | 0.9690 | 0.8581 | 0.8926 | 0.8776 |
| 579 | 0.8748 | 0.9267 | 0.9824 | 0.8408 | 0.7198 | 0.9720 | 0.9690 | 0.8581 | 0.8935 | 0.8786 |
| 580 | 0.8730 | 0.9250 | 0.9824 | 0.8372 | 0.7159 | 0.9757 | 0.9690 | 0.8599 | 0.8928 | 0.8778 |
| 581 | 0.8713 | 0.9250 | 0.9824 | 0.8372 | 0.7255 | 0.9757 | 0.9673 | 0.8616 | 0.8938 | 0.8788 |
| 582 | 0.8695 | 0.9250 | 0.9824 | 0.8372 | 0.7274 | 0.9757 | 0.9673 | 0.8616 | 0.8938 | 0.8788 |
| 583 | 0.8713 | 0.9250 | 0.9824 | 0.8354 | 0.7236 | 0.9757 | 0.9673 | 0.8616 | 0.8933 | 0.8783 |
| 584 | 0.8713 | 0.9250 | 0.9824 | 0.8354 | 0.7217 | 0.9757 | 0.9673 | 0.8599 | 0.8928 | 0.8778 |
| 585 | 0.8713 | 0.9233 | 0.9824 | 0.8390 | 0.7236 | 0.9757 | 0.9673 | 0.8634 | 0.8938 | 0.8788 |
| 586 | 0.8748 | 0.9233 | 0.9824 | 0.8354 | 0.7274 | 0.9757 | 0.9673 | 0.8634 | 0.8942 | 0.8794 |
| 587 | 0.8748 | 0.9250 | 0.9824 | 0.8372 | 0.7294 | 0.9757 | 0.9673 | 0.8651 | 0.8951 | 0.8804 |
| 588 | 0.8730 | 0.9250 | 0.9824 | 0.8372 | 0.7255 | 0.9738 | 0.9673 | 0.8634 | 0.8940 | 0.8791 |
| 589 | 0.8713 | 0.9250 | 0.9824 | 0.8354 | 0.7274 | 0.9738 | 0.9673 | 0.8651 | 0.8940 | 0.8791 |
| 590 | 0.8677 | 0.9250 | 0.9824 | 0.8372 | 0.7255 | 0.9757 | 0.9673 | 0.8634 | 0.8935 | 0.8786 |
| 591 | 0.8677 | 0.9250 | 0.9824 | 0.8390 | 0.7274 | 0.9757 | 0.9673 | 0.8616 | 0.8938 | 0.8788 |
| 592 | 0.8677 | 0.9250 | 0.9824 | 0.8426 | 0.7274 | 0.9757 | 0.9673 | 0.8634 | 0.8944 | 0.8796 |
| 593 | 0.8713 | 0.9267 | 0.9824 | 0.8408 | 0.7274 | 0.9738 | 0.9673 | 0.8634 | 0.8947 | 0.8798 |
| 594 | 0.8730 | 0.9267 | 0.9824 | 0.8408 | 0.7274 | 0.9738 | 0.9673 | 0.8634 | 0.8949 | 0.8801 |
| 595 | 0.8765 | 0.9267 | 0.9824 | 0.8390 | 0.7274 | 0.9738 | 0.9673 | 0.8634 | 0.8951 | 0.8804 |
| 596 | 0.8783 | 0.9285 | 0.9824 | 0.8426 | 0.7274 | 0.9738 | 0.9673 | 0.8651 | 0.8962 | 0.8816 |
| 597 | 0.8730 | 0.9285 | 0.9824 | 0.8390 | 0.7274 | 0.9738 | 0.9673 | 0.8651 | 0.8951 | 0.8803 |
| 598 | 0.8730 | 0.9285 | 0.9824 | 0.8372 | 0.7274 | 0.9738 | 0.9673 | 0.8651 | 0.8949 | 0.8801 |
| 599 | 0.8765 | 0.9285 | 0.9824 | 0.8372 | 0.7332 | 0.9738 | 0.9673 | 0.8669 | 0.8962 | 0.8816 |
| 600 | 0.8783 | 0.9285 | 0.9824 | 0.8390 | 0.7313 | 0.9757 | 0.9673 | 0.8687 | 0.8969 | 0.8824 |
| 601 | 0.8765 | 0.9285 | 0.9805 | 0.8372 | 0.7274 | 0.9757 | 0.9673 | 0.8687 | 0.8958 | 0.8811 |
| 602 | 0.8748 | 0.9285 | 0.9805 | 0.8390 | 0.7274 | 0.9757 | 0.9673 | 0.8687 | 0.8958 | 0.8811 |
| 603 | 0.8730 | 0.9285 | 0.9805 | 0.8372 | 0.7236 | 0.9757 | 0.9673 | 0.8687 | 0.8949 | 0.8801 |
| 604 | 0.8748 | 0.9285 | 0.9805 | 0.8390 | 0.7274 | 0.9757 | 0.9673 | 0.8687 | 0.8958 | 0.8811 |
| 605 | 0.8765 | 0.9285 | 0.9805 | 0.8426 | 0.7294 | 0.9757 | 0.9656 | 0.8687 | 0.8965 | 0.8819 |
| 606 | 0.8765 | 0.9302 | 0.9805 | 0.8354 | 0.7294 | 0.9757 | 0.9639 | 0.8687 | 0.8956 | 0.8809 |
| 607 | 0.8748 | 0.9302 | 0.9805 | 0.8372 | 0.7236 | 0.9757 | 0.9639 | 0.8704 | 0.8951 | 0.8804 |
| 608 | 0.8748 | 0.9285 | 0.9824 | 0.8390 | 0.7313 | 0.9757 | 0.9639 | 0.8722 | 0.8965 | 0.8819 |
| 609 | 0.8748 | 0.9285 | 0.9824 | 0.8390 | 0.7313 | 0.9738 | 0.9656 | 0.8722 | 0.8965 | 0.8819 |
| 610 | 0.8748 | 0.9285 | 0.9824 | 0.8390 | 0.7313 | 0.9738 | 0.9656 | 0.8704 | 0.8962 | 0.8816 |
| 611 | 0.8748 | 0.9250 | 0.9824 | 0.8372 | 0.7294 | 0.9720 | 0.9690 | 0.8722 | 0.8958 | 0.8811 |
| 612 | 0.8765 | 0.9250 | 0.9824 | 0.8354 | 0.7274 | 0.9720 | 0.9690 | 0.8722 | 0.8956 | 0.8809 |
| 613 | 0.8783 | 0.9250 | 0.9824 | 0.8372 | 0.7313 | 0.9720 | 0.9690 | 0.8722 | 0.8965 | 0.8819 |
| 614 | 0.8783 | 0.9267 | 0.9824 | 0.8372 | 0.7294 | 0.9720 | 0.9690 | 0.8722 | 0.8965 | 0.8819 |
| 615 | 0.8783 | 0.9267 | 0.9824 | 0.8372 | 0.7294 | 0.9738 | 0.9690 | 0.8757 | 0.8971 | 0.8827 |
| 616 | 0.8783 | 0.9302 | 0.9805 | 0.8336 | 0.7274 | 0.9738 | 0.9673 | 0.8739 | 0.8962 | 0.8816 |
| 617 | 0.8783 | 0.9285 | 0.9805 | 0.8336 | 0.7274 | 0.9738 | 0.9673 | 0.8739 | 0.8960 | 0.8814 |
| 618 | 0.8783 | 0.9285 | 0.9824 | 0.8336 | 0.7274 | 0.9720 | 0.9673 | 0.8739 | 0.8960 | 0.8814 |
| 619 | 0.8765 | 0.9285 | 0.9824 | 0.8354 | 0.7294 | 0.9738 | 0.9673 | 0.8757 | 0.8967 | 0.8822 |
| 620 | 0.8783 | 0.9267 | 0.9805 | 0.8318 | 0.7294 | 0.9738 | 0.9656 | 0.8739 | 0.8956 | 0.8809 |
| 621 | 0.8783 | 0.9267 | 0.9805 | 0.8283 | 0.7236 | 0.9738 | 0.9656 | 0.8704 | 0.8940 | 0.8791 |
| 622 | 0.8730 | 0.9250 | 0.9805 | 0.8318 | 0.7255 | 0.9720 | 0.9690 | 0.8739 | 0.8944 | 0.8796 |
| 623 | 0.8801 | 0.9267 | 0.9785 | 0.8354 | 0.7217 | 0.9720 | 0.9690 | 0.8739 | 0.8953 | 0.8806 |
| 624 | 0.8783 | 0.9267 | 0.9785 | 0.8336 | 0.7217 | 0.9720 | 0.9690 | 0.8739 | 0.8949 | 0.8801 |
| 625 | 0.8765 | 0.9267 | 0.9785 | 0.8336 | 0.7236 | 0.9720 | 0.9690 | 0.8722 | 0.8947 | 0.8798 |
| 626 | 0.8765 | 0.9267 | 0.9785 | 0.8354 | 0.7217 | 0.9720 | 0.9690 | 0.8704 | 0.8944 | 0.8796 |
| 627 | 0.8801 | 0.9267 | 0.9785 | 0.8336 | 0.7217 | 0.9720 | 0.9673 | 0.8739 | 0.8949 | 0.8801 |
| 628 | 0.8801 | 0.9267 | 0.9785 | 0.8336 | 0.7217 | 0.9738 | 0.9673 | 0.8722 | 0.8949 | 0.8801 |
| 629 | 0.8801 | 0.9285 | 0.9785 | 0.8336 | 0.7236 | 0.9738 | 0.9673 | 0.8722 | 0.8953 | 0.8806 |
| 630 | 0.8765 | 0.9267 | 0.9785 | 0.8283 | 0.7274 | 0.9738 | 0.9656 | 0.8687 | 0.8938 | 0.8788 |
| 631 | 0.8818 | 0.9267 | 0.9805 | 0.8354 | 0.7236 | 0.9738 | 0.9656 | 0.8704 | 0.8953 | 0.8806 |
| 632 | 0.8836 | 0.9250 | 0.9805 | 0.8408 | 0.7236 | 0.9738 | 0.9656 | 0.8704 | 0.8960 | 0.8814 |
| 633 | 0.8818 | 0.9250 | 0.9805 | 0.8372 | 0.7236 | 0.9738 | 0.9656 | 0.8687 | 0.8951 | 0.8804 |
| 634 | 0.8818 | 0.9250 | 0.9805 | 0.8354 | 0.7217 | 0.9738 | 0.9656 | 0.8687 | 0.8947 | 0.8798 |
| 635 | 0.8801 | 0.9250 | 0.9805 | 0.8354 | 0.7236 | 0.9757 | 0.9656 | 0.8669 | 0.8947 | 0.8799 |
| 636 | 0.8889 | 0.9250 | 0.9805 | 0.8372 | 0.7274 | 0.9757 | 0.9656 | 0.8687 | 0.8967 | 0.8822 |
| 637 | 0.8854 | 0.9267 | 0.9824 | 0.8318 | 0.7255 | 0.9776 | 0.9639 | 0.8651 | 0.8953 | 0.8806 |
| 638 | 0.8836 | 0.9302 | 0.9805 | 0.8336 | 0.7236 | 0.9757 | 0.9656 | 0.8651 | 0.8953 | 0.8806 |
| 639 | 0.8818 | 0.9302 | 0.9805 | 0.8354 | 0.7179 | 0.9757 | 0.9656 | 0.8651 | 0.8947 | 0.8799 |
| 640 | 0.8818 | 0.9302 | 0.9805 | 0.8354 | 0.7198 | 0.9757 | 0.9656 | 0.8651 | 0.8949 | 0.8801 |
| 641 | 0.8836 | 0.9302 | 0.9805 | 0.8354 | 0.7198 | 0.9757 | 0.9673 | 0.8651 | 0.8953 | 0.8807 |
| 642 | 0.8854 | 0.9302 | 0.9824 | 0.8301 | 0.7198 | 0.9757 | 0.9656 | 0.8669 | 0.8951 | 0.8804 |
| 643 | 0.8854 | 0.9302 | 0.9824 | 0.8318 | 0.7140 | 0.9757 | 0.9656 | 0.8687 | 0.8949 | 0.8801 |
| 644 | 0.8854 | 0.9302 | 0.9824 | 0.8301 | 0.7121 | 0.9757 | 0.9656 | 0.8704 | 0.8947 | 0.8799 |
| 645 | 0.8871 | 0.9302 | 0.9824 | 0.8336 | 0.7121 | 0.9757 | 0.9656 | 0.8704 | 0.8953 | 0.8807 |
| 646 | 0.8889 | 0.9285 | 0.9805 | 0.8336 | 0.7121 | 0.9757 | 0.9656 | 0.8722 | 0.8953 | 0.8807 |
| 647 | 0.8871 | 0.9285 | 0.9805 | 0.8336 | 0.7159 | 0.9757 | 0.9639 | 0.8722 | 0.8953 | 0.8807 |
| 648 | 0.8836 | 0.9285 | 0.9785 | 0.8318 | 0.7198 | 0.9738 | 0.9639 | 0.8669 | 0.8940 | 0.8791 |
| 649 | 0.8818 | 0.9285 | 0.9785 | 0.8336 | 0.7159 | 0.9757 | 0.9639 | 0.8669 | 0.8938 | 0.8789 |
| 650 | 0.8854 | 0.9285 | 0.9785 | 0.8301 | 0.7140 | 0.9757 | 0.9639 | 0.8687 | 0.8938 | 0.8789 |
| 651 | 0.8871 | 0.9285 | 0.9785 | 0.8301 | 0.7179 | 0.9757 | 0.9639 | 0.8687 | 0.8944 | 0.8796 |
| 652 | 0.8871 | 0.9285 | 0.9785 | 0.8301 | 0.7159 | 0.9757 | 0.9639 | 0.8687 | 0.8942 | 0.8794 |
| 653 | 0.8889 | 0.9285 | 0.9785 | 0.8336 | 0.7179 | 0.9757 | 0.9639 | 0.8669 | 0.8949 | 0.8802 |
| 654 | 0.8871 | 0.9285 | 0.9785 | 0.8336 | 0.7198 | 0.9776 | 0.9639 | 0.8651 | 0.8949 | 0.8802 |
| 655 | 0.8836 | 0.9285 | 0.9766 | 0.8354 | 0.7198 | 0.9757 | 0.9639 | 0.8616 | 0.8938 | 0.8789 |
| 656 | 0.8854 | 0.9267 | 0.9785 | 0.8336 | 0.7179 | 0.9757 | 0.9639 | 0.8651 | 0.8940 | 0.8791 |
| 657 | 0.8854 | 0.9267 | 0.9805 | 0.8336 | 0.7179 | 0.9757 | 0.9639 | 0.8634 | 0.8940 | 0.8791 |
| 658 | 0.8907 | 0.9267 | 0.9805 | 0.8318 | 0.7198 | 0.9738 | 0.9639 | 0.8651 | 0.8947 | 0.8799 |
| 659 | 0.8889 | 0.9267 | 0.9805 | 0.8336 | 0.7198 | 0.9738 | 0.9639 | 0.8651 | 0.8947 | 0.8799 |
| 660 | 0.8924 | 0.9267 | 0.9805 | 0.8301 | 0.7236 | 0.9738 | 0.9639 | 0.8687 | 0.8956 | 0.8809 |
| 661 | 0.8942 | 0.9267 | 0.9805 | 0.8283 | 0.7198 | 0.9738 | 0.9621 | 0.8687 | 0.8949 | 0.8802 |
| 662 | 0.8942 | 0.9250 | 0.9805 | 0.8301 | 0.7217 | 0.9720 | 0.9621 | 0.8669 | 0.8947 | 0.8799 |
| 663 | 0.8942 | 0.9250 | 0.9805 | 0.8283 | 0.7179 | 0.9720 | 0.9621 | 0.8669 | 0.8940 | 0.8791 |
| 664 | 0.8942 | 0.9250 | 0.9805 | 0.8283 | 0.7217 | 0.9720 | 0.9621 | 0.8669 | 0.8944 | 0.8797 |
| 665 | 0.8942 | 0.9250 | 0.9785 | 0.8265 | 0.7198 | 0.9738 | 0.9621 | 0.8669 | 0.8940 | 0.8791 |
| 666 | 0.8924 | 0.9250 | 0.9785 | 0.8301 | 0.7217 | 0.9738 | 0.9621 | 0.8669 | 0.8944 | 0.8797 |
| 667 | 0.8924 | 0.9250 | 0.9785 | 0.8283 | 0.7217 | 0.9738 | 0.9621 | 0.8651 | 0.8940 | 0.8791 |
| 668 | 0.8924 | 0.9250 | 0.9805 | 0.8283 | 0.7217 | 0.9720 | 0.9621 | 0.8651 | 0.8940 | 0.8791 |
| 669 | 0.8907 | 0.9250 | 0.9805 | 0.8301 | 0.7217 | 0.9720 | 0.9621 | 0.8651 | 0.8940 | 0.8791 |
| 670 | 0.8907 | 0.9250 | 0.9805 | 0.8283 | 0.7236 | 0.9720 | 0.9621 | 0.8634 | 0.8938 | 0.8789 |
| 671 | 0.8907 | 0.9250 | 0.9805 | 0.8301 | 0.7236 | 0.9738 | 0.9621 | 0.8634 | 0.8942 | 0.8794 |
| 672 | 0.8907 | 0.9250 | 0.9805 | 0.8301 | 0.7236 | 0.9738 | 0.9621 | 0.8616 | 0.8940 | 0.8791 |
| 673 | 0.8907 | 0.9250 | 0.9805 | 0.8301 | 0.7217 | 0.9738 | 0.9621 | 0.8616 | 0.8938 | 0.8789 |
| 674 | 0.8889 | 0.9250 | 0.9824 | 0.8318 | 0.7179 | 0.9738 | 0.9621 | 0.8634 | 0.8938 | 0.8789 |
| 675 | 0.8889 | 0.9250 | 0.9824 | 0.8301 | 0.7159 | 0.9738 | 0.9621 | 0.8616 | 0.8931 | 0.8781 |
| 676 | 0.8889 | 0.9250 | 0.9805 | 0.8318 | 0.7159 | 0.9720 | 0.9621 | 0.8616 | 0.8928 | 0.8779 |
| 677 | 0.8907 | 0.9250 | 0.9805 | 0.8318 | 0.7140 | 0.9720 | 0.9621 | 0.8616 | 0.8928 | 0.8779 |
| 678 | 0.8907 | 0.9250 | 0.9805 | 0.8301 | 0.7140 | 0.9738 | 0.9621 | 0.8634 | 0.8931 | 0.8781 |
| 679 | 0.8924 | 0.9250 | 0.9805 | 0.8336 | 0.7179 | 0.9720 | 0.9621 | 0.8634 | 0.8940 | 0.8792 |
| 680 | 0.8871 | 0.9250 | 0.9785 | 0.8336 | 0.7179 | 0.9720 | 0.9621 | 0.8634 | 0.8931 | 0.8781 |
| 681 | 0.8889 | 0.9250 | 0.9785 | 0.8336 | 0.7179 | 0.9720 | 0.9621 | 0.8634 | 0.8933 | 0.8784 |
| 682 | 0.8889 | 0.9250 | 0.9785 | 0.8336 | 0.7198 | 0.9738 | 0.9639 | 0.8634 | 0.8940 | 0.8791 |
| 683 | 0.8889 | 0.9250 | 0.9785 | 0.8372 | 0.7217 | 0.9738 | 0.9639 | 0.8651 | 0.8949 | 0.8802 |
| 684 | 0.8889 | 0.9250 | 0.9785 | 0.8408 | 0.7217 | 0.9738 | 0.9656 | 0.8669 | 0.8958 | 0.8812 |
| 685 | 0.8889 | 0.9250 | 0.9805 | 0.8390 | 0.7236 | 0.9738 | 0.9656 | 0.8669 | 0.8960 | 0.8814 |
| 686 | 0.8889 | 0.9250 | 0.9805 | 0.8408 | 0.7179 | 0.9738 | 0.9639 | 0.8669 | 0.8953 | 0.8807 |
| 687 | 0.8889 | 0.9250 | 0.9805 | 0.8390 | 0.7159 | 0.9738 | 0.9639 | 0.8634 | 0.8944 | 0.8797 |
| 688 | 0.8889 | 0.9250 | 0.9805 | 0.8390 | 0.7159 | 0.9738 | 0.9639 | 0.8634 | 0.8944 | 0.8797 |
| 689 | 0.8871 | 0.9250 | 0.9805 | 0.8390 | 0.7198 | 0.9738 | 0.9639 | 0.8634 | 0.8947 | 0.8799 |
| 690 | 0.8836 | 0.9250 | 0.9805 | 0.8372 | 0.7217 | 0.9738 | 0.9639 | 0.8634 | 0.8942 | 0.8794 |
| 691 | 0.8836 | 0.9250 | 0.9805 | 0.8390 | 0.7217 | 0.9738 | 0.9639 | 0.8634 | 0.8944 | 0.8796 |
| 692 | 0.8854 | 0.9250 | 0.9805 | 0.8390 | 0.7159 | 0.9738 | 0.9639 | 0.8616 | 0.8938 | 0.8789 |
| 693 | 0.8818 | 0.9250 | 0.9805 | 0.8444 | 0.7159 | 0.9738 | 0.9639 | 0.8616 | 0.8940 | 0.8791 |
| 694 | 0.8854 | 0.9250 | 0.9805 | 0.8408 | 0.7140 | 0.9720 | 0.9621 | 0.8616 | 0.8933 | 0.8784 |
| 695 | 0.8871 | 0.9250 | 0.9805 | 0.8372 | 0.7159 | 0.9720 | 0.9639 | 0.8599 | 0.8933 | 0.8784 |
| 696 | 0.8854 | 0.9250 | 0.9805 | 0.8372 | 0.7140 | 0.9720 | 0.9639 | 0.8581 | 0.8926 | 0.8776 |
| 697 | 0.8871 | 0.9250 | 0.9805 | 0.8283 | 0.7044 | 0.9720 | 0.9639 | 0.8581 | 0.8906 | 0.8753 |
| 698 | 0.8854 | 0.9250 | 0.9805 | 0.8283 | 0.7044 | 0.9720 | 0.9639 | 0.8581 | 0.8904 | 0.8750 |
| 699 | 0.8854 | 0.9250 | 0.9805 | 0.8283 | 0.7025 | 0.9720 | 0.9639 | 0.8564 | 0.8899 | 0.8745 |
| 700 | 0.8854 | 0.9250 | 0.9805 | 0.8318 | 0.7025 | 0.9701 | 0.9621 | 0.8581 | 0.8901 | 0.8748 |
| 701 | 0.8854 | 0.9250 | 0.9805 | 0.8318 | 0.7025 | 0.9701 | 0.9621 | 0.8581 | 0.8901 | 0.8748 |
| 702 | 0.8854 | 0.9250 | 0.9805 | 0.8336 | 0.7025 | 0.9701 | 0.9621 | 0.8581 | 0.8904 | 0.8751 |
| 703 | 0.8854 | 0.9250 | 0.9805 | 0.8372 | 0.7006 | 0.9701 | 0.9621 | 0.8599 | 0.8908 | 0.8756 |
| 704 | 0.8871 | 0.9250 | 0.9805 | 0.8390 | 0.7044 | 0.9701 | 0.9621 | 0.8599 | 0.8917 | 0.8766 |
| 705 | 0.8854 | 0.9267 | 0.9805 | 0.8390 | 0.7025 | 0.9701 | 0.9639 | 0.8634 | 0.8922 | 0.8771 |
| 706 | 0.8854 | 0.9267 | 0.9805 | 0.8426 | 0.7025 | 0.9701 | 0.9621 | 0.8599 | 0.8919 | 0.8769 |
| 707 | 0.8854 | 0.9267 | 0.9805 | 0.8426 | 0.7025 | 0.9701 | 0.9639 | 0.8581 | 0.8919 | 0.8769 |
| 708 | 0.8871 | 0.9267 | 0.9805 | 0.8444 | 0.7025 | 0.9701 | 0.9621 | 0.8599 | 0.8924 | 0.8774 |
| 709 | 0.8818 | 0.9267 | 0.9805 | 0.8479 | 0.7044 | 0.9701 | 0.9621 | 0.8616 | 0.8926 | 0.8776 |
| 710 | 0.8818 | 0.9267 | 0.9805 | 0.8462 | 0.7063 | 0.9701 | 0.9621 | 0.8599 | 0.8924 | 0.8774 |
| 711 | 0.8836 | 0.9267 | 0.9805 | 0.8462 | 0.7044 | 0.9701 | 0.9621 | 0.8599 | 0.8924 | 0.8774 |
| 712 | 0.8818 | 0.9267 | 0.9805 | 0.8462 | 0.7044 | 0.9701 | 0.9621 | 0.8599 | 0.8922 | 0.8771 |
| 713 | 0.8818 | 0.9267 | 0.9805 | 0.8462 | 0.7063 | 0.9701 | 0.9621 | 0.8599 | 0.8924 | 0.8774 |
| 714 | 0.8854 | 0.9267 | 0.9805 | 0.8444 | 0.7063 | 0.9720 | 0.9621 | 0.8599 | 0.8928 | 0.8779 |
| 715 | 0.8818 | 0.9267 | 0.9805 | 0.8444 | 0.7063 | 0.9720 | 0.9621 | 0.8581 | 0.8922 | 0.8771 |
| 716 | 0.8801 | 0.9267 | 0.9805 | 0.8426 | 0.7083 | 0.9738 | 0.9604 | 0.8581 | 0.8919 | 0.8769 |
| 717 | 0.8783 | 0.9267 | 0.9805 | 0.8426 | 0.7102 | 0.9738 | 0.9604 | 0.8564 | 0.8917 | 0.8766 |
| 718 | 0.8783 | 0.9285 | 0.9805 | 0.8390 | 0.7121 | 0.9738 | 0.9621 | 0.8564 | 0.8919 | 0.8768 |
| 719 | 0.8783 | 0.9267 | 0.9805 | 0.8372 | 0.7083 | 0.9738 | 0.9604 | 0.8581 | 0.8910 | 0.8758 |
| 720 | 0.8765 | 0.9302 | 0.9805 | 0.8372 | 0.7121 | 0.9738 | 0.9621 | 0.8581 | 0.8919 | 0.8768 |
| 721 | 0.8765 | 0.9302 | 0.9805 | 0.8372 | 0.7083 | 0.9738 | 0.9621 | 0.8599 | 0.8917 | 0.8766 |
| 722 | 0.8748 | 0.9302 | 0.9805 | 0.8390 | 0.7159 | 0.9738 | 0.9621 | 0.8616 | 0.8928 | 0.8779 |
| 723 | 0.8783 | 0.9302 | 0.9805 | 0.8390 | 0.7198 | 0.9738 | 0.9621 | 0.8616 | 0.8938 | 0.8789 |
| 724 | 0.8836 | 0.9302 | 0.9805 | 0.8390 | 0.7179 | 0.9720 | 0.9656 | 0.8616 | 0.8944 | 0.8797 |
| 725 | 0.8765 | 0.9285 | 0.9805 | 0.8318 | 0.7140 | 0.9720 | 0.9673 | 0.8581 | 0.8917 | 0.8765 |
| 726 | 0.8801 | 0.9285 | 0.9805 | 0.8318 | 0.7140 | 0.9720 | 0.9673 | 0.8581 | 0.8922 | 0.8771 |
| 727 | 0.8783 | 0.9267 | 0.9805 | 0.8301 | 0.7217 | 0.9720 | 0.9673 | 0.8599 | 0.8926 | 0.8776 |
| 728 | 0.8783 | 0.9267 | 0.9805 | 0.8336 | 0.7179 | 0.9757 | 0.9673 | 0.8599 | 0.8931 | 0.8781 |
| 729 | 0.8765 | 0.9267 | 0.9805 | 0.8336 | 0.7179 | 0.9757 | 0.9673 | 0.8599 | 0.8928 | 0.8778 |
| 730 | 0.8783 | 0.9267 | 0.9805 | 0.8372 | 0.7159 | 0.9757 | 0.9673 | 0.8616 | 0.8935 | 0.8786 |
| 731 | 0.8765 | 0.9267 | 0.9805 | 0.8336 | 0.7179 | 0.9757 | 0.9673 | 0.8599 | 0.8928 | 0.8778 |
| 732 | 0.8783 | 0.9267 | 0.9805 | 0.8318 | 0.7198 | 0.9757 | 0.9673 | 0.8599 | 0.8931 | 0.8781 |
| 733 | 0.8765 | 0.9267 | 0.9805 | 0.8354 | 0.7140 | 0.9757 | 0.9673 | 0.8616 | 0.8928 | 0.8778 |
| 734 | 0.8765 | 0.9267 | 0.9805 | 0.8354 | 0.7140 | 0.9757 | 0.9673 | 0.8599 | 0.8926 | 0.8776 |
| 735 | 0.8748 | 0.9267 | 0.9805 | 0.8354 | 0.7159 | 0.9757 | 0.9673 | 0.8599 | 0.8926 | 0.8776 |
| 736 | 0.8783 | 0.9285 | 0.9805 | 0.8354 | 0.7179 | 0.9757 | 0.9673 | 0.8616 | 0.8938 | 0.8789 |
| 737 | 0.8783 | 0.9285 | 0.9805 | 0.8336 | 0.7159 | 0.9757 | 0.9673 | 0.8616 | 0.8933 | 0.8784 |
| 738 | 0.8765 | 0.9285 | 0.9805 | 0.8318 | 0.7159 | 0.9757 | 0.9673 | 0.8634 | 0.8931 | 0.8781 |
| 739 | 0.8730 | 0.9285 | 0.9805 | 0.8301 | 0.7121 | 0.9757 | 0.9673 | 0.8564 | 0.8910 | 0.8758 |
| 740 | 0.8765 | 0.9285 | 0.9805 | 0.8336 | 0.7121 | 0.9757 | 0.9673 | 0.8546 | 0.8917 | 0.8766 |
| 741 | 0.8765 | 0.9285 | 0.9805 | 0.8318 | 0.7140 | 0.9757 | 0.9673 | 0.8529 | 0.8915 | 0.8763 |
| 742 | 0.8765 | 0.9285 | 0.9805 | 0.8336 | 0.7121 | 0.9757 | 0.9673 | 0.8546 | 0.8917 | 0.8766 |
| 743 | 0.8801 | 0.9285 | 0.9805 | 0.8336 | 0.7102 | 0.9757 | 0.9673 | 0.8546 | 0.8919 | 0.8768 |
| 744 | 0.8801 | 0.9285 | 0.9805 | 0.8336 | 0.7083 | 0.9757 | 0.9656 | 0.8546 | 0.8915 | 0.8763 |
| 745 | 0.8801 | 0.9285 | 0.9805 | 0.8301 | 0.7063 | 0.9757 | 0.9656 | 0.8564 | 0.8910 | 0.8758 |
| 746 | 0.8801 | 0.9285 | 0.9805 | 0.8301 | 0.7044 | 0.9757 | 0.9656 | 0.8546 | 0.8906 | 0.8753 |
| 747 | 0.8836 | 0.9285 | 0.9805 | 0.8283 | 0.7083 | 0.9757 | 0.9656 | 0.8546 | 0.8913 | 0.8761 |
| 748 | 0.8783 | 0.9285 | 0.9805 | 0.8211 | 0.7063 | 0.9757 | 0.9656 | 0.8546 | 0.8895 | 0.8740 |
| 749 | 0.8783 | 0.9285 | 0.9824 | 0.8211 | 0.7083 | 0.9757 | 0.9656 | 0.8564 | 0.8901 | 0.8748 |
| 750 | 0.8783 | 0.9285 | 0.9824 | 0.8265 | 0.7063 | 0.9757 | 0.9656 | 0.8564 | 0.8906 | 0.8753 |
| 751 | 0.8783 | 0.9285 | 0.9805 | 0.8283 | 0.7083 | 0.9757 | 0.9656 | 0.8564 | 0.8908 | 0.8756 |
| 752 | 0.8783 | 0.9285 | 0.9805 | 0.8265 | 0.7063 | 0.9757 | 0.9656 | 0.8616 | 0.8910 | 0.8758 |
| 753 | 0.8783 | 0.9285 | 0.9805 | 0.8265 | 0.7044 | 0.9757 | 0.9656 | 0.8634 | 0.8910 | 0.8758 |
| 754 | 0.8765 | 0.9285 | 0.9805 | 0.8229 | 0.7063 | 0.9757 | 0.9656 | 0.8616 | 0.8904 | 0.8750 |
| 755 | 0.8748 | 0.9285 | 0.9805 | 0.8247 | 0.7063 | 0.9757 | 0.9656 | 0.8616 | 0.8904 | 0.8750 |
| 756 | 0.8765 | 0.9285 | 0.9805 | 0.8265 | 0.7044 | 0.9757 | 0.9656 | 0.8616 | 0.8906 | 0.8753 |
| 757 | 0.8783 | 0.9285 | 0.9805 | 0.8265 | 0.7025 | 0.9757 | 0.9656 | 0.8581 | 0.8901 | 0.8748 |
| 758 | 0.8783 | 0.9285 | 0.9805 | 0.8247 | 0.7025 | 0.9757 | 0.9656 | 0.8581 | 0.8899 | 0.8745 |
| 759 | 0.8765 | 0.9285 | 0.9805 | 0.8247 | 0.7025 | 0.9757 | 0.9656 | 0.8581 | 0.8897 | 0.8743 |
| 760 | 0.8765 | 0.9285 | 0.9805 | 0.8265 | 0.7006 | 0.9757 | 0.9656 | 0.8599 | 0.8899 | 0.8745 |
| 761 | 0.8765 | 0.9285 | 0.9805 | 0.8265 | 0.6987 | 0.9757 | 0.9656 | 0.8599 | 0.8897 | 0.8743 |
| 762 | 0.8748 | 0.9267 | 0.9805 | 0.8265 | 0.6948 | 0.9757 | 0.9656 | 0.8581 | 0.8886 | 0.8730 |
| 763 | 0.8765 | 0.9267 | 0.9805 | 0.8247 | 0.6929 | 0.9757 | 0.9656 | 0.8581 | 0.8883 | 0.8727 |
| 764 | 0.8765 | 0.9267 | 0.9805 | 0.8265 | 0.6929 | 0.9757 | 0.9656 | 0.8546 | 0.8881 | 0.8725 |
| 765 | 0.8765 | 0.9267 | 0.9805 | 0.8247 | 0.6910 | 0.9757 | 0.9656 | 0.8546 | 0.8877 | 0.8720 |
| 766 | 0.8748 | 0.9267 | 0.9805 | 0.8283 | 0.6948 | 0.9757 | 0.9656 | 0.8581 | 0.8888 | 0.8732 |
| 767 | 0.8748 | 0.9267 | 0.9805 | 0.8265 | 0.6948 | 0.9757 | 0.9656 | 0.8546 | 0.8881 | 0.8725 |
| 768 | 0.8748 | 0.9267 | 0.9805 | 0.8283 | 0.6948 | 0.9757 | 0.9656 | 0.8546 | 0.8883 | 0.8727 |
| 769 | 0.8748 | 0.9267 | 0.9805 | 0.8265 | 0.6987 | 0.9757 | 0.9656 | 0.8529 | 0.8883 | 0.8727 |
| 770 | 0.8748 | 0.9267 | 0.9805 | 0.8265 | 0.6987 | 0.9757 | 0.9656 | 0.8529 | 0.8883 | 0.8727 |
| 771 | 0.8748 | 0.9267 | 0.9805 | 0.8265 | 0.6967 | 0.9757 | 0.9656 | 0.8529 | 0.8881 | 0.8725 |
| 772 | 0.8748 | 0.9267 | 0.9805 | 0.8265 | 0.6948 | 0.9757 | 0.9656 | 0.8494 | 0.8874 | 0.8717 |
| 773 | 0.8748 | 0.9267 | 0.9805 | 0.8265 | 0.6967 | 0.9757 | 0.9656 | 0.8511 | 0.8879 | 0.8722 |
| 774 | 0.8748 | 0.9267 | 0.9805 | 0.8265 | 0.6948 | 0.9757 | 0.9639 | 0.8511 | 0.8874 | 0.8717 |
| 775 | 0.8748 | 0.9267 | 0.9805 | 0.8265 | 0.6948 | 0.9757 | 0.9639 | 0.8529 | 0.8877 | 0.8720 |
| 776 | 0.8748 | 0.9267 | 0.9805 | 0.8283 | 0.6948 | 0.9757 | 0.9656 | 0.8546 | 0.8883 | 0.8727 |
| 777 | 0.8783 | 0.9267 | 0.9805 | 0.8301 | 0.6948 | 0.9757 | 0.9656 | 0.8529 | 0.8888 | 0.8732 |
| 778 | 0.8783 | 0.9267 | 0.9805 | 0.8265 | 0.6929 | 0.9757 | 0.9656 | 0.8529 | 0.8881 | 0.8725 |
| 779 | 0.8783 | 0.9267 | 0.9805 | 0.8247 | 0.6967 | 0.9757 | 0.9656 | 0.8546 | 0.8886 | 0.8730 |
| 780 | 0.8783 | 0.9267 | 0.9785 | 0.8247 | 0.6948 | 0.9757 | 0.9656 | 0.8546 | 0.8881 | 0.8725 |
| 781 | 0.8783 | 0.9267 | 0.9785 | 0.8247 | 0.6948 | 0.9757 | 0.9656 | 0.8529 | 0.8879 | 0.8722 |
| 782 | 0.8783 | 0.9267 | 0.9785 | 0.8247 | 0.6948 | 0.9757 | 0.9656 | 0.8529 | 0.8879 | 0.8722 |
| 783 | 0.8783 | 0.9267 | 0.9785 | 0.8247 | 0.6967 | 0.9757 | 0.9656 | 0.8529 | 0.8881 | 0.8725 |
| 784 | 0.8765 | 0.9267 | 0.9785 | 0.8247 | 0.6948 | 0.9757 | 0.9656 | 0.8511 | 0.8874 | 0.8717 |
| 785 | 0.8765 | 0.9267 | 0.9785 | 0.8265 | 0.6929 | 0.9757 | 0.9656 | 0.8511 | 0.8874 | 0.8717 |
| 786 | 0.8730 | 0.9267 | 0.9785 | 0.8265 | 0.6910 | 0.9757 | 0.9656 | 0.8511 | 0.8868 | 0.8709 |
| 787 | 0.8695 | 0.9267 | 0.9785 | 0.8301 | 0.6910 | 0.9757 | 0.9656 | 0.8511 | 0.8868 | 0.8709 |
| 788 | 0.8695 | 0.9267 | 0.9785 | 0.8301 | 0.6891 | 0.9757 | 0.9656 | 0.8546 | 0.8870 | 0.8712 |
| 789 | 0.8765 | 0.9267 | 0.9785 | 0.8301 | 0.6871 | 0.9757 | 0.9656 | 0.8546 | 0.8877 | 0.8720 |
| 790 | 0.8765 | 0.9267 | 0.9785 | 0.8318 | 0.6871 | 0.9757 | 0.9656 | 0.8546 | 0.8879 | 0.8722 |
| 791 | 0.8765 | 0.9267 | 0.9785 | 0.8318 | 0.6871 | 0.9757 | 0.9656 | 0.8546 | 0.8879 | 0.8722 |
| 792 | 0.8748 | 0.9267 | 0.9785 | 0.8318 | 0.6852 | 0.9757 | 0.9656 | 0.8546 | 0.8874 | 0.8717 |
| 793 | 0.8748 | 0.9267 | 0.9785 | 0.8318 | 0.6891 | 0.9776 | 0.9639 | 0.8546 | 0.8879 | 0.8722 |
| 794 | 0.8730 | 0.9267 | 0.9785 | 0.8336 | 0.6929 | 0.9776 | 0.9639 | 0.8529 | 0.8881 | 0.8725 |
| 795 | 0.8765 | 0.9267 | 0.9785 | 0.8336 | 0.6891 | 0.9776 | 0.9639 | 0.8511 | 0.8879 | 0.8722 |
| 796 | 0.8765 | 0.9267 | 0.9785 | 0.8336 | 0.6891 | 0.9776 | 0.9639 | 0.8529 | 0.8881 | 0.8725 |
| 797 | 0.8748 | 0.9267 | 0.9785 | 0.8318 | 0.6891 | 0.9776 | 0.9639 | 0.8546 | 0.8879 | 0.8722 |
| 798 | 0.8748 | 0.9267 | 0.9785 | 0.8336 | 0.6871 | 0.9776 | 0.9639 | 0.8564 | 0.8881 | 0.8725 |
| 799 | 0.8748 | 0.9267 | 0.9785 | 0.8354 | 0.6929 | 0.9776 | 0.9639 | 0.8599 | 0.8895 | 0.8740 |
| 800 | 0.8765 | 0.9285 | 0.9785 | 0.8372 | 0.6967 | 0.9776 | 0.9639 | 0.8616 | 0.8908 | 0.8756 |
| 801 | 0.8765 | 0.9285 | 0.9785 | 0.8372 | 0.6948 | 0.9776 | 0.9639 | 0.8599 | 0.8904 | 0.8751 |
| 802 | 0.8748 | 0.9285 | 0.9785 | 0.8372 | 0.6929 | 0.9776 | 0.9639 | 0.8599 | 0.8899 | 0.8745 |
| 803 | 0.8713 | 0.9285 | 0.9785 | 0.8354 | 0.6910 | 0.9776 | 0.9639 | 0.8599 | 0.8890 | 0.8735 |
| 804 | 0.8730 | 0.9285 | 0.9785 | 0.8354 | 0.6929 | 0.9776 | 0.9656 | 0.8599 | 0.8897 | 0.8743 |
| 805 | 0.8695 | 0.9285 | 0.9785 | 0.8354 | 0.6948 | 0.9776 | 0.9656 | 0.8599 | 0.8895 | 0.8740 |
| 806 | 0.8695 | 0.9285 | 0.9785 | 0.8354 | 0.6929 | 0.9776 | 0.9656 | 0.8581 | 0.8890 | 0.8735 |
| 807 | 0.8695 | 0.9285 | 0.9785 | 0.8318 | 0.6929 | 0.9757 | 0.9656 | 0.8564 | 0.8881 | 0.8725 |
| 808 | 0.8713 | 0.9285 | 0.9785 | 0.8301 | 0.6929 | 0.9757 | 0.9656 | 0.8581 | 0.8883 | 0.8727 |
| 809 | 0.8695 | 0.9285 | 0.9785 | 0.8283 | 0.6871 | 0.9757 | 0.9656 | 0.8581 | 0.8872 | 0.8714 |
| 810 | 0.8677 | 0.9285 | 0.9785 | 0.8318 | 0.6891 | 0.9757 | 0.9656 | 0.8599 | 0.8879 | 0.8722 |
| 811 | 0.8695 | 0.9285 | 0.9785 | 0.8318 | 0.6891 | 0.9757 | 0.9656 | 0.8599 | 0.8881 | 0.8725 |
| 812 | 0.8660 | 0.9285 | 0.9785 | 0.8283 | 0.6929 | 0.9757 | 0.9656 | 0.8564 | 0.8872 | 0.8714 |
| 813 | 0.8642 | 0.9285 | 0.9785 | 0.8318 | 0.6929 | 0.9757 | 0.9656 | 0.8564 | 0.8874 | 0.8717 |
| 814 | 0.8642 | 0.9285 | 0.9785 | 0.8318 | 0.6948 | 0.9757 | 0.9656 | 0.8564 | 0.8877 | 0.8719 |
| 815 | 0.8677 | 0.9285 | 0.9785 | 0.8301 | 0.6929 | 0.9757 | 0.9656 | 0.8564 | 0.8877 | 0.8719 |
| 816 | 0.8695 | 0.9285 | 0.9785 | 0.8301 | 0.6891 | 0.9757 | 0.9639 | 0.8564 | 0.8872 | 0.8714 |
| 817 | 0.8713 | 0.9285 | 0.9785 | 0.8265 | 0.6891 | 0.9757 | 0.9639 | 0.8564 | 0.8870 | 0.8712 |
| 818 | 0.8713 | 0.9302 | 0.9785 | 0.8265 | 0.6891 | 0.9757 | 0.9639 | 0.8564 | 0.8872 | 0.8714 |
| 819 | 0.8713 | 0.9285 | 0.9785 | 0.8265 | 0.6871 | 0.9757 | 0.9639 | 0.8581 | 0.8870 | 0.8712 |
| 820 | 0.8713 | 0.9302 | 0.9785 | 0.8247 | 0.6891 | 0.9757 | 0.9639 | 0.8599 | 0.8874 | 0.8717 |
| 821 | 0.8677 | 0.9302 | 0.9785 | 0.8265 | 0.6929 | 0.9757 | 0.9639 | 0.8564 | 0.8872 | 0.8714 |
| 822 | 0.8677 | 0.9302 | 0.9785 | 0.8265 | 0.6910 | 0.9757 | 0.9639 | 0.8564 | 0.8870 | 0.8711 |
| 823 | 0.8677 | 0.9302 | 0.9785 | 0.8265 | 0.6891 | 0.9776 | 0.9639 | 0.8564 | 0.8870 | 0.8712 |
| 824 | 0.8677 | 0.9302 | 0.9785 | 0.8283 | 0.6871 | 0.9757 | 0.9639 | 0.8581 | 0.8870 | 0.8712 |
| 825 | 0.8660 | 0.9302 | 0.9785 | 0.8283 | 0.6871 | 0.9757 | 0.9639 | 0.8581 | 0.8868 | 0.8709 |
| 826 | 0.8660 | 0.9302 | 0.9785 | 0.8301 | 0.6891 | 0.9757 | 0.9639 | 0.8581 | 0.8872 | 0.8714 |
| 827 | 0.8607 | 0.9302 | 0.9785 | 0.8283 | 0.6891 | 0.9757 | 0.9639 | 0.8599 | 0.8865 | 0.8706 |
| 828 | 0.8624 | 0.9302 | 0.9785 | 0.8265 | 0.6891 | 0.9757 | 0.9639 | 0.8581 | 0.8863 | 0.8704 |
| 829 | 0.8624 | 0.9302 | 0.9785 | 0.8265 | 0.6871 | 0.9757 | 0.9639 | 0.8581 | 0.8861 | 0.8701 |
| 830 | 0.8607 | 0.9302 | 0.9785 | 0.8265 | 0.6871 | 0.9757 | 0.9639 | 0.8564 | 0.8856 | 0.8696 |
| 831 | 0.8624 | 0.9302 | 0.9785 | 0.8247 | 0.6891 | 0.9757 | 0.9639 | 0.8529 | 0.8854 | 0.8693 |
| 832 | 0.8607 | 0.9302 | 0.9785 | 0.8265 | 0.6910 | 0.9757 | 0.9656 | 0.8529 | 0.8859 | 0.8698 |
| 833 | 0.8607 | 0.9302 | 0.9785 | 0.8265 | 0.6891 | 0.9757 | 0.9639 | 0.8529 | 0.8854 | 0.8693 |
| 834 | 0.8607 | 0.9302 | 0.9785 | 0.8247 | 0.6871 | 0.9757 | 0.9639 | 0.8546 | 0.8852 | 0.8691 |
| 835 | 0.8589 | 0.9302 | 0.9785 | 0.8247 | 0.6852 | 0.9757 | 0.9639 | 0.8546 | 0.8847 | 0.8686 |
| 836 | 0.8624 | 0.9302 | 0.9785 | 0.8265 | 0.6775 | 0.9757 | 0.9639 | 0.8529 | 0.8843 | 0.8681 |
| 837 | 0.8624 | 0.9302 | 0.9785 | 0.8265 | 0.6814 | 0.9757 | 0.9639 | 0.8511 | 0.8845 | 0.8683 |
| 838 | 0.8607 | 0.9302 | 0.9785 | 0.8247 | 0.6814 | 0.9776 | 0.9639 | 0.8511 | 0.8843 | 0.8681 |
| 839 | 0.8624 | 0.9302 | 0.9785 | 0.8247 | 0.6833 | 0.9776 | 0.9621 | 0.8511 | 0.8845 | 0.8683 |
| 840 | 0.8624 | 0.9302 | 0.9785 | 0.8229 | 0.6814 | 0.9776 | 0.9621 | 0.8511 | 0.8841 | 0.8678 |
| 841 | 0.8660 | 0.9302 | 0.9785 | 0.8265 | 0.6795 | 0.9757 | 0.9639 | 0.8511 | 0.8847 | 0.8686 |
| 842 | 0.8677 | 0.9302 | 0.9785 | 0.8265 | 0.6795 | 0.9757 | 0.9639 | 0.8494 | 0.8847 | 0.8686 |
| 843 | 0.8660 | 0.9319 | 0.9785 | 0.8301 | 0.6852 | 0.9757 | 0.9639 | 0.8494 | 0.8859 | 0.8699 |
| 844 | 0.8660 | 0.9319 | 0.9785 | 0.8301 | 0.6833 | 0.9757 | 0.9639 | 0.8494 | 0.8856 | 0.8696 |
| 845 | 0.8660 | 0.9319 | 0.9785 | 0.8283 | 0.6795 | 0.9757 | 0.9639 | 0.8494 | 0.8850 | 0.8689 |
| 846 | 0.8642 | 0.9319 | 0.9785 | 0.8265 | 0.6775 | 0.9757 | 0.9639 | 0.8494 | 0.8843 | 0.8681 |
| 847 | 0.8607 | 0.9319 | 0.9785 | 0.8283 | 0.6756 | 0.9757 | 0.9639 | 0.8494 | 0.8838 | 0.8676 |
| 848 | 0.8607 | 0.9319 | 0.9785 | 0.8247 | 0.6833 | 0.9757 | 0.9639 | 0.8494 | 0.8843 | 0.8681 |
| 849 | 0.8624 | 0.9319 | 0.9785 | 0.8247 | 0.6814 | 0.9757 | 0.9639 | 0.8476 | 0.8841 | 0.8678 |
| 850 | 0.8624 | 0.9319 | 0.9785 | 0.8229 | 0.6775 | 0.9757 | 0.9639 | 0.8476 | 0.8834 | 0.8671 |
| 851 | 0.8642 | 0.9319 | 0.9785 | 0.8229 | 0.6795 | 0.9757 | 0.9639 | 0.8494 | 0.8841 | 0.8678 |
| 852 | 0.8660 | 0.9319 | 0.9785 | 0.8193 | 0.6775 | 0.9757 | 0.9639 | 0.8494 | 0.8836 | 0.8673 |
| 853 | 0.8589 | 0.9319 | 0.9785 | 0.8211 | 0.6814 | 0.9757 | 0.9639 | 0.8511 | 0.8836 | 0.8673 |
| 854 | 0.8607 | 0.9319 | 0.9785 | 0.8175 | 0.6852 | 0.9757 | 0.9639 | 0.8494 | 0.8836 | 0.8673 |
| 855 | 0.8607 | 0.9319 | 0.9785 | 0.8247 | 0.6852 | 0.9738 | 0.9639 | 0.8511 | 0.8845 | 0.8683 |
| 856 | 0.8642 | 0.9319 | 0.9785 | 0.8229 | 0.6852 | 0.9738 | 0.9639 | 0.8511 | 0.8847 | 0.8686 |
| 857 | 0.8624 | 0.9319 | 0.9785 | 0.8211 | 0.6852 | 0.9738 | 0.9639 | 0.8494 | 0.8841 | 0.8678 |
| 858 | 0.8642 | 0.9319 | 0.9785 | 0.8211 | 0.6852 | 0.9738 | 0.9639 | 0.8511 | 0.8845 | 0.8683 |
| 859 | 0.8677 | 0.9319 | 0.9785 | 0.8211 | 0.6891 | 0.9738 | 0.9656 | 0.8511 | 0.8856 | 0.8696 |
| 860 | 0.8677 | 0.9319 | 0.9785 | 0.8229 | 0.6891 | 0.9738 | 0.9639 | 0.8529 | 0.8859 | 0.8699 |
| 861 | 0.8677 | 0.9319 | 0.9785 | 0.8211 | 0.6891 | 0.9738 | 0.9639 | 0.8529 | 0.8856 | 0.8696 |
| 862 | 0.8677 | 0.9319 | 0.9785 | 0.8229 | 0.6871 | 0.9738 | 0.9639 | 0.8511 | 0.8854 | 0.8694 |
| 863 | 0.8677 | 0.9319 | 0.9805 | 0.8247 | 0.6891 | 0.9738 | 0.9639 | 0.8476 | 0.8856 | 0.8696 |
| 864 | 0.8677 | 0.9319 | 0.9805 | 0.8247 | 0.6871 | 0.9757 | 0.9639 | 0.8511 | 0.8861 | 0.8701 |
| 865 | 0.8660 | 0.9319 | 0.9805 | 0.8229 | 0.6871 | 0.9757 | 0.9621 | 0.8494 | 0.8852 | 0.8691 |
| 866 | 0.8660 | 0.9319 | 0.9805 | 0.8229 | 0.6910 | 0.9757 | 0.9621 | 0.8494 | 0.8856 | 0.8696 |
| 867 | 0.8660 | 0.9319 | 0.9805 | 0.8193 | 0.6910 | 0.9757 | 0.9621 | 0.8511 | 0.8854 | 0.8693 |
| 868 | 0.8677 | 0.9302 | 0.9805 | 0.8211 | 0.6891 | 0.9757 | 0.9621 | 0.8511 | 0.8854 | 0.8694 |
| 869 | 0.8660 | 0.9302 | 0.9805 | 0.8211 | 0.6910 | 0.9757 | 0.9621 | 0.8529 | 0.8856 | 0.8696 |
| 870 | 0.8660 | 0.9319 | 0.9805 | 0.8211 | 0.6910 | 0.9757 | 0.9621 | 0.8511 | 0.8856 | 0.8696 |
| 871 | 0.8642 | 0.9302 | 0.9805 | 0.8211 | 0.6891 | 0.9757 | 0.9639 | 0.8511 | 0.8852 | 0.8691 |
| 872 | 0.8642 | 0.9302 | 0.9805 | 0.8175 | 0.6891 | 0.9757 | 0.9639 | 0.8494 | 0.8845 | 0.8683 |
| 873 | 0.8642 | 0.9319 | 0.9805 | 0.8229 | 0.6910 | 0.9757 | 0.9621 | 0.8494 | 0.8854 | 0.8694 |
| 874 | 0.8642 | 0.9319 | 0.9805 | 0.8229 | 0.6929 | 0.9757 | 0.9621 | 0.8511 | 0.8859 | 0.8699 |
| 875 | 0.8695 | 0.9319 | 0.9805 | 0.8247 | 0.6929 | 0.9757 | 0.9621 | 0.8494 | 0.8865 | 0.8706 |
| 876 | 0.8695 | 0.9319 | 0.9805 | 0.8229 | 0.6910 | 0.9757 | 0.9621 | 0.8494 | 0.8861 | 0.8701 |
| 877 | 0.8695 | 0.9319 | 0.9805 | 0.8247 | 0.6948 | 0.9738 | 0.9621 | 0.8494 | 0.8865 | 0.8706 |
| 878 | 0.8677 | 0.9319 | 0.9805 | 0.8265 | 0.6948 | 0.9738 | 0.9621 | 0.8476 | 0.8863 | 0.8704 |
| 879 | 0.8660 | 0.9319 | 0.9805 | 0.8247 | 0.6948 | 0.9738 | 0.9621 | 0.8476 | 0.8859 | 0.8699 |
| 880 | 0.8660 | 0.9302 | 0.9805 | 0.8211 | 0.6948 | 0.9757 | 0.9639 | 0.8476 | 0.8856 | 0.8696 |
| 881 | 0.8660 | 0.9302 | 0.9805 | 0.8211 | 0.6948 | 0.9738 | 0.9639 | 0.8476 | 0.8854 | 0.8693 |
| 882 | 0.8677 | 0.9302 | 0.9805 | 0.8211 | 0.6929 | 0.9738 | 0.9639 | 0.8476 | 0.8854 | 0.8693 |
| 883 | 0.8677 | 0.9302 | 0.9805 | 0.8247 | 0.6929 | 0.9738 | 0.9639 | 0.8476 | 0.8859 | 0.8699 |
| 884 | 0.8677 | 0.9302 | 0.9805 | 0.8229 | 0.6948 | 0.9738 | 0.9639 | 0.8494 | 0.8861 | 0.8701 |
| 885 | 0.8642 | 0.9285 | 0.9805 | 0.8229 | 0.6929 | 0.9738 | 0.9639 | 0.8494 | 0.8852 | 0.8691 |
| 886 | 0.8660 | 0.9285 | 0.9805 | 0.8229 | 0.6910 | 0.9738 | 0.9639 | 0.8476 | 0.8850 | 0.8688 |
| 887 | 0.8677 | 0.9285 | 0.9805 | 0.8229 | 0.6891 | 0.9738 | 0.9639 | 0.8476 | 0.8850 | 0.8688 |
| 888 | 0.8660 | 0.9285 | 0.9805 | 0.8247 | 0.6852 | 0.9738 | 0.9639 | 0.8476 | 0.8845 | 0.8683 |
| 889 | 0.8660 | 0.9285 | 0.9785 | 0.8265 | 0.6891 | 0.9738 | 0.9639 | 0.8476 | 0.8850 | 0.8688 |
| 890 | 0.8660 | 0.9302 | 0.9785 | 0.8229 | 0.6891 | 0.9738 | 0.9639 | 0.8476 | 0.8847 | 0.8686 |
| 891 | 0.8660 | 0.9285 | 0.9785 | 0.8247 | 0.6891 | 0.9738 | 0.9639 | 0.8476 | 0.8847 | 0.8686 |
| 892 | 0.8660 | 0.9285 | 0.9785 | 0.8229 | 0.6891 | 0.9738 | 0.9639 | 0.8476 | 0.8845 | 0.8683 |
| 893 | 0.8642 | 0.9267 | 0.9785 | 0.8265 | 0.6910 | 0.9738 | 0.9639 | 0.8476 | 0.8847 | 0.8686 |
| 894 | 0.8642 | 0.9267 | 0.9785 | 0.8229 | 0.6948 | 0.9738 | 0.9639 | 0.8441 | 0.8843 | 0.8681 |
| 895 | 0.8642 | 0.9285 | 0.9766 | 0.8265 | 0.6948 | 0.9738 | 0.9639 | 0.8476 | 0.8852 | 0.8691 |
| 896 | 0.8624 | 0.9285 | 0.9766 | 0.8229 | 0.6948 | 0.9738 | 0.9639 | 0.8494 | 0.8847 | 0.8686 |
| 897 | 0.8642 | 0.9267 | 0.9766 | 0.8229 | 0.6948 | 0.9738 | 0.9639 | 0.8511 | 0.8850 | 0.8688 |
| 898 | 0.8642 | 0.9285 | 0.9766 | 0.8229 | 0.6967 | 0.9738 | 0.9639 | 0.8511 | 0.8854 | 0.8693 |
| 899 | 0.8624 | 0.9285 | 0.9766 | 0.8229 | 0.6967 | 0.9738 | 0.9639 | 0.8494 | 0.8850 | 0.8688 |
| 900 | 0.8642 | 0.9285 | 0.9766 | 0.8247 | 0.6891 | 0.9738 | 0.9639 | 0.8511 | 0.8847 | 0.8685 |
| 901 | 0.8624 | 0.9267 | 0.9766 | 0.8229 | 0.6910 | 0.9738 | 0.9639 | 0.8511 | 0.8843 | 0.8680 |
| 902 | 0.8624 | 0.9267 | 0.9766 | 0.8229 | 0.6910 | 0.9738 | 0.9639 | 0.8511 | 0.8843 | 0.8680 |
| 903 | 0.8624 | 0.9267 | 0.9766 | 0.8229 | 0.6910 | 0.9738 | 0.9639 | 0.8511 | 0.8843 | 0.8680 |
| 904 | 0.8624 | 0.9267 | 0.9766 | 0.8229 | 0.6910 | 0.9738 | 0.9639 | 0.8511 | 0.8843 | 0.8680 |
| 905 | 0.8624 | 0.9267 | 0.9766 | 0.8211 | 0.6891 | 0.9738 | 0.9639 | 0.8494 | 0.8836 | 0.8673 |
| 906 | 0.8624 | 0.9267 | 0.9766 | 0.8229 | 0.6910 | 0.9738 | 0.9656 | 0.8476 | 0.8841 | 0.8678 |
| 907 | 0.8624 | 0.9267 | 0.9766 | 0.8229 | 0.6891 | 0.9738 | 0.9656 | 0.8476 | 0.8838 | 0.8675 |
| 908 | 0.8624 | 0.9267 | 0.9766 | 0.8229 | 0.6929 | 0.9738 | 0.9656 | 0.8476 | 0.8843 | 0.8680 |
| 909 | 0.8607 | 0.9285 | 0.9766 | 0.8193 | 0.6929 | 0.9738 | 0.9656 | 0.8494 | 0.8841 | 0.8678 |
| 910 | 0.8589 | 0.9267 | 0.9766 | 0.8193 | 0.6910 | 0.9738 | 0.9656 | 0.8476 | 0.8831 | 0.8667 |
| 911 | 0.8589 | 0.9267 | 0.9785 | 0.8175 | 0.6929 | 0.9757 | 0.9656 | 0.8459 | 0.8834 | 0.8670 |
| 912 | 0.8589 | 0.9267 | 0.9785 | 0.8175 | 0.6871 | 0.9757 | 0.9656 | 0.8459 | 0.8827 | 0.8662 |
| 913 | 0.8589 | 0.9267 | 0.9785 | 0.8193 | 0.6891 | 0.9757 | 0.9656 | 0.8441 | 0.8829 | 0.8665 |
| 914 | 0.8589 | 0.9267 | 0.9785 | 0.8175 | 0.6891 | 0.9757 | 0.9656 | 0.8459 | 0.8829 | 0.8665 |
| 915 | 0.8607 | 0.9267 | 0.9785 | 0.8140 | 0.6871 | 0.9757 | 0.9656 | 0.8459 | 0.8825 | 0.8660 |
| 916 | 0.8589 | 0.9267 | 0.9785 | 0.8122 | 0.6833 | 0.9794 | 0.9656 | 0.8459 | 0.8820 | 0.8655 |
| 917 | 0.8677 | 0.9267 | 0.9785 | 0.8086 | 0.6795 | 0.9794 | 0.9656 | 0.8459 | 0.8822 | 0.8658 |
| 918 | 0.8677 | 0.9267 | 0.9785 | 0.8086 | 0.6775 | 0.9794 | 0.9656 | 0.8476 | 0.8822 | 0.8658 |
| 919 | 0.8677 | 0.9267 | 0.9785 | 0.8086 | 0.6814 | 0.9794 | 0.9656 | 0.8441 | 0.8822 | 0.8658 |
| 920 | 0.8677 | 0.9285 | 0.9785 | 0.8104 | 0.6795 | 0.9794 | 0.9656 | 0.8441 | 0.8825 | 0.8660 |
| 921 | 0.8695 | 0.9285 | 0.9785 | 0.8104 | 0.6871 | 0.9794 | 0.9656 | 0.8441 | 0.8836 | 0.8673 |
| 922 | 0.8677 | 0.9285 | 0.9785 | 0.8104 | 0.6871 | 0.9794 | 0.9656 | 0.8406 | 0.8829 | 0.8665 |
| 923 | 0.8660 | 0.9285 | 0.9785 | 0.8122 | 0.6871 | 0.9794 | 0.9656 | 0.8424 | 0.8831 | 0.8668 |
| 924 | 0.8677 | 0.9285 | 0.9785 | 0.8122 | 0.6852 | 0.9794 | 0.9656 | 0.8424 | 0.8831 | 0.8668 |
| 925 | 0.8677 | 0.9285 | 0.9785 | 0.8140 | 0.6871 | 0.9794 | 0.9656 | 0.8424 | 0.8836 | 0.8673 |
| 926 | 0.8660 | 0.9285 | 0.9785 | 0.8157 | 0.6871 | 0.9794 | 0.9656 | 0.8424 | 0.8836 | 0.8673 |
| 927 | 0.8677 | 0.9285 | 0.9785 | 0.8122 | 0.6891 | 0.9794 | 0.9656 | 0.8406 | 0.8834 | 0.8670 |
| 928 | 0.8642 | 0.9285 | 0.9785 | 0.8122 | 0.6833 | 0.9794 | 0.9656 | 0.8424 | 0.8825 | 0.8660 |
| 929 | 0.8642 | 0.9285 | 0.9785 | 0.8122 | 0.6852 | 0.9794 | 0.9656 | 0.8406 | 0.8825 | 0.8660 |
| 930 | 0.8642 | 0.9267 | 0.9785 | 0.8157 | 0.6852 | 0.9794 | 0.9656 | 0.8406 | 0.8827 | 0.8663 |
| 931 | 0.8642 | 0.9267 | 0.9785 | 0.8157 | 0.6852 | 0.9794 | 0.9656 | 0.8424 | 0.8829 | 0.8665 |
| 932 | 0.8642 | 0.9267 | 0.9785 | 0.8140 | 0.6852 | 0.9794 | 0.9656 | 0.8424 | 0.8827 | 0.8663 |
| 933 | 0.8642 | 0.9267 | 0.9785 | 0.8140 | 0.6852 | 0.9794 | 0.9656 | 0.8424 | 0.8827 | 0.8663 |
| 934 | 0.8642 | 0.9267 | 0.9785 | 0.8122 | 0.6852 | 0.9776 | 0.9656 | 0.8424 | 0.8822 | 0.8658 |
| 935 | 0.8642 | 0.9267 | 0.9785 | 0.8104 | 0.6871 | 0.9776 | 0.9656 | 0.8424 | 0.8822 | 0.8657 |
| 936 | 0.8624 | 0.9267 | 0.9785 | 0.8122 | 0.6852 | 0.9776 | 0.9656 | 0.8424 | 0.8820 | 0.8655 |
| 937 | 0.8695 | 0.9267 | 0.9785 | 0.8086 | 0.6910 | 0.9776 | 0.9656 | 0.8424 | 0.8831 | 0.8668 |
| 938 | 0.8695 | 0.9267 | 0.9785 | 0.8086 | 0.6891 | 0.9776 | 0.9656 | 0.8424 | 0.8829 | 0.8665 |
| 939 | 0.8695 | 0.9285 | 0.9785 | 0.8104 | 0.6910 | 0.9776 | 0.9656 | 0.8424 | 0.8836 | 0.8673 |
| 940 | 0.8713 | 0.9302 | 0.9785 | 0.8122 | 0.6891 | 0.9776 | 0.9656 | 0.8424 | 0.8841 | 0.8678 |
| 941 | 0.8713 | 0.9302 | 0.9785 | 0.8122 | 0.6929 | 0.9776 | 0.9656 | 0.8424 | 0.8845 | 0.8683 |
| 942 | 0.8730 | 0.9302 | 0.9785 | 0.8140 | 0.6910 | 0.9776 | 0.9656 | 0.8424 | 0.8847 | 0.8686 |
| 943 | 0.8730 | 0.9302 | 0.9805 | 0.8140 | 0.6910 | 0.9776 | 0.9656 | 0.8424 | 0.8850 | 0.8688 |
| 944 | 0.8730 | 0.9302 | 0.9805 | 0.8140 | 0.6910 | 0.9776 | 0.9656 | 0.8424 | 0.8850 | 0.8688 |
| 945 | 0.8695 | 0.9285 | 0.9805 | 0.8140 | 0.6929 | 0.9776 | 0.9656 | 0.8424 | 0.8845 | 0.8683 |
| 946 | 0.8713 | 0.9285 | 0.9805 | 0.8140 | 0.6929 | 0.9776 | 0.9656 | 0.8441 | 0.8850 | 0.8688 |
| 947 | 0.8695 | 0.9285 | 0.9805 | 0.8140 | 0.6987 | 0.9776 | 0.9656 | 0.8441 | 0.8854 | 0.8693 |
| 948 | 0.8713 | 0.9285 | 0.9805 | 0.8140 | 0.6987 | 0.9776 | 0.9656 | 0.8459 | 0.8859 | 0.8699 |
| 949 | 0.8677 | 0.9285 | 0.9805 | 0.8140 | 0.7006 | 0.9776 | 0.9673 | 0.8441 | 0.8856 | 0.8696 |
| 950 | 0.8695 | 0.9285 | 0.9785 | 0.8122 | 0.6967 | 0.9794 | 0.9673 | 0.8441 | 0.8852 | 0.8691 |
| 951 | 0.8695 | 0.9267 | 0.9785 | 0.8122 | 0.6967 | 0.9794 | 0.9673 | 0.8441 | 0.8850 | 0.8688 |
| 952 | 0.8695 | 0.9285 | 0.9785 | 0.8122 | 0.6948 | 0.9794 | 0.9673 | 0.8441 | 0.8850 | 0.8688 |
| 953 | 0.8677 | 0.9285 | 0.9785 | 0.8122 | 0.6948 | 0.9776 | 0.9690 | 0.8441 | 0.8847 | 0.8686 |
| 954 | 0.8660 | 0.9285 | 0.9785 | 0.8122 | 0.6948 | 0.9794 | 0.9690 | 0.8441 | 0.8847 | 0.8686 |
| 955 | 0.8660 | 0.9285 | 0.9785 | 0.8104 | 0.6948 | 0.9757 | 0.9690 | 0.8424 | 0.8838 | 0.8675 |
| 956 | 0.8660 | 0.9285 | 0.9785 | 0.8122 | 0.6948 | 0.9757 | 0.9690 | 0.8441 | 0.8843 | 0.8680 |
| 957 | 0.8624 | 0.9285 | 0.9785 | 0.8086 | 0.6910 | 0.9776 | 0.9690 | 0.8424 | 0.8829 | 0.8665 |
| 958 | 0.8642 | 0.9285 | 0.9785 | 0.8104 | 0.6948 | 0.9776 | 0.9690 | 0.8424 | 0.8838 | 0.8675 |
| 959 | 0.8642 | 0.9285 | 0.9785 | 0.8104 | 0.6910 | 0.9776 | 0.9690 | 0.8424 | 0.8834 | 0.8670 |
| 960 | 0.8624 | 0.9285 | 0.9785 | 0.8086 | 0.6910 | 0.9776 | 0.9690 | 0.8424 | 0.8829 | 0.8665 |
| 961 | 0.8642 | 0.9285 | 0.9805 | 0.8086 | 0.6910 | 0.9776 | 0.9690 | 0.8424 | 0.8834 | 0.8670 |
| 962 | 0.8660 | 0.9285 | 0.9805 | 0.8086 | 0.6891 | 0.9776 | 0.9690 | 0.8406 | 0.8831 | 0.8667 |
| 963 | 0.8660 | 0.9285 | 0.9805 | 0.8050 | 0.6910 | 0.9776 | 0.9690 | 0.8406 | 0.8829 | 0.8665 |
| 964 | 0.8660 | 0.9285 | 0.9805 | 0.8050 | 0.6910 | 0.9776 | 0.9690 | 0.8406 | 0.8829 | 0.8665 |
| 965 | 0.8660 | 0.9285 | 0.9805 | 0.8050 | 0.6871 | 0.9776 | 0.9690 | 0.8441 | 0.8829 | 0.8665 |
| 966 | 0.8677 | 0.9285 | 0.9805 | 0.8050 | 0.6852 | 0.9776 | 0.9690 | 0.8424 | 0.8827 | 0.8662 |
| 967 | 0.8677 | 0.9285 | 0.9805 | 0.8050 | 0.6852 | 0.9776 | 0.9690 | 0.8424 | 0.8827 | 0.8662 |
| 968 | 0.8695 | 0.9285 | 0.9805 | 0.8050 | 0.6852 | 0.9776 | 0.9690 | 0.8424 | 0.8829 | 0.8665 |
| 969 | 0.8695 | 0.9285 | 0.9805 | 0.8068 | 0.6852 | 0.9776 | 0.9690 | 0.8424 | 0.8831 | 0.8668 |
| 970 | 0.8695 | 0.9285 | 0.9805 | 0.8068 | 0.6871 | 0.9776 | 0.9690 | 0.8424 | 0.8834 | 0.8670 |
| 971 | 0.8695 | 0.9285 | 0.9805 | 0.8050 | 0.6871 | 0.9776 | 0.9690 | 0.8424 | 0.8831 | 0.8668 |
| 972 | 0.8695 | 0.9285 | 0.9805 | 0.8050 | 0.6871 | 0.9776 | 0.9690 | 0.8424 | 0.8831 | 0.8668 |
| 973 | 0.8713 | 0.9302 | 0.9805 | 0.8032 | 0.6871 | 0.9738 | 0.9690 | 0.8424 | 0.8829 | 0.8665 |
| 974 | 0.8713 | 0.9302 | 0.9805 | 0.8032 | 0.6871 | 0.9738 | 0.9690 | 0.8406 | 0.8827 | 0.8662 |
| 975 | 0.8713 | 0.9302 | 0.9805 | 0.8032 | 0.6871 | 0.9738 | 0.9690 | 0.8424 | 0.8829 | 0.8665 |
| 976 | 0.8713 | 0.9302 | 0.9805 | 0.8032 | 0.6871 | 0.9738 | 0.9690 | 0.8424 | 0.8829 | 0.8665 |
| 977 | 0.8713 | 0.9302 | 0.9805 | 0.8032 | 0.6891 | 0.9738 | 0.9690 | 0.8424 | 0.8831 | 0.8667 |
| 978 | 0.8713 | 0.9285 | 0.9805 | 0.8032 | 0.6910 | 0.9738 | 0.9690 | 0.8406 | 0.8829 | 0.8665 |
| 979 | 0.8713 | 0.9285 | 0.9805 | 0.8050 | 0.6910 | 0.9738 | 0.9690 | 0.8424 | 0.8834 | 0.8670 |
| 980 | 0.8695 | 0.9285 | 0.9805 | 0.8050 | 0.6871 | 0.9738 | 0.9690 | 0.8424 | 0.8827 | 0.8662 |
| 981 | 0.8695 | 0.9285 | 0.9805 | 0.8086 | 0.6871 | 0.9738 | 0.9690 | 0.8441 | 0.8834 | 0.8670 |
| 982 | 0.8695 | 0.9285 | 0.9805 | 0.8086 | 0.6871 | 0.9738 | 0.9690 | 0.8441 | 0.8834 | 0.8670 |
| 983 | 0.8677 | 0.9285 | 0.9805 | 0.8050 | 0.6910 | 0.9738 | 0.9690 | 0.8441 | 0.8831 | 0.8667 |
| 984 | 0.8695 | 0.9285 | 0.9805 | 0.8050 | 0.6910 | 0.9738 | 0.9690 | 0.8441 | 0.8834 | 0.8670 |
| 985 | 0.8695 | 0.9285 | 0.9805 | 0.8050 | 0.6910 | 0.9738 | 0.9690 | 0.8459 | 0.8836 | 0.8672 |
| 986 | 0.8677 | 0.9285 | 0.9805 | 0.8050 | 0.6929 | 0.9738 | 0.9690 | 0.8459 | 0.8836 | 0.8672 |
| 987 | 0.8677 | 0.9285 | 0.9805 | 0.8068 | 0.6929 | 0.9738 | 0.9690 | 0.8459 | 0.8838 | 0.8675 |
| 988 | 0.8677 | 0.9285 | 0.9805 | 0.8086 | 0.6948 | 0.9738 | 0.9690 | 0.8424 | 0.8838 | 0.8675 |
| 989 | 0.8677 | 0.9285 | 0.9805 | 0.8086 | 0.6948 | 0.9738 | 0.9690 | 0.8441 | 0.8841 | 0.8678 |
| 990 | 0.8677 | 0.9285 | 0.9805 | 0.8086 | 0.6948 | 0.9738 | 0.9690 | 0.8441 | 0.8841 | 0.8678 |
| 991 | 0.8677 | 0.9285 | 0.9805 | 0.8104 | 0.6948 | 0.9738 | 0.9690 | 0.8424 | 0.8841 | 0.8678 |
| 992 | 0.8660 | 0.9285 | 0.9805 | 0.8122 | 0.6948 | 0.9738 | 0.9690 | 0.8424 | 0.8841 | 0.8678 |
| 993 | 0.8660 | 0.9285 | 0.9805 | 0.8104 | 0.6948 | 0.9738 | 0.9690 | 0.8424 | 0.8838 | 0.8675 |
| 994 | 0.8677 | 0.9285 | 0.9805 | 0.8086 | 0.6967 | 0.9738 | 0.9690 | 0.8424 | 0.8841 | 0.8678 |
| 995 | 0.8677 | 0.9285 | 0.9805 | 0.8086 | 0.6967 | 0.9738 | 0.9690 | 0.8424 | 0.8841 | 0.8678 |
| 996 | 0.8660 | 0.9285 | 0.9805 | 0.8122 | 0.6948 | 0.9738 | 0.9690 | 0.8441 | 0.8843 | 0.8680 |
| 997 | 0.8660 | 0.9285 | 0.9805 | 0.8104 | 0.6929 | 0.9738 | 0.9690 | 0.8441 | 0.8838 | 0.8675 |
| 998 | 0.8677 | 0.9285 | 0.9805 | 0.8122 | 0.6891 | 0.9738 | 0.9690 | 0.8441 | 0.8838 | 0.8675 |
| 999 | 0.8677 | 0.9285 | 0.9805 | 0.8122 | 0.6891 | 0.9738 | 0.9690 | 0.8441 | 0.8838 | 0.8675 |
| 1000 | 0.8660 | 0.9285 | 0.9805 | 0.8122 | 0.6891 | 0.9738 | 0.9690 | 0.8441 | 0.8836 | 0.8673 |
| 1001 | 0.8677 | 0.9285 | 0.9805 | 0.8122 | 0.6891 | 0.9738 | 0.9690 | 0.8441 | 0.8838 | 0.8675 |
| 1002 | 0.8677 | 0.9285 | 0.9805 | 0.8122 | 0.6871 | 0.9757 | 0.9690 | 0.8441 | 0.8838 | 0.8675 |
| 1003 | 0.8660 | 0.9285 | 0.9805 | 0.8122 | 0.6871 | 0.9757 | 0.9690 | 0.8441 | 0.8836 | 0.8673 |
| 1004 | 0.8677 | 0.9285 | 0.9805 | 0.8104 | 0.6871 | 0.9757 | 0.9690 | 0.8441 | 0.8836 | 0.8673 |
| 1005 | 0.8677 | 0.9285 | 0.9805 | 0.8104 | 0.6871 | 0.9757 | 0.9690 | 0.8441 | 0.8836 | 0.8673 |
| 1006 | 0.8695 | 0.9285 | 0.9805 | 0.8104 | 0.6929 | 0.9757 | 0.9690 | 0.8424 | 0.8843 | 0.8680 |
| 1007 | 0.8695 | 0.9285 | 0.9805 | 0.8122 | 0.6929 | 0.9757 | 0.9690 | 0.8424 | 0.8845 | 0.8683 |
| 1008 | 0.8695 | 0.9285 | 0.9805 | 0.8122 | 0.6910 | 0.9757 | 0.9690 | 0.8424 | 0.8843 | 0.8681 |
| 1009 | 0.8695 | 0.9285 | 0.9805 | 0.8122 | 0.6929 | 0.9757 | 0.9690 | 0.8424 | 0.8845 | 0.8683 |
| 1010 | 0.8695 | 0.9302 | 0.9805 | 0.8122 | 0.6929 | 0.9757 | 0.9690 | 0.8406 | 0.8845 | 0.8683 |
| 1011 | 0.8695 | 0.9302 | 0.9805 | 0.8104 | 0.6929 | 0.9757 | 0.9690 | 0.8424 | 0.8845 | 0.8683 |
| 1012 | 0.8695 | 0.9302 | 0.9805 | 0.8086 | 0.6910 | 0.9757 | 0.9690 | 0.8406 | 0.8838 | 0.8675 |
| 1013 | 0.8695 | 0.9302 | 0.9805 | 0.8086 | 0.6929 | 0.9757 | 0.9690 | 0.8406 | 0.8841 | 0.8678 |
| 1014 | 0.8695 | 0.9302 | 0.9805 | 0.8086 | 0.6910 | 0.9738 | 0.9690 | 0.8424 | 0.8838 | 0.8675 |
| 1015 | 0.8677 | 0.9285 | 0.9805 | 0.8086 | 0.6910 | 0.9738 | 0.9690 | 0.8424 | 0.8834 | 0.8670 |
| 1016 | 0.8677 | 0.9302 | 0.9805 | 0.8068 | 0.6891 | 0.9738 | 0.9690 | 0.8424 | 0.8831 | 0.8667 |
| 1017 | 0.8660 | 0.9302 | 0.9805 | 0.8086 | 0.6891 | 0.9738 | 0.9690 | 0.8424 | 0.8831 | 0.8667 |
| 1018 | 0.8660 | 0.9302 | 0.9805 | 0.8104 | 0.6871 | 0.9738 | 0.9690 | 0.8424 | 0.8831 | 0.8667 |
| 1019 | 0.8642 | 0.9285 | 0.9805 | 0.8086 | 0.6871 | 0.9738 | 0.9690 | 0.8424 | 0.8825 | 0.8660 |
| 1020 | 0.8660 | 0.9285 | 0.9805 | 0.8086 | 0.6871 | 0.9738 | 0.9690 | 0.8441 | 0.8829 | 0.8665 |
| 1021 | 0.8677 | 0.9302 | 0.9805 | 0.8086 | 0.6910 | 0.9738 | 0.9690 | 0.8441 | 0.8838 | 0.8675 |
| 1022 | 0.8677 | 0.9302 | 0.9805 | 0.8068 | 0.6910 | 0.9738 | 0.9690 | 0.8441 | 0.8836 | 0.8673 |
| 1023 | 0.8677 | 0.9302 | 0.9805 | 0.8086 | 0.6910 | 0.9738 | 0.9690 | 0.8424 | 0.8836 | 0.8673 |
| 1024 | 0.8677 | 0.9302 | 0.9805 | 0.8068 | 0.6910 | 0.9738 | 0.9690 | 0.8424 | 0.8834 | 0.8670 |
| 1025 | 0.8677 | 0.9302 | 0.9805 | 0.8086 | 0.6910 | 0.9738 | 0.9690 | 0.8424 | 0.8836 | 0.8673 |
| 1026 | 0.8624 | 0.9302 | 0.9805 | 0.8086 | 0.6910 | 0.9738 | 0.9690 | 0.8441 | 0.8831 | 0.8667 |
| 1027 | 0.8607 | 0.9302 | 0.9805 | 0.8086 | 0.6910 | 0.9738 | 0.9690 | 0.8441 | 0.8829 | 0.8665 |
| 1028 | 0.8642 | 0.9302 | 0.9805 | 0.8068 | 0.6852 | 0.9738 | 0.9690 | 0.8424 | 0.8822 | 0.8657 |
| 1029 | 0.8642 | 0.9302 | 0.9805 | 0.8086 | 0.6871 | 0.9738 | 0.9690 | 0.8406 | 0.8825 | 0.8660 |
| 1030 | 0.8642 | 0.9302 | 0.9805 | 0.8122 | 0.6852 | 0.9757 | 0.9690 | 0.8406 | 0.8829 | 0.8665 |
| 1031 | 0.8642 | 0.9302 | 0.9805 | 0.8122 | 0.6852 | 0.9757 | 0.9690 | 0.8389 | 0.8827 | 0.8663 |
| 1032 | 0.8642 | 0.9302 | 0.9805 | 0.8122 | 0.6852 | 0.9738 | 0.9690 | 0.8406 | 0.8827 | 0.8663 |
| 1033 | 0.8607 | 0.9302 | 0.9805 | 0.8157 | 0.6852 | 0.9738 | 0.9690 | 0.8371 | 0.8822 | 0.8658 |
| 1034 | 0.8607 | 0.9302 | 0.9805 | 0.8157 | 0.6852 | 0.9738 | 0.9690 | 0.8371 | 0.8822 | 0.8658 |
| 1035 | 0.8607 | 0.9302 | 0.9805 | 0.8122 | 0.6852 | 0.9738 | 0.9690 | 0.8371 | 0.8818 | 0.8652 |
| 1036 | 0.8607 | 0.9302 | 0.9805 | 0.8122 | 0.6852 | 0.9738 | 0.9690 | 0.8371 | 0.8818 | 0.8652 |
| 1037 | 0.8624 | 0.9302 | 0.9805 | 0.8122 | 0.6852 | 0.9738 | 0.9690 | 0.8371 | 0.8820 | 0.8655 |
| 1038 | 0.8660 | 0.9302 | 0.9805 | 0.8122 | 0.6852 | 0.9738 | 0.9690 | 0.8371 | 0.8825 | 0.8660 |
| 1039 | 0.8642 | 0.9302 | 0.9805 | 0.8122 | 0.6833 | 0.9738 | 0.9690 | 0.8371 | 0.8820 | 0.8655 |
| 1040 | 0.8677 | 0.9302 | 0.9805 | 0.8175 | 0.6929 | 0.9738 | 0.9690 | 0.8406 | 0.8847 | 0.8686 |
| 1041 | 0.8660 | 0.9302 | 0.9805 | 0.8175 | 0.6891 | 0.9738 | 0.9690 | 0.8406 | 0.8841 | 0.8678 |
| 1042 | 0.8677 | 0.9302 | 0.9805 | 0.8175 | 0.6910 | 0.9738 | 0.9690 | 0.8406 | 0.8845 | 0.8683 |
| 1043 | 0.8660 | 0.9302 | 0.9805 | 0.8193 | 0.6910 | 0.9738 | 0.9690 | 0.8406 | 0.8845 | 0.8683 |
| 1044 | 0.8642 | 0.9302 | 0.9805 | 0.8193 | 0.6929 | 0.9738 | 0.9690 | 0.8406 | 0.8845 | 0.8683 |
| 1045 | 0.8624 | 0.9302 | 0.9805 | 0.8229 | 0.6948 | 0.9738 | 0.9690 | 0.8406 | 0.8850 | 0.8688 |
| 1046 | 0.8624 | 0.9302 | 0.9805 | 0.8229 | 0.6948 | 0.9738 | 0.9690 | 0.8389 | 0.8847 | 0.8686 |
| 1047 | 0.8571 | 0.9302 | 0.9805 | 0.8229 | 0.6948 | 0.9738 | 0.9690 | 0.8389 | 0.8841 | 0.8678 |
| 1048 | 0.8571 | 0.9319 | 0.9805 | 0.8229 | 0.6948 | 0.9738 | 0.9690 | 0.8371 | 0.8841 | 0.8678 |
| 1049 | 0.8571 | 0.9319 | 0.9805 | 0.8229 | 0.6948 | 0.9738 | 0.9690 | 0.8371 | 0.8841 | 0.8678 |
| 1050 | 0.8571 | 0.9319 | 0.9805 | 0.8229 | 0.6948 | 0.9738 | 0.9690 | 0.8371 | 0.8841 | 0.8678 |
| 1051 | 0.8554 | 0.9302 | 0.9805 | 0.8229 | 0.6948 | 0.9738 | 0.9690 | 0.8371 | 0.8836 | 0.8673 |
| 1052 | 0.8571 | 0.9302 | 0.9805 | 0.8229 | 0.6929 | 0.9738 | 0.9690 | 0.8371 | 0.8836 | 0.8673 |
| 1053 | 0.8589 | 0.9319 | 0.9805 | 0.8229 | 0.6948 | 0.9738 | 0.9690 | 0.8371 | 0.8843 | 0.8681 |
| 1054 | 0.8589 | 0.9319 | 0.9805 | 0.8229 | 0.6948 | 0.9738 | 0.9690 | 0.8371 | 0.8843 | 0.8681 |
| 1055 | 0.8519 | 0.9302 | 0.9805 | 0.8211 | 0.6910 | 0.9738 | 0.9690 | 0.8371 | 0.8825 | 0.8660 |
| 1056 | 0.8519 | 0.9302 | 0.9805 | 0.8211 | 0.6910 | 0.9738 | 0.9690 | 0.8371 | 0.8825 | 0.8660 |
| 1057 | 0.8554 | 0.9285 | 0.9805 | 0.8211 | 0.6910 | 0.9738 | 0.9690 | 0.8371 | 0.8827 | 0.8663 |
| 1058 | 0.8554 | 0.9302 | 0.9805 | 0.8193 | 0.6929 | 0.9738 | 0.9690 | 0.8371 | 0.8829 | 0.8665 |
| 1059 | 0.8554 | 0.9302 | 0.9805 | 0.8211 | 0.6910 | 0.9738 | 0.9690 | 0.8371 | 0.8829 | 0.8665 |
| 1060 | 0.8536 | 0.9319 | 0.9805 | 0.8193 | 0.6910 | 0.9738 | 0.9690 | 0.8371 | 0.8827 | 0.8662 |
| 1061 | 0.8571 | 0.9319 | 0.9805 | 0.8175 | 0.6891 | 0.9738 | 0.9690 | 0.8371 | 0.8827 | 0.8663 |
| 1062 | 0.8554 | 0.9319 | 0.9805 | 0.8157 | 0.6910 | 0.9738 | 0.9690 | 0.8371 | 0.8825 | 0.8660 |
| 1063 | 0.8571 | 0.9302 | 0.9805 | 0.8157 | 0.6891 | 0.9738 | 0.9690 | 0.8371 | 0.8822 | 0.8657 |
| 1064 | 0.8571 | 0.9302 | 0.9805 | 0.8157 | 0.6891 | 0.9738 | 0.9690 | 0.8371 | 0.8822 | 0.8657 |
| 1065 | 0.8607 | 0.9302 | 0.9805 | 0.8140 | 0.6891 | 0.9738 | 0.9690 | 0.8371 | 0.8825 | 0.8660 |
| 1066 | 0.8589 | 0.9302 | 0.9805 | 0.8140 | 0.6910 | 0.9738 | 0.9690 | 0.8371 | 0.8825 | 0.8660 |
| 1067 | 0.8607 | 0.9302 | 0.9805 | 0.8140 | 0.6910 | 0.9757 | 0.9690 | 0.8371 | 0.8829 | 0.8665 |
| 1068 | 0.8624 | 0.9302 | 0.9805 | 0.8140 | 0.6891 | 0.9757 | 0.9690 | 0.8371 | 0.8829 | 0.8665 |
| 1069 | 0.8607 | 0.9302 | 0.9805 | 0.8140 | 0.6891 | 0.9757 | 0.9690 | 0.8371 | 0.8827 | 0.8663 |
| 1070 | 0.8571 | 0.9302 | 0.9805 | 0.8140 | 0.6929 | 0.9757 | 0.9690 | 0.8371 | 0.8827 | 0.8662 |
| 1071 | 0.8624 | 0.9302 | 0.9805 | 0.8122 | 0.6929 | 0.9757 | 0.9690 | 0.8371 | 0.8831 | 0.8668 |
| 1072 | 0.8624 | 0.9302 | 0.9805 | 0.8122 | 0.6929 | 0.9757 | 0.9690 | 0.8371 | 0.8831 | 0.8668 |
| 1073 | 0.8624 | 0.9302 | 0.9805 | 0.8175 | 0.6910 | 0.9738 | 0.9690 | 0.8371 | 0.8834 | 0.8670 |
| 1074 | 0.8607 | 0.9302 | 0.9805 | 0.8175 | 0.6891 | 0.9738 | 0.9690 | 0.8371 | 0.8829 | 0.8665 |
| 1075 | 0.8660 | 0.9302 | 0.9805 | 0.8157 | 0.6929 | 0.9738 | 0.9690 | 0.8371 | 0.8838 | 0.8675 |
| 1076 | 0.8660 | 0.9302 | 0.9805 | 0.8157 | 0.6929 | 0.9738 | 0.9690 | 0.8371 | 0.8838 | 0.8675 |
| 1077 | 0.8660 | 0.9302 | 0.9805 | 0.8157 | 0.6929 | 0.9757 | 0.9690 | 0.8371 | 0.8841 | 0.8678 |
| 1078 | 0.8589 | 0.9302 | 0.9805 | 0.8175 | 0.6891 | 0.9757 | 0.9690 | 0.8389 | 0.8831 | 0.8668 |
| 1079 | 0.8589 | 0.9302 | 0.9805 | 0.8157 | 0.6891 | 0.9757 | 0.9690 | 0.8371 | 0.8827 | 0.8663 |
| 1080 | 0.8607 | 0.9302 | 0.9805 | 0.8175 | 0.6891 | 0.9757 | 0.9690 | 0.8371 | 0.8831 | 0.8668 |
| 1081 | 0.8607 | 0.9302 | 0.9805 | 0.8175 | 0.6891 | 0.9757 | 0.9690 | 0.8371 | 0.8831 | 0.8668 |
| 1082 | 0.8660 | 0.9302 | 0.9805 | 0.8175 | 0.6891 | 0.9757 | 0.9690 | 0.8389 | 0.8841 | 0.8678 |
| 1083 | 0.8660 | 0.9285 | 0.9805 | 0.8175 | 0.6891 | 0.9757 | 0.9690 | 0.8389 | 0.8838 | 0.8676 |
| 1084 | 0.8660 | 0.9285 | 0.9805 | 0.8175 | 0.6891 | 0.9757 | 0.9690 | 0.8389 | 0.8838 | 0.8676 |
| 1085 | 0.8660 | 0.9285 | 0.9805 | 0.8175 | 0.6891 | 0.9757 | 0.9690 | 0.8389 | 0.8838 | 0.8676 |
| 1086 | 0.8660 | 0.9285 | 0.9805 | 0.8175 | 0.6891 | 0.9757 | 0.9690 | 0.8389 | 0.8838 | 0.8676 |
| 1087 | 0.8660 | 0.9285 | 0.9805 | 0.8157 | 0.6910 | 0.9757 | 0.9690 | 0.8406 | 0.8841 | 0.8678 |
| 1088 | 0.8642 | 0.9285 | 0.9805 | 0.8157 | 0.6910 | 0.9757 | 0.9690 | 0.8406 | 0.8838 | 0.8675 |
| 1089 | 0.8660 | 0.9285 | 0.9805 | 0.8157 | 0.6910 | 0.9757 | 0.9690 | 0.8406 | 0.8841 | 0.8678 |
| 1090 | 0.8660 | 0.9285 | 0.9805 | 0.8175 | 0.6910 | 0.9757 | 0.9690 | 0.8406 | 0.8843 | 0.8681 |
| 1091 | 0.8660 | 0.9285 | 0.9805 | 0.8175 | 0.6852 | 0.9757 | 0.9690 | 0.8406 | 0.8836 | 0.8673 |
| 1092 | 0.8642 | 0.9285 | 0.9805 | 0.8175 | 0.6852 | 0.9738 | 0.9690 | 0.8389 | 0.8829 | 0.8665 |
| 1093 | 0.8660 | 0.9285 | 0.9805 | 0.8175 | 0.6852 | 0.9738 | 0.9690 | 0.8389 | 0.8831 | 0.8668 |
| 1094 | 0.8677 | 0.9285 | 0.9805 | 0.8157 | 0.6852 | 0.9738 | 0.9690 | 0.8389 | 0.8831 | 0.8668 |
| 1095 | 0.8642 | 0.9285 | 0.9805 | 0.8175 | 0.6871 | 0.9738 | 0.9690 | 0.8389 | 0.8831 | 0.8668 |
| 1096 | 0.8642 | 0.9285 | 0.9805 | 0.8140 | 0.6871 | 0.9738 | 0.9690 | 0.8389 | 0.8827 | 0.8663 |
| 1097 | 0.8642 | 0.9285 | 0.9805 | 0.8140 | 0.6871 | 0.9738 | 0.9690 | 0.8406 | 0.8829 | 0.8665 |
| 1098 | 0.8677 | 0.9285 | 0.9805 | 0.8140 | 0.6871 | 0.9738 | 0.9690 | 0.8406 | 0.8834 | 0.8670 |
| 1099 | 0.8677 | 0.9285 | 0.9805 | 0.8140 | 0.6833 | 0.9738 | 0.9690 | 0.8389 | 0.8827 | 0.8663 |
| 1100 | 0.8695 | 0.9285 | 0.9805 | 0.8140 | 0.6871 | 0.9738 | 0.9690 | 0.8354 | 0.8829 | 0.8665 |
| 1101 | 0.8660 | 0.9285 | 0.9805 | 0.8157 | 0.6929 | 0.9738 | 0.9690 | 0.8336 | 0.8831 | 0.8668 |
| 1102 | 0.8660 | 0.9285 | 0.9805 | 0.8157 | 0.6891 | 0.9738 | 0.9690 | 0.8336 | 0.8827 | 0.8663 |
| 1103 | 0.8660 | 0.9285 | 0.9805 | 0.8175 | 0.6891 | 0.9738 | 0.9690 | 0.8336 | 0.8829 | 0.8665 |
| 1104 | 0.8642 | 0.9285 | 0.9805 | 0.8140 | 0.6891 | 0.9738 | 0.9690 | 0.8336 | 0.8822 | 0.8657 |
| 1105 | 0.8642 | 0.9285 | 0.9805 | 0.8140 | 0.6871 | 0.9738 | 0.9690 | 0.8336 | 0.8820 | 0.8655 |
| 1106 | 0.8660 | 0.9285 | 0.9805 | 0.8140 | 0.6871 | 0.9738 | 0.9690 | 0.8336 | 0.8822 | 0.8657 |
| 1107 | 0.8660 | 0.9285 | 0.9805 | 0.8140 | 0.6852 | 0.9738 | 0.9690 | 0.8336 | 0.8820 | 0.8655 |
| 1108 | 0.8660 | 0.9285 | 0.9805 | 0.8157 | 0.6891 | 0.9738 | 0.9690 | 0.8354 | 0.8829 | 0.8665 |
| 1109 | 0.8677 | 0.9285 | 0.9805 | 0.8175 | 0.6871 | 0.9738 | 0.9690 | 0.8354 | 0.8831 | 0.8668 |
| 1110 | 0.8695 | 0.9285 | 0.9805 | 0.8175 | 0.6871 | 0.9738 | 0.9690 | 0.8354 | 0.8834 | 0.8670 |
| 1111 | 0.8713 | 0.9285 | 0.9805 | 0.8175 | 0.6871 | 0.9738 | 0.9690 | 0.8354 | 0.8836 | 0.8673 |
| 1112 | 0.8677 | 0.9285 | 0.9805 | 0.8175 | 0.6871 | 0.9738 | 0.9690 | 0.8354 | 0.8831 | 0.8668 |
| 1113 | 0.8677 | 0.9285 | 0.9805 | 0.8175 | 0.6871 | 0.9738 | 0.9690 | 0.8354 | 0.8831 | 0.8668 |
| 1114 | 0.8677 | 0.9285 | 0.9805 | 0.8193 | 0.6871 | 0.9738 | 0.9690 | 0.8354 | 0.8834 | 0.8670 |
| 1115 | 0.8677 | 0.9285 | 0.9805 | 0.8193 | 0.6871 | 0.9738 | 0.9690 | 0.8319 | 0.8829 | 0.8665 |
| 1116 | 0.8677 | 0.9285 | 0.9805 | 0.8122 | 0.6891 | 0.9738 | 0.9690 | 0.8319 | 0.8822 | 0.8657 |
| 1117 | 0.8677 | 0.9285 | 0.9805 | 0.8122 | 0.6871 | 0.9738 | 0.9690 | 0.8319 | 0.8820 | 0.8655 |
| 1118 | 0.8660 | 0.9285 | 0.9805 | 0.8104 | 0.6871 | 0.9738 | 0.9690 | 0.8319 | 0.8816 | 0.8650 |
| 1119 | 0.8660 | 0.9285 | 0.9805 | 0.8086 | 0.6891 | 0.9738 | 0.9690 | 0.8336 | 0.8818 | 0.8652 |
| 1120 | 0.8660 | 0.9285 | 0.9805 | 0.8086 | 0.6929 | 0.9738 | 0.9690 | 0.8336 | 0.8822 | 0.8657 |
| 1121 | 0.8660 | 0.9285 | 0.9805 | 0.8122 | 0.6929 | 0.9720 | 0.9690 | 0.8336 | 0.8825 | 0.8660 |
| 1122 | 0.8695 | 0.9285 | 0.9805 | 0.8140 | 0.6948 | 0.9720 | 0.9690 | 0.8319 | 0.8831 | 0.8668 |
| 1123 | 0.8713 | 0.9285 | 0.9805 | 0.8122 | 0.6987 | 0.9720 | 0.9690 | 0.8319 | 0.8836 | 0.8673 |
| 1124 | 0.8695 | 0.9285 | 0.9805 | 0.8140 | 0.6929 | 0.9720 | 0.9690 | 0.8319 | 0.8829 | 0.8665 |
| 1125 | 0.8695 | 0.9285 | 0.9805 | 0.8140 | 0.6910 | 0.9720 | 0.9690 | 0.8319 | 0.8827 | 0.8662 |
| 1126 | 0.8713 | 0.9285 | 0.9805 | 0.8122 | 0.6929 | 0.9720 | 0.9690 | 0.8319 | 0.8829 | 0.8665 |
| 1127 | 0.8713 | 0.9285 | 0.9805 | 0.8122 | 0.6929 | 0.9720 | 0.9690 | 0.8336 | 0.8831 | 0.8668 |
| 1128 | 0.8713 | 0.9285 | 0.9805 | 0.8122 | 0.6929 | 0.9720 | 0.9690 | 0.8336 | 0.8831 | 0.8668 |
| 1129 | 0.8677 | 0.9285 | 0.9805 | 0.8122 | 0.6929 | 0.9720 | 0.9690 | 0.8336 | 0.8827 | 0.8662 |
| 1130 | 0.8677 | 0.9285 | 0.9805 | 0.8122 | 0.6871 | 0.9720 | 0.9690 | 0.8336 | 0.8820 | 0.8655 |
| 1131 | 0.8642 | 0.9285 | 0.9805 | 0.8122 | 0.6871 | 0.9720 | 0.9690 | 0.8336 | 0.8816 | 0.8650 |
| 1132 | 0.8642 | 0.9285 | 0.9805 | 0.8122 | 0.6891 | 0.9720 | 0.9690 | 0.8336 | 0.8818 | 0.8652 |
| 1133 | 0.8642 | 0.9285 | 0.9805 | 0.8122 | 0.6871 | 0.9720 | 0.9690 | 0.8336 | 0.8816 | 0.8649 |
| 1134 | 0.8642 | 0.9285 | 0.9805 | 0.8122 | 0.6891 | 0.9720 | 0.9690 | 0.8336 | 0.8818 | 0.8652 |
| 1135 | 0.8642 | 0.9285 | 0.9805 | 0.8122 | 0.6852 | 0.9720 | 0.9690 | 0.8336 | 0.8813 | 0.8647 |
| 1136 | 0.8660 | 0.9285 | 0.9805 | 0.8122 | 0.6871 | 0.9720 | 0.9690 | 0.8336 | 0.8818 | 0.8652 |
| 1137 | 0.8677 | 0.9285 | 0.9805 | 0.8122 | 0.6852 | 0.9720 | 0.9690 | 0.8354 | 0.8820 | 0.8655 |
| 1138 | 0.8677 | 0.9285 | 0.9805 | 0.8122 | 0.6852 | 0.9720 | 0.9690 | 0.8336 | 0.8818 | 0.8652 |
| 1139 | 0.8660 | 0.9285 | 0.9805 | 0.8104 | 0.6852 | 0.9720 | 0.9690 | 0.8354 | 0.8816 | 0.8650 |
| 1140 | 0.8677 | 0.9285 | 0.9805 | 0.8104 | 0.6871 | 0.9720 | 0.9690 | 0.8301 | 0.8813 | 0.8647 |
| 1141 | 0.8677 | 0.9285 | 0.9805 | 0.8104 | 0.6871 | 0.9720 | 0.9690 | 0.8301 | 0.8813 | 0.8647 |
| 1142 | 0.8660 | 0.9285 | 0.9805 | 0.8104 | 0.6871 | 0.9720 | 0.9690 | 0.8301 | 0.8811 | 0.8644 |
| 1143 | 0.8660 | 0.9285 | 0.9805 | 0.8122 | 0.6891 | 0.9720 | 0.9690 | 0.8301 | 0.8816 | 0.8649 |
| 1144 | 0.8677 | 0.9285 | 0.9805 | 0.8157 | 0.6871 | 0.9720 | 0.9690 | 0.8301 | 0.8820 | 0.8655 |
| 1145 | 0.8677 | 0.9285 | 0.9805 | 0.8140 | 0.6871 | 0.9720 | 0.9690 | 0.8301 | 0.8818 | 0.8652 |
| 1146 | 0.8695 | 0.9285 | 0.9805 | 0.8140 | 0.6891 | 0.9720 | 0.9690 | 0.8319 | 0.8825 | 0.8660 |
| 1147 | 0.8695 | 0.9285 | 0.9805 | 0.8140 | 0.6891 | 0.9720 | 0.9690 | 0.8319 | 0.8825 | 0.8660 |
| 1148 | 0.8695 | 0.9285 | 0.9805 | 0.8140 | 0.6891 | 0.9738 | 0.9690 | 0.8336 | 0.8829 | 0.8665 |
| 1149 | 0.8695 | 0.9285 | 0.9805 | 0.8175 | 0.6871 | 0.9738 | 0.9690 | 0.8336 | 0.8831 | 0.8668 |
| 1150 | 0.8660 | 0.9285 | 0.9805 | 0.8175 | 0.6871 | 0.9738 | 0.9690 | 0.8354 | 0.8829 | 0.8665 |
| 1151 | 0.8660 | 0.9285 | 0.9805 | 0.8157 | 0.6871 | 0.9738 | 0.9690 | 0.8354 | 0.8827 | 0.8663 |
| 1152 | 0.8660 | 0.9285 | 0.9805 | 0.8157 | 0.6871 | 0.9738 | 0.9690 | 0.8354 | 0.8827 | 0.8663 |
| 1153 | 0.8660 | 0.9285 | 0.9805 | 0.8140 | 0.6871 | 0.9738 | 0.9690 | 0.8354 | 0.8825 | 0.8660 |
| 1154 | 0.8660 | 0.9285 | 0.9805 | 0.8122 | 0.6891 | 0.9720 | 0.9690 | 0.8336 | 0.8820 | 0.8655 |
| 1155 | 0.8642 | 0.9285 | 0.9805 | 0.8122 | 0.6852 | 0.9720 | 0.9690 | 0.8336 | 0.8813 | 0.8647 |
| 1156 | 0.8642 | 0.9285 | 0.9805 | 0.8140 | 0.6852 | 0.9720 | 0.9690 | 0.8336 | 0.8816 | 0.8650 |
| 1157 | 0.8642 | 0.9285 | 0.9805 | 0.8140 | 0.6852 | 0.9720 | 0.9690 | 0.8336 | 0.8816 | 0.8650 |
| 1158 | 0.8607 | 0.9285 | 0.9805 | 0.8122 | 0.6833 | 0.9720 | 0.9690 | 0.8336 | 0.8807 | 0.8639 |
| 1159 | 0.8624 | 0.9285 | 0.9805 | 0.8140 | 0.6871 | 0.9720 | 0.9690 | 0.8354 | 0.8818 | 0.8652 |
| 1160 | 0.8624 | 0.9285 | 0.9805 | 0.8140 | 0.6852 | 0.9720 | 0.9690 | 0.8354 | 0.8816 | 0.8649 |
| 1161 | 0.8624 | 0.9285 | 0.9805 | 0.8122 | 0.6871 | 0.9720 | 0.9690 | 0.8354 | 0.8816 | 0.8649 |
| 1162 | 0.8624 | 0.9285 | 0.9805 | 0.8140 | 0.6871 | 0.9720 | 0.9690 | 0.8354 | 0.8818 | 0.8652 |
| 1163 | 0.8624 | 0.9285 | 0.9805 | 0.8140 | 0.6852 | 0.9701 | 0.9690 | 0.8354 | 0.8813 | 0.8647 |
| 1164 | 0.8624 | 0.9285 | 0.9805 | 0.8140 | 0.6871 | 0.9701 | 0.9690 | 0.8336 | 0.8813 | 0.8647 |
| 1165 | 0.8660 | 0.9285 | 0.9805 | 0.8122 | 0.6852 | 0.9701 | 0.9690 | 0.8319 | 0.8811 | 0.8644 |
| 1166 | 0.8660 | 0.9285 | 0.9805 | 0.8122 | 0.6852 | 0.9701 | 0.9690 | 0.8319 | 0.8811 | 0.8644 |
| 1167 | 0.8660 | 0.9285 | 0.9805 | 0.8122 | 0.6833 | 0.9701 | 0.9690 | 0.8319 | 0.8809 | 0.8642 |
| 1168 | 0.8660 | 0.9285 | 0.9805 | 0.8122 | 0.6833 | 0.9701 | 0.9690 | 0.8319 | 0.8809 | 0.8642 |
| 1169 | 0.8660 | 0.9285 | 0.9805 | 0.8140 | 0.6833 | 0.9701 | 0.9690 | 0.8319 | 0.8811 | 0.8644 |
| 1170 | 0.8660 | 0.9285 | 0.9805 | 0.8140 | 0.6833 | 0.9701 | 0.9690 | 0.8319 | 0.8811 | 0.8644 |
| 1171 | 0.8607 | 0.9302 | 0.9805 | 0.8175 | 0.6814 | 0.9701 | 0.9690 | 0.8319 | 0.8809 | 0.8642 |
| 1172 | 0.8607 | 0.9302 | 0.9805 | 0.8175 | 0.6814 | 0.9701 | 0.9690 | 0.8319 | 0.8809 | 0.8642 |
| 1173 | 0.8624 | 0.9302 | 0.9805 | 0.8175 | 0.6814 | 0.9701 | 0.9690 | 0.8319 | 0.8811 | 0.8644 |
| 1174 | 0.8642 | 0.9302 | 0.9805 | 0.8140 | 0.6795 | 0.9701 | 0.9690 | 0.8319 | 0.8807 | 0.8639 |
| 1175 | 0.8624 | 0.9302 | 0.9805 | 0.8157 | 0.6814 | 0.9720 | 0.9690 | 0.8319 | 0.8811 | 0.8644 |
| 1176 | 0.8642 | 0.9302 | 0.9805 | 0.8157 | 0.6814 | 0.9720 | 0.9690 | 0.8319 | 0.8813 | 0.8647 |
| 1177 | 0.8624 | 0.9302 | 0.9805 | 0.8157 | 0.6833 | 0.9720 | 0.9673 | 0.8319 | 0.8811 | 0.8644 |
| 1178 | 0.8642 | 0.9302 | 0.9805 | 0.8157 | 0.6833 | 0.9720 | 0.9673 | 0.8319 | 0.8813 | 0.8647 |
| 1179 | 0.8642 | 0.9302 | 0.9805 | 0.8157 | 0.6833 | 0.9720 | 0.9673 | 0.8319 | 0.8813 | 0.8647 |
| 1180 | 0.8624 | 0.9302 | 0.9785 | 0.8104 | 0.6833 | 0.9720 | 0.9673 | 0.8301 | 0.8800 | 0.8631 |
| 1181 | 0.8607 | 0.9302 | 0.9785 | 0.8086 | 0.6833 | 0.9720 | 0.9673 | 0.8301 | 0.8795 | 0.8626 |
| 1182 | 0.8589 | 0.9302 | 0.9785 | 0.8104 | 0.6833 | 0.9720 | 0.9673 | 0.8301 | 0.8795 | 0.8626 |
| 1183 | 0.8589 | 0.9302 | 0.9785 | 0.8104 | 0.6833 | 0.9701 | 0.9673 | 0.8301 | 0.8793 | 0.8624 |
| 1184 | 0.8624 | 0.9302 | 0.9785 | 0.8086 | 0.6833 | 0.9701 | 0.9690 | 0.8301 | 0.8798 | 0.8629 |
| 1185 | 0.8624 | 0.9302 | 0.9785 | 0.8086 | 0.6852 | 0.9701 | 0.9690 | 0.8301 | 0.8800 | 0.8631 |
| 1186 | 0.8624 | 0.9302 | 0.9785 | 0.8086 | 0.6833 | 0.9701 | 0.9690 | 0.8301 | 0.8798 | 0.8629 |
| 1187 | 0.8624 | 0.9302 | 0.9785 | 0.8086 | 0.6833 | 0.9682 | 0.9690 | 0.8301 | 0.8795 | 0.8626 |
| 1188 | 0.8624 | 0.9285 | 0.9805 | 0.8086 | 0.6833 | 0.9701 | 0.9690 | 0.8301 | 0.8798 | 0.8629 |
| 1189 | 0.8624 | 0.9285 | 0.9805 | 0.8086 | 0.6833 | 0.9682 | 0.9690 | 0.8301 | 0.8795 | 0.8626 |
| 1190 | 0.8624 | 0.9285 | 0.9805 | 0.8068 | 0.6833 | 0.9701 | 0.9690 | 0.8301 | 0.8795 | 0.8626 |
| 1191 | 0.8660 | 0.9285 | 0.9805 | 0.8104 | 0.6833 | 0.9701 | 0.9690 | 0.8301 | 0.8804 | 0.8637 |
| 1192 | 0.8642 | 0.9285 | 0.9805 | 0.8104 | 0.6833 | 0.9701 | 0.9690 | 0.8301 | 0.8802 | 0.8634 |
| 1193 | 0.8624 | 0.9285 | 0.9805 | 0.8068 | 0.6814 | 0.9701 | 0.9690 | 0.8301 | 0.8793 | 0.8624 |
| 1194 | 0.8624 | 0.9285 | 0.9805 | 0.8050 | 0.6814 | 0.9701 | 0.9690 | 0.8301 | 0.8791 | 0.8621 |
| 1195 | 0.8624 | 0.9285 | 0.9805 | 0.8050 | 0.6814 | 0.9701 | 0.9690 | 0.8301 | 0.8791 | 0.8621 |
| 1196 | 0.8642 | 0.9285 | 0.9805 | 0.8050 | 0.6814 | 0.9701 | 0.9690 | 0.8301 | 0.8793 | 0.8624 |
| 1197 | 0.8642 | 0.9285 | 0.9805 | 0.8068 | 0.6814 | 0.9701 | 0.9690 | 0.8301 | 0.8795 | 0.8626 |
| 1198 | 0.8642 | 0.9302 | 0.9805 | 0.8068 | 0.6814 | 0.9682 | 0.9690 | 0.8301 | 0.8795 | 0.8626 |
| 1199 | 0.8624 | 0.9302 | 0.9805 | 0.8068 | 0.6833 | 0.9682 | 0.9690 | 0.8301 | 0.8795 | 0.8626 |
| 1200 | 0.8624 | 0.9302 | 0.9805 | 0.8086 | 0.6814 | 0.9682 | 0.9690 | 0.8319 | 0.8798 | 0.8629 |
| 1201 | 0.8624 | 0.9302 | 0.9805 | 0.8086 | 0.6833 | 0.9682 | 0.9690 | 0.8336 | 0.8802 | 0.8634 |
| 1202 | 0.8624 | 0.9302 | 0.9805 | 0.8086 | 0.6795 | 0.9682 | 0.9690 | 0.8336 | 0.8798 | 0.8629 |
| 1203 | 0.8624 | 0.9302 | 0.9805 | 0.8086 | 0.6795 | 0.9682 | 0.9690 | 0.8354 | 0.8800 | 0.8631 |
| 1204 | 0.8607 | 0.9302 | 0.9805 | 0.8086 | 0.6795 | 0.9682 | 0.9690 | 0.8354 | 0.8798 | 0.8629 |
| 1205 | 0.8607 | 0.9302 | 0.9805 | 0.8086 | 0.6795 | 0.9682 | 0.9690 | 0.8354 | 0.8798 | 0.8629 |
| 1206 | 0.8607 | 0.9285 | 0.9805 | 0.8068 | 0.6795 | 0.9682 | 0.9690 | 0.8354 | 0.8793 | 0.8624 |
| 1207 | 0.8607 | 0.9285 | 0.9805 | 0.8068 | 0.6795 | 0.9682 | 0.9690 | 0.8354 | 0.8793 | 0.8624 |
| 1208 | 0.8607 | 0.9302 | 0.9805 | 0.8068 | 0.6795 | 0.9682 | 0.9690 | 0.8354 | 0.8795 | 0.8626 |
| 1209 | 0.8589 | 0.9302 | 0.9805 | 0.8086 | 0.6795 | 0.9682 | 0.9690 | 0.8354 | 0.8795 | 0.8626 |
| 1210 | 0.8571 | 0.9302 | 0.9805 | 0.8068 | 0.6795 | 0.9682 | 0.9690 | 0.8354 | 0.8791 | 0.8621 |
| 1211 | 0.8571 | 0.9302 | 0.9805 | 0.8086 | 0.6795 | 0.9682 | 0.9690 | 0.8354 | 0.8793 | 0.8624 |
| 1212 | 0.8571 | 0.9302 | 0.9805 | 0.8086 | 0.6795 | 0.9682 | 0.9690 | 0.8354 | 0.8793 | 0.8624 |
| 1213 | 0.8571 | 0.9302 | 0.9805 | 0.8086 | 0.6795 | 0.9682 | 0.9690 | 0.8354 | 0.8793 | 0.8624 |
| 1214 | 0.8571 | 0.9285 | 0.9805 | 0.8086 | 0.6814 | 0.9682 | 0.9690 | 0.8354 | 0.8793 | 0.8624 |
| 1215 | 0.8571 | 0.9285 | 0.9805 | 0.8086 | 0.6814 | 0.9682 | 0.9690 | 0.8354 | 0.8793 | 0.8624 |
| 1216 | 0.8571 | 0.9285 | 0.9805 | 0.8086 | 0.6814 | 0.9682 | 0.9690 | 0.8354 | 0.8793 | 0.8624 |
| 1217 | 0.8571 | 0.9285 | 0.9785 | 0.8086 | 0.6795 | 0.9682 | 0.9690 | 0.8354 | 0.8789 | 0.8618 |
| 1218 | 0.8571 | 0.9285 | 0.9785 | 0.8086 | 0.6814 | 0.9682 | 0.9690 | 0.8354 | 0.8791 | 0.8621 |
| 1219 | 0.8571 | 0.9285 | 0.9785 | 0.8086 | 0.6814 | 0.9682 | 0.9690 | 0.8354 | 0.8791 | 0.8621 |
| 1220 | 0.8571 | 0.9285 | 0.9805 | 0.8086 | 0.6833 | 0.9682 | 0.9690 | 0.8354 | 0.8795 | 0.8626 |
| 1221 | 0.8607 | 0.9285 | 0.9805 | 0.8086 | 0.6814 | 0.9682 | 0.9690 | 0.8354 | 0.8798 | 0.8629 |
| 1222 | 0.8607 | 0.9302 | 0.9805 | 0.8086 | 0.6814 | 0.9682 | 0.9690 | 0.8354 | 0.8800 | 0.8631 |
| 1223 | 0.8607 | 0.9302 | 0.9805 | 0.8086 | 0.6795 | 0.9682 | 0.9690 | 0.8354 | 0.8798 | 0.8629 |
| 1224 | 0.8607 | 0.9302 | 0.9805 | 0.8086 | 0.6795 | 0.9682 | 0.9690 | 0.8354 | 0.8798 | 0.8629 |
| 1225 | 0.8554 | 0.9302 | 0.9805 | 0.8086 | 0.6795 | 0.9682 | 0.9690 | 0.8354 | 0.8791 | 0.8621 |
| 1226 | 0.8536 | 0.9302 | 0.9805 | 0.8086 | 0.6795 | 0.9682 | 0.9690 | 0.8336 | 0.8786 | 0.8616 |
| 1227 | 0.8571 | 0.9302 | 0.9805 | 0.8086 | 0.6795 | 0.9682 | 0.9690 | 0.8336 | 0.8791 | 0.8621 |
| 1228 | 0.8554 | 0.9302 | 0.9805 | 0.8086 | 0.6814 | 0.9682 | 0.9690 | 0.8336 | 0.8791 | 0.8621 |
| 1229 | 0.8554 | 0.9302 | 0.9805 | 0.8086 | 0.6814 | 0.9682 | 0.9690 | 0.8336 | 0.8791 | 0.8621 |
| 1230 | 0.8571 | 0.9302 | 0.9805 | 0.8086 | 0.6814 | 0.9682 | 0.9690 | 0.8336 | 0.8793 | 0.8624 |
| 1231 | 0.8536 | 0.9302 | 0.9805 | 0.8086 | 0.6814 | 0.9682 | 0.9690 | 0.8336 | 0.8789 | 0.8618 |
| 1232 | 0.8536 | 0.9302 | 0.9805 | 0.8086 | 0.6833 | 0.9682 | 0.9690 | 0.8336 | 0.8791 | 0.8621 |
| 1233 | 0.8536 | 0.9302 | 0.9805 | 0.8086 | 0.6833 | 0.9701 | 0.9690 | 0.8336 | 0.8793 | 0.8624 |
| 1234 | 0.8536 | 0.9302 | 0.9805 | 0.8086 | 0.6833 | 0.9682 | 0.9690 | 0.8336 | 0.8791 | 0.8621 |
| 1235 | 0.8519 | 0.9285 | 0.9805 | 0.8086 | 0.6833 | 0.9682 | 0.9690 | 0.8336 | 0.8786 | 0.8616 |
| 1236 | 0.8519 | 0.9285 | 0.9805 | 0.8086 | 0.6833 | 0.9682 | 0.9690 | 0.8336 | 0.8786 | 0.8616 |
| 1237 | 0.8519 | 0.9285 | 0.9805 | 0.8086 | 0.6833 | 0.9682 | 0.9690 | 0.8354 | 0.8789 | 0.8618 |
| 1238 | 0.8519 | 0.9285 | 0.9805 | 0.8086 | 0.6833 | 0.9682 | 0.9690 | 0.8354 | 0.8789 | 0.8618 |
| 1239 | 0.8519 | 0.9285 | 0.9805 | 0.8086 | 0.6852 | 0.9682 | 0.9690 | 0.8354 | 0.8791 | 0.8621 |
| 1240 | 0.8519 | 0.9285 | 0.9805 | 0.8086 | 0.6852 | 0.9682 | 0.9690 | 0.8354 | 0.8791 | 0.8621 |
| 1241 | 0.8519 | 0.9285 | 0.9805 | 0.8086 | 0.6852 | 0.9682 | 0.9690 | 0.8354 | 0.8791 | 0.8621 |
| 1242 | 0.8536 | 0.9285 | 0.9805 | 0.8086 | 0.6852 | 0.9682 | 0.9690 | 0.8354 | 0.8793 | 0.8624 |
| 1243 | 0.8536 | 0.9267 | 0.9805 | 0.8086 | 0.6852 | 0.9682 | 0.9690 | 0.8354 | 0.8791 | 0.8621 |
| 1244 | 0.8536 | 0.9267 | 0.9805 | 0.8086 | 0.6852 | 0.9682 | 0.9690 | 0.8354 | 0.8791 | 0.8621 |
| 1245 | 0.8519 | 0.9267 | 0.9805 | 0.8068 | 0.6852 | 0.9682 | 0.9690 | 0.8354 | 0.8786 | 0.8616 |
| 1246 | 0.8519 | 0.9267 | 0.9805 | 0.8068 | 0.6852 | 0.9682 | 0.9690 | 0.8354 | 0.8786 | 0.8616 |
| 1247 | 0.8501 | 0.9267 | 0.9805 | 0.8068 | 0.6852 | 0.9682 | 0.9690 | 0.8354 | 0.8784 | 0.8613 |
| 1248 | 0.8501 | 0.9267 | 0.9805 | 0.8068 | 0.6833 | 0.9682 | 0.9690 | 0.8354 | 0.8782 | 0.8611 |
| 1249 | 0.8501 | 0.9267 | 0.9805 | 0.8068 | 0.6833 | 0.9682 | 0.9690 | 0.8354 | 0.8782 | 0.8611 |
| 1250 | 0.8501 | 0.9267 | 0.9805 | 0.8086 | 0.6852 | 0.9682 | 0.9690 | 0.8354 | 0.8786 | 0.8616 |
| 1251 | 0.8466 | 0.9267 | 0.9785 | 0.8104 | 0.6871 | 0.9682 | 0.9690 | 0.8319 | 0.8780 | 0.8608 |
| 1252 | 0.8483 | 0.9267 | 0.9785 | 0.8104 | 0.6852 | 0.9682 | 0.9690 | 0.8301 | 0.8777 | 0.8606 |
| 1253 | 0.8448 | 0.9267 | 0.9785 | 0.8104 | 0.6852 | 0.9682 | 0.9690 | 0.8301 | 0.8773 | 0.8600 |
| 1254 | 0.8448 | 0.9267 | 0.9785 | 0.8104 | 0.6852 | 0.9682 | 0.9690 | 0.8301 | 0.8773 | 0.8600 |
| 1255 | 0.8448 | 0.9267 | 0.9785 | 0.8104 | 0.6891 | 0.9682 | 0.9690 | 0.8301 | 0.8777 | 0.8606 |
| 1256 | 0.8448 | 0.9267 | 0.9785 | 0.8104 | 0.6891 | 0.9682 | 0.9690 | 0.8301 | 0.8777 | 0.8606 |
| 1257 | 0.8466 | 0.9267 | 0.9785 | 0.8104 | 0.6891 | 0.9682 | 0.9690 | 0.8301 | 0.8780 | 0.8608 |
| 1258 | 0.8466 | 0.9267 | 0.9785 | 0.8104 | 0.6891 | 0.9682 | 0.9690 | 0.8301 | 0.8780 | 0.8608 |
| 1259 | 0.8448 | 0.9267 | 0.9785 | 0.8104 | 0.6891 | 0.9682 | 0.9690 | 0.8301 | 0.8777 | 0.8606 |
| 1260 | 0.8466 | 0.9267 | 0.9785 | 0.8104 | 0.6871 | 0.9682 | 0.9690 | 0.8301 | 0.8777 | 0.8606 |
| 1261 | 0.8466 | 0.9267 | 0.9785 | 0.8104 | 0.6891 | 0.9682 | 0.9690 | 0.8284 | 0.8777 | 0.8606 |
| 1262 | 0.8448 | 0.9267 | 0.9785 | 0.8104 | 0.6891 | 0.9682 | 0.9690 | 0.8284 | 0.8775 | 0.8603 |
| 1263 | 0.8448 | 0.9267 | 0.9785 | 0.8122 | 0.6891 | 0.9682 | 0.9690 | 0.8266 | 0.8775 | 0.8603 |
| 1264 | 0.8448 | 0.9267 | 0.9785 | 0.8122 | 0.6910 | 0.9682 | 0.9690 | 0.8266 | 0.8777 | 0.8606 |
| 1265 | 0.8448 | 0.9267 | 0.9785 | 0.8122 | 0.6910 | 0.9682 | 0.9690 | 0.8284 | 0.8780 | 0.8608 |
| 1266 | 0.8448 | 0.9267 | 0.9785 | 0.8140 | 0.6910 | 0.9682 | 0.9690 | 0.8284 | 0.8782 | 0.8611 |
| 1267 | 0.8430 | 0.9267 | 0.9785 | 0.8140 | 0.6929 | 0.9682 | 0.9690 | 0.8284 | 0.8782 | 0.8611 |
| 1268 | 0.8430 | 0.9267 | 0.9785 | 0.8122 | 0.6929 | 0.9682 | 0.9690 | 0.8284 | 0.8780 | 0.8608 |
| 1269 | 0.8466 | 0.9285 | 0.9785 | 0.8122 | 0.6929 | 0.9682 | 0.9690 | 0.8284 | 0.8786 | 0.8616 |
| 1270 | 0.8466 | 0.9285 | 0.9785 | 0.8122 | 0.6929 | 0.9682 | 0.9690 | 0.8284 | 0.8786 | 0.8616 |
| 1271 | 0.8466 | 0.9285 | 0.9805 | 0.8104 | 0.6929 | 0.9682 | 0.9690 | 0.8266 | 0.8784 | 0.8613 |
| 1272 | 0.8466 | 0.9285 | 0.9785 | 0.8104 | 0.6929 | 0.9682 | 0.9690 | 0.8266 | 0.8782 | 0.8611 |
| 1273 | 0.8466 | 0.9285 | 0.9785 | 0.8104 | 0.6929 | 0.9682 | 0.9690 | 0.8266 | 0.8782 | 0.8611 |
| 1274 | 0.8448 | 0.9267 | 0.9785 | 0.8068 | 0.6910 | 0.9682 | 0.9690 | 0.8266 | 0.8771 | 0.8598 |
| 1275 | 0.8448 | 0.9267 | 0.9785 | 0.8068 | 0.6910 | 0.9682 | 0.9690 | 0.8266 | 0.8771 | 0.8598 |
| 1276 | 0.8430 | 0.9285 | 0.9785 | 0.8086 | 0.6910 | 0.9682 | 0.9690 | 0.8266 | 0.8773 | 0.8600 |
| 1277 | 0.8430 | 0.9285 | 0.9785 | 0.8086 | 0.6910 | 0.9682 | 0.9690 | 0.8266 | 0.8773 | 0.8600 |
| 1278 | 0.8430 | 0.9285 | 0.9785 | 0.8086 | 0.6910 | 0.9682 | 0.9690 | 0.8266 | 0.8773 | 0.8600 |
| 1279 | 0.8413 | 0.9285 | 0.9785 | 0.8086 | 0.6910 | 0.9682 | 0.9690 | 0.8266 | 0.8771 | 0.8598 |
| 1280 | 0.8413 | 0.9285 | 0.9785 | 0.8086 | 0.6910 | 0.9682 | 0.9690 | 0.8284 | 0.8773 | 0.8600 |
| 1281 | 0.8413 | 0.9285 | 0.9785 | 0.8104 | 0.6910 | 0.9682 | 0.9690 | 0.8284 | 0.8775 | 0.8603 |
| 1282 | 0.8413 | 0.9285 | 0.9785 | 0.8104 | 0.6891 | 0.9682 | 0.9690 | 0.8284 | 0.8773 | 0.8600 |
| 1283 | 0.8413 | 0.9285 | 0.9785 | 0.8104 | 0.6891 | 0.9682 | 0.9690 | 0.8284 | 0.8773 | 0.8600 |
| 1284 | 0.8413 | 0.9285 | 0.9785 | 0.8104 | 0.6871 | 0.9682 | 0.9690 | 0.8284 | 0.8771 | 0.8598 |
| 1285 | 0.8413 | 0.9302 | 0.9785 | 0.8104 | 0.6871 | 0.9701 | 0.9690 | 0.8301 | 0.8777 | 0.8605 |
| 1286 | 0.8413 | 0.9302 | 0.9785 | 0.8104 | 0.6871 | 0.9701 | 0.9690 | 0.8301 | 0.8777 | 0.8605 |
| 1287 | 0.8413 | 0.9302 | 0.9785 | 0.8104 | 0.6871 | 0.9701 | 0.9690 | 0.8301 | 0.8777 | 0.8605 |
| 1288 | 0.8413 | 0.9302 | 0.9785 | 0.8104 | 0.6871 | 0.9701 | 0.9690 | 0.8301 | 0.8777 | 0.8605 |
| 1289 | 0.8430 | 0.9302 | 0.9805 | 0.8104 | 0.6891 | 0.9701 | 0.9690 | 0.8301 | 0.8784 | 0.8613 |
| 1290 | 0.8395 | 0.9302 | 0.9805 | 0.8104 | 0.6891 | 0.9701 | 0.9690 | 0.8301 | 0.8780 | 0.8608 |
| 1291 | 0.8395 | 0.9302 | 0.9805 | 0.8104 | 0.6891 | 0.9701 | 0.9690 | 0.8301 | 0.8780 | 0.8608 |
| 1292 | 0.8413 | 0.9302 | 0.9785 | 0.8104 | 0.6891 | 0.9701 | 0.9690 | 0.8301 | 0.8780 | 0.8608 |
| 1293 | 0.8413 | 0.9302 | 0.9785 | 0.8104 | 0.6891 | 0.9701 | 0.9690 | 0.8301 | 0.8780 | 0.8608 |
| 1294 | 0.8413 | 0.9302 | 0.9766 | 0.8104 | 0.6891 | 0.9701 | 0.9690 | 0.8301 | 0.8777 | 0.8605 |
| 1295 | 0.8413 | 0.9302 | 0.9766 | 0.8104 | 0.6891 | 0.9701 | 0.9690 | 0.8301 | 0.8777 | 0.8605 |
| 1296 | 0.8413 | 0.9302 | 0.9766 | 0.8104 | 0.6891 | 0.9701 | 0.9690 | 0.8301 | 0.8777 | 0.8605 |
| 1297 | 0.8430 | 0.9302 | 0.9766 | 0.8104 | 0.6910 | 0.9701 | 0.9690 | 0.8301 | 0.8782 | 0.8611 |
| 1298 | 0.8430 | 0.9302 | 0.9766 | 0.8086 | 0.6910 | 0.9701 | 0.9690 | 0.8319 | 0.8782 | 0.8611 |
| 1299 | 0.8430 | 0.9302 | 0.9766 | 0.8086 | 0.6929 | 0.9701 | 0.9690 | 0.8319 | 0.8784 | 0.8613 |
| 1300 | 0.8430 | 0.9302 | 0.9766 | 0.8086 | 0.6929 | 0.9701 | 0.9690 | 0.8319 | 0.8784 | 0.8613 |
| 1301 | 0.8430 | 0.9302 | 0.9785 | 0.8086 | 0.6910 | 0.9701 | 0.9690 | 0.8319 | 0.8784 | 0.8613 |
| 1302 | 0.8430 | 0.9302 | 0.9785 | 0.8086 | 0.6910 | 0.9682 | 0.9690 | 0.8319 | 0.8782 | 0.8610 |
| 1303 | 0.8430 | 0.9302 | 0.9785 | 0.8086 | 0.6891 | 0.9682 | 0.9690 | 0.8319 | 0.8780 | 0.8608 |
| 1304 | 0.8448 | 0.9302 | 0.9785 | 0.8086 | 0.6910 | 0.9701 | 0.9690 | 0.8319 | 0.8786 | 0.8616 |
| 1305 | 0.8448 | 0.9302 | 0.9785 | 0.8086 | 0.6910 | 0.9701 | 0.9690 | 0.8319 | 0.8786 | 0.8616 |
| 1306 | 0.8448 | 0.9302 | 0.9785 | 0.8086 | 0.6929 | 0.9701 | 0.9690 | 0.8319 | 0.8789 | 0.8618 |
| 1307 | 0.8448 | 0.9302 | 0.9785 | 0.8086 | 0.6948 | 0.9701 | 0.9690 | 0.8319 | 0.8791 | 0.8621 |
| 1308 | 0.8430 | 0.9302 | 0.9785 | 0.8086 | 0.6948 | 0.9701 | 0.9690 | 0.8319 | 0.8789 | 0.8618 |
| 1309 | 0.8430 | 0.9302 | 0.9785 | 0.8086 | 0.6948 | 0.9701 | 0.9690 | 0.8319 | 0.8789 | 0.8618 |
| 1310 | 0.8448 | 0.9302 | 0.9785 | 0.8086 | 0.6948 | 0.9701 | 0.9690 | 0.8319 | 0.8791 | 0.8621 |
| 1311 | 0.8448 | 0.9302 | 0.9785 | 0.8086 | 0.6948 | 0.9701 | 0.9690 | 0.8301 | 0.8789 | 0.8618 |
| 1312 | 0.8448 | 0.9302 | 0.9785 | 0.8086 | 0.6948 | 0.9701 | 0.9690 | 0.8301 | 0.8789 | 0.8618 |
| 1313 | 0.8448 | 0.9302 | 0.9805 | 0.8086 | 0.6948 | 0.9701 | 0.9690 | 0.8284 | 0.8789 | 0.8618 |
| 1314 | 0.8448 | 0.9302 | 0.9785 | 0.8068 | 0.6948 | 0.9701 | 0.9690 | 0.8284 | 0.8784 | 0.8613 |
| 1315 | 0.8448 | 0.9302 | 0.9785 | 0.8068 | 0.6948 | 0.9701 | 0.9690 | 0.8301 | 0.8786 | 0.8616 |
| 1316 | 0.8448 | 0.9302 | 0.9766 | 0.8050 | 0.6948 | 0.9701 | 0.9690 | 0.8301 | 0.8782 | 0.8610 |
| 1317 | 0.8466 | 0.9302 | 0.9785 | 0.8068 | 0.6967 | 0.9701 | 0.9690 | 0.8301 | 0.8791 | 0.8621 |
| 1318 | 0.8466 | 0.9302 | 0.9785 | 0.8068 | 0.6967 | 0.9701 | 0.9690 | 0.8301 | 0.8791 | 0.8621 |
| 1319 | 0.8466 | 0.9302 | 0.9785 | 0.8050 | 0.6967 | 0.9701 | 0.9690 | 0.8301 | 0.8789 | 0.8618 |
| 1320 | 0.8466 | 0.9302 | 0.9766 | 0.8086 | 0.6967 | 0.9701 | 0.9690 | 0.8301 | 0.8791 | 0.8621 |
| 1321 | 0.8448 | 0.9302 | 0.9766 | 0.8086 | 0.6967 | 0.9701 | 0.9707 | 0.8301 | 0.8791 | 0.8621 |
| 1322 | 0.8448 | 0.9302 | 0.9766 | 0.8122 | 0.6967 | 0.9682 | 0.9707 | 0.8301 | 0.8793 | 0.8623 |
| 1323 | 0.8430 | 0.9302 | 0.9766 | 0.8122 | 0.6948 | 0.9701 | 0.9707 | 0.8301 | 0.8791 | 0.8621 |
| 1324 | 0.8413 | 0.9302 | 0.9785 | 0.8104 | 0.6929 | 0.9701 | 0.9707 | 0.8301 | 0.8786 | 0.8616 |
| 1325 | 0.8413 | 0.9302 | 0.9785 | 0.8104 | 0.6948 | 0.9701 | 0.9707 | 0.8301 | 0.8789 | 0.8618 |
| 1326 | 0.8413 | 0.9302 | 0.9805 | 0.8104 | 0.6948 | 0.9701 | 0.9707 | 0.8301 | 0.8791 | 0.8621 |
| 1327 | 0.8413 | 0.9302 | 0.9805 | 0.8104 | 0.6948 | 0.9701 | 0.9690 | 0.8301 | 0.8789 | 0.8618 |
| 1328 | 0.8395 | 0.9302 | 0.9805 | 0.8104 | 0.6929 | 0.9701 | 0.9690 | 0.8301 | 0.8784 | 0.8613 |
| 1329 | 0.8377 | 0.9302 | 0.9766 | 0.8086 | 0.6948 | 0.9701 | 0.9690 | 0.8284 | 0.8775 | 0.8603 |
| 1330 | 0.8395 | 0.9302 | 0.9766 | 0.8086 | 0.6948 | 0.9701 | 0.9690 | 0.8284 | 0.8777 | 0.8605 |
| 1331 | 0.8377 | 0.9302 | 0.9766 | 0.8086 | 0.6948 | 0.9701 | 0.9690 | 0.8284 | 0.8775 | 0.8603 |
| 1332 | 0.8377 | 0.9302 | 0.9766 | 0.8086 | 0.6948 | 0.9701 | 0.9690 | 0.8284 | 0.8775 | 0.8603 |
| 1333 | 0.8395 | 0.9302 | 0.9766 | 0.8104 | 0.6948 | 0.9701 | 0.9690 | 0.8284 | 0.8780 | 0.8608 |
| 1334 | 0.8395 | 0.9302 | 0.9766 | 0.8104 | 0.6948 | 0.9701 | 0.9707 | 0.8284 | 0.8782 | 0.8611 |
| 1335 | 0.8395 | 0.9285 | 0.9766 | 0.8104 | 0.6948 | 0.9701 | 0.9690 | 0.8284 | 0.8777 | 0.8605 |
| 1336 | 0.8395 | 0.9285 | 0.9746 | 0.8104 | 0.6910 | 0.9701 | 0.9690 | 0.8284 | 0.8771 | 0.8598 |
| 1337 | 0.8395 | 0.9285 | 0.9746 | 0.8104 | 0.6910 | 0.9701 | 0.9690 | 0.8284 | 0.8771 | 0.8598 |
| 1338 | 0.8395 | 0.9285 | 0.9746 | 0.8104 | 0.6910 | 0.9701 | 0.9690 | 0.8284 | 0.8771 | 0.8598 |
| 1339 | 0.8395 | 0.9285 | 0.9746 | 0.8104 | 0.6910 | 0.9701 | 0.9690 | 0.8266 | 0.8768 | 0.8595 |
| 1340 | 0.8395 | 0.9285 | 0.9746 | 0.8104 | 0.6910 | 0.9701 | 0.9690 | 0.8266 | 0.8768 | 0.8595 |
| 1341 | 0.8377 | 0.9285 | 0.9746 | 0.8086 | 0.6891 | 0.9701 | 0.9690 | 0.8266 | 0.8762 | 0.8587 |
| 1342 | 0.8395 | 0.9285 | 0.9746 | 0.8140 | 0.6891 | 0.9701 | 0.9690 | 0.8266 | 0.8771 | 0.8598 |
| 1343 | 0.8395 | 0.9285 | 0.9746 | 0.8140 | 0.6891 | 0.9701 | 0.9690 | 0.8266 | 0.8771 | 0.8598 |
| 1344 | 0.8395 | 0.9285 | 0.9746 | 0.8140 | 0.6910 | 0.9701 | 0.9690 | 0.8266 | 0.8773 | 0.8600 |
| 1345 | 0.8395 | 0.9285 | 0.9746 | 0.8140 | 0.6910 | 0.9701 | 0.9690 | 0.8266 | 0.8773 | 0.8600 |
| 1346 | 0.8395 | 0.9285 | 0.9746 | 0.8140 | 0.6910 | 0.9701 | 0.9690 | 0.8266 | 0.8773 | 0.8600 |
| 1347 | 0.8395 | 0.9285 | 0.9746 | 0.8140 | 0.6910 | 0.9701 | 0.9690 | 0.8266 | 0.8773 | 0.8600 |
| 1348 | 0.8395 | 0.9285 | 0.9766 | 0.8140 | 0.6910 | 0.9701 | 0.9690 | 0.8266 | 0.8775 | 0.8603 |
| 1349 | 0.8395 | 0.9285 | 0.9746 | 0.8140 | 0.6910 | 0.9701 | 0.9707 | 0.8266 | 0.8775 | 0.8603 |
| 1350 | 0.8430 | 0.9285 | 0.9746 | 0.8122 | 0.6910 | 0.9701 | 0.9707 | 0.8266 | 0.8777 | 0.8606 |
| 1351 | 0.8430 | 0.9285 | 0.9746 | 0.8122 | 0.6910 | 0.9701 | 0.9707 | 0.8266 | 0.8777 | 0.8606 |
| 1352 | 0.8430 | 0.9285 | 0.9746 | 0.8104 | 0.6910 | 0.9701 | 0.9707 | 0.8266 | 0.8775 | 0.8603 |
| 1353 | 0.8430 | 0.9285 | 0.9746 | 0.8104 | 0.6910 | 0.9701 | 0.9707 | 0.8266 | 0.8775 | 0.8603 |
| 1354 | 0.8430 | 0.9285 | 0.9746 | 0.8104 | 0.6910 | 0.9701 | 0.9707 | 0.8231 | 0.8771 | 0.8598 |
| 1355 | 0.8395 | 0.9285 | 0.9746 | 0.8104 | 0.6948 | 0.9701 | 0.9707 | 0.8231 | 0.8771 | 0.8598 |
| 1356 | 0.8395 | 0.9285 | 0.9746 | 0.8104 | 0.6929 | 0.9701 | 0.9707 | 0.8231 | 0.8768 | 0.8595 |
| 1357 | 0.8377 | 0.9285 | 0.9746 | 0.8086 | 0.6910 | 0.9701 | 0.9707 | 0.8231 | 0.8762 | 0.8587 |
| 1358 | 0.8395 | 0.9285 | 0.9746 | 0.8086 | 0.6929 | 0.9701 | 0.9707 | 0.8231 | 0.8766 | 0.8593 |
| 1359 | 0.8395 | 0.9285 | 0.9746 | 0.8086 | 0.6891 | 0.9682 | 0.9707 | 0.8231 | 0.8759 | 0.8585 |
| 1360 | 0.8395 | 0.9285 | 0.9746 | 0.8086 | 0.6891 | 0.9682 | 0.9707 | 0.8214 | 0.8757 | 0.8582 |
| 1361 | 0.8395 | 0.9285 | 0.9746 | 0.8086 | 0.6891 | 0.9682 | 0.9707 | 0.8214 | 0.8757 | 0.8582 |
| 1362 | 0.8395 | 0.9285 | 0.9746 | 0.8104 | 0.6929 | 0.9682 | 0.9707 | 0.8214 | 0.8764 | 0.8590 |
| 1363 | 0.8395 | 0.9285 | 0.9746 | 0.8068 | 0.6948 | 0.9682 | 0.9707 | 0.8214 | 0.8762 | 0.8587 |
| 1364 | 0.8395 | 0.9285 | 0.9746 | 0.8068 | 0.6948 | 0.9682 | 0.9707 | 0.8214 | 0.8762 | 0.8587 |
| 1365 | 0.8377 | 0.9285 | 0.9746 | 0.8068 | 0.6948 | 0.9682 | 0.9707 | 0.8214 | 0.8759 | 0.8585 |
| 1366 | 0.8377 | 0.9285 | 0.9746 | 0.8050 | 0.6948 | 0.9664 | 0.9707 | 0.8214 | 0.8755 | 0.8580 |
| 1367 | 0.8377 | 0.9285 | 0.9746 | 0.8050 | 0.6948 | 0.9664 | 0.9707 | 0.8214 | 0.8755 | 0.8580 |
| 1368 | 0.8360 | 0.9285 | 0.9746 | 0.8050 | 0.6948 | 0.9664 | 0.9707 | 0.8214 | 0.8753 | 0.8577 |
| 1369 | 0.8360 | 0.9285 | 0.9746 | 0.8050 | 0.6929 | 0.9664 | 0.9707 | 0.8214 | 0.8750 | 0.8574 |
| 1370 | 0.8360 | 0.9285 | 0.9746 | 0.8050 | 0.6948 | 0.9664 | 0.9707 | 0.8214 | 0.8753 | 0.8577 |
| 1371 | 0.8360 | 0.9285 | 0.9746 | 0.8050 | 0.6948 | 0.9664 | 0.9707 | 0.8214 | 0.8753 | 0.8577 |
| 1372 | 0.8360 | 0.9285 | 0.9746 | 0.8050 | 0.6929 | 0.9664 | 0.9707 | 0.8214 | 0.8750 | 0.8574 |
| 1373 | 0.8360 | 0.9285 | 0.9746 | 0.8050 | 0.6929 | 0.9664 | 0.9707 | 0.8214 | 0.8750 | 0.8574 |
| 1374 | 0.8360 | 0.9285 | 0.9746 | 0.8050 | 0.6929 | 0.9664 | 0.9707 | 0.8214 | 0.8750 | 0.8574 |
| 1375 | 0.8360 | 0.9285 | 0.9746 | 0.8068 | 0.6967 | 0.9664 | 0.9707 | 0.8214 | 0.8757 | 0.8582 |
| 1376 | 0.8360 | 0.9285 | 0.9746 | 0.8068 | 0.6967 | 0.9664 | 0.9707 | 0.8214 | 0.8757 | 0.8582 |
| 1377 | 0.8360 | 0.9285 | 0.9746 | 0.8068 | 0.6967 | 0.9664 | 0.9707 | 0.8214 | 0.8757 | 0.8582 |
| 1378 | 0.8360 | 0.9285 | 0.9746 | 0.8068 | 0.6948 | 0.9664 | 0.9707 | 0.8214 | 0.8755 | 0.8580 |
| 1379 | 0.8377 | 0.9285 | 0.9746 | 0.8068 | 0.6967 | 0.9664 | 0.9707 | 0.8214 | 0.8759 | 0.8585 |
| 1380 | 0.8377 | 0.9285 | 0.9766 | 0.8068 | 0.6967 | 0.9664 | 0.9690 | 0.8214 | 0.8759 | 0.8585 |
| 1381 | 0.8342 | 0.9285 | 0.9766 | 0.8086 | 0.6910 | 0.9664 | 0.9690 | 0.8231 | 0.8753 | 0.8577 |
| 1382 | 0.8377 | 0.9285 | 0.9766 | 0.8068 | 0.6891 | 0.9664 | 0.9690 | 0.8196 | 0.8748 | 0.8572 |
| 1383 | 0.8377 | 0.9302 | 0.9766 | 0.8068 | 0.6891 | 0.9664 | 0.9690 | 0.8196 | 0.8750 | 0.8575 |
| 1384 | 0.8377 | 0.9302 | 0.9766 | 0.8068 | 0.6929 | 0.9664 | 0.9690 | 0.8196 | 0.8755 | 0.8580 |
| 1385 | 0.8377 | 0.9302 | 0.9766 | 0.8068 | 0.6929 | 0.9664 | 0.9690 | 0.8196 | 0.8755 | 0.8580 |
| 1386 | 0.8377 | 0.9302 | 0.9766 | 0.8068 | 0.6891 | 0.9664 | 0.9690 | 0.8196 | 0.8750 | 0.8575 |
| 1387 | 0.8377 | 0.9302 | 0.9766 | 0.8068 | 0.6891 | 0.9664 | 0.9690 | 0.8196 | 0.8750 | 0.8575 |
| 1388 | 0.8395 | 0.9302 | 0.9766 | 0.8086 | 0.6871 | 0.9664 | 0.9690 | 0.8196 | 0.8753 | 0.8577 |
| 1389 | 0.8377 | 0.9302 | 0.9766 | 0.8068 | 0.6871 | 0.9664 | 0.9690 | 0.8196 | 0.8748 | 0.8572 |
| 1390 | 0.8377 | 0.9302 | 0.9766 | 0.8104 | 0.6871 | 0.9664 | 0.9690 | 0.8196 | 0.8753 | 0.8577 |
| 1391 | 0.8377 | 0.9302 | 0.9766 | 0.8104 | 0.6871 | 0.9664 | 0.9690 | 0.8196 | 0.8753 | 0.8577 |
| 1392 | 0.8377 | 0.9302 | 0.9766 | 0.8086 | 0.6891 | 0.9664 | 0.9690 | 0.8214 | 0.8755 | 0.8580 |
| 1393 | 0.8377 | 0.9302 | 0.9766 | 0.8068 | 0.6929 | 0.9664 | 0.9690 | 0.8214 | 0.8757 | 0.8582 |
| 1394 | 0.8360 | 0.9302 | 0.9766 | 0.8068 | 0.6910 | 0.9664 | 0.9690 | 0.8214 | 0.8753 | 0.8577 |
| 1395 | 0.8360 | 0.9302 | 0.9766 | 0.8068 | 0.6910 | 0.9664 | 0.9690 | 0.8214 | 0.8753 | 0.8577 |
| 1396 | 0.8360 | 0.9302 | 0.9766 | 0.8068 | 0.6929 | 0.9664 | 0.9690 | 0.8214 | 0.8755 | 0.8580 |
| 1397 | 0.8360 | 0.9302 | 0.9766 | 0.8068 | 0.6929 | 0.9664 | 0.9690 | 0.8214 | 0.8755 | 0.8580 |
| 1398 | 0.8360 | 0.9302 | 0.9766 | 0.8068 | 0.6948 | 0.9664 | 0.9690 | 0.8214 | 0.8757 | 0.8582 |
| 1399 | 0.8360 | 0.9302 | 0.9746 | 0.8068 | 0.6948 | 0.9664 | 0.9690 | 0.8214 | 0.8755 | 0.8580 |
| 1400 | 0.8360 | 0.9302 | 0.9746 | 0.8068 | 0.6948 | 0.9664 | 0.9690 | 0.8214 | 0.8755 | 0.8580 |
| 1401 | 0.8360 | 0.9302 | 0.9746 | 0.8068 | 0.6948 | 0.9664 | 0.9690 | 0.8214 | 0.8755 | 0.8580 |
| 1402 | 0.8377 | 0.9302 | 0.9746 | 0.8068 | 0.6929 | 0.9664 | 0.9690 | 0.8214 | 0.8755 | 0.8580 |
| 1403 | 0.8360 | 0.9302 | 0.9746 | 0.8068 | 0.6929 | 0.9664 | 0.9690 | 0.8214 | 0.8753 | 0.8577 |
| 1404 | 0.8360 | 0.9302 | 0.9746 | 0.8068 | 0.6929 | 0.9664 | 0.9690 | 0.8214 | 0.8753 | 0.8577 |
| 1405 | 0.8360 | 0.9302 | 0.9746 | 0.8068 | 0.6929 | 0.9664 | 0.9690 | 0.8214 | 0.8753 | 0.8577 |
| 1406 | 0.8360 | 0.9302 | 0.9746 | 0.8068 | 0.6910 | 0.9664 | 0.9690 | 0.8214 | 0.8750 | 0.8575 |
| 1407 | 0.8360 | 0.9302 | 0.9746 | 0.8068 | 0.6929 | 0.9664 | 0.9690 | 0.8196 | 0.8750 | 0.8575 |
| 1408 | 0.8360 | 0.9285 | 0.9746 | 0.8068 | 0.6929 | 0.9664 | 0.9690 | 0.8196 | 0.8748 | 0.8572 |
| 1409 | 0.8360 | 0.9285 | 0.9746 | 0.8068 | 0.6929 | 0.9664 | 0.9690 | 0.8196 | 0.8748 | 0.8572 |
| 1410 | 0.8360 | 0.9302 | 0.9746 | 0.8086 | 0.6891 | 0.9664 | 0.9690 | 0.8196 | 0.8748 | 0.8572 |
| 1411 | 0.8360 | 0.9302 | 0.9746 | 0.8086 | 0.6891 | 0.9664 | 0.9690 | 0.8196 | 0.8748 | 0.8572 |
| 1412 | 0.8360 | 0.9302 | 0.9746 | 0.8086 | 0.6891 | 0.9664 | 0.9690 | 0.8196 | 0.8748 | 0.8572 |
| 1413 | 0.8360 | 0.9302 | 0.9746 | 0.8086 | 0.6871 | 0.9664 | 0.9690 | 0.8196 | 0.8746 | 0.8570 |
| 1414 | 0.8360 | 0.9302 | 0.9746 | 0.8086 | 0.6871 | 0.9664 | 0.9690 | 0.8196 | 0.8746 | 0.8570 |
| 1415 | 0.8360 | 0.9302 | 0.9746 | 0.8086 | 0.6871 | 0.9664 | 0.9690 | 0.8196 | 0.8746 | 0.8570 |
| 1416 | 0.8360 | 0.9302 | 0.9746 | 0.8086 | 0.6891 | 0.9664 | 0.9690 | 0.8196 | 0.8748 | 0.8572 |
| 1417 | 0.8360 | 0.9302 | 0.9746 | 0.8086 | 0.6891 | 0.9664 | 0.9690 | 0.8196 | 0.8748 | 0.8572 |
| 1418 | 0.8342 | 0.9319 | 0.9746 | 0.8068 | 0.6871 | 0.9664 | 0.9690 | 0.8179 | 0.8741 | 0.8564 |
| 1419 | 0.8342 | 0.9302 | 0.9746 | 0.8068 | 0.6871 | 0.9664 | 0.9690 | 0.8179 | 0.8739 | 0.8562 |
| 1420 | 0.8342 | 0.9285 | 0.9746 | 0.8050 | 0.6871 | 0.9664 | 0.9690 | 0.8179 | 0.8734 | 0.8557 |
| 1421 | 0.8342 | 0.9285 | 0.9746 | 0.8050 | 0.6871 | 0.9664 | 0.9690 | 0.8161 | 0.8732 | 0.8554 |
| 1422 | 0.8342 | 0.9285 | 0.9746 | 0.8050 | 0.6833 | 0.9664 | 0.9690 | 0.8161 | 0.8728 | 0.8549 |
| 1423 | 0.8342 | 0.9285 | 0.9746 | 0.8050 | 0.6833 | 0.9664 | 0.9690 | 0.8179 | 0.8730 | 0.8552 |
| 1424 | 0.8342 | 0.9285 | 0.9746 | 0.8050 | 0.6814 | 0.9664 | 0.9690 | 0.8179 | 0.8728 | 0.8549 |
| 1425 | 0.8342 | 0.9285 | 0.9746 | 0.8050 | 0.6814 | 0.9664 | 0.9690 | 0.8179 | 0.8728 | 0.8549 |
| 1426 | 0.8325 | 0.9285 | 0.9746 | 0.8050 | 0.6833 | 0.9664 | 0.9690 | 0.8179 | 0.8728 | 0.8549 |
| 1427 | 0.8325 | 0.9285 | 0.9746 | 0.8050 | 0.6833 | 0.9664 | 0.9690 | 0.8179 | 0.8728 | 0.8549 |
| 1428 | 0.8325 | 0.9285 | 0.9746 | 0.8050 | 0.6833 | 0.9664 | 0.9690 | 0.8179 | 0.8728 | 0.8549 |
| 1429 | 0.8325 | 0.9285 | 0.9746 | 0.8068 | 0.6833 | 0.9664 | 0.9690 | 0.8179 | 0.8730 | 0.8552 |
| 1430 | 0.8325 | 0.9285 | 0.9746 | 0.8068 | 0.6833 | 0.9664 | 0.9690 | 0.8179 | 0.8730 | 0.8552 |
| 1431 | 0.8360 | 0.9285 | 0.9746 | 0.8068 | 0.6833 | 0.9664 | 0.9690 | 0.8179 | 0.8734 | 0.8557 |
| 1432 | 0.8360 | 0.9285 | 0.9746 | 0.8068 | 0.6833 | 0.9664 | 0.9690 | 0.8179 | 0.8734 | 0.8557 |
| 1433 | 0.8360 | 0.9285 | 0.9746 | 0.8086 | 0.6852 | 0.9664 | 0.9690 | 0.8179 | 0.8739 | 0.8562 |
| 1434 | 0.8342 | 0.9285 | 0.9746 | 0.8086 | 0.6910 | 0.9664 | 0.9690 | 0.8179 | 0.8744 | 0.8567 |
| 1435 | 0.8342 | 0.9285 | 0.9746 | 0.8086 | 0.6891 | 0.9664 | 0.9690 | 0.8179 | 0.8741 | 0.8564 |
| 1436 | 0.8342 | 0.9285 | 0.9746 | 0.8086 | 0.6910 | 0.9664 | 0.9690 | 0.8179 | 0.8744 | 0.8567 |
| 1437 | 0.8342 | 0.9285 | 0.9746 | 0.8068 | 0.6910 | 0.9664 | 0.9690 | 0.8179 | 0.8741 | 0.8564 |
| 1438 | 0.8325 | 0.9285 | 0.9746 | 0.8086 | 0.6891 | 0.9664 | 0.9690 | 0.8179 | 0.8739 | 0.8562 |
| 1439 | 0.8325 | 0.9285 | 0.9746 | 0.8104 | 0.6891 | 0.9664 | 0.9690 | 0.8179 | 0.8741 | 0.8564 |
| 1440 | 0.8325 | 0.9285 | 0.9746 | 0.8104 | 0.6871 | 0.9664 | 0.9690 | 0.8179 | 0.8739 | 0.8562 |
| 1441 | 0.8325 | 0.9285 | 0.9746 | 0.8104 | 0.6871 | 0.9664 | 0.9690 | 0.8179 | 0.8739 | 0.8562 |
| 1442 | 0.8325 | 0.9285 | 0.9746 | 0.8104 | 0.6871 | 0.9664 | 0.9690 | 0.8179 | 0.8739 | 0.8562 |
| 1443 | 0.8325 | 0.9285 | 0.9746 | 0.8104 | 0.6871 | 0.9664 | 0.9690 | 0.8179 | 0.8739 | 0.8562 |
| 1444 | 0.8360 | 0.9285 | 0.9746 | 0.8104 | 0.6891 | 0.9664 | 0.9690 | 0.8179 | 0.8746 | 0.8569 |
| 1445 | 0.8360 | 0.9285 | 0.9746 | 0.8104 | 0.6891 | 0.9664 | 0.9690 | 0.8179 | 0.8746 | 0.8569 |
| 1446 | 0.8360 | 0.9285 | 0.9746 | 0.8104 | 0.6891 | 0.9664 | 0.9690 | 0.8179 | 0.8746 | 0.8569 |
| 1447 | 0.8360 | 0.9285 | 0.9746 | 0.8104 | 0.6891 | 0.9664 | 0.9690 | 0.8179 | 0.8746 | 0.8569 |
| 1448 | 0.8360 | 0.9285 | 0.9746 | 0.8104 | 0.6891 | 0.9664 | 0.9690 | 0.8179 | 0.8746 | 0.8569 |
| 1449 | 0.8360 | 0.9285 | 0.9746 | 0.8104 | 0.6891 | 0.9664 | 0.9690 | 0.8179 | 0.8746 | 0.8569 |
| 1450 | 0.8360 | 0.9285 | 0.9746 | 0.8104 | 0.6891 | 0.9664 | 0.9690 | 0.8179 | 0.8746 | 0.8569 |
| 1451 | 0.8360 | 0.9285 | 0.9746 | 0.8104 | 0.6891 | 0.9664 | 0.9690 | 0.8179 | 0.8746 | 0.8569 |
| 1452 | 0.8360 | 0.9285 | 0.9746 | 0.8104 | 0.6891 | 0.9664 | 0.9690 | 0.8179 | 0.8746 | 0.8569 |
| 1453 | 0.8360 | 0.9285 | 0.9746 | 0.8104 | 0.6891 | 0.9664 | 0.9690 | 0.8179 | 0.8746 | 0.8569 |
| 1454 | 0.8360 | 0.9285 | 0.9746 | 0.8104 | 0.6891 | 0.9664 | 0.9690 | 0.8179 | 0.8746 | 0.8569 |
| 1455 | 0.8360 | 0.9302 | 0.9746 | 0.8104 | 0.6910 | 0.9664 | 0.9690 | 0.8179 | 0.8750 | 0.8575 |
| 1456 | 0.8360 | 0.9302 | 0.9746 | 0.8104 | 0.6910 | 0.9664 | 0.9690 | 0.8179 | 0.8750 | 0.8575 |
| 1457 | 0.8360 | 0.9302 | 0.9746 | 0.8104 | 0.6910 | 0.9664 | 0.9690 | 0.8179 | 0.8750 | 0.8575 |
| 1458 | 0.8360 | 0.9302 | 0.9746 | 0.8104 | 0.6891 | 0.9664 | 0.9690 | 0.8179 | 0.8748 | 0.8572 |
| 1459 | 0.8360 | 0.9302 | 0.9746 | 0.8104 | 0.6891 | 0.9664 | 0.9690 | 0.8179 | 0.8748 | 0.8572 |
| 1460 | 0.8360 | 0.9302 | 0.9746 | 0.8104 | 0.6891 | 0.9664 | 0.9690 | 0.8179 | 0.8748 | 0.8572 |
| 1461 | 0.8360 | 0.9302 | 0.9746 | 0.8104 | 0.6910 | 0.9664 | 0.9690 | 0.8179 | 0.8750 | 0.8575 |
| 1462 | 0.8360 | 0.9302 | 0.9746 | 0.8104 | 0.6910 | 0.9664 | 0.9690 | 0.8179 | 0.8750 | 0.8575 |
| 1463 | 0.8360 | 0.9302 | 0.9746 | 0.8104 | 0.6891 | 0.9664 | 0.9690 | 0.8179 | 0.8748 | 0.8572 |
| 1464 | 0.8360 | 0.9302 | 0.9746 | 0.8104 | 0.6891 | 0.9664 | 0.9690 | 0.8179 | 0.8748 | 0.8572 |
| 1465 | 0.8360 | 0.9302 | 0.9746 | 0.8104 | 0.6891 | 0.9664 | 0.9690 | 0.8179 | 0.8748 | 0.8572 |
| 1466 | 0.8360 | 0.9302 | 0.9746 | 0.8086 | 0.6891 | 0.9664 | 0.9690 | 0.8179 | 0.8746 | 0.8569 |
| 1467 | 0.8360 | 0.9302 | 0.9746 | 0.8086 | 0.6891 | 0.9664 | 0.9690 | 0.8179 | 0.8746 | 0.8569 |
| 1468 | 0.8342 | 0.9302 | 0.9746 | 0.8068 | 0.6871 | 0.9664 | 0.9690 | 0.8214 | 0.8744 | 0.8567 |
| 1469 | 0.8342 | 0.9302 | 0.9746 | 0.8086 | 0.6871 | 0.9664 | 0.9690 | 0.8214 | 0.8746 | 0.8569 |
| 1470 | 0.8342 | 0.9302 | 0.9746 | 0.8086 | 0.6871 | 0.9664 | 0.9690 | 0.8214 | 0.8746 | 0.8569 |
| 1471 | 0.8342 | 0.9302 | 0.9746 | 0.8086 | 0.6871 | 0.9664 | 0.9690 | 0.8214 | 0.8746 | 0.8569 |
| 1472 | 0.8342 | 0.9302 | 0.9746 | 0.8068 | 0.6871 | 0.9664 | 0.9690 | 0.8214 | 0.8744 | 0.8567 |
| 1473 | 0.8342 | 0.9302 | 0.9746 | 0.8068 | 0.6871 | 0.9664 | 0.9690 | 0.8214 | 0.8744 | 0.8567 |
| 1474 | 0.8342 | 0.9302 | 0.9746 | 0.8068 | 0.6871 | 0.9664 | 0.9690 | 0.8214 | 0.8744 | 0.8567 |
| 1475 | 0.8342 | 0.9285 | 0.9746 | 0.8068 | 0.6871 | 0.9664 | 0.9690 | 0.8196 | 0.8739 | 0.8562 |
| 1476 | 0.8342 | 0.9285 | 0.9746 | 0.8068 | 0.6871 | 0.9664 | 0.9690 | 0.8196 | 0.8739 | 0.8562 |
| 1477 | 0.8342 | 0.9285 | 0.9746 | 0.8068 | 0.6871 | 0.9664 | 0.9690 | 0.8214 | 0.8741 | 0.8564 |
| 1478 | 0.8342 | 0.9285 | 0.9746 | 0.8068 | 0.6871 | 0.9664 | 0.9690 | 0.8214 | 0.8741 | 0.8564 |
| 1479 | 0.8342 | 0.9285 | 0.9746 | 0.8068 | 0.6891 | 0.9664 | 0.9690 | 0.8214 | 0.8744 | 0.8567 |
| 1480 | 0.8342 | 0.9285 | 0.9746 | 0.8068 | 0.6891 | 0.9664 | 0.9690 | 0.8214 | 0.8744 | 0.8567 |
| 1481 | 0.8342 | 0.9285 | 0.9746 | 0.8068 | 0.6891 | 0.9664 | 0.9690 | 0.8214 | 0.8744 | 0.8567 |
| 1482 | 0.8342 | 0.9285 | 0.9746 | 0.8068 | 0.6891 | 0.9664 | 0.9690 | 0.8214 | 0.8744 | 0.8567 |
| 1483 | 0.8342 | 0.9285 | 0.9746 | 0.8068 | 0.6891 | 0.9664 | 0.9690 | 0.8231 | 0.8746 | 0.8569 |
| 1484 | 0.8342 | 0.9285 | 0.9746 | 0.8068 | 0.6891 | 0.9664 | 0.9690 | 0.8231 | 0.8746 | 0.8569 |
| 1485 | 0.8342 | 0.9285 | 0.9746 | 0.8068 | 0.6891 | 0.9664 | 0.9690 | 0.8231 | 0.8746 | 0.8569 |
| 1486 | 0.8342 | 0.9285 | 0.9746 | 0.8068 | 0.6891 | 0.9664 | 0.9690 | 0.8231 | 0.8746 | 0.8569 |
| 1487 | 0.8342 | 0.9285 | 0.9746 | 0.8068 | 0.6891 | 0.9664 | 0.9690 | 0.8231 | 0.8746 | 0.8569 |
| 1488 | 0.8342 | 0.9285 | 0.9746 | 0.8068 | 0.6891 | 0.9664 | 0.9690 | 0.8231 | 0.8746 | 0.8569 |
| 1489 | 0.8342 | 0.9285 | 0.9746 | 0.8068 | 0.6891 | 0.9664 | 0.9690 | 0.8231 | 0.8746 | 0.8569 |
| 1490 | 0.8342 | 0.9285 | 0.9746 | 0.8068 | 0.6891 | 0.9664 | 0.9690 | 0.8231 | 0.8746 | 0.8569 |
| 1491 | 0.8342 | 0.9285 | 0.9746 | 0.8068 | 0.6891 | 0.9664 | 0.9690 | 0.8231 | 0.8746 | 0.8569 |
| 1492 | 0.8342 | 0.9285 | 0.9746 | 0.8068 | 0.6891 | 0.9664 | 0.9690 | 0.8231 | 0.8746 | 0.8569 |
| 1493 | 0.8342 | 0.9285 | 0.9766 | 0.8068 | 0.6852 | 0.9664 | 0.9690 | 0.8231 | 0.8744 | 0.8567 |
| 1494 | 0.8342 | 0.9285 | 0.9766 | 0.8068 | 0.6852 | 0.9664 | 0.9690 | 0.8231 | 0.8744 | 0.8567 |
| 1495 | 0.8342 | 0.9285 | 0.9766 | 0.8068 | 0.6871 | 0.9664 | 0.9690 | 0.8231 | 0.8746 | 0.8569 |
| 1496 | 0.8342 | 0.9285 | 0.9766 | 0.8068 | 0.6871 | 0.9664 | 0.9690 | 0.8231 | 0.8746 | 0.8569 |
| 1497 | 0.8325 | 0.9285 | 0.9766 | 0.8068 | 0.6871 | 0.9664 | 0.9690 | 0.8231 | 0.8744 | 0.8567 |
| 1498 | 0.8325 | 0.9285 | 0.9766 | 0.8068 | 0.6871 | 0.9664 | 0.9690 | 0.8231 | 0.8744 | 0.8567 |
| 1499 | 0.8342 | 0.9285 | 0.9766 | 0.8068 | 0.6871 | 0.9664 | 0.9690 | 0.8231 | 0.8746 | 0.8569 |
| 1500 | 0.8342 | 0.9285 | 0.9766 | 0.8068 | 0.6871 | 0.9664 | 0.9690 | 0.8231 | 0.8746 | 0.8569 |
| 1501 | 0.8360 | 0.9285 | 0.9766 | 0.8068 | 0.6814 | 0.9664 | 0.9690 | 0.8231 | 0.8741 | 0.8564 |
| 1502 | 0.8360 | 0.9285 | 0.9766 | 0.8068 | 0.6814 | 0.9664 | 0.9690 | 0.8231 | 0.8741 | 0.8564 |
| 1503 | 0.8360 | 0.9285 | 0.9766 | 0.8068 | 0.6814 | 0.9664 | 0.9690 | 0.8231 | 0.8741 | 0.8564 |
| 1504 | 0.8360 | 0.9285 | 0.9766 | 0.8068 | 0.6814 | 0.9664 | 0.9690 | 0.8231 | 0.8741 | 0.8564 |
| 1505 | 0.8360 | 0.9285 | 0.9766 | 0.8068 | 0.6814 | 0.9664 | 0.9690 | 0.8231 | 0.8741 | 0.8564 |
| 1506 | 0.8360 | 0.9285 | 0.9746 | 0.8068 | 0.6814 | 0.9664 | 0.9690 | 0.8231 | 0.8739 | 0.8562 |
| 1507 | 0.8360 | 0.9285 | 0.9746 | 0.8068 | 0.6852 | 0.9664 | 0.9690 | 0.8231 | 0.8744 | 0.8567 |
| 1508 | 0.8360 | 0.9285 | 0.9746 | 0.8068 | 0.6852 | 0.9664 | 0.9690 | 0.8231 | 0.8744 | 0.8567 |
| 1509 | 0.8360 | 0.9285 | 0.9746 | 0.8068 | 0.6852 | 0.9664 | 0.9690 | 0.8231 | 0.8744 | 0.8567 |
| 1510 | 0.8360 | 0.9285 | 0.9746 | 0.8068 | 0.6852 | 0.9682 | 0.9690 | 0.8231 | 0.8746 | 0.8569 |
| 1511 | 0.8360 | 0.9285 | 0.9746 | 0.8068 | 0.6852 | 0.9682 | 0.9690 | 0.8231 | 0.8746 | 0.8569 |
| 1512 | 0.8360 | 0.9285 | 0.9746 | 0.8068 | 0.6852 | 0.9682 | 0.9690 | 0.8231 | 0.8746 | 0.8569 |
| 1513 | 0.8360 | 0.9285 | 0.9746 | 0.8068 | 0.6852 | 0.9682 | 0.9690 | 0.8231 | 0.8746 | 0.8569 |
| 1514 | 0.8360 | 0.9285 | 0.9746 | 0.8068 | 0.6852 | 0.9682 | 0.9690 | 0.8231 | 0.8746 | 0.8569 |
| 1515 | 0.8360 | 0.9285 | 0.9746 | 0.8068 | 0.6871 | 0.9682 | 0.9690 | 0.8214 | 0.8746 | 0.8569 |
| 1516 | 0.8360 | 0.9285 | 0.9746 | 0.8068 | 0.6871 | 0.9682 | 0.9690 | 0.8214 | 0.8746 | 0.8569 |
| 1517 | 0.8360 | 0.9285 | 0.9746 | 0.8068 | 0.6871 | 0.9682 | 0.9690 | 0.8214 | 0.8746 | 0.8569 |
| 1518 | 0.8360 | 0.9285 | 0.9746 | 0.8068 | 0.6929 | 0.9682 | 0.9690 | 0.8214 | 0.8753 | 0.8577 |
| 1519 | 0.8360 | 0.9285 | 0.9746 | 0.8068 | 0.6929 | 0.9682 | 0.9690 | 0.8196 | 0.8750 | 0.8574 |
| 1520 | 0.8360 | 0.9285 | 0.9746 | 0.8050 | 0.6929 | 0.9682 | 0.9690 | 0.8196 | 0.8748 | 0.8572 |
| 1521 | 0.8360 | 0.9285 | 0.9746 | 0.8050 | 0.6891 | 0.9682 | 0.9690 | 0.8196 | 0.8744 | 0.8567 |
| 1522 | 0.8360 | 0.9285 | 0.9746 | 0.8050 | 0.6891 | 0.9682 | 0.9690 | 0.8196 | 0.8744 | 0.8567 |
| 1523 | 0.8360 | 0.9285 | 0.9746 | 0.8050 | 0.6891 | 0.9682 | 0.9690 | 0.8196 | 0.8744 | 0.8567 |
| 1524 | 0.8360 | 0.9285 | 0.9746 | 0.8032 | 0.6891 | 0.9682 | 0.9690 | 0.8196 | 0.8741 | 0.8564 |
